# Seminoma and Embryonal Carcinoma Footprints Identified by Analysis of Integrated Genome-Wide Epigenetic and Expression Profiles of Germ Cell Cancer Cell Lines

**DOI:** 10.1371/journal.pone.0098330

**Published:** 2014-06-02

**Authors:** Yvonne G. van der Zwan, Martin A. Rijlaarsdam, Fernando J. Rossello, Amanda J. Notini, Suzan de Boer, D. Neil Watkins, Ad J. M. Gillis, Lambert C. J. Dorssers, Stefan J. White, Leendert H. J. Looijenga

**Affiliations:** 1 Department of Pathology, Erasmus MC - University Medical Center Rotterdam, Rotterdam, The Netherlands; 2 Centre for Cancer Research, MIMR-PHI Institute of Medical Research, Monash University, Clayton, Victoria, Australia; 3 Centre for Genetic Diseases, MIMR-PHI Institute of Medical Research, Monash University, Clayton, Victoria, Australia; University of Michigan, United States of America

## Abstract

**Background:**

Originating from Primordial Germ Cells/gonocytes and developing via a precursor lesion called Carcinoma *In Situ* (CIS), Germ Cell Cancers (GCC) are the most common cancer in young men, subdivided in seminoma (SE) and non-seminoma (NS). During physiological germ cell formation/maturation, epigenetic processes guard homeostasis by regulating the accessibility of the DNA to facilitate transcription. Epigenetic deregulation through genetic and environmental parameters (i.e. genvironment) could disrupt embryonic germ cell development, resulting in delayed or blocked maturation. This potentially facilitates the formation of CIS and progression to invasive GCC. Therefore, determining the epigenetic and functional genomic landscape in GCC cell lines could provide insight into the pathophysiology and etiology of GCC and provide guidance for targeted functional experiments.

**Results:**

This study aims at identifying epigenetic footprints in SE and EC cell lines in genome-wide profiles by studying the interaction between gene expression, DNA CpG methylation and histone modifications, and their function in the pathophysiology and etiology of GCC. Two well characterized GCC-derived cell lines were compared, one representative for SE (TCam-2) and the other for EC (NCCIT). Data were acquired using the Illumina HumanHT-12-v4 (gene expression) and HumanMethylation450 BeadChip (methylation) microarrays as well as ChIP-sequencing (activating histone modifications (H3K4me3, H3K27ac)). Results indicate known germ cell markers not only to be differentiating between SE and NS at the expression level, but also in the epigenetic landscape.

**Conclusion:**

The overall similarity between TCam-2/NCCIT support an erased embryonic germ cell arrested in early gonadal development as common cell of origin although the exact developmental stage from which the tumor cells are derived might differ. Indeed, subtle difference in the (integrated) epigenetic and expression profiles indicate TCam-2 to exhibit a more germ cell-like profile, whereas NCCIT shows a more pluripotent phenotype. The results provide insight into the functional genome in GCC cell lines.

## Introduction

Type II (testicular) germ cell tumors, here referred to as Germ Cell Cancers (GCC), are the most common malignancy in Caucasian adolescents and young adults, and their incidence is still rising [1]–[3]. GCC originate from primordial germ cells or gonocytes, and are subdivided into seminomas (SE) and non-seminomas (NS), with carcinoma *in situ* (CIS) of the testis as their common precursor lesion [1], also known as Intratubular Germ Cell Neoplasia Unclassified (IGCNU) [3]. In contrast to CIS and SE, the stem cell component of NS (i.e., embryonal carcinoma, EC) is characterized by pluripotent potential [4]. EC can differentiate into somatic lineages and extra–embryonic tissues (teratoma vs yolk sac tumor and choriocarcinoma, respectively), including the germ cell lineage [4]. Various clinical, environmental and genetic risk factors for GCC have been identified, although the exact role of these factors is not completely clear. Clinical risk factors constitute urological/andrological/gonadal aberrations [5]–[8], while environmental factors focus on endocrine disruptors and androgen - estrogen balance [9]–[11]. Genetic risk factors include a number of susceptibility Single Nucleotide Polymorphisms, likely related to early gonadal development [12]–[15] and an association with familial predisposition [16]. Somatic mutations are rarely found in GCC [17]. There are strong indications that the micro-environment of the developing testis is of significant importance in the pathogenesis of GCC. Patients with Testicular Dysgenesis Syndrome (TDS) and specific forms of Disorders of Sex Development (DSD) are known to have an increased risk of developing GCC due to abnormal gonadal development, i.e. hypovirilization [18].

Epigenetic processes have a clear role in both the initiation and protection of pluripotency [19]. Deregulation of these tightly controlled processes is known to be involved in the formation and progression of various cancer types [20]–[24], including GCC [25]. During physiological germ cell formation and maturation, epigenetic processes (e.g. DNA methylation, histone modifications) guard homeostasis by regulating the accessibility of the DNA to facilitate transcription [25], [26]. The epigenome is highly dynamic, and changes occur depending on cell type and developmental stage, influenced by/reflecting the (micro-) environment. In spite of this knowledge, little is known about the role of histone modifications and DNA methylation regarding gene expression in GCC in general, and the possible similarities and differences between SE and EC [25], [27]. Epigenetic deregulation through genetic and environmental parameters (referred to as genvironment) could disrupt physiological embryonic germ cell development, resulting in delayed or blocked maturation, thereby facilitating the formation of CIS, and potentially progression to an invasive GCC [25], [28]–[30]. Therefore, determining the epigenetic and functional genomic landscape in GCC cell lines could provide insight into the pathophysiology and etiology of GCC. The results could provide guidance for targeted functional experiments.

In this study, epigenetic footprints of SE and EC cell lines were identified by studying the interaction between gene expression, DNA methylation and histone modifications. Two well characterized GCC-derived cell lines were used, one representative for SE (TCam-2) [31], [32] and the other for EC (NCCIT) [33]. Two types of epigenetic modifications were investigated and related to genome wide expression analysis: CpG DNA methylation status, and enrichment of activating histone marks (H3K4me3, H3K27ac).

## Methods

### Cell culture

TCam-2 [31], [32], [34] and NCCIT [33] cells were cultured in DMEM medium (#31966-021, Thermo Fisher Scientific/Life Technologies, Carlsbad, CA, USA) containing 10% fetal calf serum (FCS, GE Healthcare Life Sciences, HyClone Laboratories, Utah, USA) in T75 cm^2^ flasks to 75–90% confluence. For RNA preparation, fresh medium was added 24 hours before harvest. Cells were washed once with Hanks balanced Salt Solution (HBSS, #14175-053, Thermo Fisher Scientific/Life Technologies, Carlsbad, CA, USA), and lysed with 7 ml of ice-cold RNA-Bee (#Cs-105B, TEL-TEST Inc, Friendswood, Texas, USA). For methylation, gene expression (biological duplicates) and ChiP-seq analyses, different cultures of cells from a single source were used (LEPO lab, Department of Pathology, Erasmus MC Rotterdam). Biological replicates were started as independent cultures at different days and processed similarly.

### Methylation profiling

DNA was isolated using the DNeasy kit according to manufacturer's instructions (#69504, QIAGEN, Hilden, Germany). Bisulfite conversion (EZ DNA Methylation Gold Kit, Zymo Research, Irvine, CA, USA) and methylation detection was performed at ServiceXS (ServiceXS B.V., Leiden, The Netherlands). Illumina's HumanMethylation450 BeadChip was used (Illumina, Inc., San Diego, CA, U.S.A, processing and hybridization according to the manufacturer's instructions). Image processing took place on the iScan system and the data was extracted using GenomeStudio, using default analysis settings (including background correction and normalization based on internal controls) and v 1.2 of the annotation manifest (http://support.illumina.com/downloads/humanmethylation450_15017482_v12.ilmn). Further processing was carried out in R using the LUMI (http://www.bioconductor.org/) package [35] following the optimized “lumi: QN+BMIQ” pipeline [36] This includes exclusion of poorly performing probes (p<0.01), color adjustment, quantile normalization and correction for probe type bias (Infinium I vs II) using the BMIQ algorithm [37]. All raw and processed data files are submitted as a GEO SuperSeries and accessible via GSE56454 (http://www.ncbi.nlm.nih.gov/geo/). Differentially methylated regions were identified using the DMRforPairs algorithm using the default settings [38]. DMRforPairs is available via Bioconductor: (http://www.bioconductor.org/packages/release/bioc/html/DMRforPairs.html). Briefly, DMRforPairs defines genomic regions using local probe density and optionally functional homogeneity (e.g. all probes in a region should be gene associated). It quantifies, tests and visualizes (differential) methylation patterns between unique samples. Differences were calculated as NCCIT versus TCam-2.

### Gene expression profiling

Approximately 20 µg of RNA was treated with RNase-free DNaseI (#2238, Ambion, Ambion Inc., Austin, TX, U.S.A.) for 30 minutes at 37°C and subsequently purified using the RNeasy mini kit (#74104, Qiagen, Hilden, Germany) according to the manufacturer's instructions. Pure RNA was eluted in 50 µl of water, and quantified using a Nanodrop (Thermo Scientific). Quality control, RNA labeling, hybridization and data extraction were performed at ServiceXS B.V. (Leiden, The Netherlands) according to their in-house protocol. Biotinylated cRNA was prepared using the Illumina TotalPrep RNA Amplification Kit (#AMIL1791, Ambion Inc., Austin, TX, USA) according to the manufacturer's specifications with an input of 200 ng total RNA. Per sample, 750 ng of the biotinylated cRNA was hybridized onto the Illumina HumanHT-12 v4 (Illumina, Inc., San Diego, CA, U.S.A.) according to the Illumina Manual “Direct Hybridization Assay Guide”. Image processing took place on the iScan system and the data was extracted using GenomeStudio (default settings). Further processing was carried out in R using the LUMI package (http://www.bioconductor.org/) [35]. Following the guidelines presented in [39], robust spline normalization was applied to the log2 transformed intensity values. Probes with p_detection_>0.05 in >50% of the samples were excluded from the analysis (n = 27,964 out of 47,323). Average log2 intensities of biological replicates and per gene were used to assess expression levels (GEO accession number GSE56454). Log2 ratios (R) of the average intensities in the two cell lines (NCCIT/TCam-2) were used to identify significantly differentially expressed genes. Genes with expression levels outside the 99% confidence interval (CI) of this log ratio were identified as differentially expressed between NCCIT and TCam-2.

### Histone modification profiling (H3K27ac, H3K4me3)

The ChIP assay was performed according to the low cell number ChIP protocol from Diagenode (Liege, Belgium), with minor modifications. In brief, 1×10^6^ cells were cross-linked for eight minutes by addition of formaldehyde to a final concentration of 1%, followed by neutralization with 1.25 M glycine. The cells were then lysed, and chromatin was sheared to ∼500 bp fragments using the Covaris sonicator under the following conditions; duty cycle 20%, peak incident power 200 watts, cycles/burst 200, time 5 min, temperature 4°C. Protein A-coated Dynabeads (#10002D, Invitrogen, Thermo Fisher Scientific/Life Technologies, Carlsbad, CA, USA) were incubated with 7 µg of the following antibodies: H3K4me3 (Diagenode pAb-003-050) or H3K27ac (Ab4729, Abcam, Cambridge, UK). The beads were combined with chromatin from 1×10^6^ cells overnight on a rotating wheel. The immunobeads were washed, and DNA was purified using the iPure DNA purification kit (AL-100-0100, Diagenode, Liège, Belgium) according to manufacturer's instructions. DNA fragments were sheared a second time using a Covaris sonicator (duty cycle 10%, peak incidence power 175 watts, cycles/burst 200, time 5 minutes, temperature 4°C). Massively parallel sequencing of ChIP DNA (ChIP-Seq) was performed using the 5500xl SOLiD™ sequencing platform (Applied Biosystems, Foster City, CA, USA) at the Monash Health Translation Precinct Medical Genomics Facility. The sequencing experiments were single-end with 50 nt read length (300 nt average fragment size). Sequencing reads were aligned to the complete hg19 human genome (UCSC version, February 2009) using LifeScope™ Genomic Analysis Software v2.5 (http://www.lifetechnologies.com/lifescope). ChIP-Seq experimental samples were normalized to a total of 10^7^ uniquely mapped sequencing tags. Data was processed in HOMER ([40], http://homer.salk.edu/homer/chipseq/) to detect peaks and motif enrichments using the default settings (except “fold enrichment over input”; used 2; threshold for p-value 0.01) (GEO accession number GSE56454). Peak heights from HOMER were corrected for background (lowest peak height detectable). Heights were then summed per gene (ΣP) as annotated by HOMER and genes without any detectable peak were set to 0. The difference in summed peak heights (ΔΣP = ΣP_TCam-2_-ΣP_NCCIT_) was used to quantify differences between the cell lines. Genes with significantly differential histone modification patterns were identified for both marks separately (outside 99% CI of ΔΣP). Association of a peak with TSS was used as annotated by HOMER (1 kb upstream of the TSS - 100 bp downstream).

### MLPA-DNaseI analysis

MLPA probes were designed following previously described criteria [41]. Based on differential modification patterns in NCCIT and TCam-2 probes were designed for the following loci: NCCIT: chr3:181425532-181425720, chr5:146699813-146699953, chr3:181577755-181577830, chr6:15240050-15240160, chr15:93191596-93191696, chr3:178908801-178908966, chr5:101550991-101551125, TCam-2: chr11:10613134-10613220, chr1:201278163-201278251, chr12:3091402-3091483, chr19:13985162-13985322, chr2:38323813-38323931, chr9:843018-843110, chr5:140762378-140762475. MLPA-DNaseI was performed as previously described [42], with minor modifications. In brief, nuclei from 1×10^6^ cells were isolated, and treated with a range of DNaseI concentrations (0; 2 and 5 Units). Digested genomic DNA was purified, and 50–100 ng was used in an MLPA reaction. Following PCR amplification of ligated probes, products were separated on an ABI3700 DNA sequencer. Data was analyzed as previously described, with a reduction to 75% of peak height in undigested DNA used as a threshold for defining DNaseI-hypersensitivity [43].

### Software

Analyses were performed in R 3.0.1 (Windows 7×64) and 2.15.2 (Redhat Linux ×64). The networks/enrichment analyses were performed in IPA (Ingenuity® Systems, www.ingenuity.com). Genomic positions reported in this manuscript are based on the GRch37/hg19 assembly.

## Results

To investigate epigenetic characteristics of SE and EC and their relationship to gene expression, genome-wide histone modification and DNA methylation patterns were investigated in the cell lines TCam-2 (SE) and NCCIT (EC), and matched to gene expression profiles. The differences between the two cell lines with regard to histone modification and DNA methylation status were first investigated separately. Subsequently, the resulting datasets were integrated to identify (target) genes with a strong relationship between primed DNA configuration and higher expression levels.

### Histone modification

Histone modification patterns were assessed using chromatin immunoprecipitation combined with high throughput sequencing (ChIP-seq). Data analysis was performed as described in the materials and methods section. Alterations in H3K4me3 and H3K27ac were investigated, which are markers associated with promoter activation (transcription start site (TSS), H3K4me3 and H3K27ac) and enhancer activation (primarily H3K27ac) [44], [45]. In addition to the analysis of (differential) modification patterns, motif enrichment of the modified regions was investigated and compared between the cell lines.

#### H3K4me3 and H3K27ac do not show differential enrichment near transcription start sites and their peak heights correlated within genes

Depending on the cell line, 10.2/11.3% of the H3K27ac enriched loci were located within 1 kb of a TSS against a comparable 14.7/16.8% of the H3K4me3 loci (TCam-2/NCCIT). This is in line with observations in other cell types showing that, even though H3K4me3 is directly related to promoter activation, a large majority of the H3K4me3 loci are located distally of the TSS [46]. H3K27ac has no reported preferential localization to TSS [47]. The level of summed peaks per gene (ΣP, see Methods) was used to compare histone modification patterns between the two histone marks. There was significant correlation in both cell lines between the peak levels at genes where both were present (ρ_TCam-2_ = 0.62, p_TCam-2_<0.001, n_TCam-2_ = 1837; ρ_NCCIT_ = 0.37, p_NCCIT_<0.001, n_NCCIT_ = 746; Spearman's ρ). This is in line with their overlapping function: open chromatin configuration is associated with both marks [19], [22] and allowed us to combine the histone modification results in the subsequent analysis.

#### H3K4me3 and H3K27ac enrichment patterns in TCam-2 and NCCIT are in accordance with known SE/EC markers specificity

**Figure 1:**
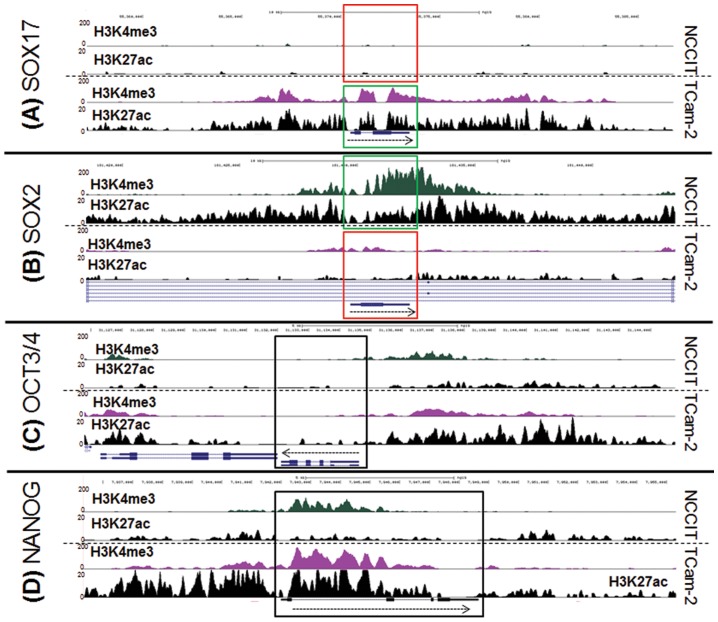
Display of H3K4me3 and H3K27ac tracks for both NCCIT and TCam-2. (A) *SOX17*, (B) *SOX2*, (C) *OCT3/4* (*POU5F1*), (D) *NANOG*. Arrows indicate direction of transcription. Green boxes indicate markers specific for the histological subtype represented by the cell line. Black boxes  =  no difference between the cell lines; red boxes  =  not a marker for that cell type. Note the different ranges on the y-axis for H3K4me3 and H3K27ac.

We previously showed that active chromatin modification patterns for *SOX17* and *SOX2* in the cell lines TCam-2 and NCCIT match the expected pattern, based on gene and protein expression and histological constitution (*SOX17* active in TCam-2, *SOX2* active in NCCIT) [25]. In line with this, *SOX17* and *SOX2* were differentially enriched for both H3K4me3 and H3K27ac in TCam-2 and NCCIT respectively (Figure 1). *OCT3/4* showed no enrichment within the coding sequence, however there was enrichment of both markers close to the TSS in NCCIT and TCam-2. This is consistent with known OCT3/4 mRNA and protein expression in both cell lines [31], [48]. NANOG was more enriched for both markers in TCam-2, in line with differences in expression level (see below).

On a genome-wide scale, 29,428 H3K4me3 enriched loci were identified in TCam-2 and 19,015 in NCCIT. 25–41% more enriched loci were identified for H3K27ac than for H3K4me3 (n_TCam-2_ = 41,569, n_NCCIT_ = 23,763). Genes with significant differences in summed peak height per gene (ΔΣP, see Methods) were selected for further analysis. For TCam-2, genes showing differential histone modifications were higher in number for H3K27ac (n_TCam-2_ = 433, N_NCCIT_ = 28). For NCCIT, there were substantially more genes selected for H3K4me3 compared to H3K27ac (n_TCam-2_ = 215, N_NCCIT_ = 325). For both marks, 86/11 genes overlapped between top differentiating lists in TCam-2 and NCCIT respectively. These included the SE marker *SOX17* in TCam-2 and the EC marker *SOX2* in NCCIT (Figure S1, Table S1). Functionally, the gene lists of both cell lines showed significant enrichment for (embryonic) stem cell maintenance/pluripotency (Table S2). Enrichment of biological functions in TCam-2 indicated similarity to more mature germ cells, which was lacking in the list of NCCIT (GO categories TCam-2 included development of normal testis morphology and germ cell maintenance). Moreover, two germ cell-specific canonical pathways IGF1 signaling (log_p_ = 3.42) and germ cell-Sertoli cell Junction Signaling (log_p_ = 2.11)) showed enrichment. In TCam-2, two functional networks were identified incorporating the AR pathway and lipid metabolism (Table S2).

#### Germ cell markers AP-2α and AP-2γ are top enriched motifs in TCam-2, while embryonic stem cell specific motifs SOX2/OCT4/TCF/NANOG are enriched in both cell lines

**Figure 2:**
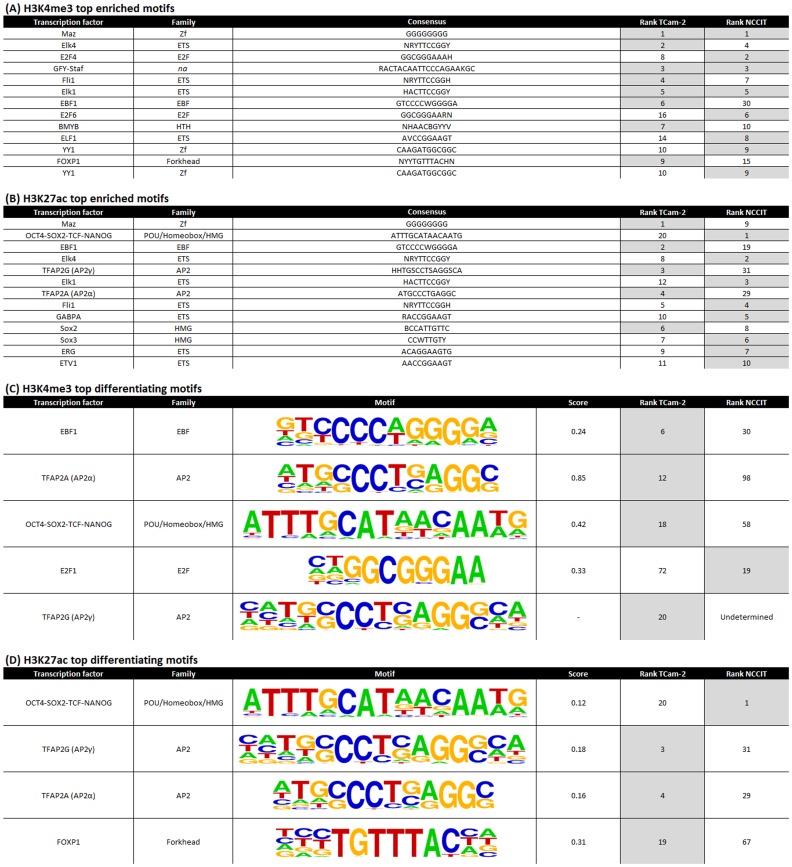
Motif enrichment in histone modification data. All motifs were significantly enriched in target over background sequences (p<0.01). Fold enrichment is indicated relative to background. (A,B) Top ranking motifs in both cell lines showed strong overlap (top 10). (C,D) Motifs that differed strongly between the cell lines with regard to their enrichments were selected. A motif was assessed favorably if its ranking was high (≤20) for one cell line and low for the other cell line (or was absent in the other list of enriched motifs). Score: The difference in ranking was assessed based on the difference in relative position in the list (|1-(r_TCam-2_/n_TCam-2_)–1-(r_NCCIT_/n_NCCIT_)|≥15%, n = nr of enriched motifs, r is the rank of a specific motif in the list of enriched motifs for either cell line).

Significantly enriched motifs were identified for each cell line and histone mark (HOMER tool, see Methods). There was strong overlap between the top-ranked enriched motifs in NCCIT and TCam-2. This was true for both activating markers. For example, for H3K4me3 the top enriched motif was MAZ for both TCam-2 and NCCIT, a transcription factor associated with MYC (binds to two sites in its promoter) and known to be involved in transcription initiation as well as termination [49] (Figure 2A,B; Table S3).

A limited number of markers showed differences in enrichment between the cell lines (Figure 2C,D, Table S3). For H3K4me3, five motifs were identified which showed sufficient difference. Four were higher ranked in TCam-2 and one higher in NCCIT. The top ranked TCam-2 motifs presented in this differentiating list were EBF1 (role in developmental processes), AP-2α and AP-2γ (known germ cell markers) and OCT4/SOX2/TCF/NANOG (pluripotency motif). For NCCIT, E2F1 (cell cycle control, action of tumor suppressor proteins, cell proliferation) was ranked higher. For H3K27ac, there were four differentiating motifs, of which three were ranked higher in TCam-2 and one higher in NCCIT. The OCT4/SOX2/TCF/NANOG motif was the most significantly enriched motif for H3K27ac in NCCIT (ranked 20 in TCam-2). This motif is known to be predominantly enriched in embryonic stem (ES) cells [50] as well as embryonic germ cells, which is in line with the stem cell-like origin (EC) of NCCIT and the germ cell-like origin of TCam-2 respectively. Moreover, for H3K27ac, AP-2α and AP-2γ were ranked as 3^rd^ and 4^th^ most enriched motif in TCam-2 (compared to 29^th^ and 31^st^ in NCCIT) reflecting their (embryonic) germ cell origin (Figure 2C,D). For ES cells the enrichment rankings for these two motifs were 87^th^ and 105^th^ [50]. These observations fit with the proposed more differentiated (germ cell lineage) cell of origin of SE as compared to EC [28], and are in line with the findings of related histone peaks (see Discussion section).

#### Verification of the H3K4me3 & H3K27ac enrichment-based open chromatin configuration was independently confirmed using DNaseI-hypersensitivity

As the investigated chromatin marks (H3K4me3 & H3K27ac) are considered to be associated with active chromatin, we explored whether their presence was associated with another characteristic of active chromatin: DNaseI-hypersensitivity [51]. Using a DNaseI-MLPA approach [43] we targeted 14 regions which showed the greatest differences in either H3K4me3 and/or H3K27ac enrichment between the same two cell lines. Six of seven enriched regions in NCCIT (Figure S2A: N1,N2,N3,N4,N6,N7), and five of seven enriched regions in TCam-2 (Figure S2B: S1,S2,S5,S6,S7), showed significant DNaseI-hypersensitivity in the respective cell lines. In contrast, only one H3K27ac-negative locus in NCCIT (Figure S2A, S1), and no H3K27ac-negative loci in TCam-2 (Figure S2B), showed DNaseI-hypersensitivity.

### CpG Methylation

The DMRforPairs algorithm was used to identify differentially methylated regions (DMR) [38]. The algorithm was set to detect strong differences, i.e. using stringent settings. Regions containing a minimum of four probes within 200 bp distance of each other were considered for further analysis (n = 30,306). Regions in which median methylation levels (M-values) between the samples differed at least |1.4| (n = 5,139) were tested for statistical significance (significant: p<0.05; Bonferroni adjusted, Wilcoxon-rank-sum test, n = 143) (Output DMRforPairs: File S1).

#### Methylation patterns at DMRs are in line with marker positivity in SE and EC

**Figure 3:**
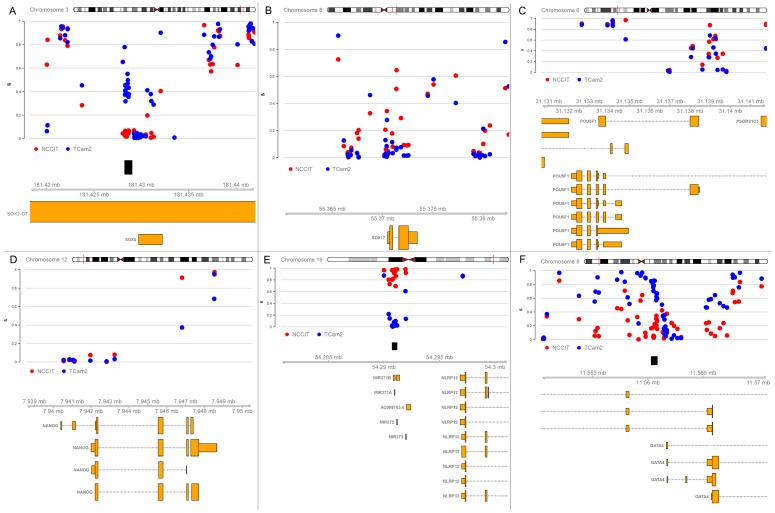
Methylation patterns of known germ cell markers (A–D) and significant DMRs for both cell lines (E, F). Dots depict individual CpGs and black boxes denote DMRs identified by DMRforPairs. Percentages below indicate average CG density in the plotted regions (calculated using the Repitools R package, gcContentCalc function (http://www.bioconductor.org/)). (A) *SOX2* [44%], (B) *SOX17* [46%], (C) *OCT3/4* (*POU5F1*), [52%] (D) *NANOG*, [44%] (E) *miR-371/2/3* cluster, [49%] (F) *GATA4* [53%].

Because of the activating histone modifications investigated, we focused on hypo- or absence of DNA methylation. Global methylation levels were in line with the hyper- and hypomethylated global status of NS and SE respectively, and the previously shown intermediate status of TCam-2 (Figure S3) [52], [53]. After DMR identification using DMRforPairs, a total of 99 DMRs (annotating to 170 unique gene symbols) were hypomethylated in TCam-2, compared to 44 in NCCIT (annotating to 64 unique gene symbols) (Table S1). In line with the histone modifications (see above), the *SOX2* promoter region was found to be strongly hypomethylated in NCCIT (Figure 3A). *SOX2* was partly methylated in TCam-2, in line with findings illustrating that TCam-2 can differentiate and become *SOX2* positive after extra-gonadal injection in mice [54]. A 220 bp region directly upstream of the TSS of *SOX2* (chr3:181429712) has previously been shown to be completely hypomethylated in TCam-2 [55]. This is in line with our findings as a consistently hypomethylated region (chr3:181429233-181430485) is shown directly upstream of the TSS while a 652 bp long DMR (chr3:181428046-181428697) between NCCIT and TCam-2 is detected by DMRforPairs in a region ca. 800 bp upstream of the region sequenced by Nettersheim et al. *SOX17* did not show a significant differential methylation pattern, indicating that it is, in principle, accessible for transcription in both cell types (Figure 3B). Indeed, *SOX17* expression can be induced in NCCIT (unpublished observation). In line with known gene expression in both cell lines, *OCT3/4* showed an inconsistent, but non-differential methylation pattern (Figure 3C). The TSS of *NANOG* was hypomethylated in both cell lines (Figure 3D), in line with the expression data and previous reports [56]. In the list of top DMRs, the miR-371/2/3 cluster stood out by significant differential hypermethylation in NCCIT (Figure 3E). The promoter region of *GATA4* was significantly hypermethylated in TCam-2 (Figure 3F). In general, 33% (47/143) DMRs were annotated to TSSs (File S1, according to Illumina's manifest) which is similar to the fraction of TSS associated regions identified by DMRforPairs (12,652/30,306). Functionally, the DMR list of both cell lines showed enrichment for (embryonic) stem cell maintenance/pluripotency. Biological functions indicating similarity to more mature germ cells were enriched in TCam-2 (Table S2).

#### DMRs were significantly enriched for imprinted genes, and 59% (51/86) of all imprinted genes showed loss of methylation around their TSS in one or both cell lines

**Figure 4:**
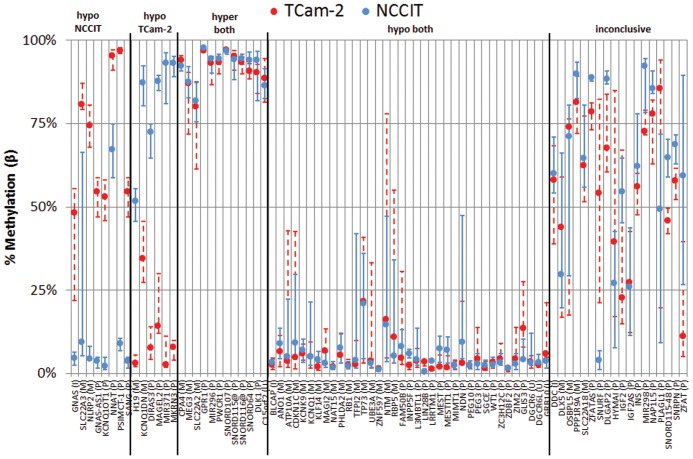
Methylation patterns in promoter regions of imprinted genes. Localization of TSS was retrieved from Ensembl and manually corrected for genes with multiple transcripts to select a region with representative coverage on the Illumina BeadChip (n_probes_ varied between the genes: median = 10, inter-quartile range 6–21). Promoter region was defined as TSS-1000–TSS+100 (or opposite on reverse strand). Genes with >0.25 difference in median β and a consistent (stable) methylation pattern were identified as differentially methylated at the TSS between the two cell lines. Median methylation <25% for both cell lines was interpreted as a hypomethylated state in both cell line. Median methylation >75% was interpreted as hypermethylation. No probes were annotated around the TSS of *TCEB3C* (M), *RNU5D* (P), *SNORD108* (P), SNORD109A (P) and *SNORD109B* (P). *SANG* and *GNAS-AS1* are known aliases but separate entities on geneimprint.com and are depicted separately to preserve consistency. P = paternally imprinted/expressed, M = maternally imprinted/expressed, I = isoform-dependent imprinting.

From the list of verified imprinted human genes (n = 88 retrieved from geneimprint.com), 82 were also annotated in Illumina's manifest (total of 21,243 unique gene symbols annotated) and 10 were present in the top DMRs between TCam-2 and NCCIT. This overrepresentation of imprinted genes in the list of DMRs was significant (p<0.0001, χ^2^ test). When investigating the region surrounding the TSS of the 86 imprinted genes with known genomic localization, 14 showed a differential status between the cell lines (8/6 hypomethylated in NCCIT and TCam-2 respectively). In total, 37 imprinted genes displayed a hypomethylated status in both cell lines, compared with 12 hypermethylated genes (Figure 4). In summary, these results indicate an overall erased status of the imprinted regions in both cell lines. Regarding genes with a differential methylation status, there is no clear difference in number of hypermethylated genes that would indicate a difference in maturation status or environmental disruption.

### Expression

#### Expression levels of markers matched histological origin of both cell lines

**Figure 5:**
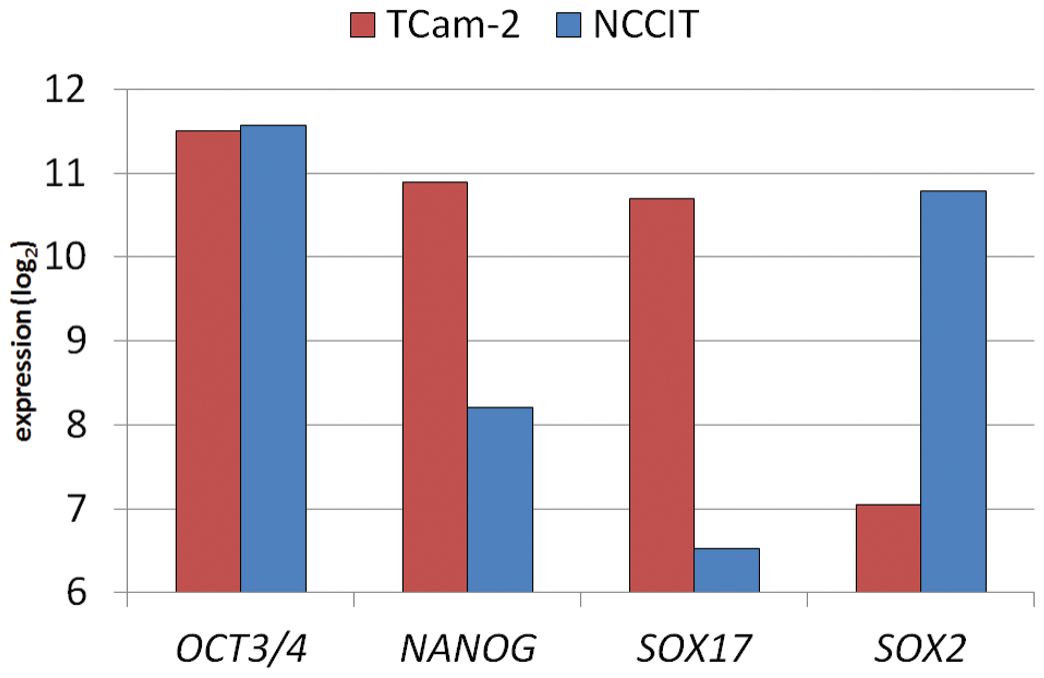
Expression levels of known GCC markers match the histological subtype from which the cell lines originate.

In total, 257 genes were expressed higher in TCam-2, compared to 149 in NCCIT (Table S1). Greater than 3.65 fold difference in expression level (99% confidence interval (CI) of log2-ratio of intensities) was considered significant. The expression levels were in agreement with the classification of the cell lines: *SOX17* was higher in TCam-2 compared with NCCIT, with the opposite observed for *SOX2* [57] (Figure 5). *OCT3/4*, a general marker for the stem cell components (SE/EC) of GCC, was expressed at equal levels in both cell lines. *NANOG* was expressed higher in TCam-2, which is in line with the open histone configuration (Figure 1D, transcription possible in both cell lines). Functionally, the gene lists of both cell lines showed enrichment for (embryonic) stem cell maintenance/pluripotency, and Wnt/β-catenin signaling. Enrichment of biological functions consistent with more mature germ cells was present in TCam-2, and absent in NCCIT. In addition, network analysis revealed the androgen pathway in TCam-2, represented by both the AR and testosterone, thus showing major overlap between the networks found by genes that had differential histone modification patterns (Table S2).

### Integration of epigenetic and expression data

#### Differential gene lists from histone modification, methylation and expression data showed limited overlap

**Figure 6:**
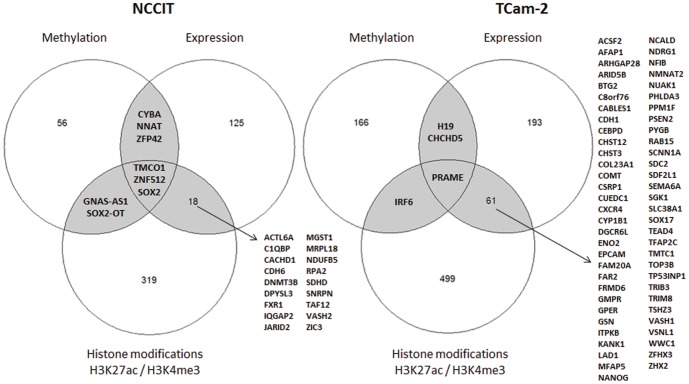
Overlap between top differentiating genes (methylation/histone modification/gene expression). (Hypo) methylation, (high) gene expression and histone marker (enrichment) should be interpreted relative to the other cell line. Criteria for selection are described in the main text. Briefly, significant differential methylation of regions with sufficient probe density was identified by DMRforPairs (frequently, but not necessarily close to, the TSS). The difference in histone modification enrichment was assessed by significant differences in summed peak heights between the cell lines. Finally, a fold difference of 3.65 (boundary of 99% CI) was used as cutoff for differential gene expression. Gene lists are presented in Table S1, and overlap was determined based on matching gene symbol.

Differentiating gene lists from the separate analyses discussed above were matched based on Gene Symbol to assess the relationship between active histone modifications, the absence of CpG methylation and gene expression. Overlap between gene expression and one of the epigenetic regulatory mechanisms is of interest as expression of a specific gene does not need to be regulated by both mechanisms. Figure 6 shows the overlap of the different variables for the differentiating gene lists between TCam-2 and NCCIT. In general, little overlap between relative hypomethylation/histone marker enrichment and relatively high expression is observed, but this overlap was significant (Figure S4). In TCam-2, one gene, *PRAME*, was present in all three differential lists. *H19* and *CHCHD5* were differentially hypomethylated in TCam-2 and showed high expression compared to NCCIT, but no differential enrichment for H3K27ac or H3K4me3. There were 62 genes with overlapping active chromatin marks and expression, including *SOX17* and *NANOG*. In NCCIT, three genes showed overlap between the histone marks, hypomethylation and expression. Significantly, one of these genes was *SOX2*. An additional 18 genes showed overlap between active chromatin marks and expression.

#### Enrichment of both histone marks in general and absence of DNA methylation around the TSS is related to higher expression levels

**Figure 7:**
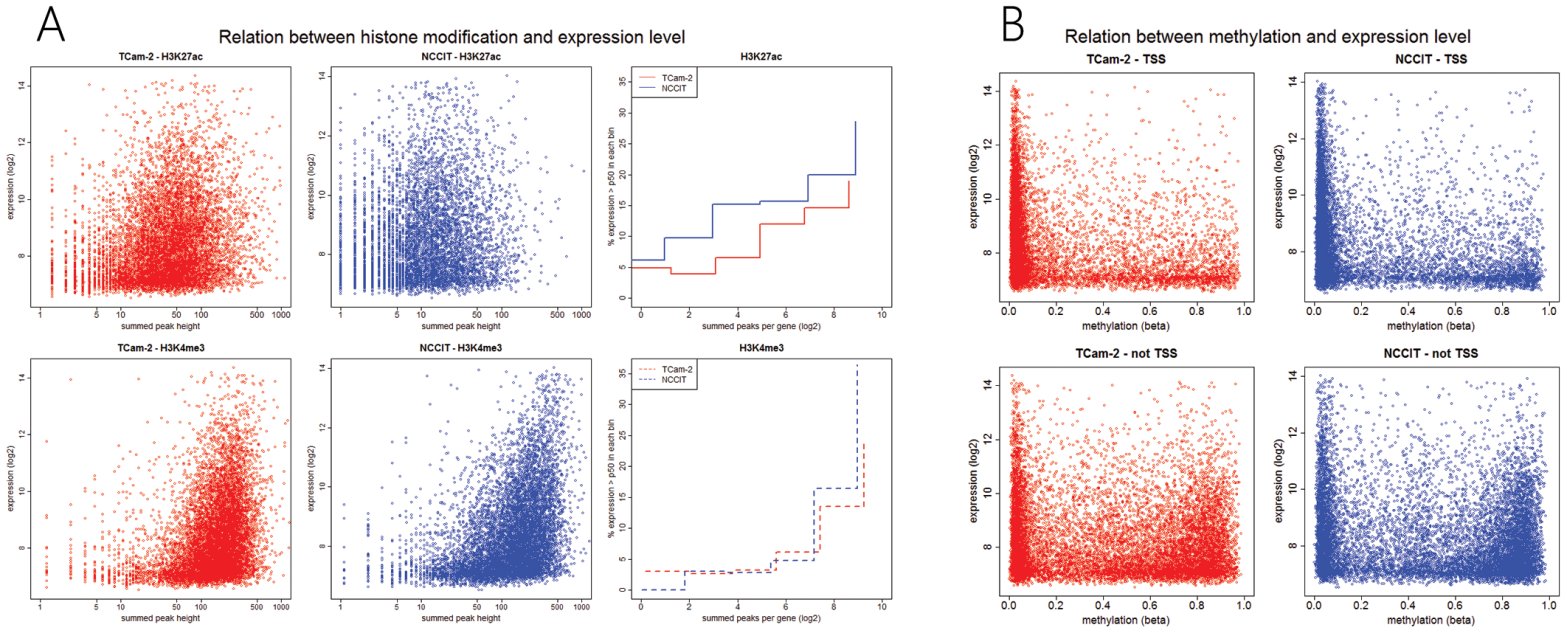
Relation between histone modification level (summed peak heights per gene) and expression level. Top and bottom right images depict the percentage of highly (>p50) expressed genes calculated for an interval of summed peaks. For example, 5% of genes with a log2(summed peak height) of ≈5.5–7.5 were highly expressed. (B) Relation between CpG methylation (TSS/no TSS) and gene expression.

Higher expression levels are present when genes are more enriched for either histone mark. Quantification of the fraction of genes with higher than median expression at various intervals of summed peak heights confirmed the general trend towards higher expression at higher levels of enrichment (Figure 7A). DNA methylation levels were only correlated to expression around the TSS: a fully methylated TSS is predominantly associated with low gene expression levels while low methylation status is not predictive of expression level (Figure 7B).

#### NCCIT and TCam-2 show a largely overlapping epigenetically open network with specific elements that are differentially regulated based on cell type

**Figure 8:**
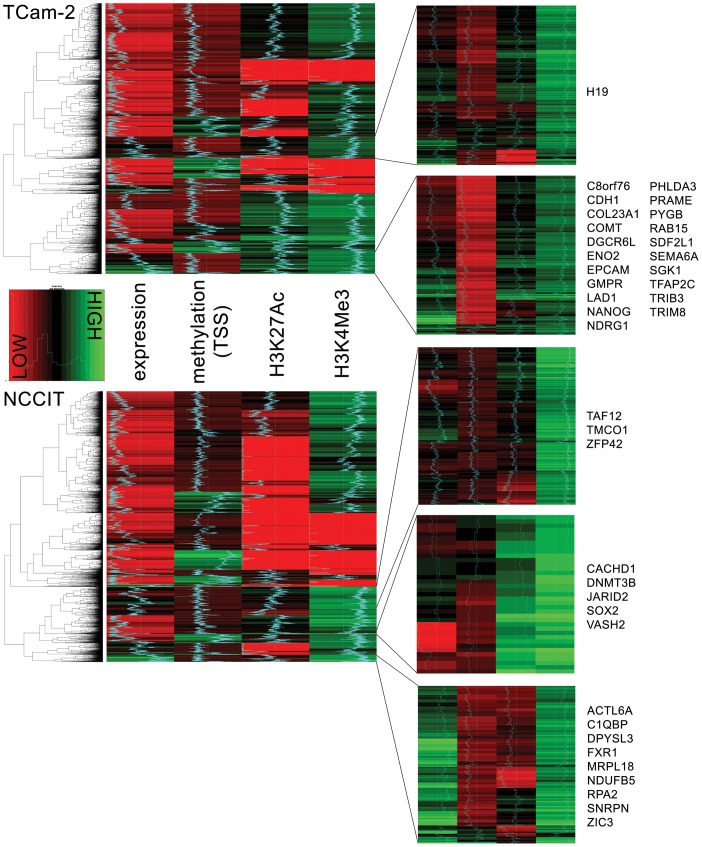
Heat map of epigenetic markers and gene expression profiles. Genes with quantified methylation status around their TSS (based on Illumina annotation) and valid (see Materials & Methods) measurement of their expression level were included (n = 11,620). Log-2 summed peak heights per gene were used as an estimate of histone marker enrichment. Variables scaled between 0 and 1. Hierarchical clustering was performed using complete linkage. Clusters of interest were identified based on a consistent enriched state for one or more of the active histone markers and a hypomethylated state around the TSS. Number of genes in the displayed right panel (zoomed in heat maps, top→bottom): 899/892 (TCam-2) and 1,224/37/308 (NCCIT). Gene expression was allowed to vary within clusters, but clusters with almost absent expression levels (completely red) were not selected. Gene symbols indicate genes that overlap with the analysis of top differentiating genes between the cell lines (Figure 6). Gene symbols are listed alphabetically. An indication of the level for each gene in each column is presented by the color/shade and a blue line (for each column: left = 0, right = 1).

Histone modification, methylation and gene expression data were analyzed together using hierarchical clustering. This unsupervised clustering procedure revealed specific groups of genes with a profile poised for transcription. These gene clusters showed active histone marks, combined with an activating methylation landscape and are hypothesized to contain genes accessible for transcription (e.g. epigenetically ON = transcription possible, Figure 8). Functionally, the ON network for both cell lines showed a large degree of overlap (related to the androgen pathway, lipid metabolism and pluripotency) (Table S2). There was considerable overlap between the genes found in this separate analysis and the differential gene lists (Figure 6, overlap indicated by gene symbols in Figure 8).

## Discussion

Histone and DNA methylation signatures were studied to explore the epigenetic differences between representative cell lines for the GCC histological subtypes and their relation to expression. TCam-2 and NCCIT cells were used as representatives of SE and EC respectively. Our study includes the generation, integration and interpretation of genome-wide profiles for histone marks (H3K4me3 and H3K27ac), DNA methylation and gene expression. H3K4me3 and H3K27ac are well-characterized markers for active promoter and enhancer sites, respectively. Analysis of the histone marks matched the classification of the cell-lines: *SOX17* was strongly enriched for H3K4me3 and H3K27ac in TCam-2 compared to NCCIT cells, whereas the opposite pattern was observed for *SOX2*. Motifs for germ cell-specific transcription factors AP-2α/γ were enriched in TCam-2, but not in NCCIT. Methylation profiling showed *SOX2* to be in the top DMRs, being more methylated in TCam-2 as expected. In addition, *SOX2* and *SOX17* expression levels confirmed and matched the previously described patterns in SE and EC [2]. General SE/EC markers (*NANOG* and *OCT3/4*) showed expression patterns compatible with their epigenetic configurations [58]. Moreover, imprinting patterns confirm the suggested overall erased status of genomic imprinting in both cell lines, in line with the situation in early germ cells [59].

The cell lines were studied for their differences with regard to epigenetic marks and expression. In line with the early germ cell origin of both cell lines (NCCIT more stem cell-like than TCam-2) canonical pathways related to stem cell maintenance and regulation were significantly overrepresented for all three variables studied (NCCIT more pronounced than TCam-2). In TCam-2 (differential histone modification) there was strong overrepresentation of genes involved in IGF1 signaling, a pathway that is implicated in maintenance of spermatogonia [60]. Moreover, genes involved in germ cell – Sertoli cell junction signaling were significantly overrepresented in this cell line, fitting with a more mature type of germ cell depending on the Sertoli cell niche [2]. In the list of top DMRs, the miR-371/2/3 cluster stood out by significant differential hypermethylation in NCCIT. These miRs have shown to be specific biomarkers for GCC in serum and tumor tissue [61]–[65]. Even though GCCs are reported to express these embryonic miRs [63], [66], [67], NCCIT has been shown to exhibit low expression levels due to the absence of a functional TP53 pathway (i.e. lacking the need to inactivate this pathway by miR-372/3 expression via LATS2 inhibition) [67].

Pathway analysis using IPA revealed a network including the androgen receptor (AR) and testosterone targets enriched for open chromatin configuration marks in TCam-2. Such enrichment was also identified based on the expression data of TCam-2, and to a lesser extent in NCCIT. Despite of this observation, no differential AR expression was present between the cell lines, and no enrichment of DMRs in AR targets was identified. Future experiments are needed to validate the differential role of the AR pathway between NCCIT and TCam-2 and their *in vivo* counterparts. A second network was closely related, focused on lipid metabolism (LEP central, late germ cell differentiation and survival [68]). Androgens are well-known environmental and physiological factors that influence epigenetic marks and phenotypes. They are related to GCC risk and are considered to be crucial for the progression of germ cell development [28] Ammerpohl *et al.* found significant enrichment of hypermethylated AR target genes in androgen insensitivity syndrome (AIS) patients versus controls [69]. This is consistent with earlier reports that diminished gene activation (due to an *AR* mutation) results in subsequent increased DNA methylation of target genes [70], [71], linking DSD and GCC at the epigenetic level.

On the other hand, functional analysis for NCCIT predominantly revealed genes involved in embryonic stem cell maintenance (less pronounced in TCam-2). More specifically, an interaction between *SOX2* and *DMRT1* was the most important network identified. Both are involved in stem cell maintenance in embryonic (mouse) germ cells [28]. It has been reported that Dmrt1 can bind the mouse Sox2 promoter, and Dmrt1 controls expression of Sox2 and other pluripotency genes (such as Nanog and Oct3/4) in the embryonic testis, in part via transcriptional repression [72]. In addition, *DMRT1* has a role in sex determination, as it prevents female reprogramming in the postnatal mammalian testis [73]. A study by Murphy et al confirmed the influence of *DMRT1* on *SOX2* expression while no change in expression of *SOX17* was observed [74]. However, these observations in mice are not necessarily representative for the human situation. No consistent networks related to progression of germ cell differentiation (as with AR in TCam-2) were identified in NCCIT.

As stated above, motif enrichment based on the histone modification data showed that the AP-2α and AP-2γ motifs were enriched in TCam-2 only. The ETS family was enriched in both TCam-2 and NCCIT. AP-2γ is a known germ cell marker, abundantly expressed in CIS and SE, and heterogeneously expressed in NS and somatic tumors [75], [76]. *AP-2y* and *KIT* are co-expressed in gonocytes [76], which could point to a direct regulation loop which supports proliferation, and agrees with the observation that TCam-2 is more germ cell-like than NCCIT. AP-2y would then serve as a molecule that keeps fetal germ cells in a pluripotent state by suppressing differentiation and supporting proliferation [76], [77]. *AP-2y* expression is induced by estrogens [78] and *AP-2α* and *AP-2y* are able to induce changes in the chromatin structure known to be associated with *ERα* (*ESR1*) transcription [79]. The importance of the androgen-estrogen balance is also indicated by the strongly androgen/estrogen-centered gene networks identified in this study. Additionally, the ETS family was present in the top motif enrichments for both TCam-2 and NCCIT. Recently it was shown that overexpression of ETS, combined with loss of *PTEN*, increases AR binding and restores AR transcriptional activity in prostate [80]. Indeed, disruption of the PTEN pathway has been suggested to be part of the pathogenesis of GCC [81].

## Conclusions

In conclusion, this study provides an integrated analysis of the functional genome in GCC cell lines. Our data show that known germ cell markers are not only present and differentiating between SE and NS at the expression level, but also in the epigenetic landscape. The overall similarity between TCam-2/NCCIT support an erased embryonic germ cell arrested in early gonadal development as common cell of origin although the exact developmental stage from which the tumor cells are derived might differ. Indeed, subtle difference in the (integrated) epigenetic and expression profiles indicate TCam-2 to exhibit a more germ cell-like profile, whereas NCCIT shows a more pluripotent phenotype. Future research has already been initiated to investigate primary cancer samples from patients to confirm and further expand the integrated epigenetic EC and SE footprints identified in this study.

## Supporting Information

Figure S1 Number of top differentially modified regions between TCam-2 and NCCIT, and their overlap between H3K27ac and H3K4me3 based on associated genes. Genes with significant differences (outside 99% confidence interval) in summed peak height per gene (ΔΣP) were identified as top-differentially modified.
(TIF)

Figure S2 Normalized ratios for each of the 14 loci tested in the MLPA-DNaseI assay. A threshold of <0.75 was defined for DNaseI-hypersensitivity. N = enriched in ChIP-seq analysis in NCCIT (non-seminoma cell model), S = enriched in ChIP-seq analysis in TCam-2 (seminoma model). (A) Analysis of NCCIT cells. (B) Analysis of TCam-2 cells. (C) Overview of loci and interpretation of results. DNaseI hypersensitivity is indicated if present in the cell line in which marker enrichment was also found in the ChIP-seq analysis.
(TIF)

Figure S3 Visualization of global methylation patterns in both cell lines. Depicted is a violin plot of the distribution of methylation values (β) for both cell lines. In general, NS are considered globally hypermethylated in comparison to SE but TCam-2 is known to show an intermediate phenotype with regard to global methylation status (see Wermann et al 2010 and Netto et al 2008 in reference list). Indeed, significantly lower methylation levels were detected in TCam-2 but the quantitative difference in methylation distribution was very moderate (p<0.01, Mann Whitney U test, median_β_ (1^st^–3^rd^ quantile_β_): 51%_TCam-2_ (46%–84%) versus 63%_NCCIT_ (58%–87%)).
(TIFF)

Figure S4 Venn diagrams analogous to Figure 6 corrected for gene symbols that are not represented by valid measurements in the expression or methylation data (histone modification  =  genome wide assessment). NCCIT: 33 genes differentially methylated were not annotated in the expression data. 17 overexpressed genes were not annotated in the methylation data. 97 genes showing differential histone modifications were not present in the expression or methylation data. For TCam-2 these numbers were 101/28/198. Based on an empirical probability distribution we assessed random overlap using 10,000 draws from simulated genelists with n_expression (EXPR)_ = 14,525, n_histone-modification (HM)_ = 22,000 and n_methylation (MEHTY)_ = 21,243 genes. These numbers correspond with the number of genes with valid measurements on the arrays (histone modification: genome wide proxy). Significant overlap indicates more overlapping genes identified in these venn diagrams than would we expected based on random subsets of genes. Significant overlap is indicated with a * (p<0.05). p-values TCam-2: p_EXPR_HM_ = <0.0001, p_EXPR_METHY_ = 0.0370–0.1604, p_HM_METHY_ = 0.3229–0.6860, p_all3_ = 0.0003–0.0167. p-values NCCIT: p_EXPR_HM_ = 0–0, p_EXPR_METHY_ = 0.0001–0.0007, p_HM_METHY_ = 0.3110–1.0000, p_all3_ = <0.0001. (P-values are ranges if in the repeated random draws used to construct the empirical cumulative distribution function a specific count of overlapping genes occurred more than once).
(TIF)

Table S1 Top differentiating genes in histone modification, CpG methylation and gene expression analyses.
(XLSX)

Table S2 Results of IPA/functional analysis of (differentiating) gene lists. IPA was performed using the default settings including “testis” as specific tissue of interest, and only incorporating experimentally observed evidence. Green fill indicates overlap between TCam-2 and NCCIT. Reported log(p) values are the result of IPA's internal enrichment tests.
(XLSX)

Table S3 Detailed results of motif enrichment analysis (HOMER) per cell line and per histone mark.
(PDF)

File S1 ZIP file containing DMRforPairs output for significant regions. Please start from the html files.
(ZIP)
