# Supplementary figures and images for: Seminoma and Embryonal Carcinoma Footprints Identified by Analysis of Integrated Genome-Wide Epigenetic and Expression Profiles of Germ Cell Cancer Cell Lines (part 1 of 2)
Source: PLoS One. 2014 Jun 2;9(6):e98330. doi: 10.1371/journal.pone.0098330 (PMC4041891; doi:10.1371/journal.pone.0098330)

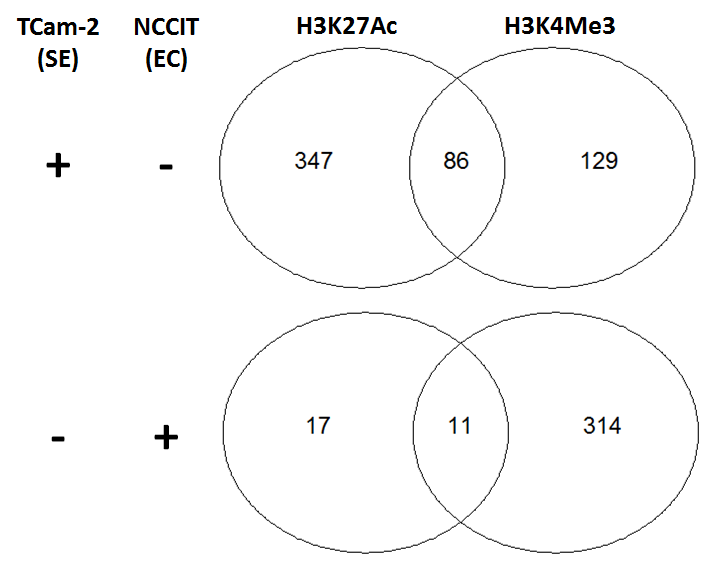

Supplement: Figure S1 — Number of top differentially modified regions between TCam-2 and NCCIT, and their overlap between H3K27ac and H3K4me3 based on associated genes. Genes with significant differences (outside 99% confidence interval) in summed peak height per gene (ΔΣP) were identified as top-differentially modified. (TIF) [file pone.0098330.s001.tif]

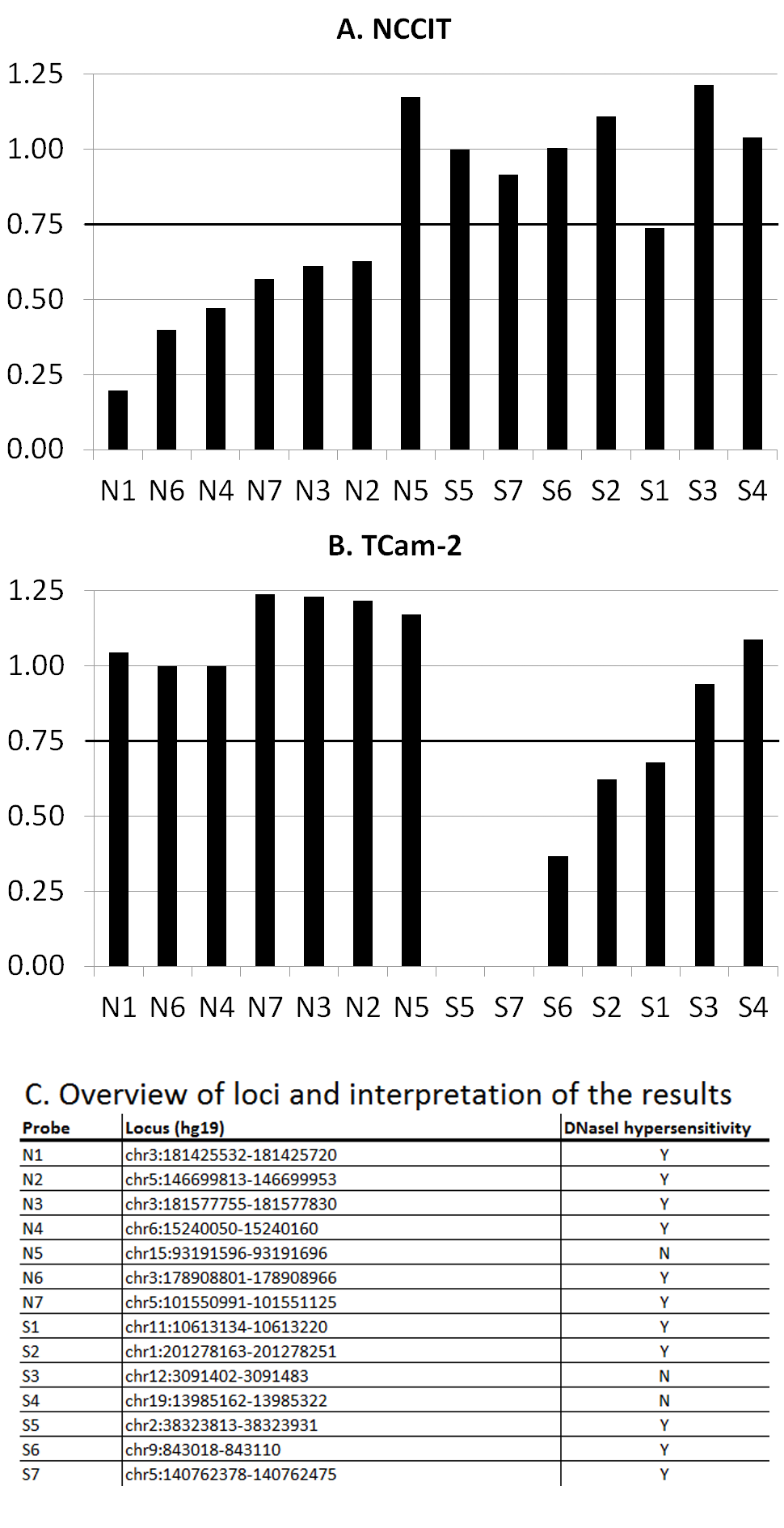

Supplement: Figure S2 — Normalized ratios for each of the 14 loci tested in the MLPA-DNaseI assay. A threshold of <0.75 was defined for DNaseI-hypersensitivity. N = enriched in ChIP-seq analysis in NCCIT (non-seminoma cell model), S = enriched in ChIP-seq analysis in TCam-2 (seminoma model). (A) Analysis of NCCIT cells. (B) Analysis of TCam-2 cells. (C) Overview of loci and interpretation of results. DNaseI hypersensitivity is indicated if present in the cell line in which marker enrichment was also found in the ChIP-seq analysis. (TIF) [file pone.0098330.s002.tif]

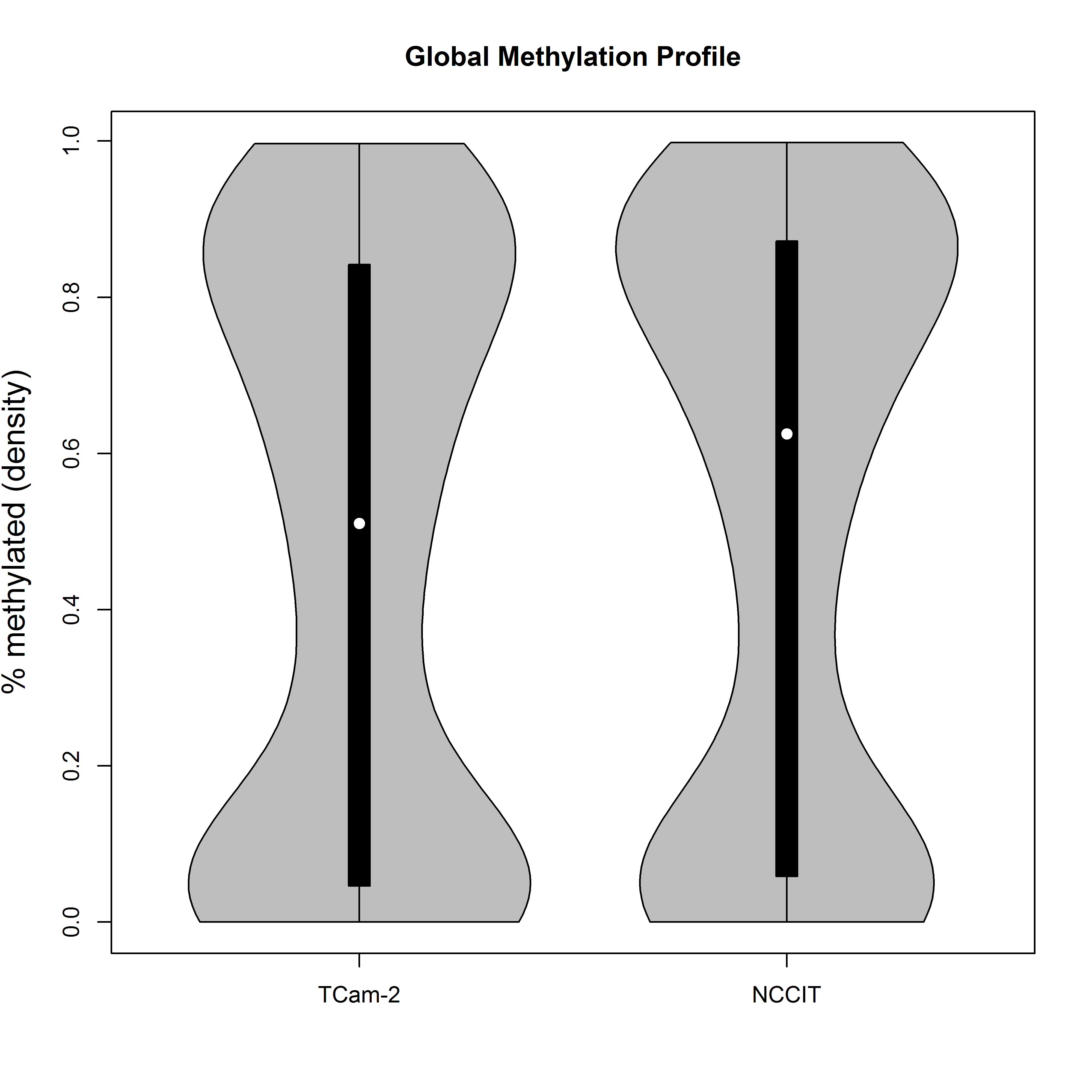

Supplement: Figure S3 — Visualization of global methylation patterns in both cell lines. Depicted is a violin plot of the distribution of methylation values (β) for both cell lines. In general, NS are considered globally hypermethylated in comparison to SE but TCam-2 is known to show an intermediate phenotype with regard to global methylation status (see Wermann et al 2010 and Netto et al 2008 in reference list). Indeed, significantly lower methylation levels were detected in TCam-2 but the quantitative difference in methylation distribution was very moderate (p<0.01, Mann Whitney U test, medianβ (1st–3rd quantileβ): 51%TCam-2 (46%–84%) versus 63%NCCIT (58%–87%)). (TIFF) [file pone.0098330.s003.tiff]

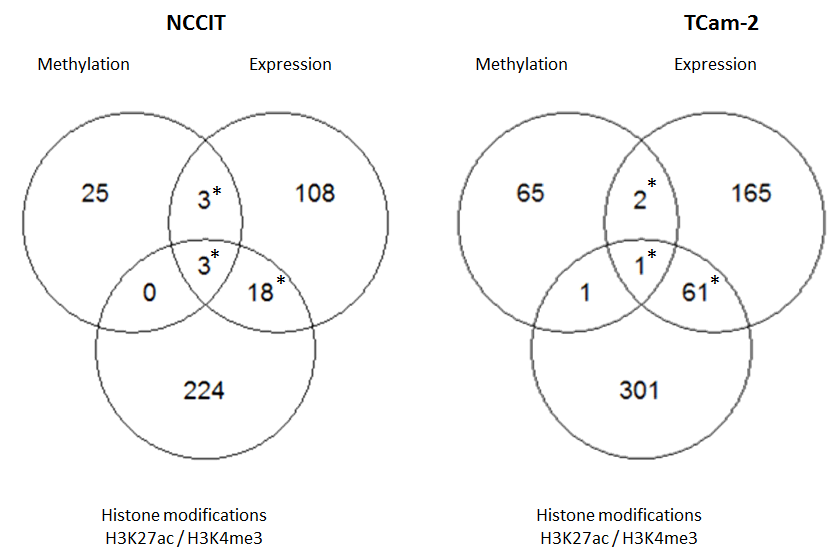

Supplement: Figure S4 — Venn diagrams analogous to Figure 6 corrected for gene symbols that are not represented by valid measurements in the expression or methylation data (histone modification = genome wide assessment). NCCIT: 33 genes differentially methylated were not annotated in the expression data. 17 overexpressed genes were not annotated in the methylation data. 97 genes showing differential histone modifications were not present in the expression or methylation data. For TCam-2 these numbers were 101/28/198. Based on an empirical probability distribution we assessed random overlap using 10,000 draws from simulated genelists with nexpression (EXPR) = 14,525, nhistone-modification (HM) = 22,000 and nmethylation (MEHTY) = 21,243 genes. These numbers correspond with the number of genes with valid measurements on the arrays (histone modification: genome wide proxy). Significant overlap indicates more overlapping genes identified in these venn diagrams than would we expected based on random subsets of genes. Significant overlap is indicated with a * (p<0.05). p-values TCam-2: pEXPR_HM = <0.0001, pEXPR_METHY = 0.0370–0.1604, pHM_METHY = 0.3229–0.6860, pall3 = 0.0003–0.0167. p-values NCCIT: pEXPR_HM = 0–0, pEXPR_METHY = 0.0001–0.0007, pHM_METHY = 0.3110–1.0000, pall3 = <0.0001. (P-values are ranges if in the repeated random draws used to construct the empirical cumulative distribution function a specific count of overlapping genes occurred more than once). (TIF) [file pone.0098330.s004.tif]

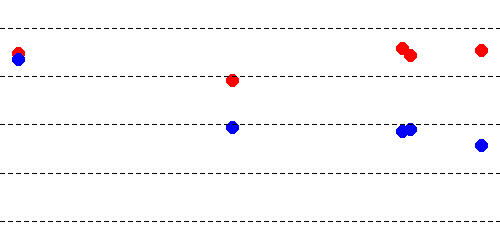

Supplement: File S1 — ZIP file containing DMRforPairs output for significant regions. Please start from the html files. (ZIP) [file pone.0098330.s008.zip › figures/20.png]

RegionID: 20, chr1:1141617-1141991-M\_values

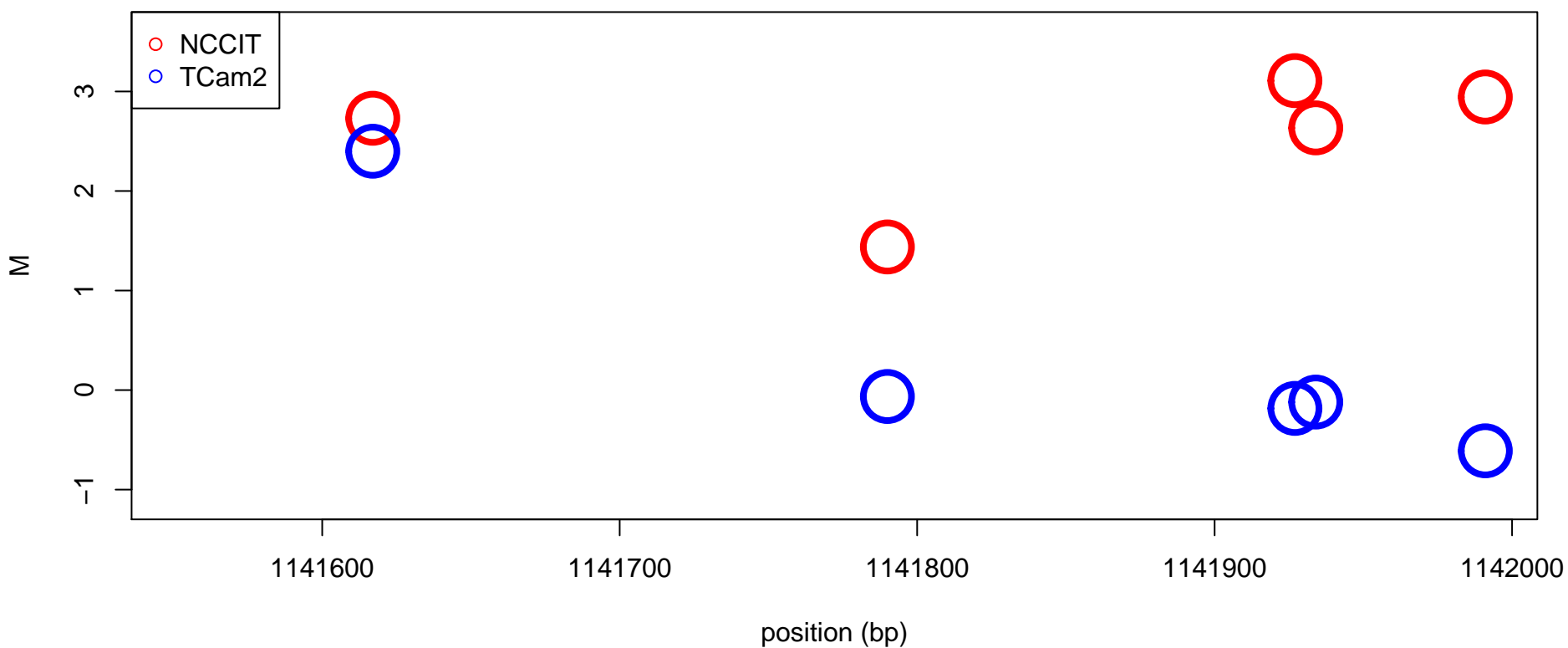

RegionID: 20, chr1:1141617-1141991-Beta\_values

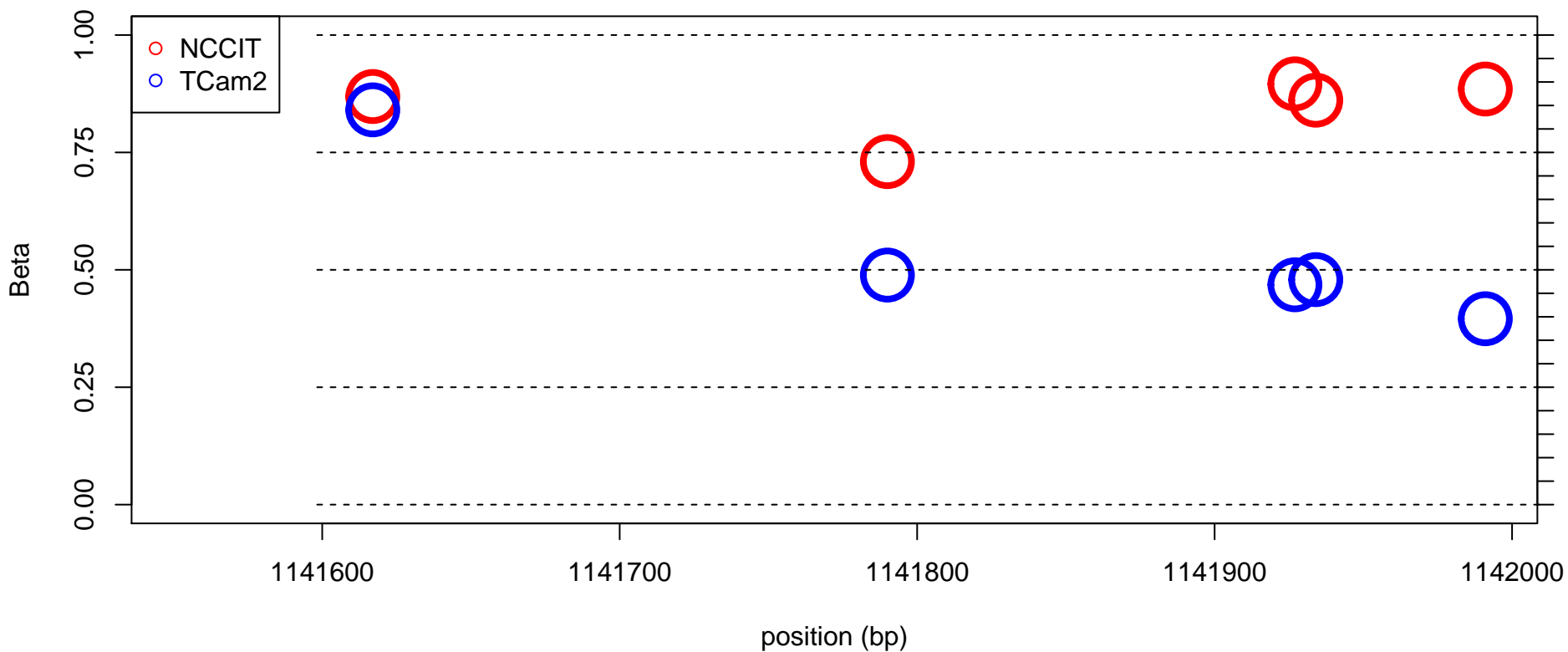

Supplement: File S1 — ZIP file containing DMRforPairs output for significant regions. Please start from the html files. (ZIP) [file pone.0098330.s008.zip › figures/20.pdf]

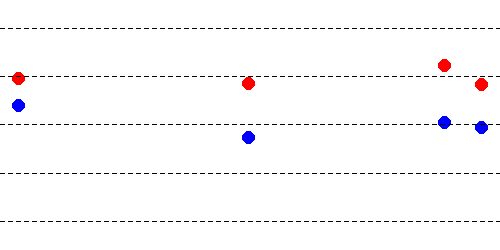

Supplement: File S1 — ZIP file containing DMRforPairs output for significant regions. Please start from the html files. (ZIP) [file pone.0098330.s008.zip › figures/21.png]

RegionID: 21, chr1:1148973–1149211–M\_values

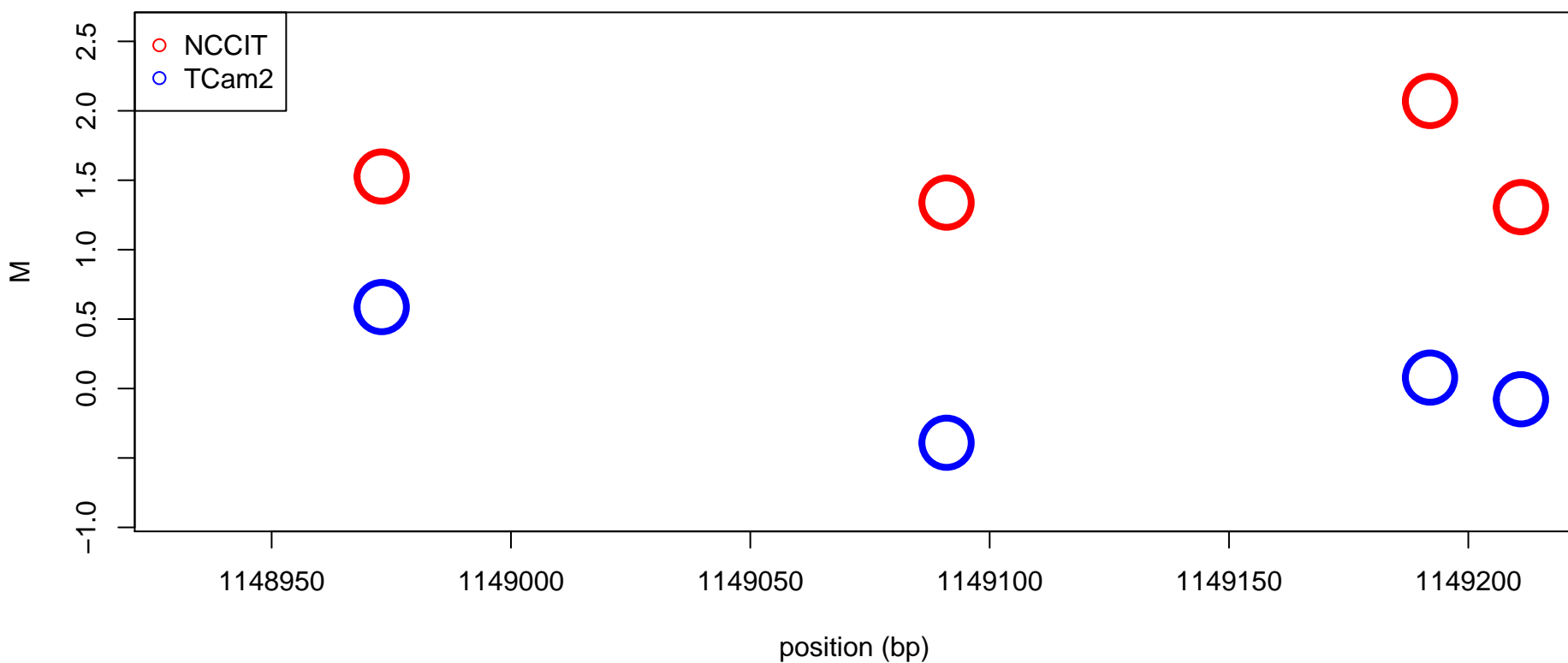

RegionID: 21, chr1:1148973–1149211–Beta\_values

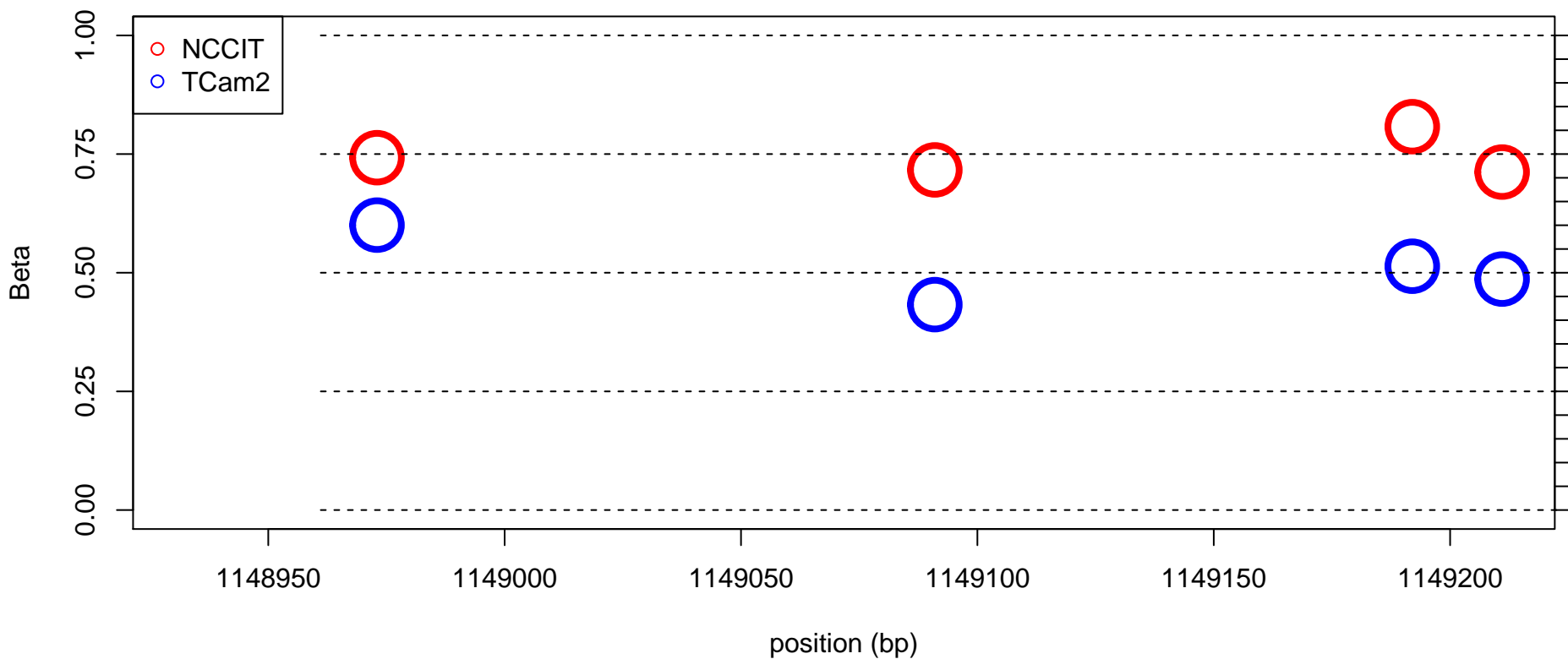

Supplement: File S1 — ZIP file containing DMRforPairs output for significant regions. Please start from the html files. (ZIP) [file pone.0098330.s008.zip › figures/21.pdf]

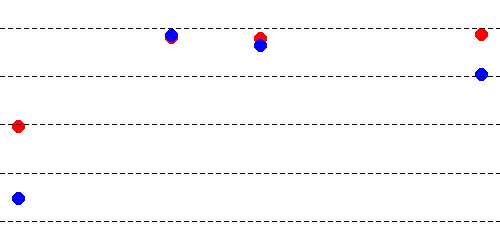

Supplement: File S1 — ZIP file containing DMRforPairs output for significant regions. Please start from the html files. (ZIP) [file pone.0098330.s008.zip › figures/24.png]

RegionID: 24, chr1:1190026–1190238–M\_values

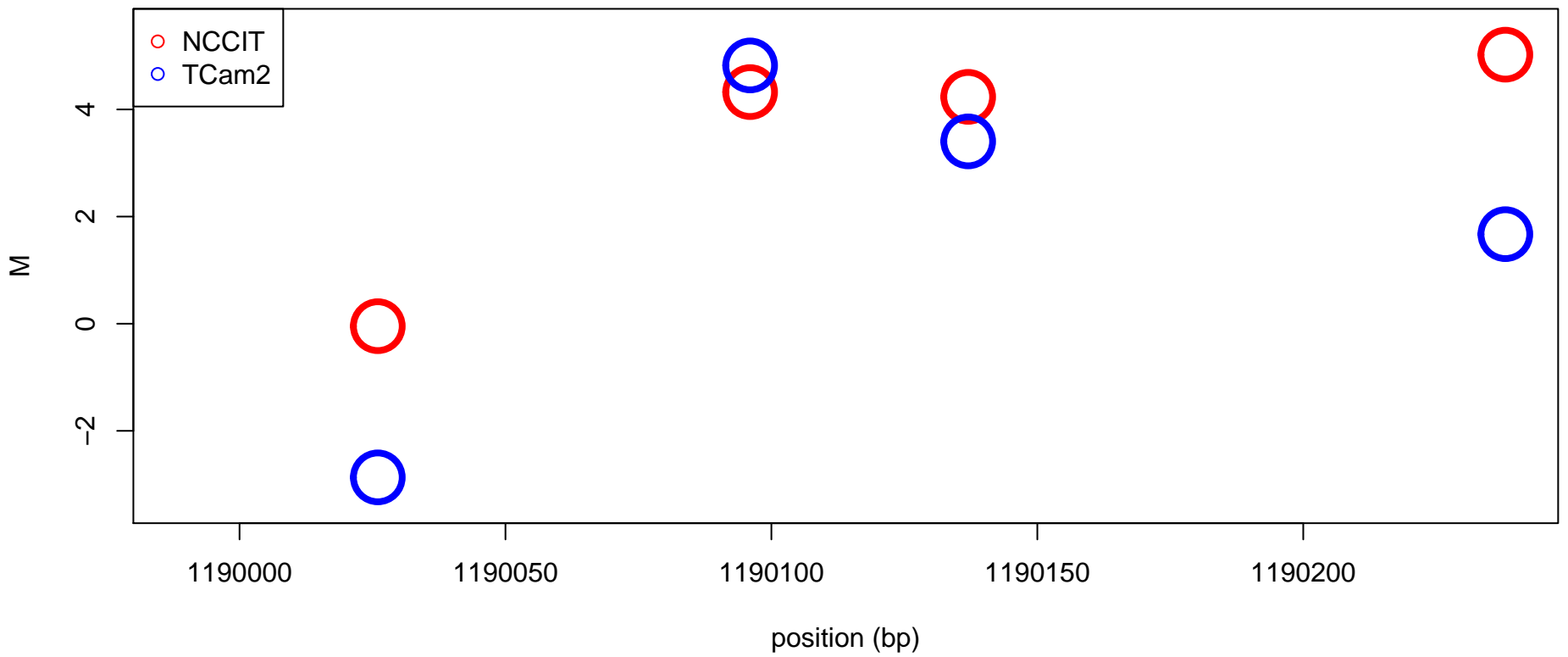

RegionID: 24, chr1:1190026–1190238–Beta\_values

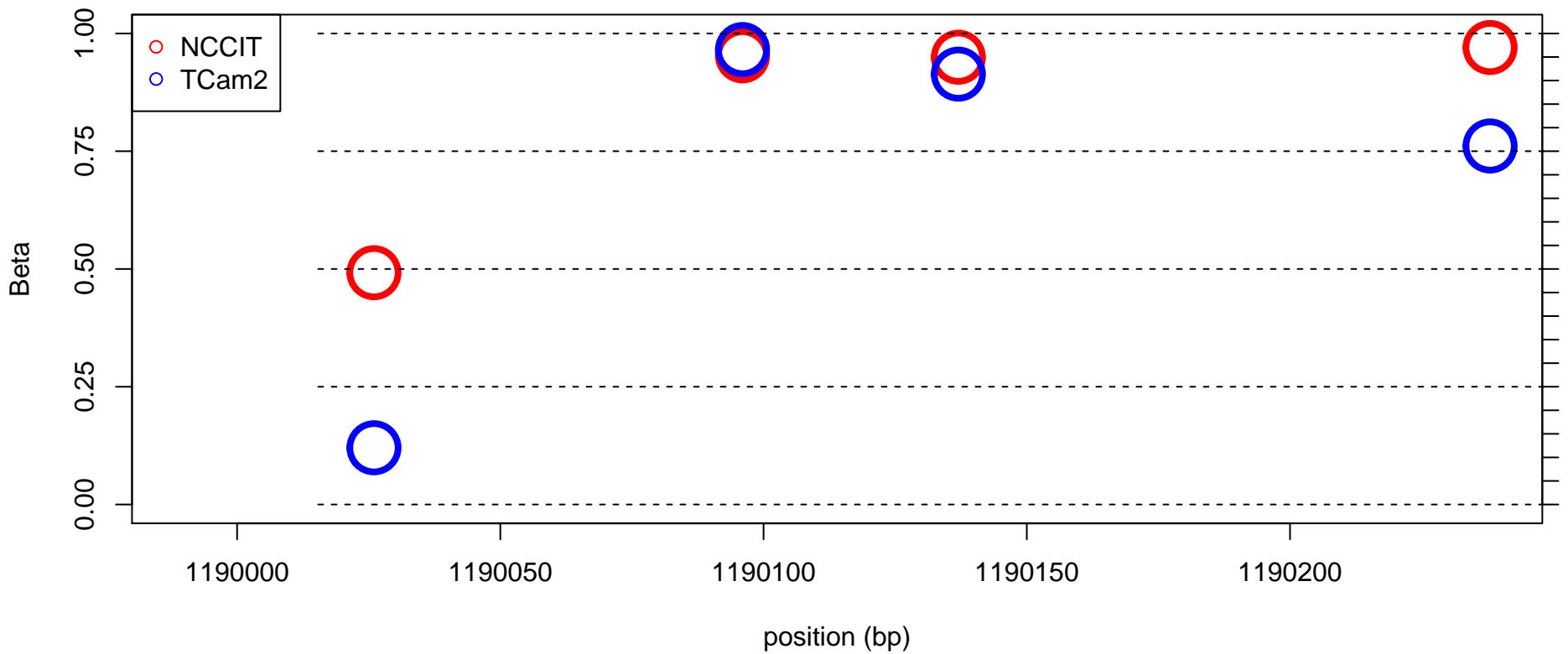

Supplement: File S1 — ZIP file containing DMRforPairs output for significant regions. Please start from the html files. (ZIP) [file pone.0098330.s008.zip › figures/24.pdf]

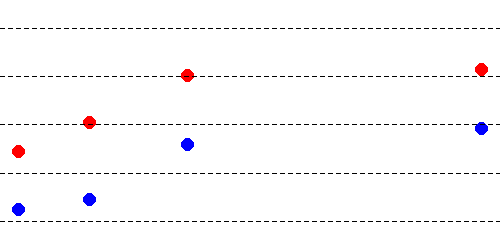

Supplement: File S1 — ZIP file containing DMRforPairs output for significant regions. Please start from the html files. (ZIP) [file pone.0098330.s008.zip › figures/29.png]

RegionID: 29, chr1:1245014–1245184–M\_values

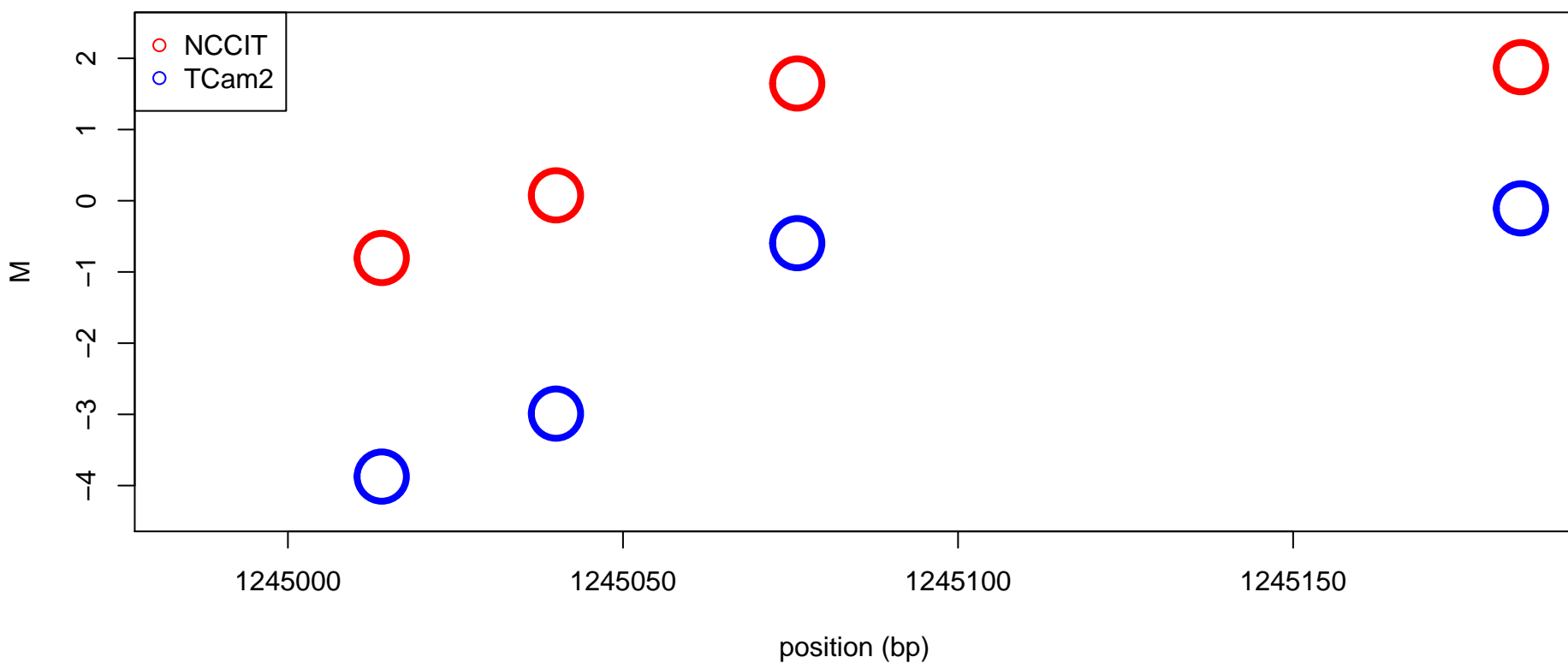

RegionID: 29, chr1:1245014–1245184–Beta\_values

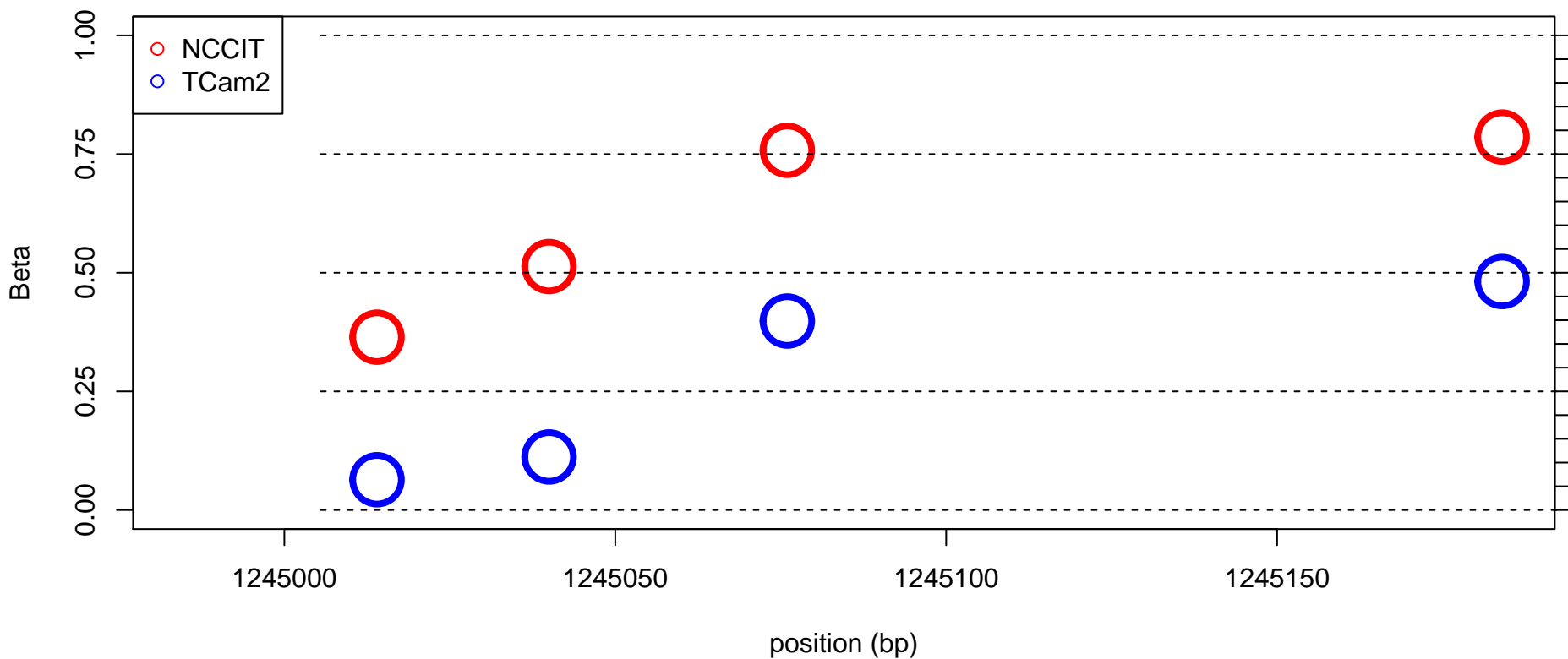

Supplement: File S1 — ZIP file containing DMRforPairs output for significant regions. Please start from the html files. (ZIP) [file pone.0098330.s008.zip › figures/29.pdf]

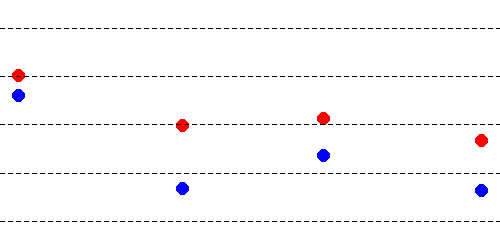

Supplement: File S1 — ZIP file containing DMRforPairs output for significant regions. Please start from the html files. (ZIP) [file pone.0098330.s008.zip › figures/33.png]

RegionID: 33, chr1:1309161–1309641–M\_values

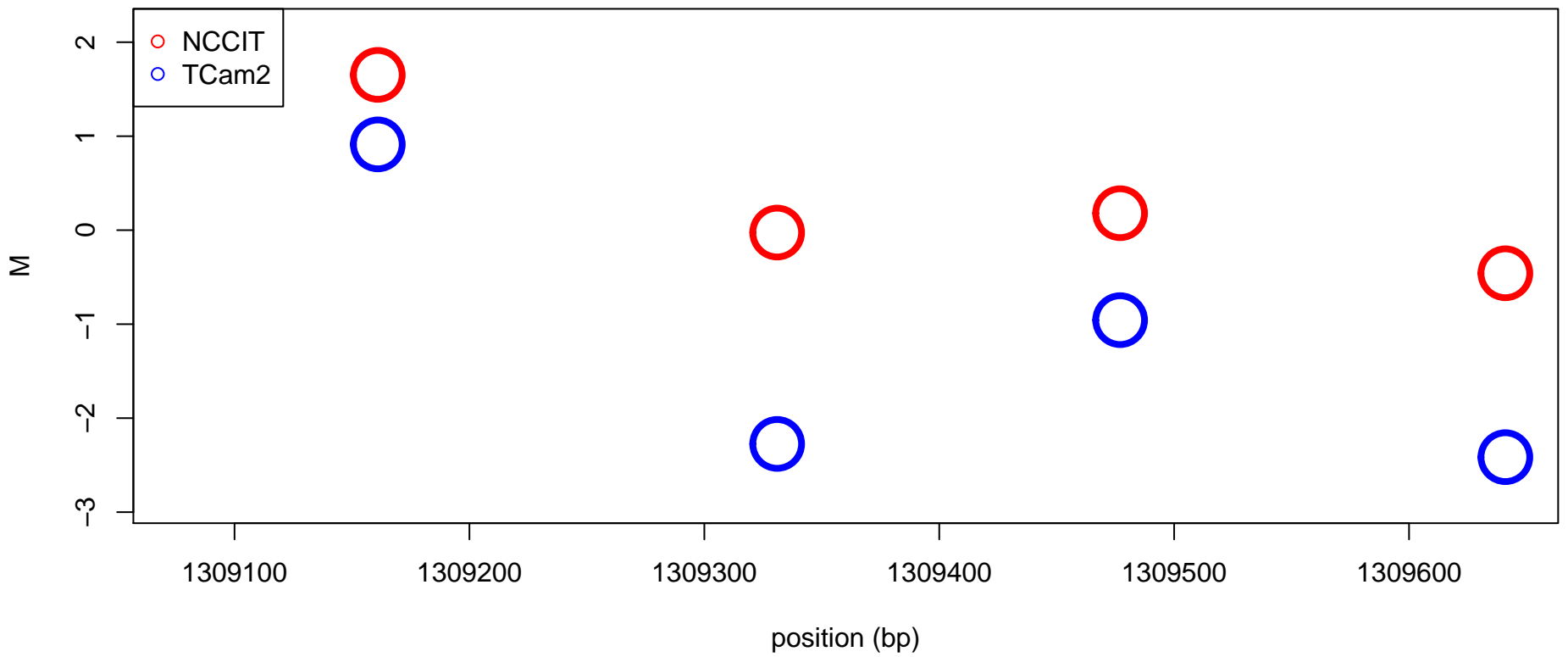

RegionID: 33, chr1:1309161–1309641–Beta\_values

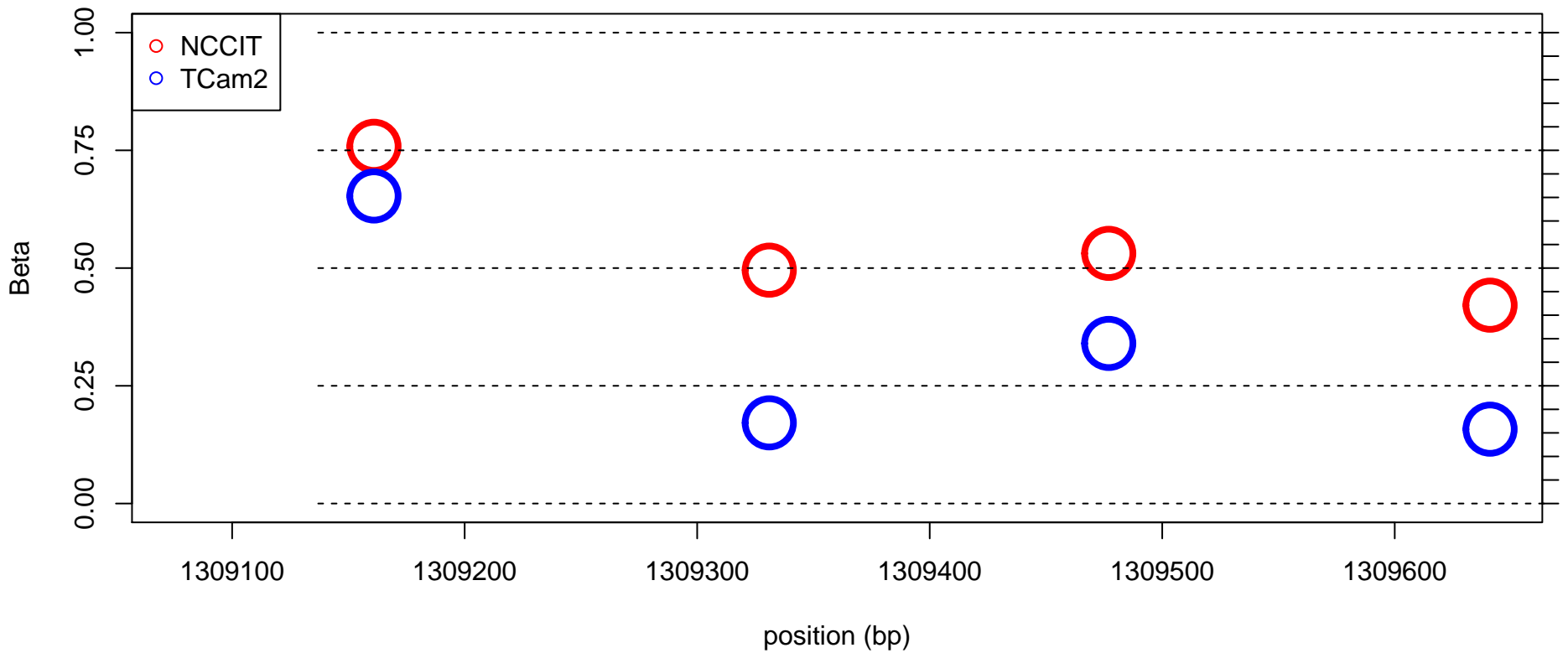

Supplement: File S1 — ZIP file containing DMRforPairs output for significant regions. Please start from the html files. (ZIP) [file pone.0098330.s008.zip › figures/33.pdf]

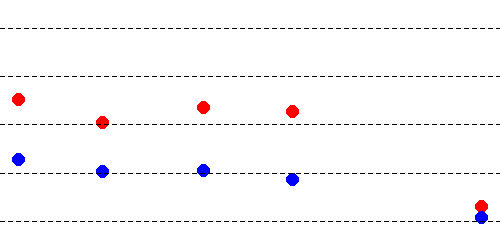

Supplement: File S1 — ZIP file containing DMRforPairs output for significant regions. Please start from the html files. (ZIP) [file pone.0098330.s008.zip › figures/63.png]

RegionID: 63, chr1:2063799–2064216–M\_values

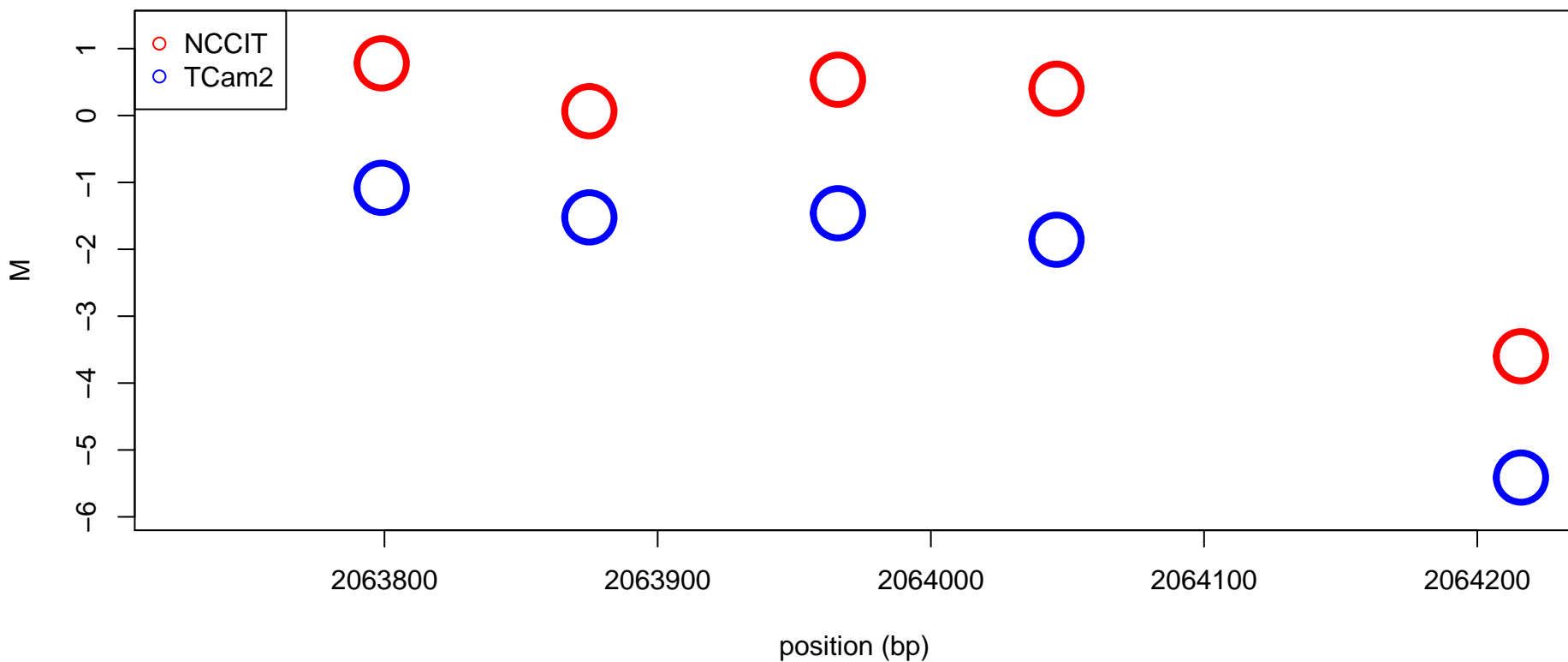

RegionID: 63, chr1:2063799–2064216–Beta\_values

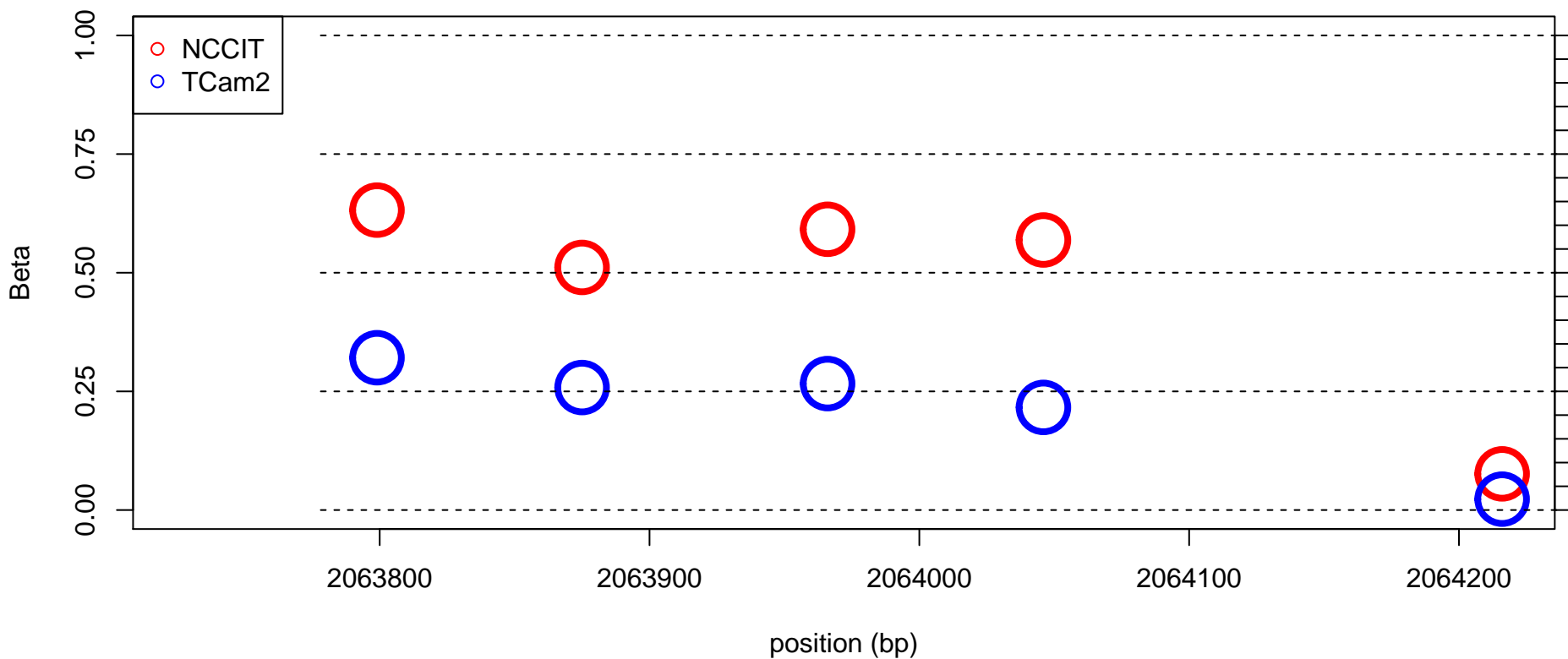

Supplement: File S1 — ZIP file containing DMRforPairs output for significant regions. Please start from the html files. (ZIP) [file pone.0098330.s008.zip › figures/63.pdf]

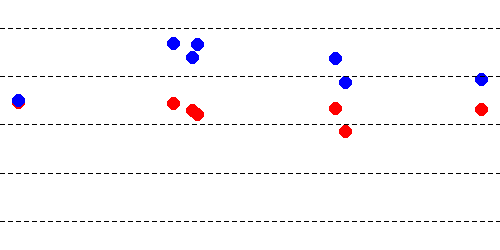

Supplement: File S1 — ZIP file containing DMRforPairs output for significant regions. Please start from the html files. (ZIP) [file pone.0098330.s008.zip › figures/69.png]

RegionID: 69, chr1:2125049–2125243–M\_values

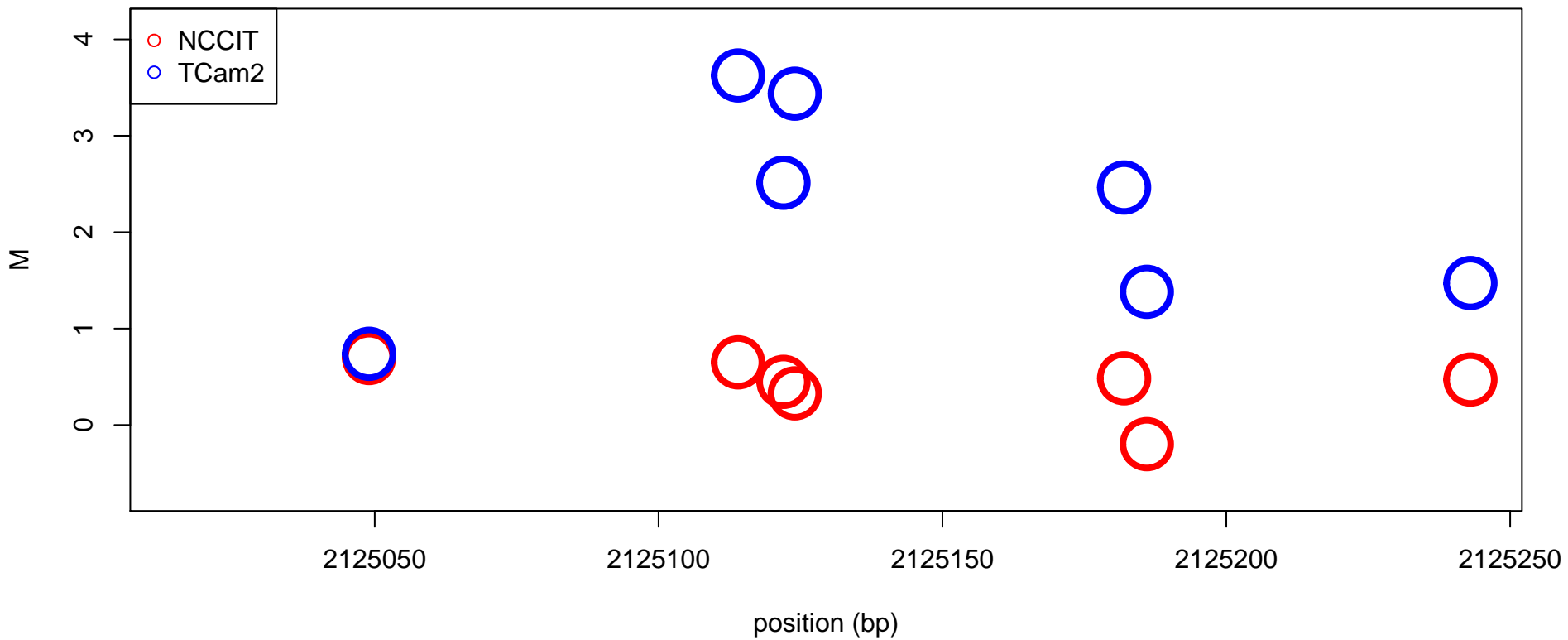

RegionID: 69, chr1:2125049–2125243–Beta\_values

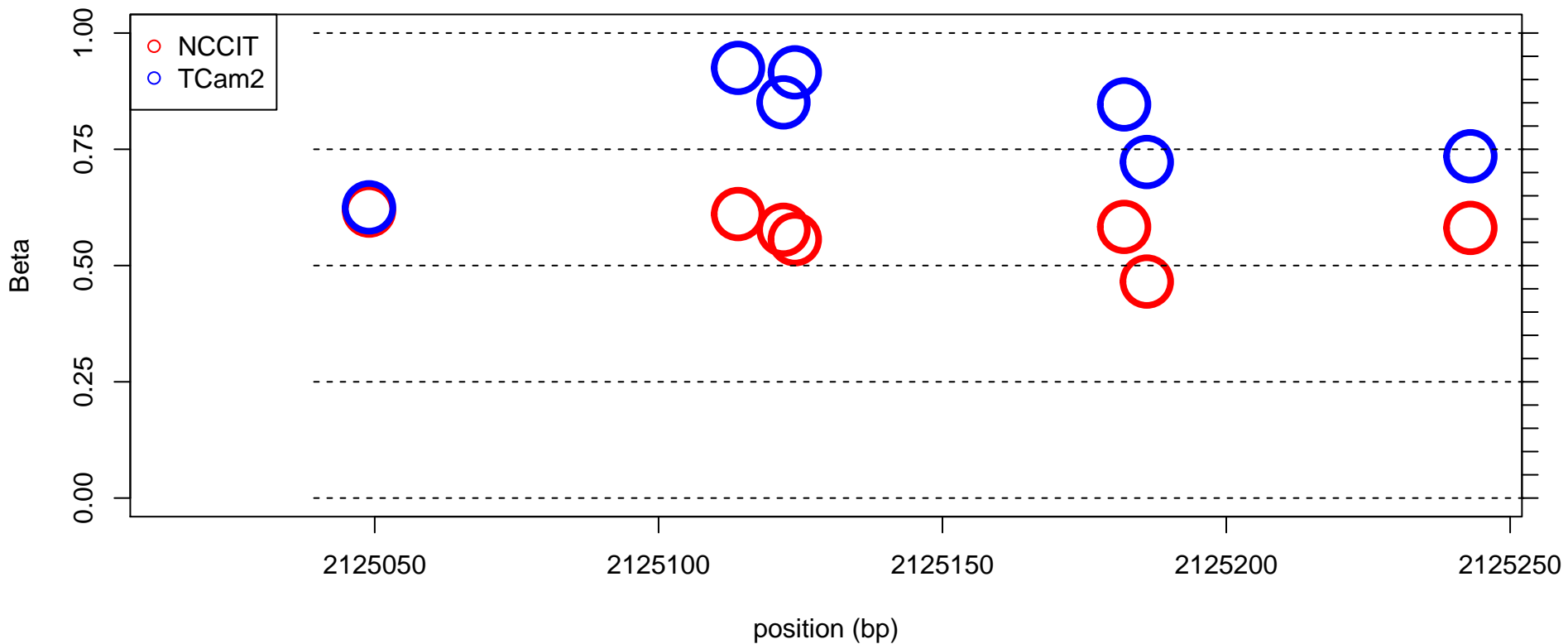

Supplement: File S1 — ZIP file containing DMRforPairs output for significant regions. Please start from the html files. (ZIP) [file pone.0098330.s008.zip › figures/69.pdf]

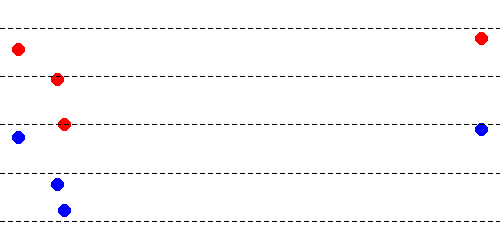

Supplement: File S1 — ZIP file containing DMRforPairs output for significant regions. Please start from the html files. (ZIP) [file pone.0098330.s008.zip › figures/81.png]

RegionID: 81, chr1:2324205–2324396–M\_values

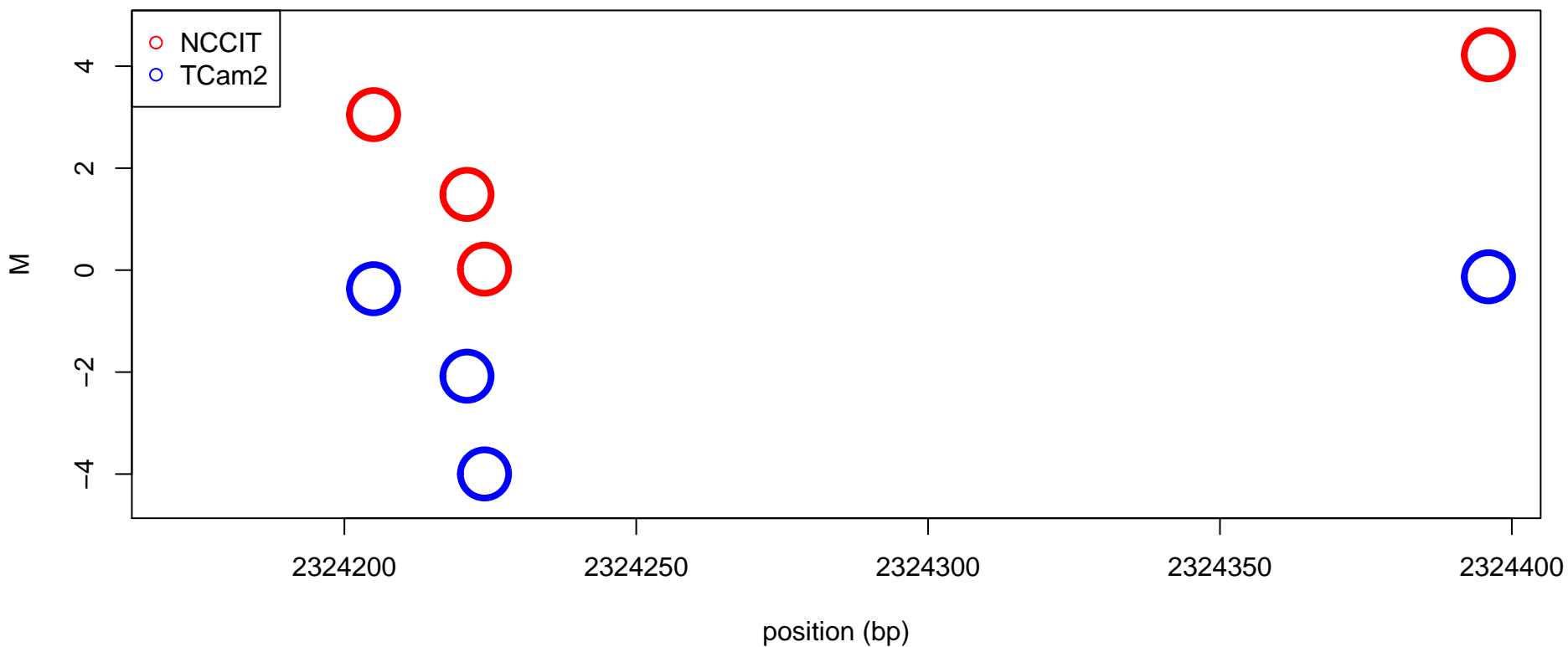

RegionID: 81, chr1:2324205–2324396–Beta\_values

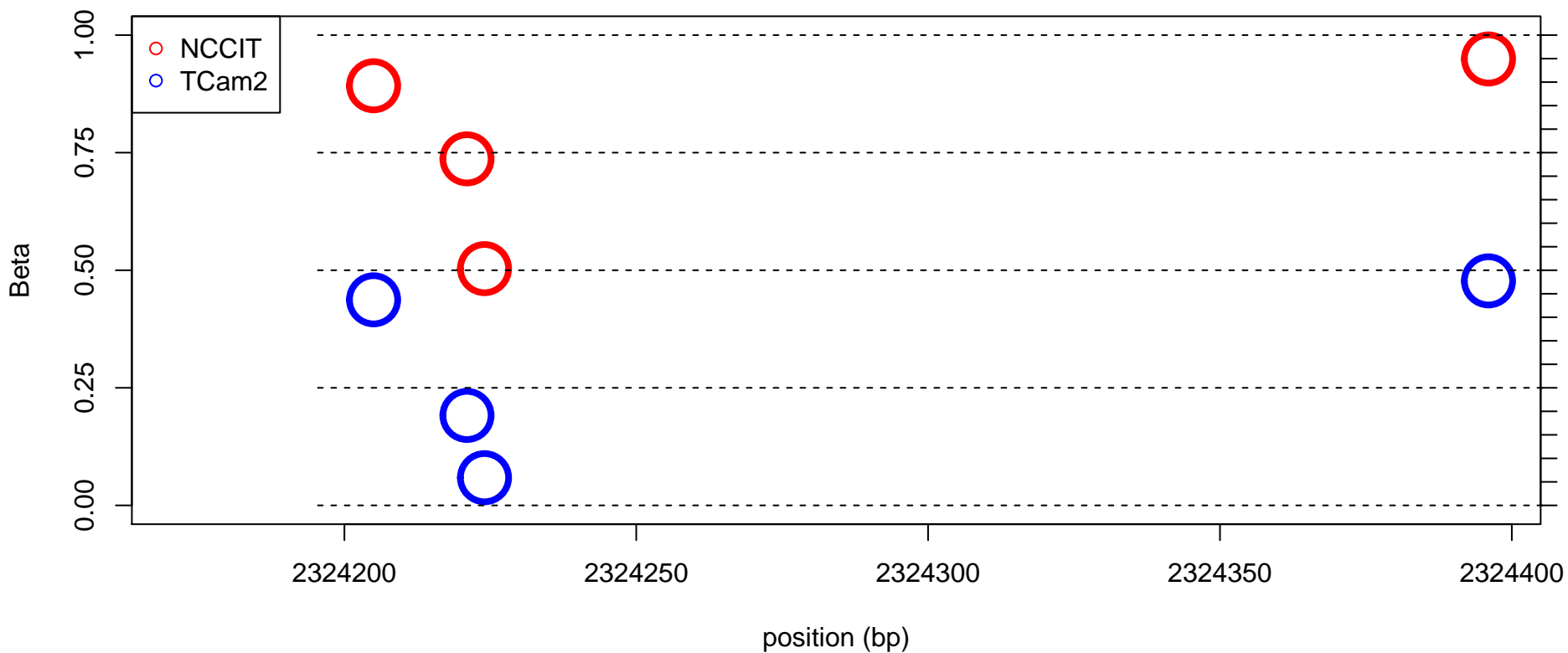

Supplement: File S1 — ZIP file containing DMRforPairs output for significant regions. Please start from the html files. (ZIP) [file pone.0098330.s008.zip › figures/81.pdf]

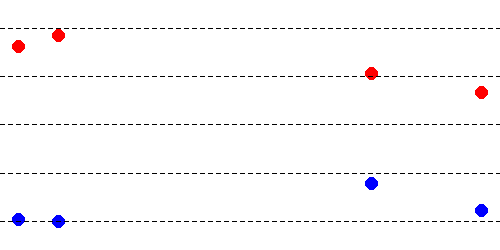

Supplement: File S1 — ZIP file containing DMRforPairs output for significant regions. Please start from the html files. (ZIP) [file pone.0098330.s008.zip › figures/94.png]

RegionID: 94, chr1:2980380-2980598-M\_values

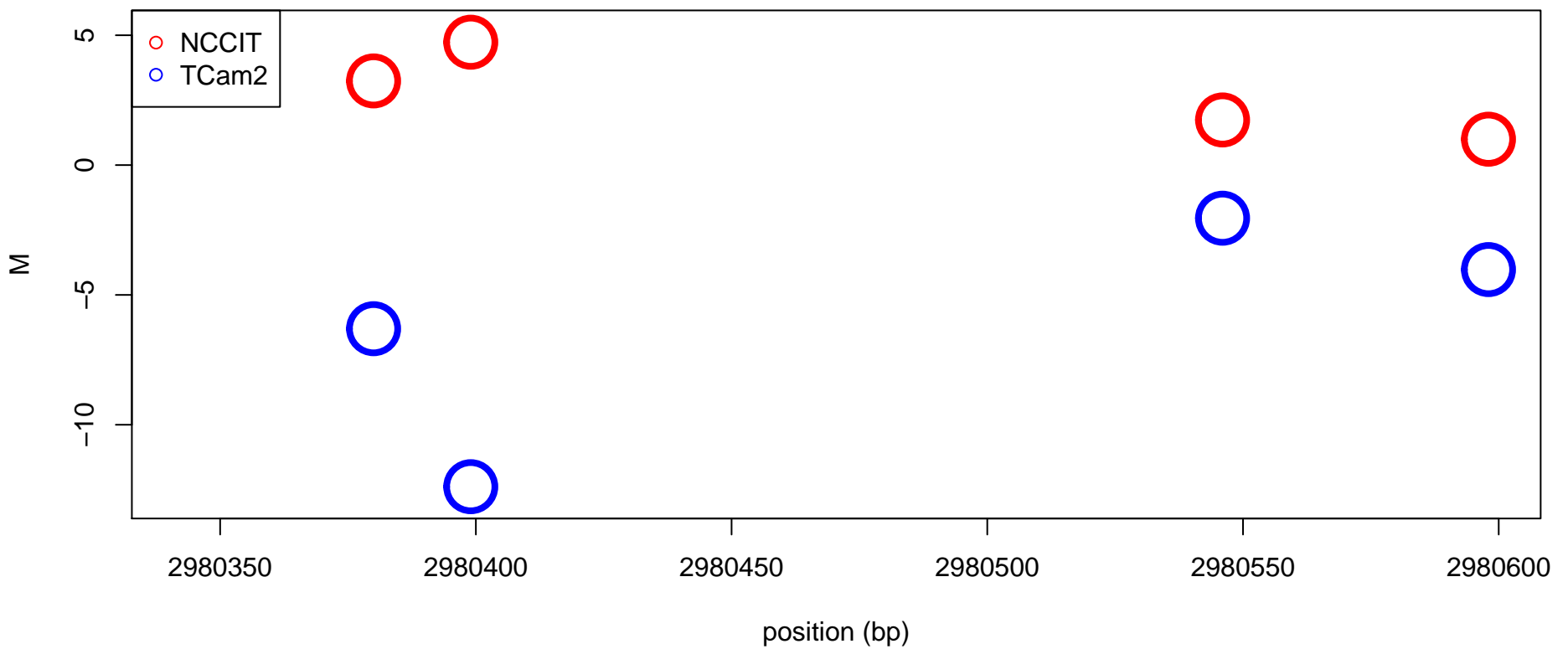

RegionID: 94, chr1:2980380-2980598-Beta\_values

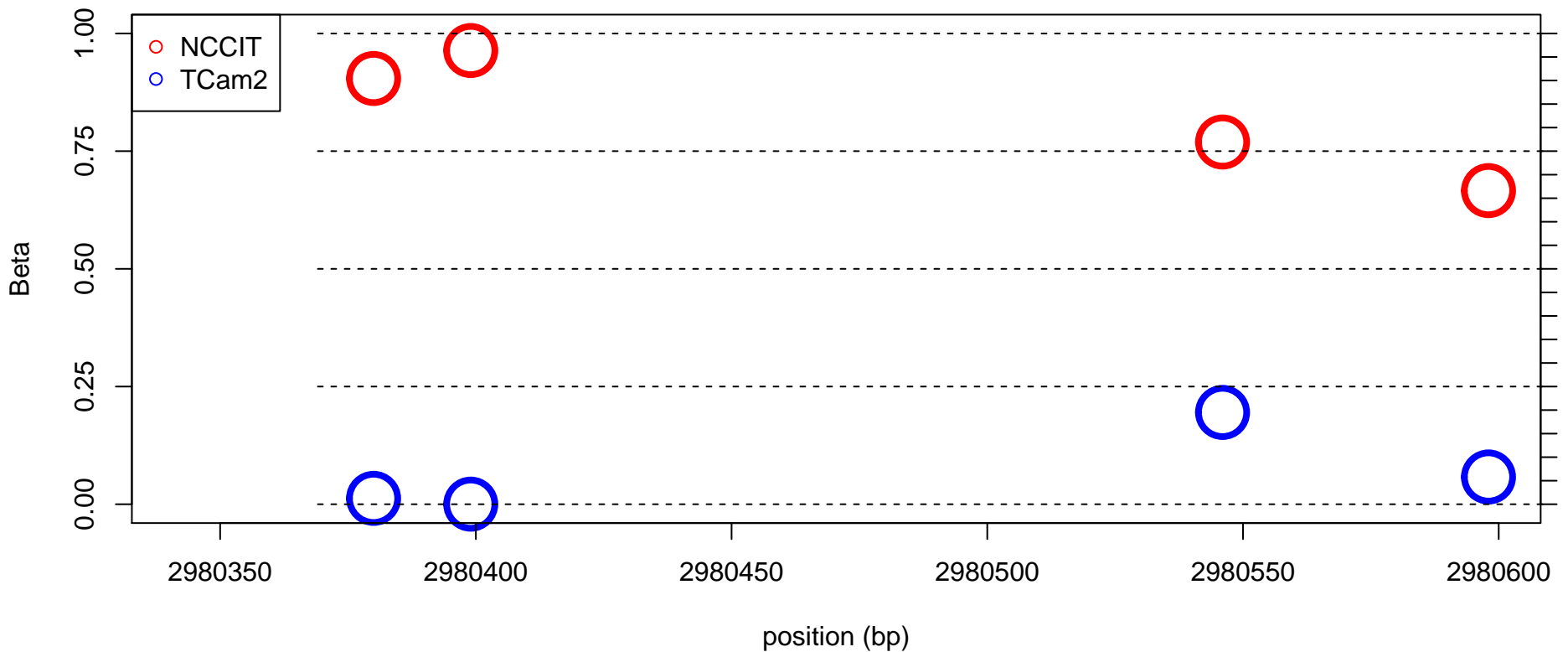

Supplement: File S1 — ZIP file containing DMRforPairs output for significant regions. Please start from the html files. (ZIP) [file pone.0098330.s008.zip › figures/94.pdf]

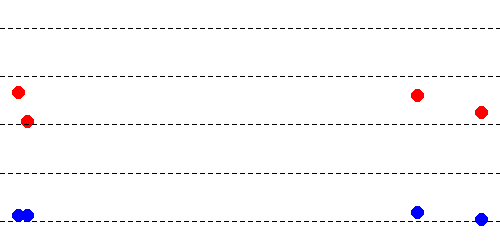

Supplement: File S1 — ZIP file containing DMRforPairs output for significant regions. Please start from the html files. (ZIP) [file pone.0098330.s008.zip › figures/96.png]

RegionID: 96, chr1:2986362–2986566–M\_values

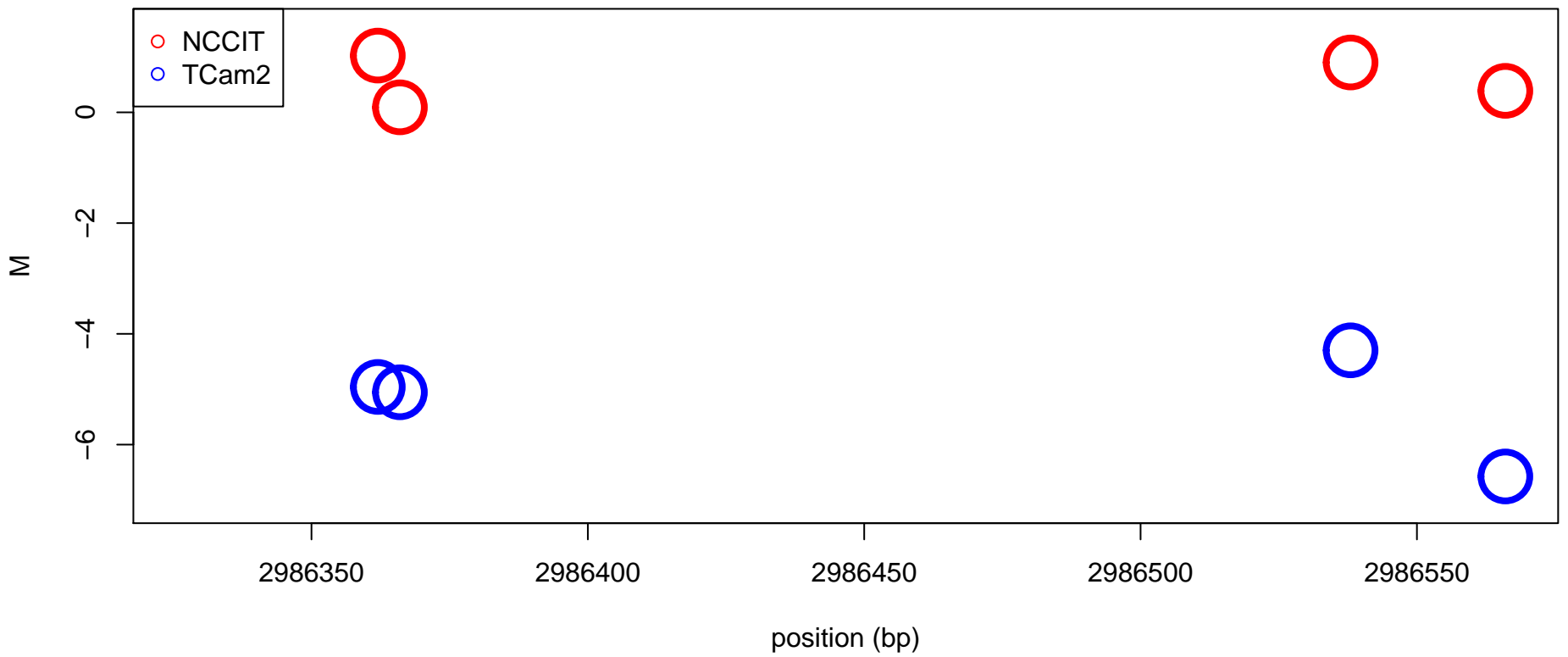

RegionID: 96, chr1:2986362–2986566–Beta\_values

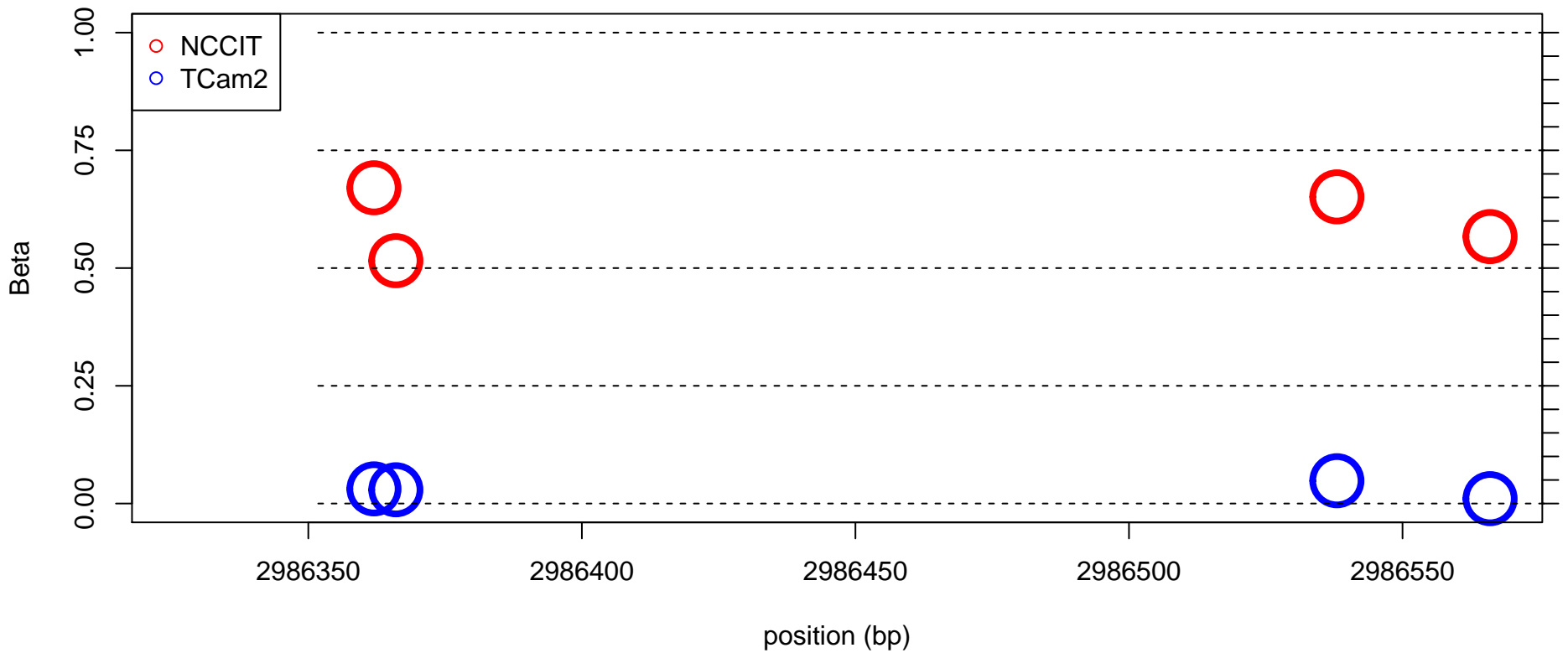

Supplement: File S1 — ZIP file containing DMRforPairs output for significant regions. Please start from the html files. (ZIP) [file pone.0098330.s008.zip › figures/96.pdf]

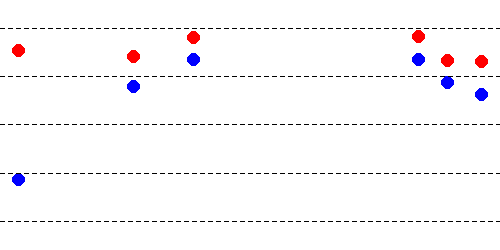

Supplement: File S1 — ZIP file containing DMRforPairs output for significant regions. Please start from the html files. (ZIP) [file pone.0098330.s008.zip › figures/99.png]

RegionID: 99, chr1:3028813–3029070–M\_values

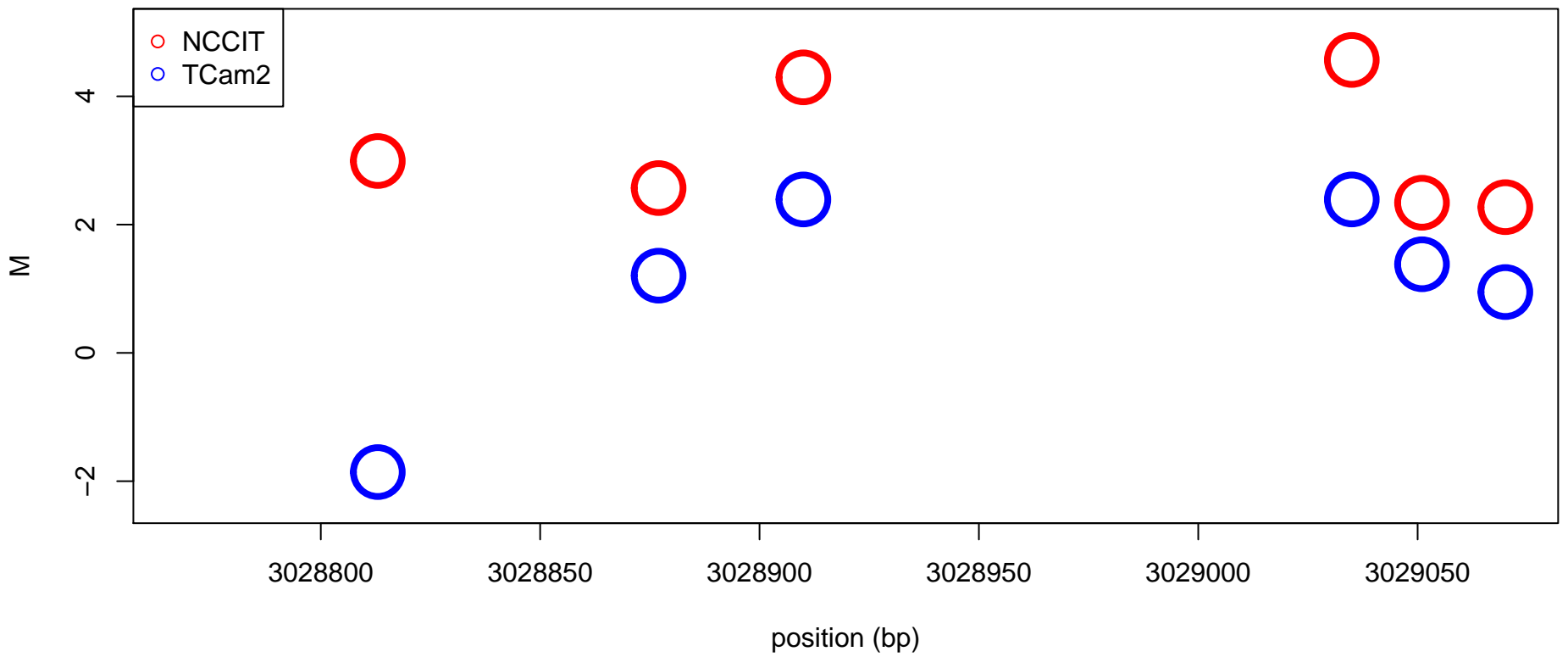

RegionID: 99, chr1:3028813–3029070–Beta\_values

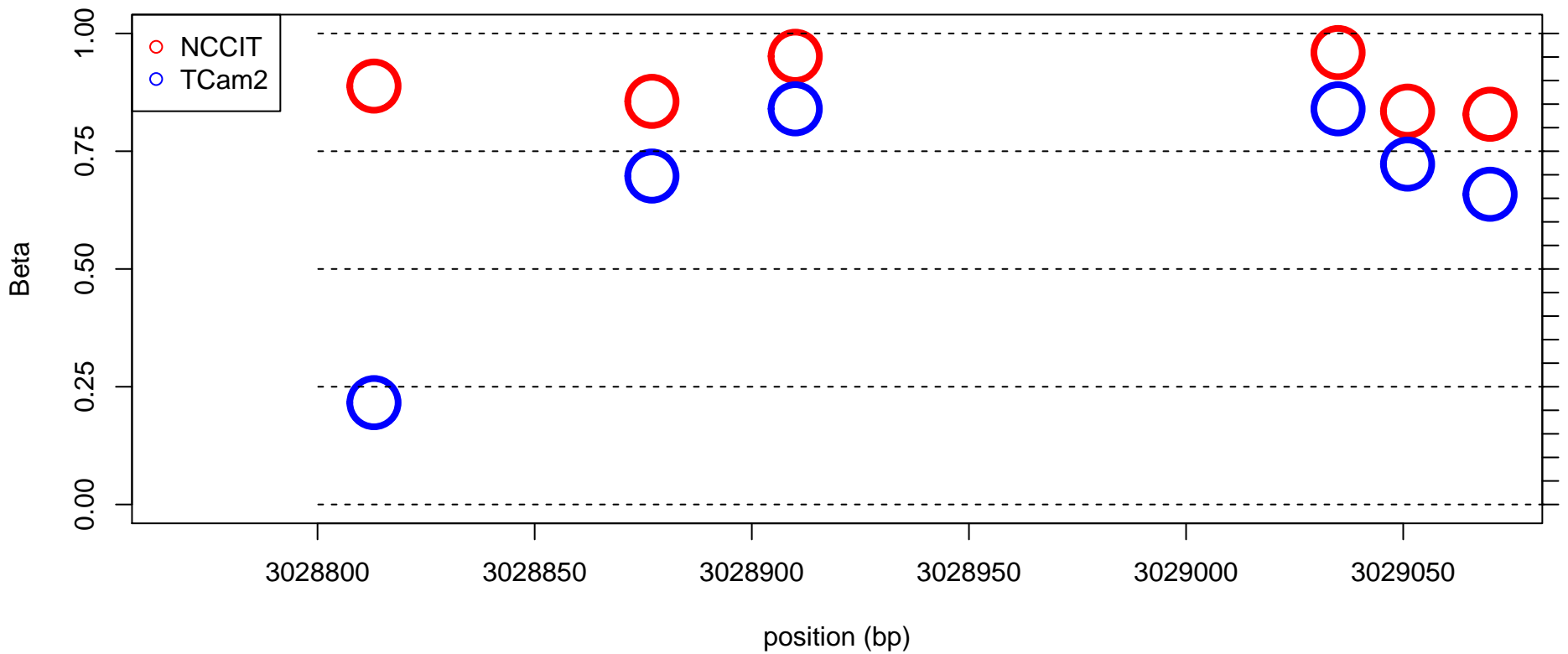

Supplement: File S1 — ZIP file containing DMRforPairs output for significant regions. Please start from the html files. (ZIP) [file pone.0098330.s008.zip › figures/99.pdf]

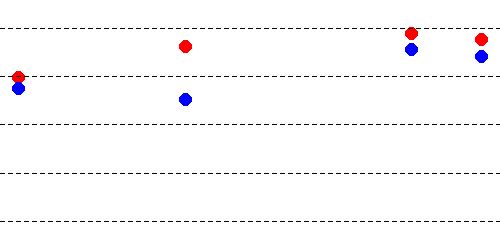

Supplement: File S1 — ZIP file containing DMRforPairs output for significant regions. Please start from the html files. (ZIP) [file pone.0098330.s008.zip › figures/103.png]

RegionID: 103, chr1:3059517–3059820–M\_values

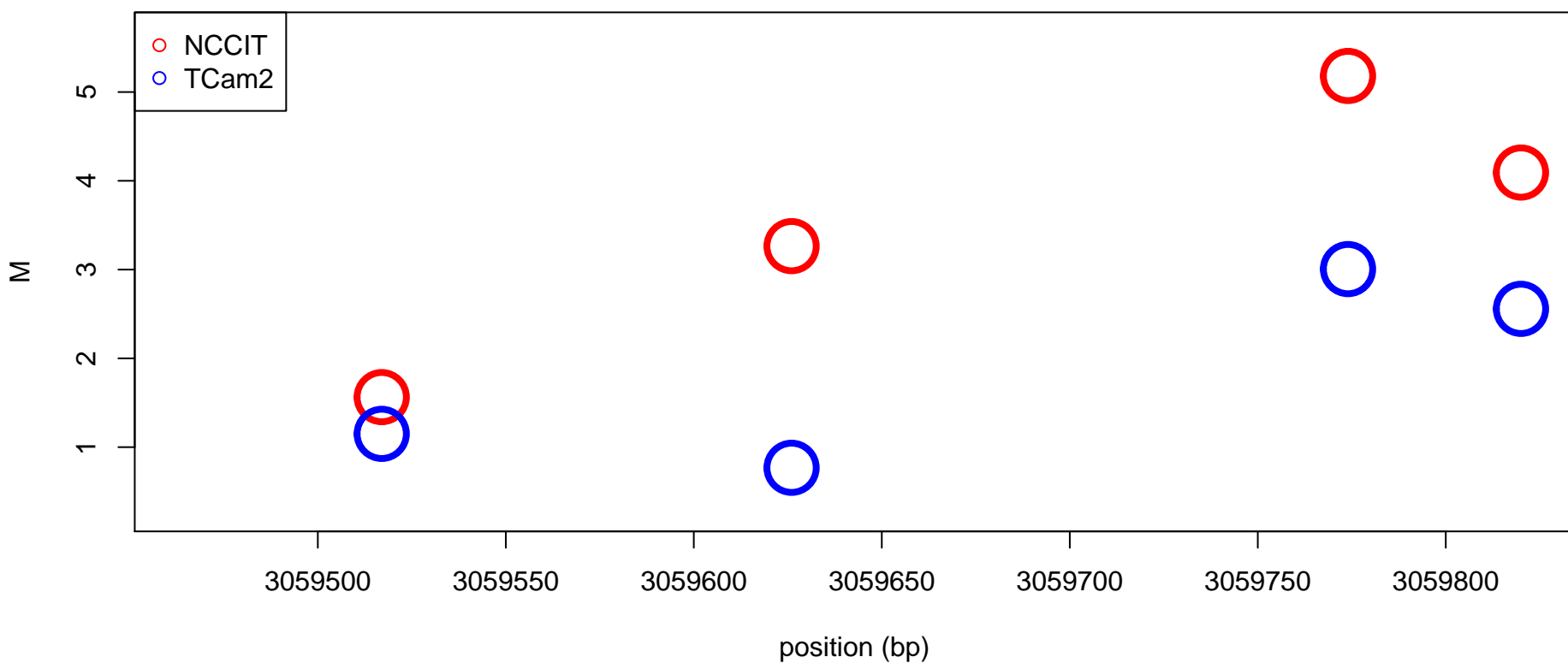

RegionID: 103, chr1:3059517–3059820–Beta\_values

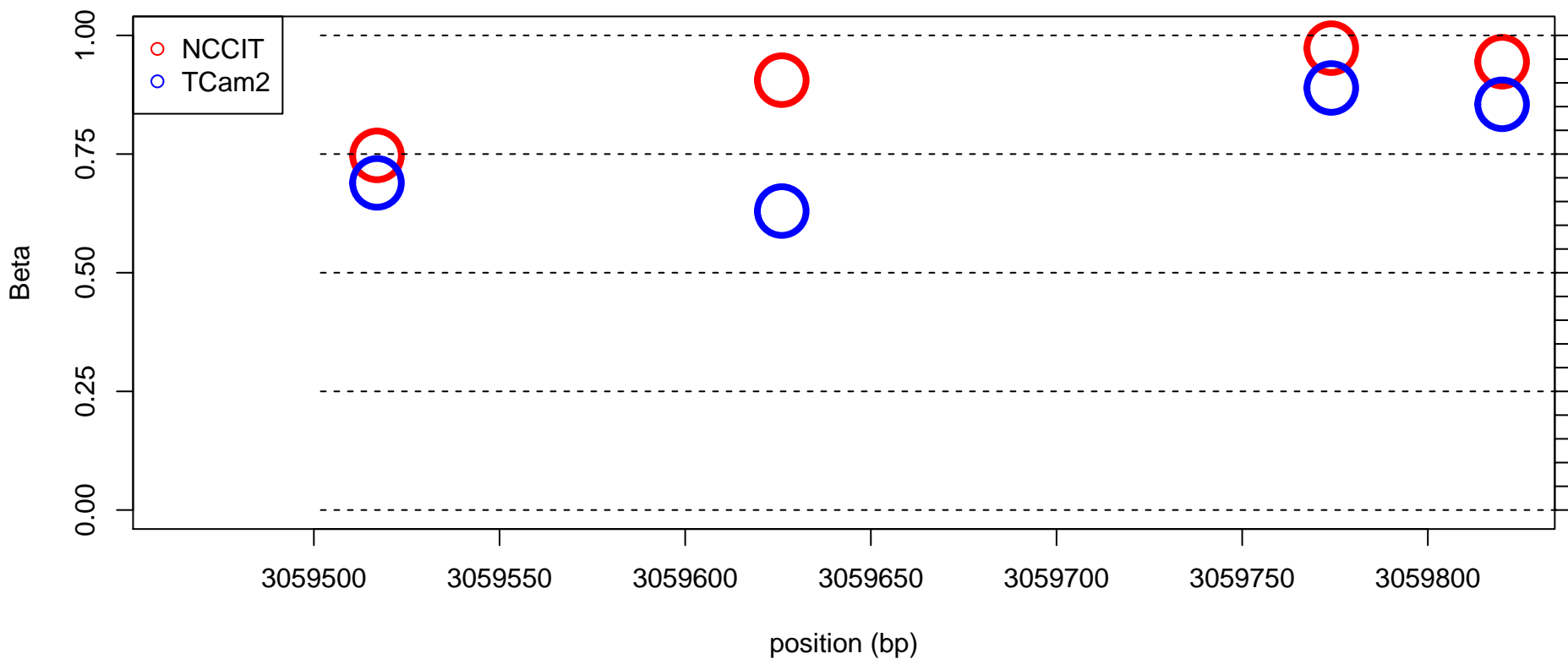

Supplement: File S1 — ZIP file containing DMRforPairs output for significant regions. Please start from the html files. (ZIP) [file pone.0098330.s008.zip › figures/103.pdf]

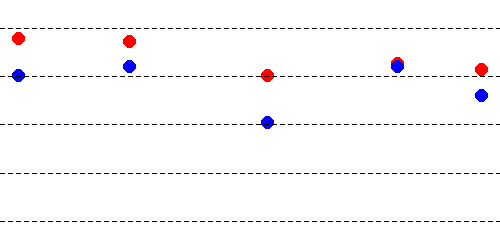

Supplement: File S1 — ZIP file containing DMRforPairs output for significant regions. Please start from the html files. (ZIP) [file pone.0098330.s008.zip › figures/108.png]

RegionID: 108, chr1:3143682–3144209–M\_values

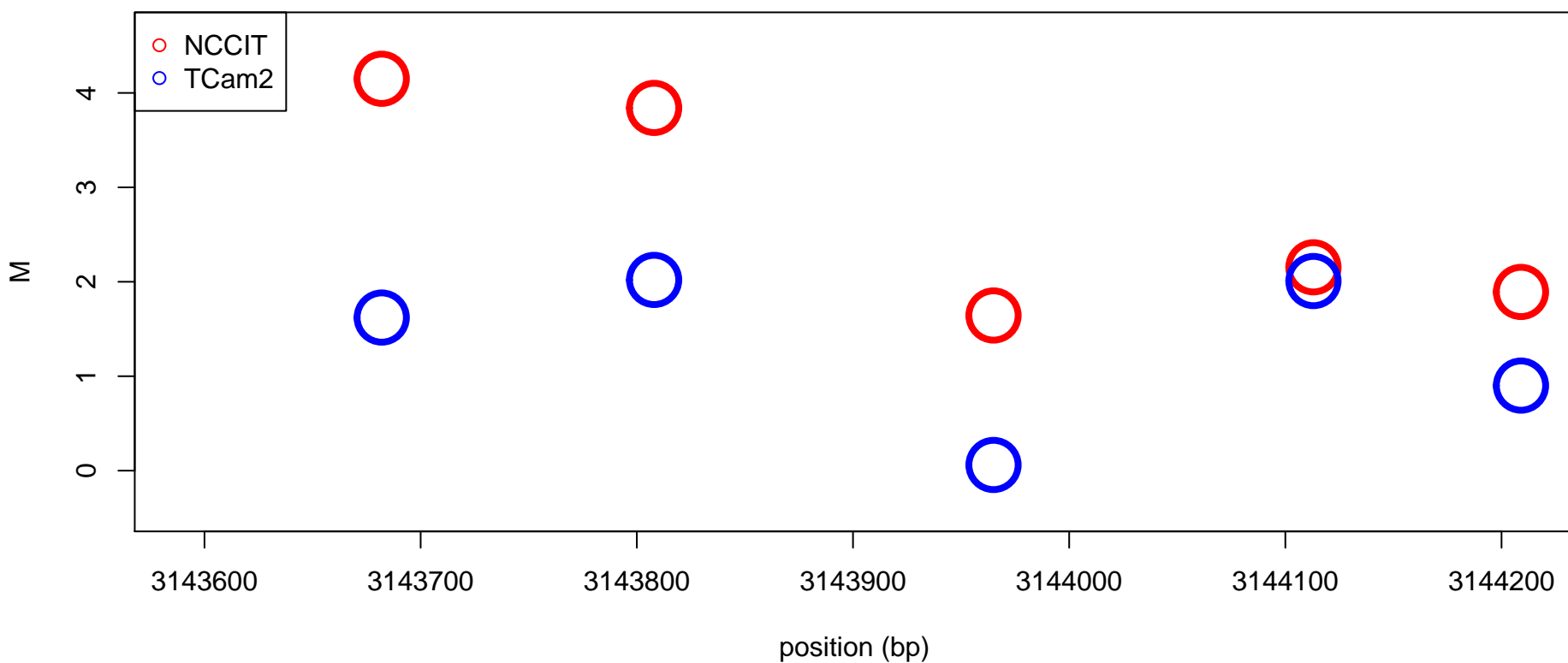

RegionID: 108, chr1:3143682–3144209–Beta\_values

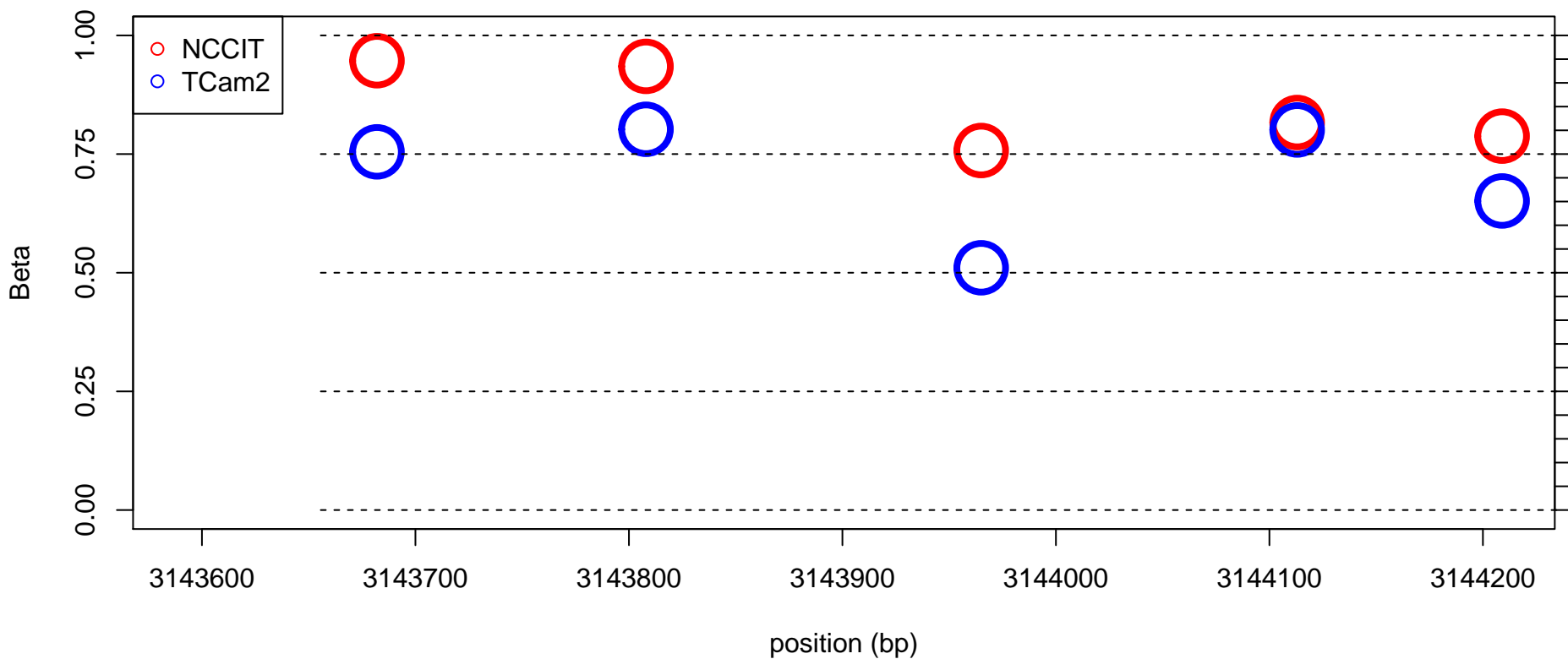

Supplement: File S1 — ZIP file containing DMRforPairs output for significant regions. Please start from the html files. (ZIP) [file pone.0098330.s008.zip › figures/108.pdf]

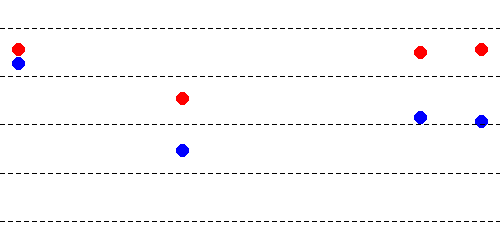

Supplement: File S1 — ZIP file containing DMRforPairs output for significant regions. Please start from the html files. (ZIP) [file pone.0098330.s008.zip › figures/111.png]

RegionID: 111, chr1:3162066–3162173–M\_values

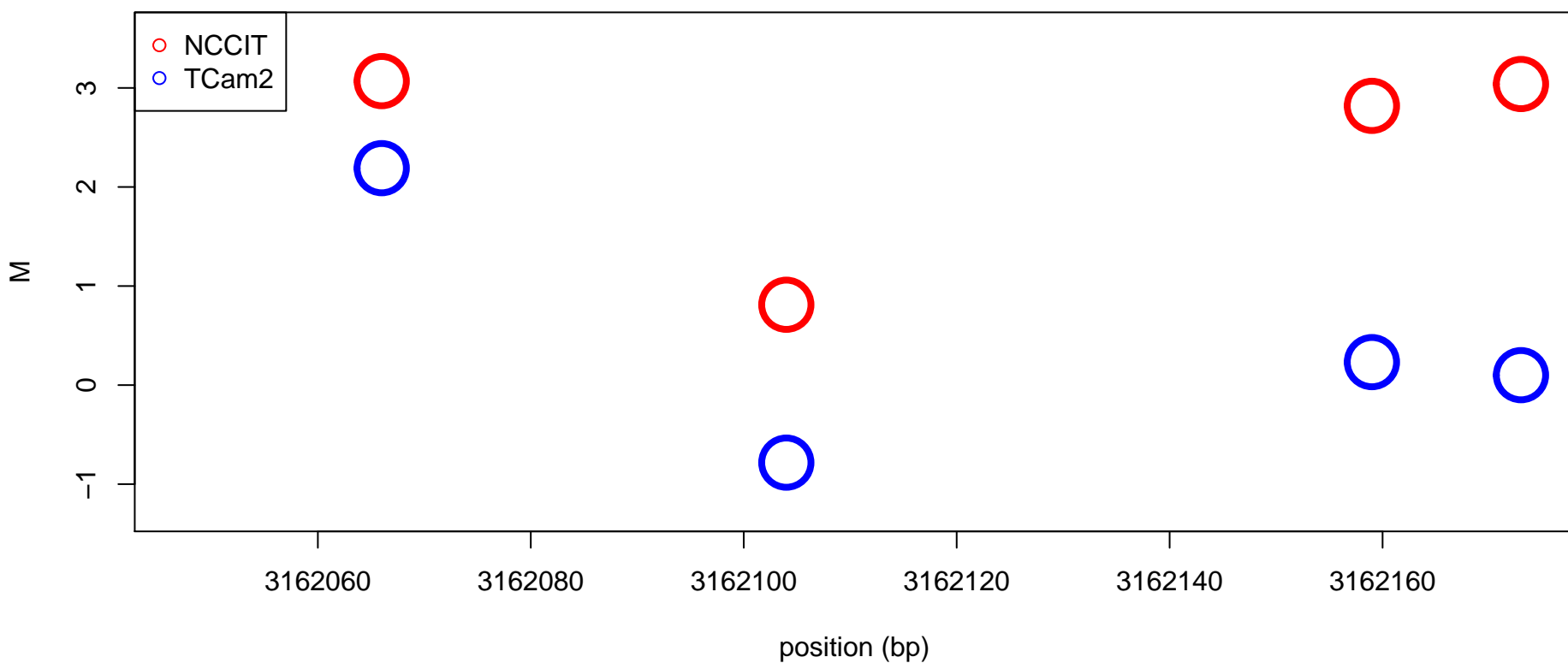

RegionID: 111, chr1:3162066–3162173–Beta\_values

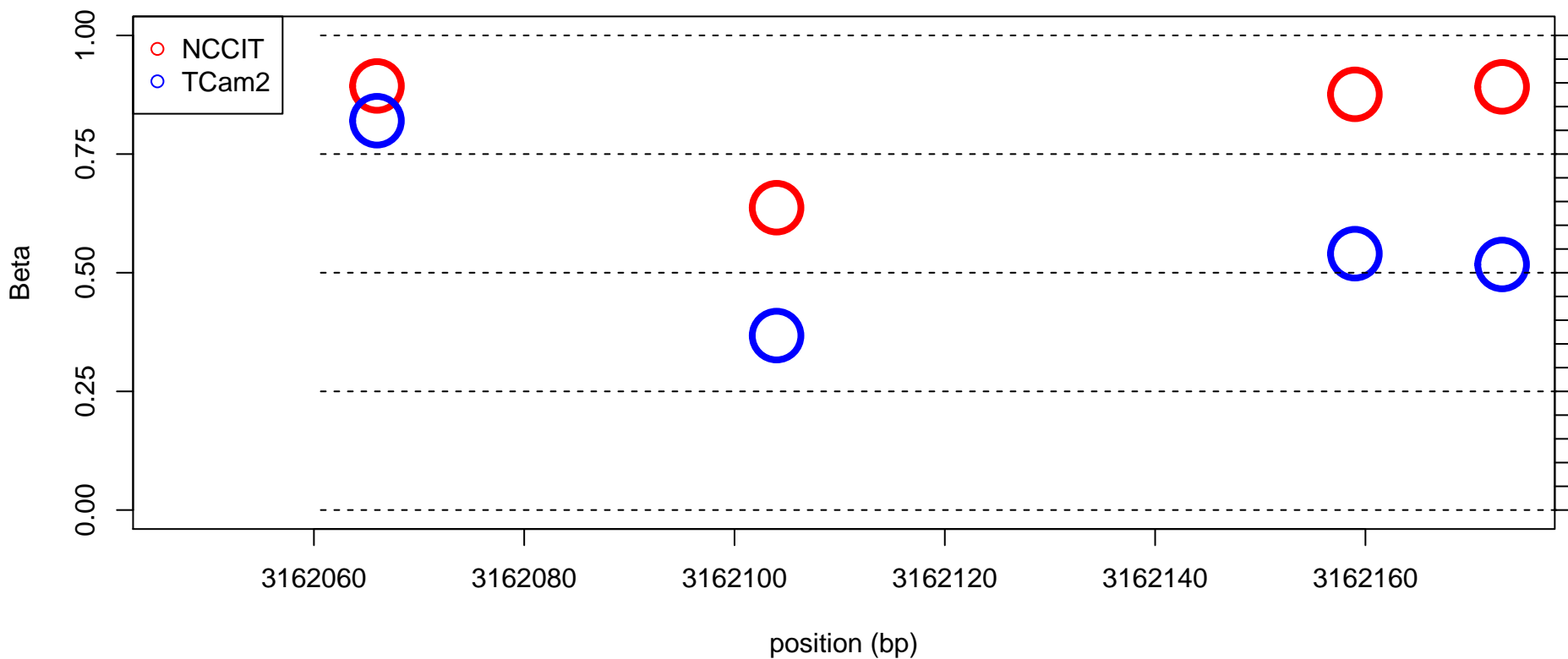

Supplement: File S1 — ZIP file containing DMRforPairs output for significant regions. Please start from the html files. (ZIP) [file pone.0098330.s008.zip › figures/111.pdf]

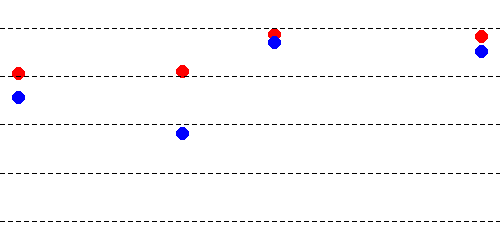

Supplement: File S1 — ZIP file containing DMRforPairs output for significant regions. Please start from the html files. (ZIP) [file pone.0098330.s008.zip › figures/112.png]

RegionID: 112, chr1:3163341-3163710-M\_values

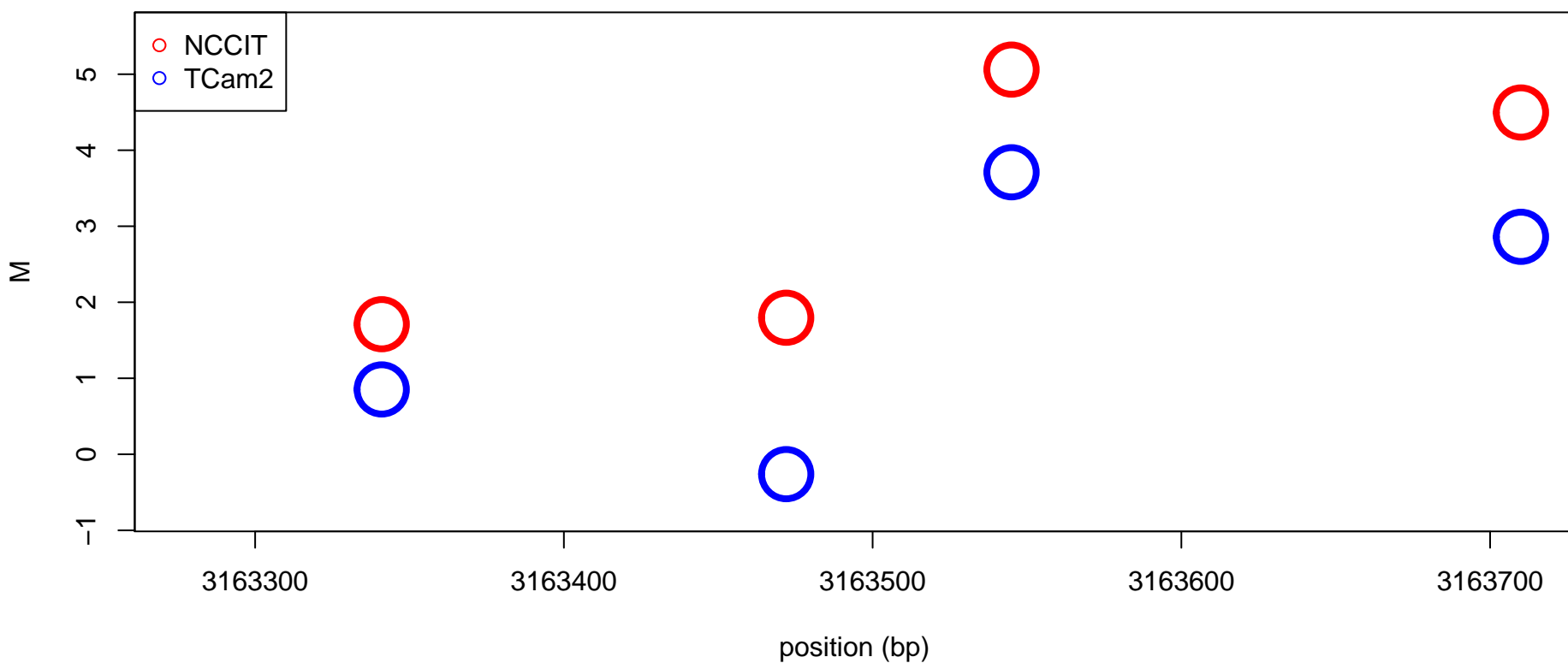

RegionID: 112, chr1:3163341-3163710-Beta\_values

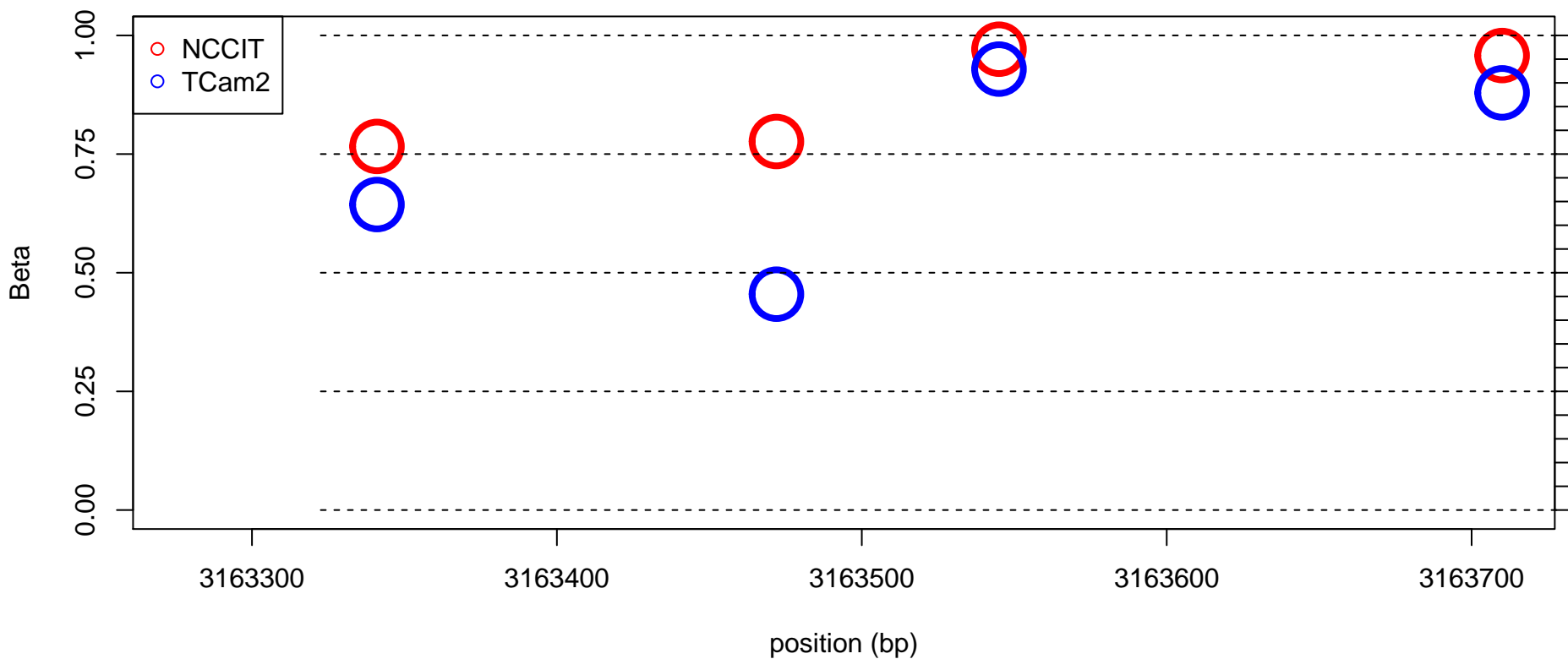

Supplement: File S1 — ZIP file containing DMRforPairs output for significant regions. Please start from the html files. (ZIP) [file pone.0098330.s008.zip › figures/112.pdf]

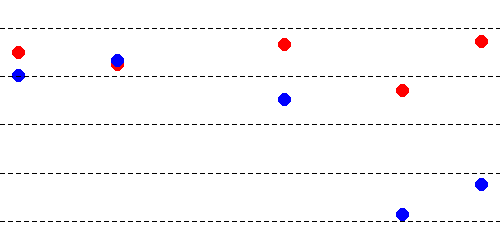

Supplement: File S1 — ZIP file containing DMRforPairs output for significant regions. Please start from the html files. (ZIP) [file pone.0098330.s008.zip › figures/113.png]

RegionID: 113, chr1:3182620–3183165–M\_values

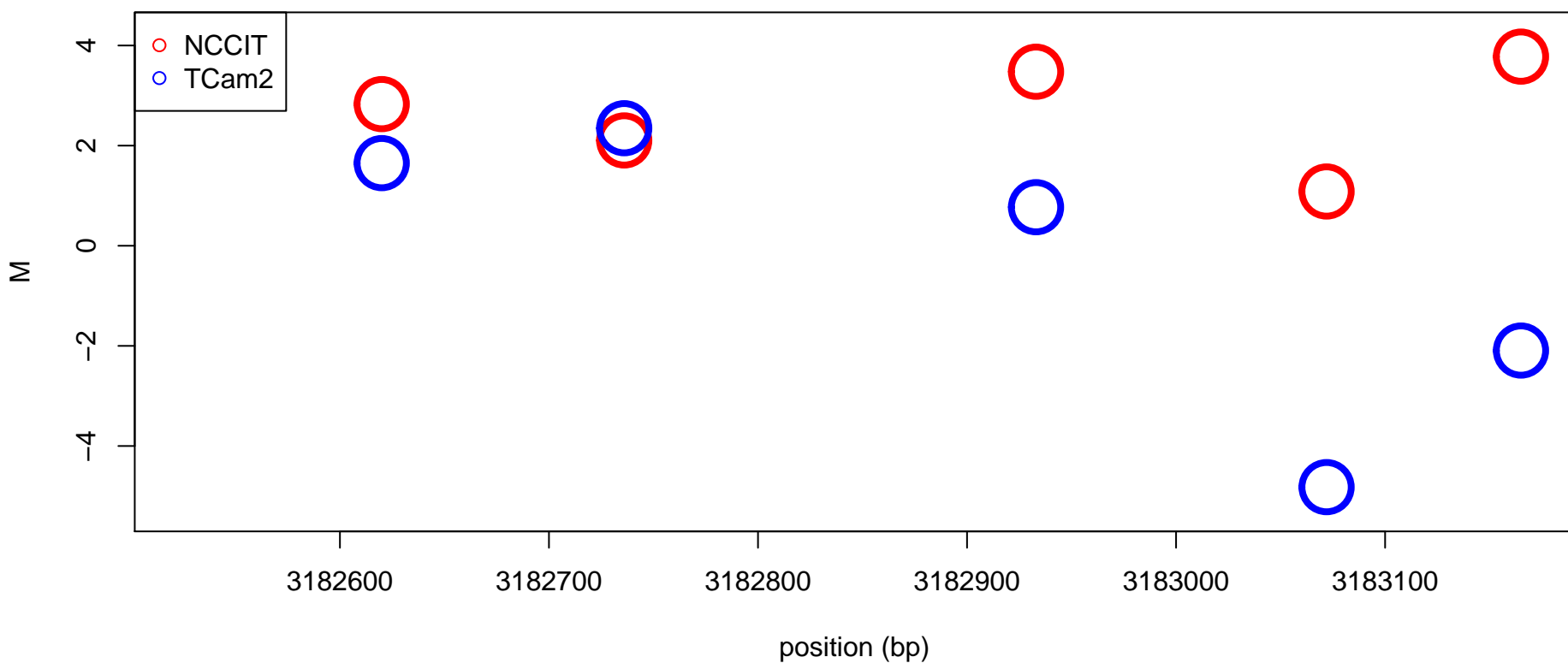

RegionID: 113, chr1:3182620–3183165–Beta\_values

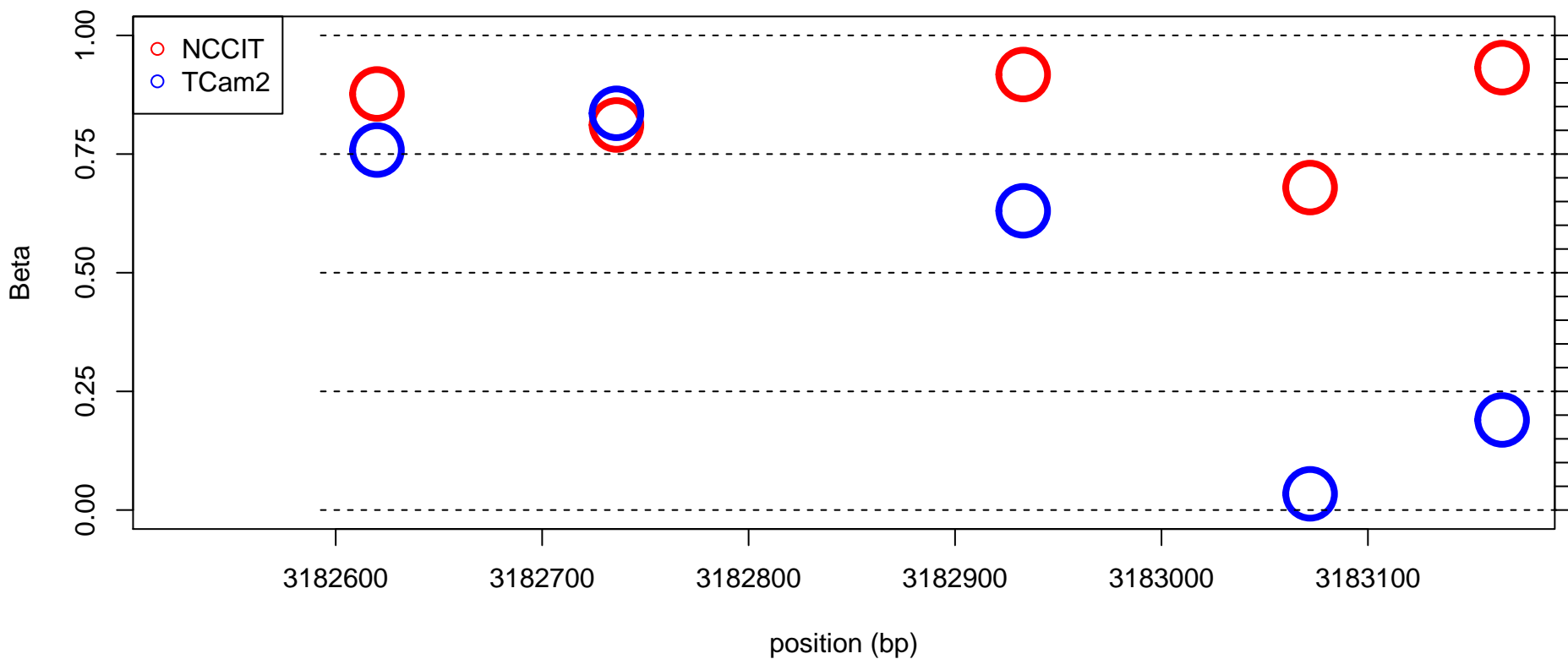

Supplement: File S1 — ZIP file containing DMRforPairs output for significant regions. Please start from the html files. (ZIP) [file pone.0098330.s008.zip › figures/113.pdf]

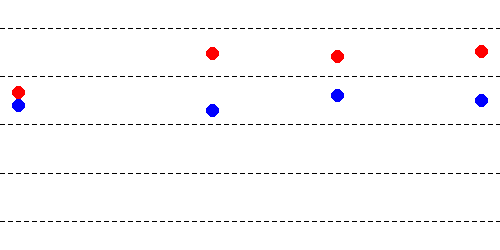

Supplement: File S1 — ZIP file containing DMRforPairs output for significant regions. Please start from the html files. (ZIP) [file pone.0098330.s008.zip › figures/114.png]

RegionID: 114, chr1:3184434–3184704–M\_values

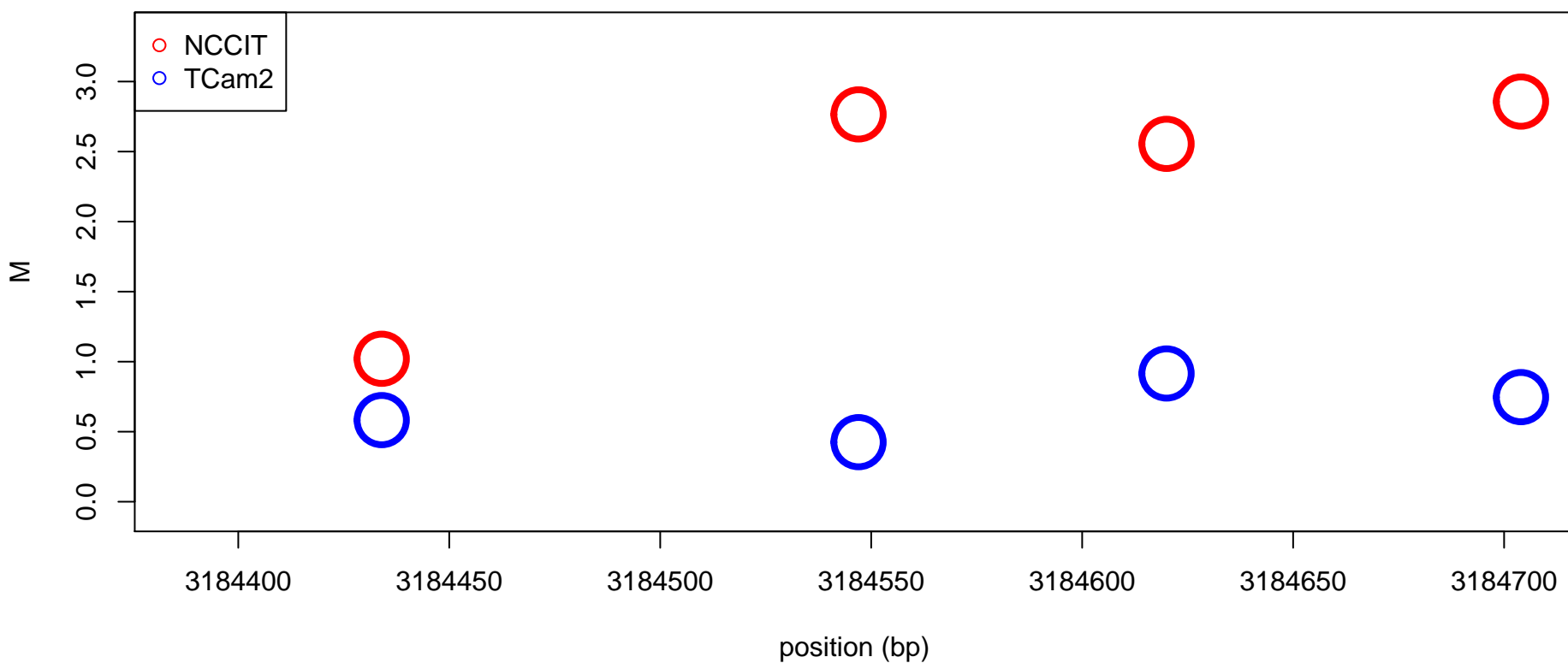

RegionID: 114, chr1:3184434–3184704–Beta\_values

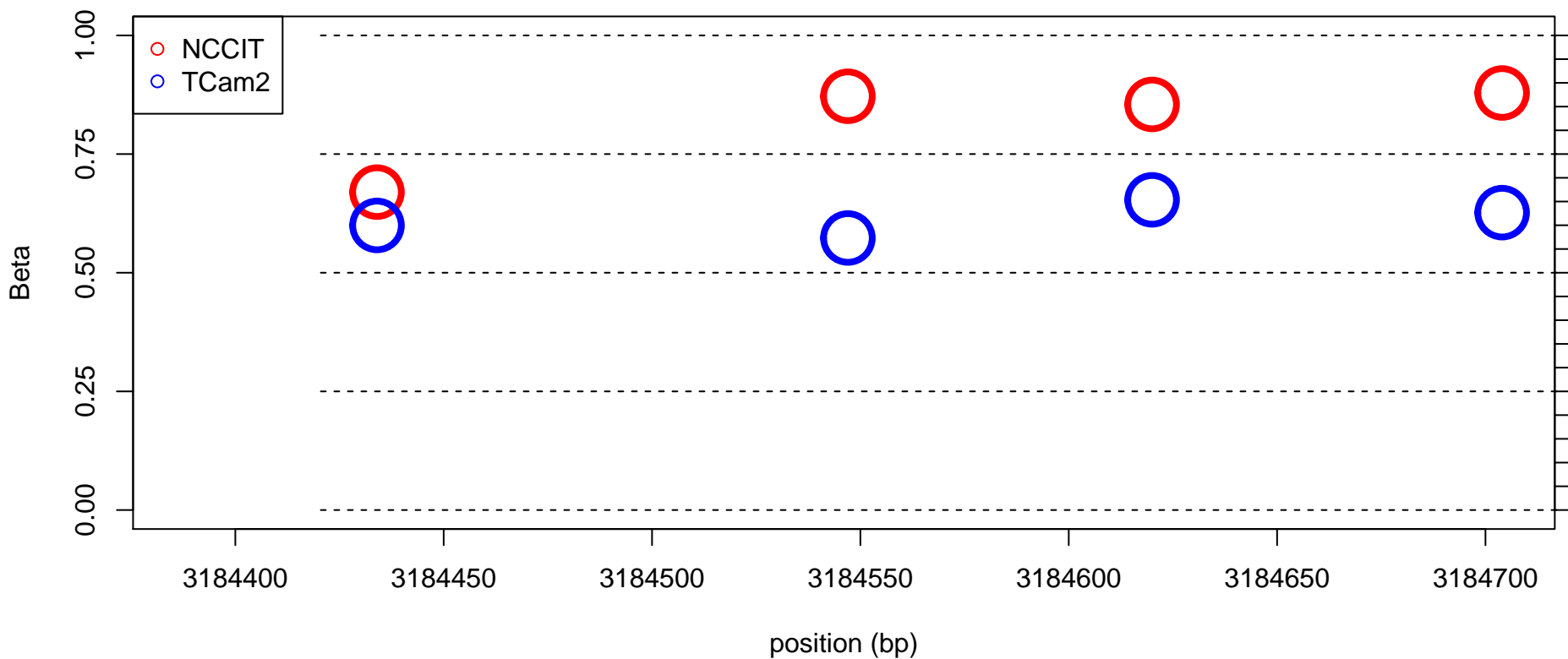

Supplement: File S1 — ZIP file containing DMRforPairs output for significant regions. Please start from the html files. (ZIP) [file pone.0098330.s008.zip › figures/114.pdf]

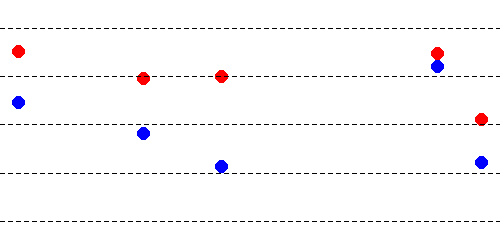

Supplement: File S1 — ZIP file containing DMRforPairs output for significant regions. Please start from the html files. (ZIP) [file pone.0098330.s008.zip › figures/118.png]

RegionID: 118, chr1:3230250–3230647–M\_values

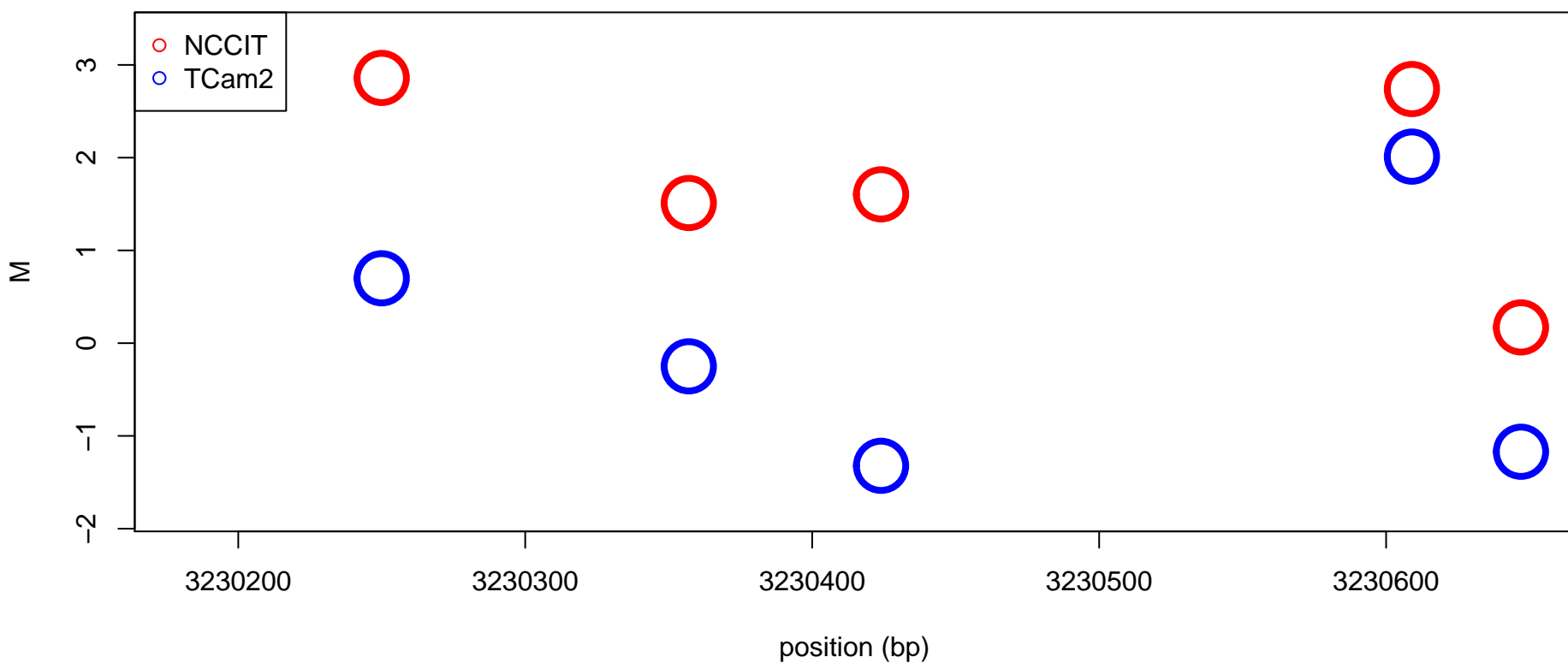

RegionID: 118, chr1:3230250–3230647–Beta\_values

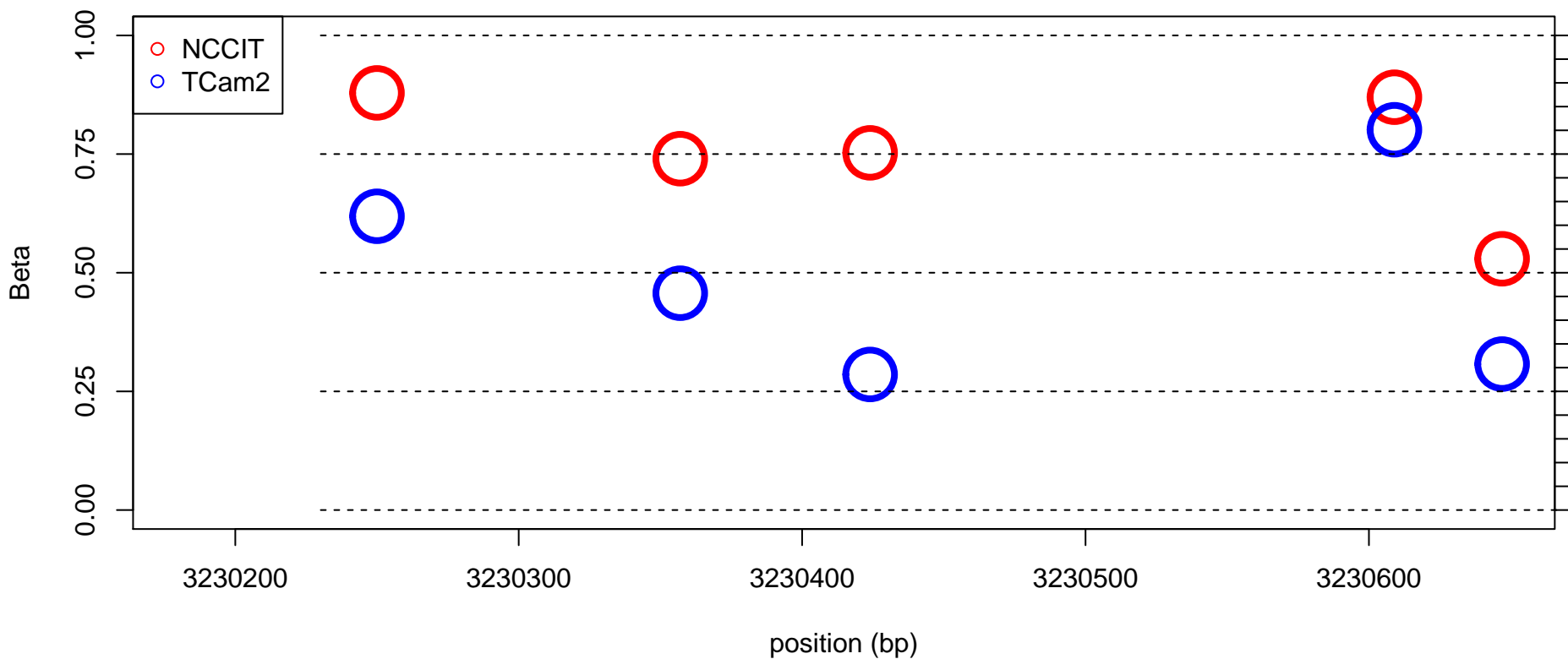

Supplement: File S1 — ZIP file containing DMRforPairs output for significant regions. Please start from the html files. (ZIP) [file pone.0098330.s008.zip › figures/118.pdf]

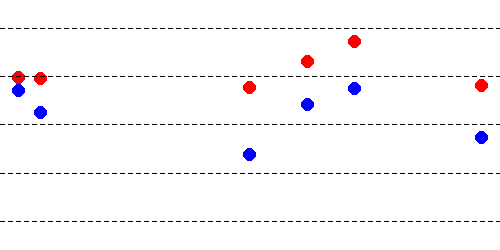

Supplement: File S1 — ZIP file containing DMRforPairs output for significant regions. Please start from the html files. (ZIP) [file pone.0098330.s008.zip › figures/124.png]

RegionID: 124, chr1:3331987-3332264-M\_values

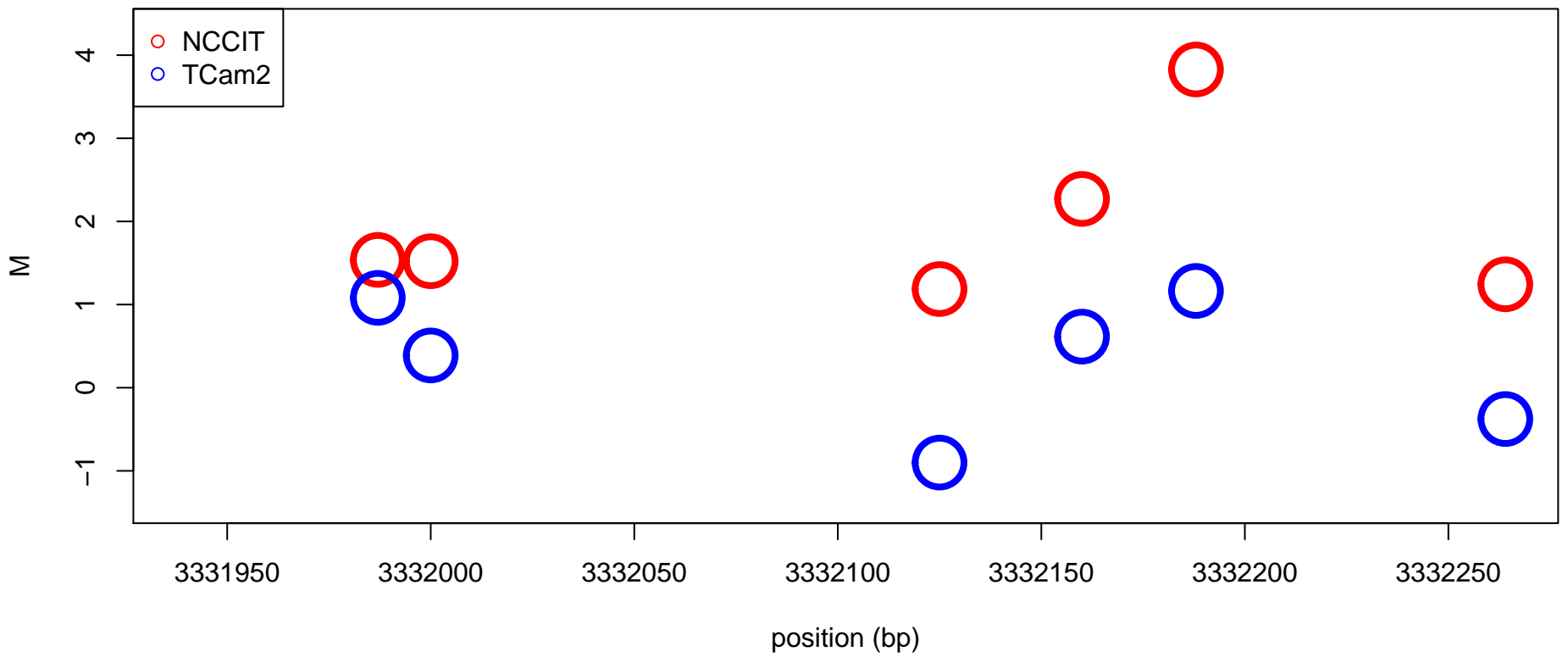

RegionID: 124, chr1:3331987-3332264-Beta\_values

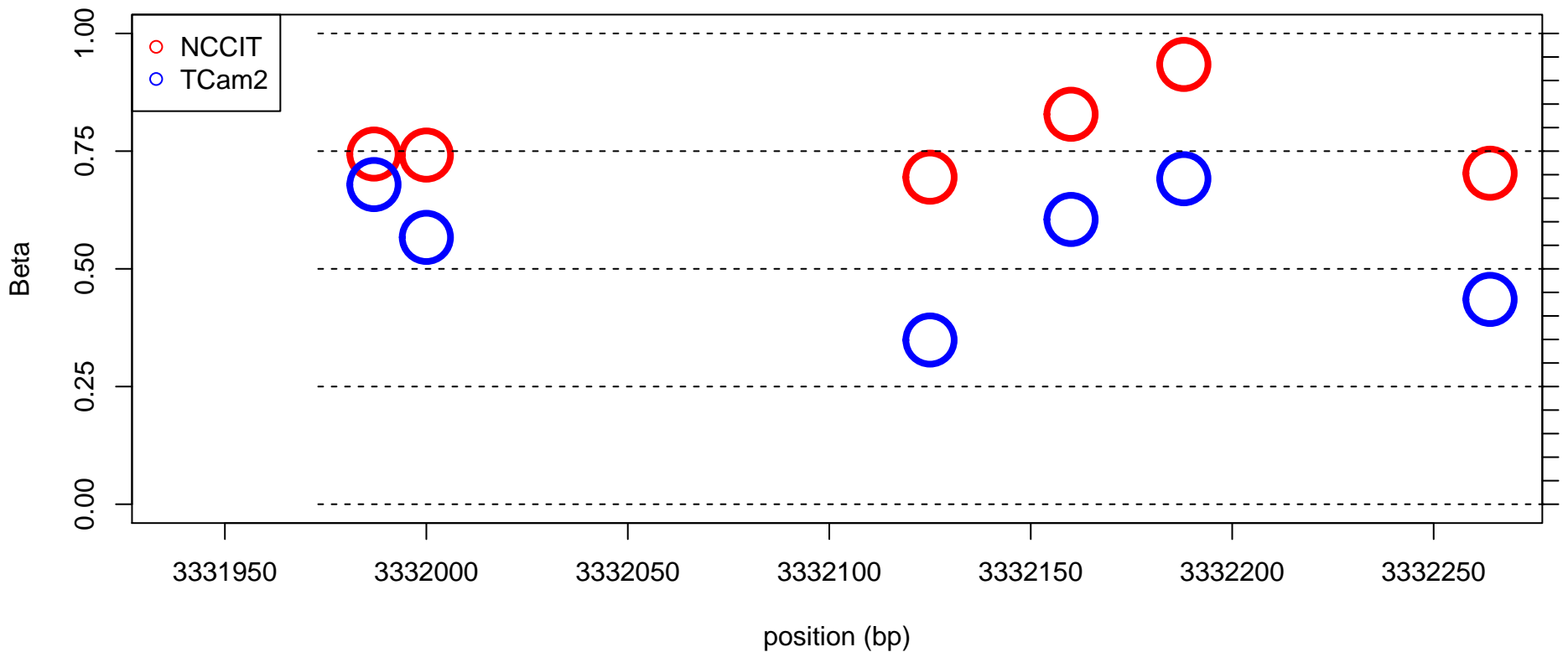

Supplement: File S1 — ZIP file containing DMRforPairs output for significant regions. Please start from the html files. (ZIP) [file pone.0098330.s008.zip › figures/124.pdf]

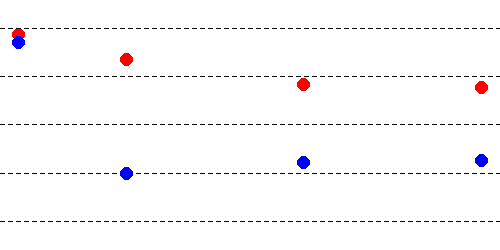

Supplement: File S1 — ZIP file containing DMRforPairs output for significant regions. Please start from the html files. (ZIP) [file pone.0098330.s008.zip › figures/128.png]

RegionID: 128, chr1:3459949–3460467–M\_values

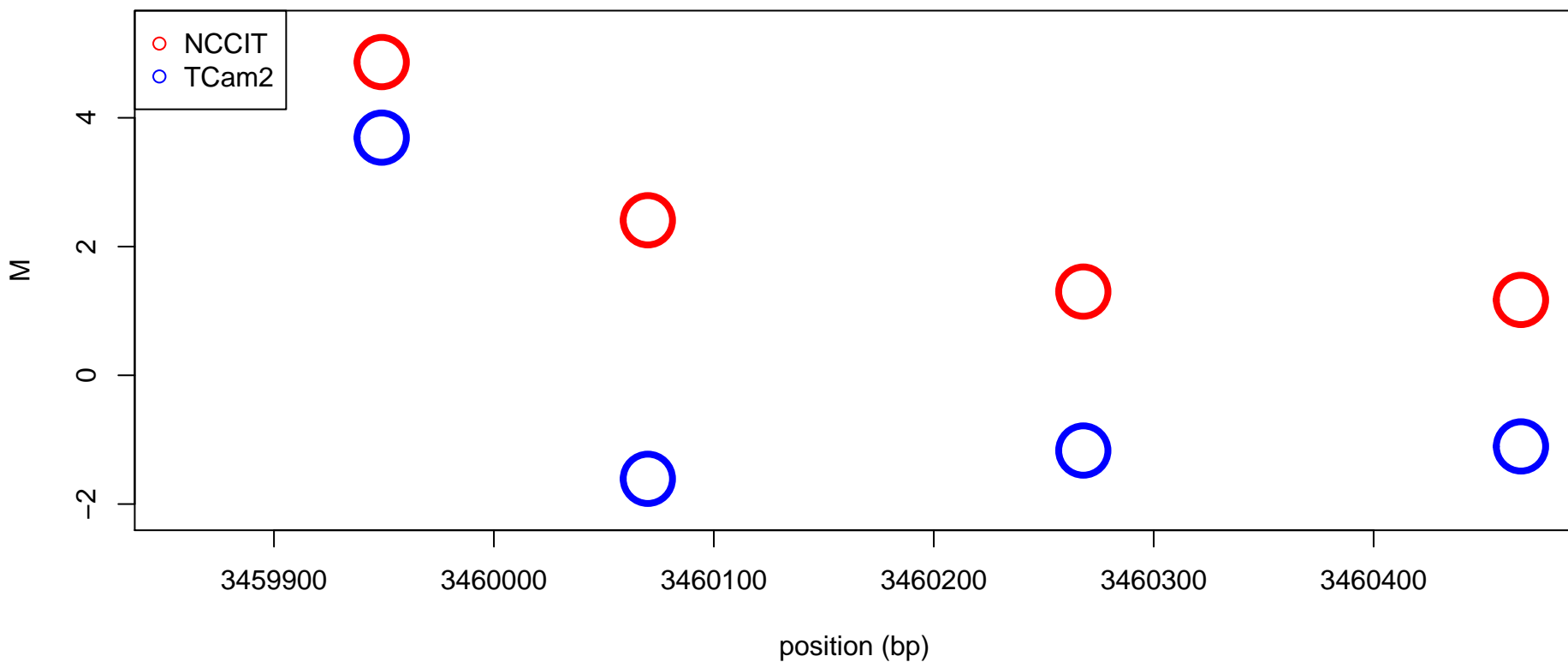

RegionID: 128, chr1:3459949–3460467–Beta\_values

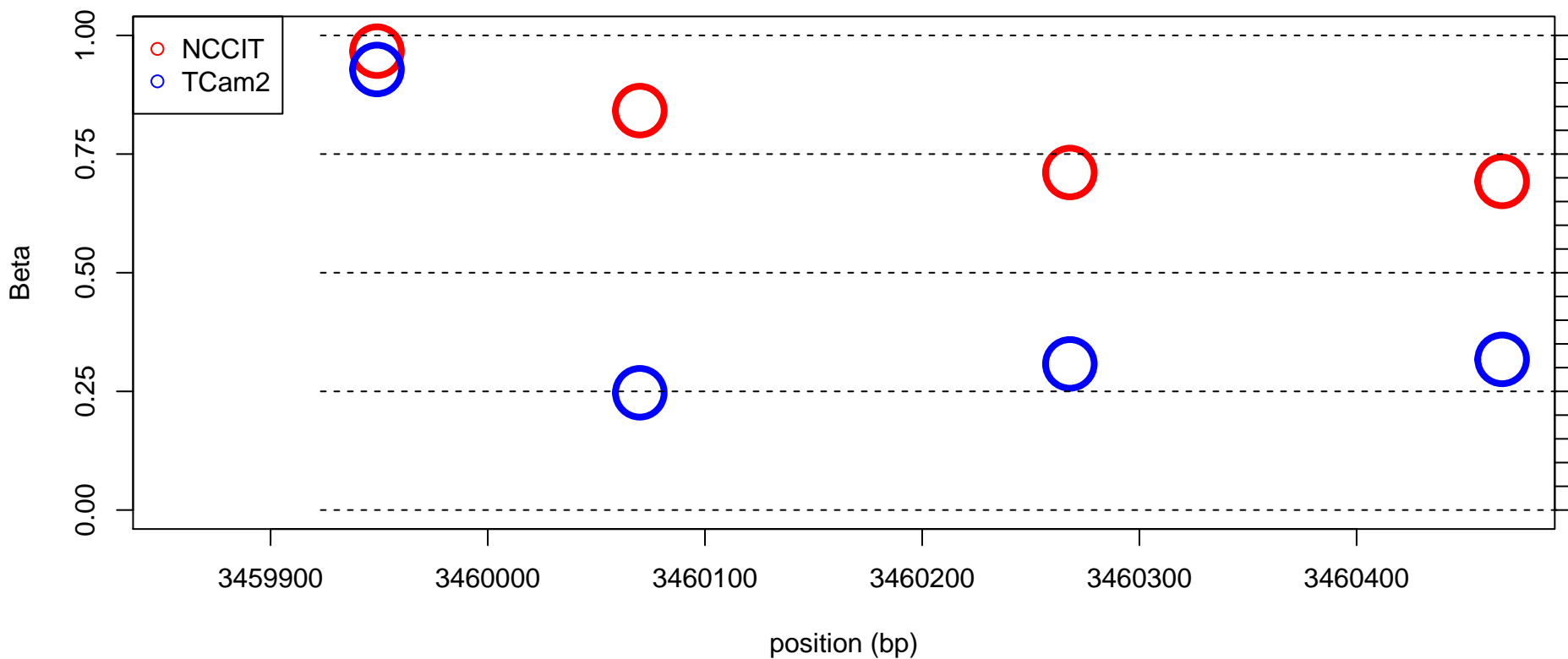

Supplement: File S1 — ZIP file containing DMRforPairs output for significant regions. Please start from the html files. (ZIP) [file pone.0098330.s008.zip › figures/128.pdf]

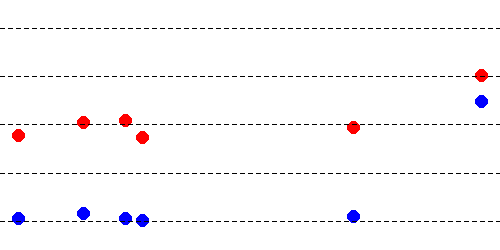

Supplement: File S1 — ZIP file containing DMRforPairs output for significant regions. Please start from the html files. (ZIP) [file pone.0098330.s008.zip › figures/141.png]

RegionID: 141, chr1:3634811-3635210-M\_values

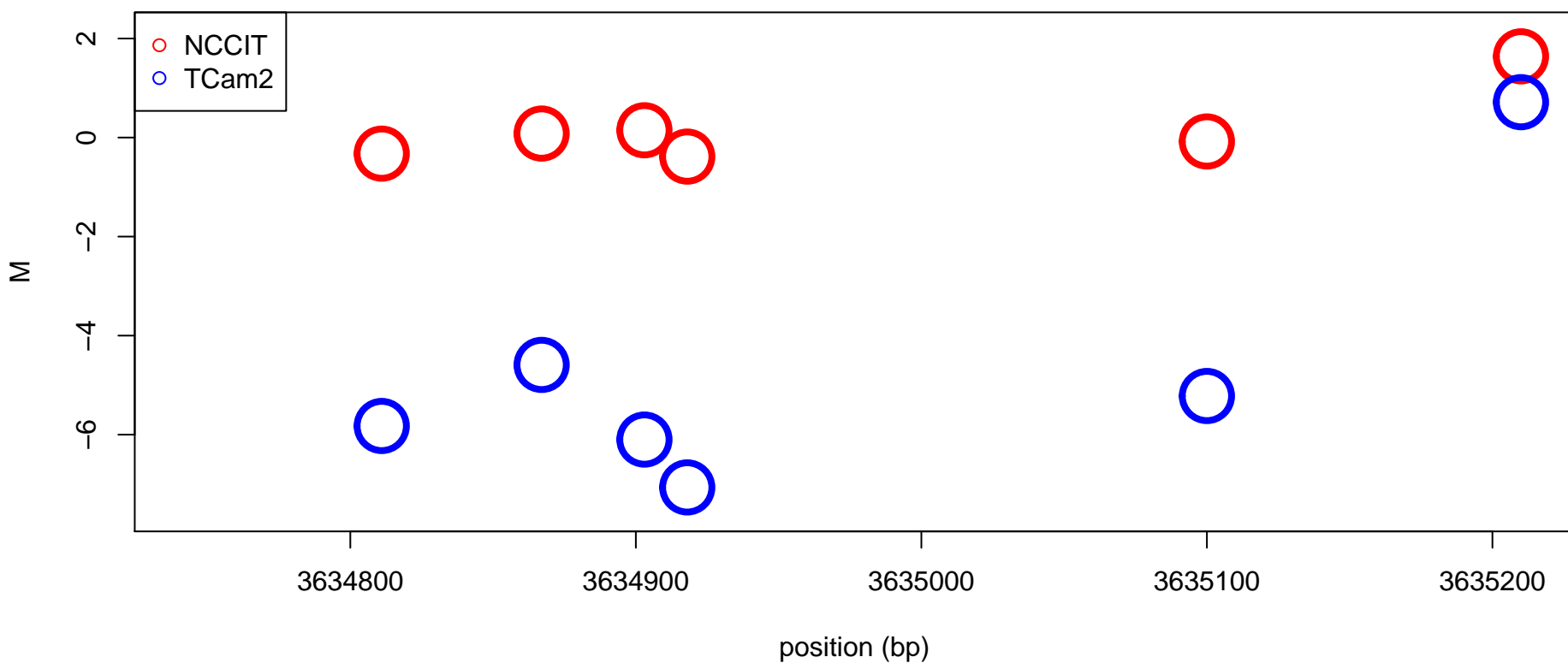

RegionID: 141, chr1:3634811-3635210-Beta\_values

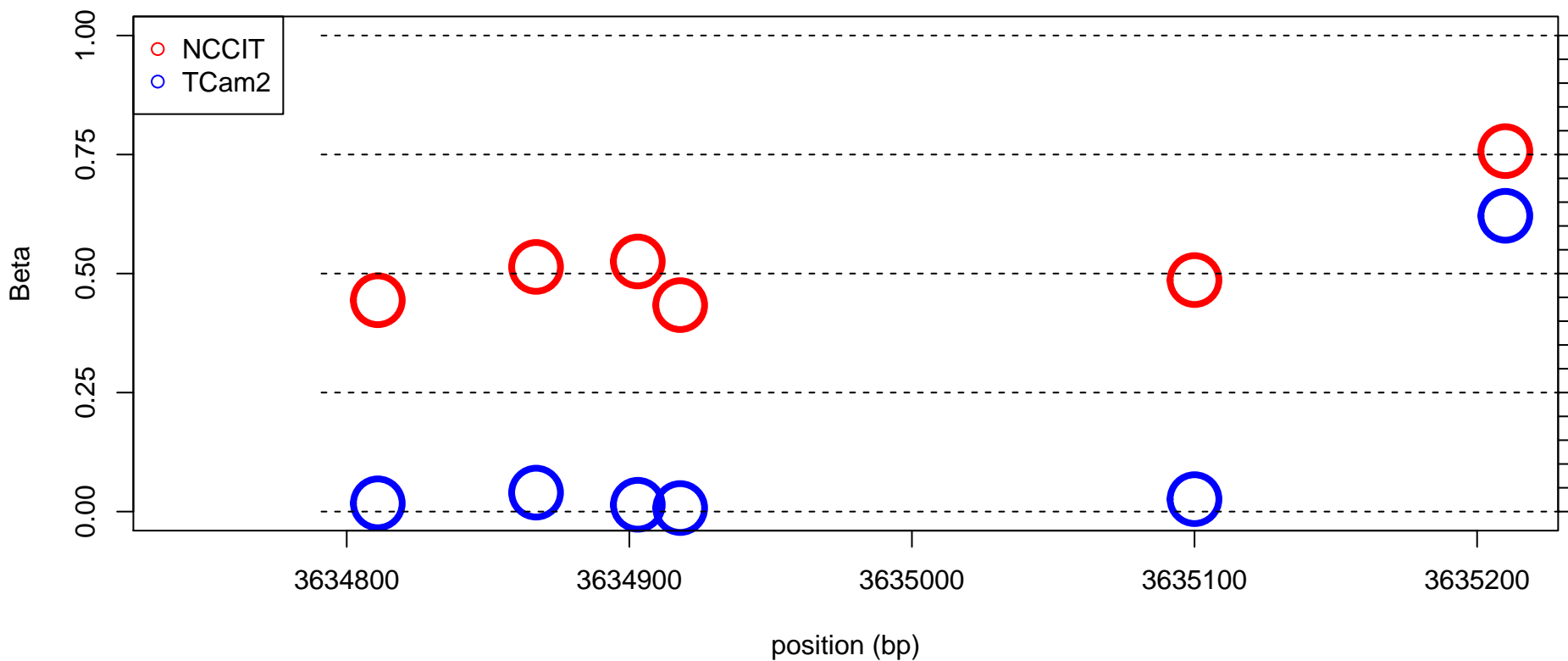

Supplement: File S1 — ZIP file containing DMRforPairs output for significant regions. Please start from the html files. (ZIP) [file pone.0098330.s008.zip › figures/141.pdf]

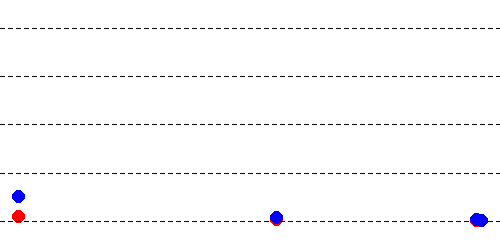

Supplement: File S1 — ZIP file containing DMRforPairs output for significant regions. Please start from the html files. (ZIP) [file pone.0098330.s008.zip › figures/147.png]

RegionID: 147, chr1:3772978–3773316–M\_values

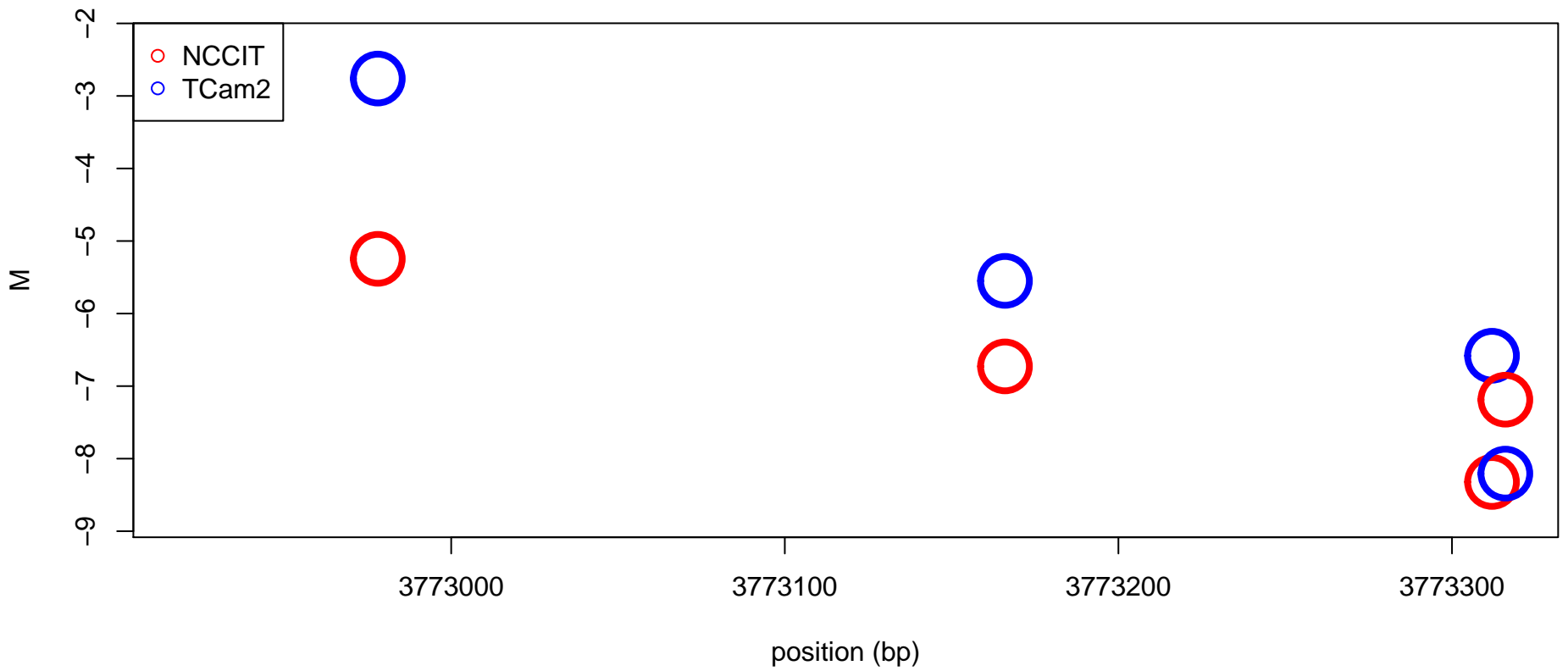

RegionID: 147, chr1:3772978–3773316–Beta\_values

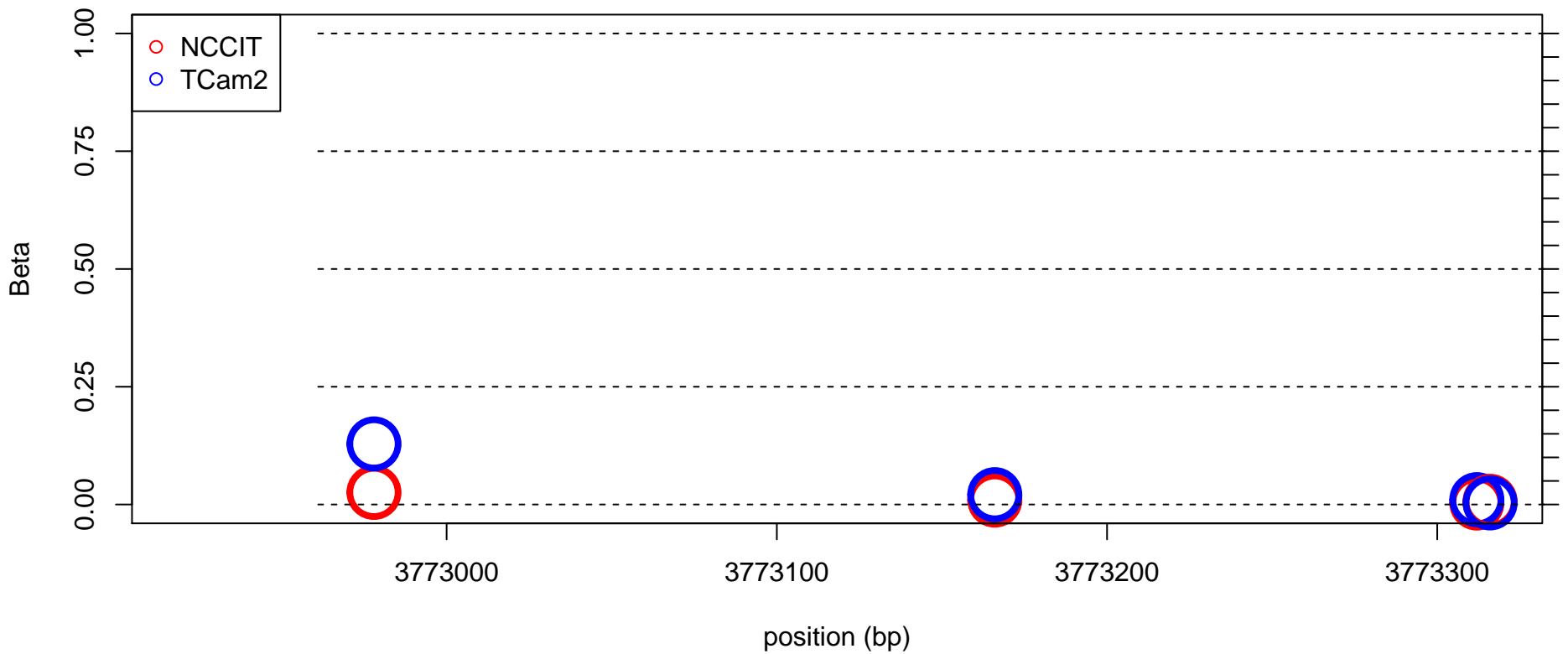

Supplement: File S1 — ZIP file containing DMRforPairs output for significant regions. Please start from the html files. (ZIP) [file pone.0098330.s008.zip › figures/147.pdf]

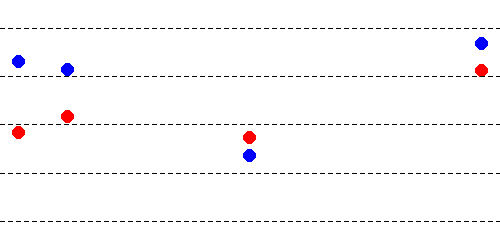

Supplement: File S1 — ZIP file containing DMRforPairs output for significant regions. Please start from the html files. (ZIP) [file pone.0098330.s008.zip › figures/148.png]

RegionID: 148, chr1:3775078–3775334–M\_values

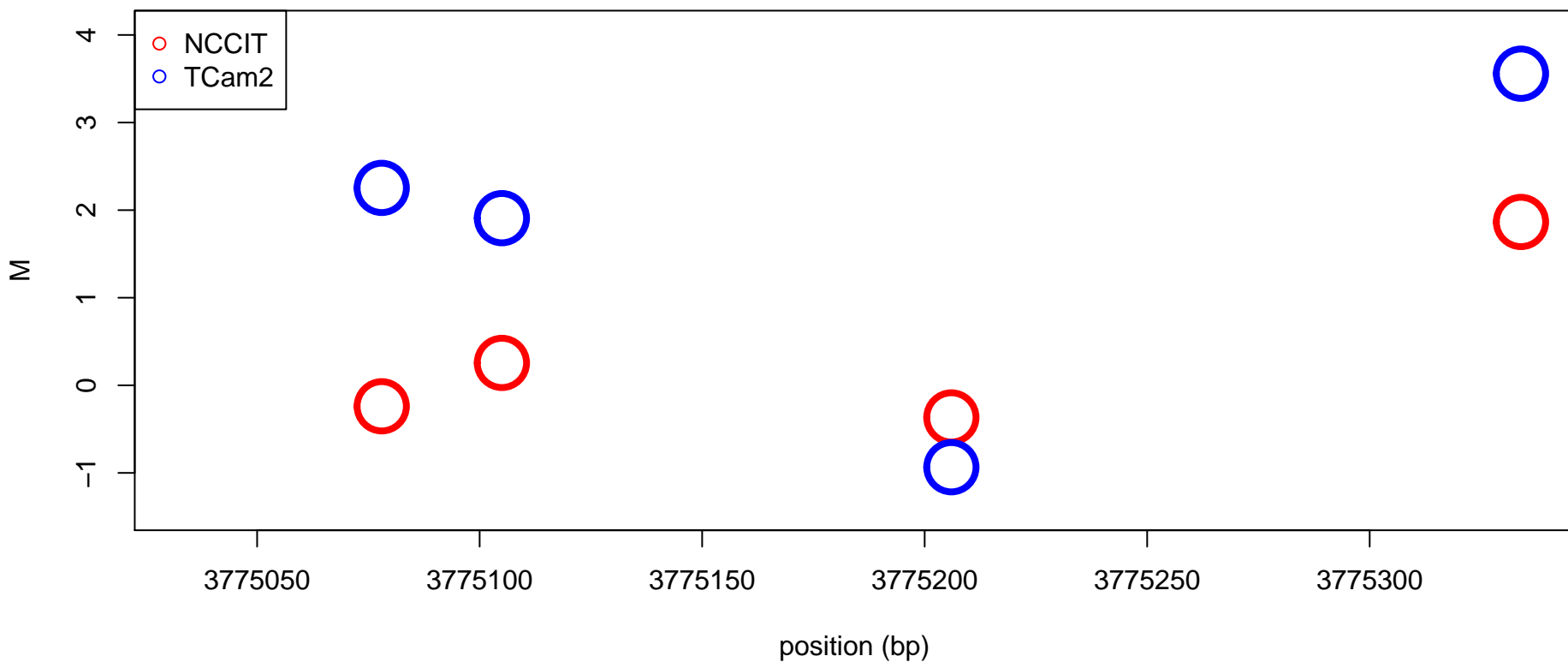

RegionID: 148, chr1:3775078–3775334–Beta\_values

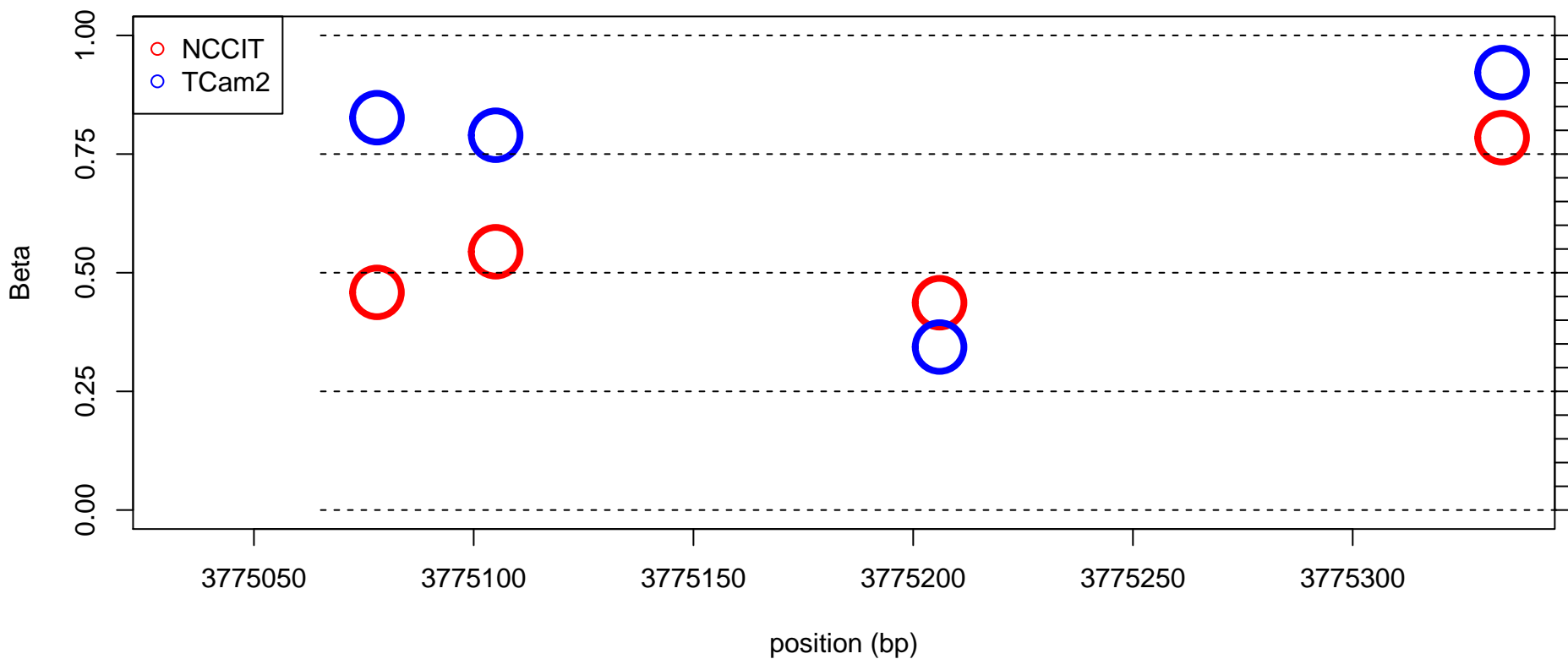

Supplement: File S1 — ZIP file containing DMRforPairs output for significant regions. Please start from the html files. (ZIP) [file pone.0098330.s008.zip › figures/148.pdf]

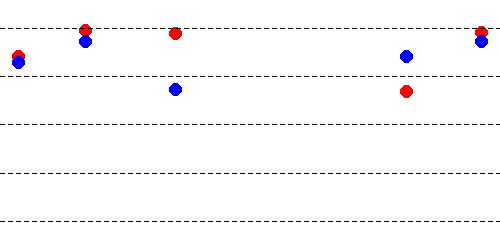

Supplement: File S1 — ZIP file containing DMRforPairs output for significant regions. Please start from the html files. (ZIP) [file pone.0098330.s008.zip › figures/154.png]

RegionID: 154, chr1:5932351–5932678–M\_values

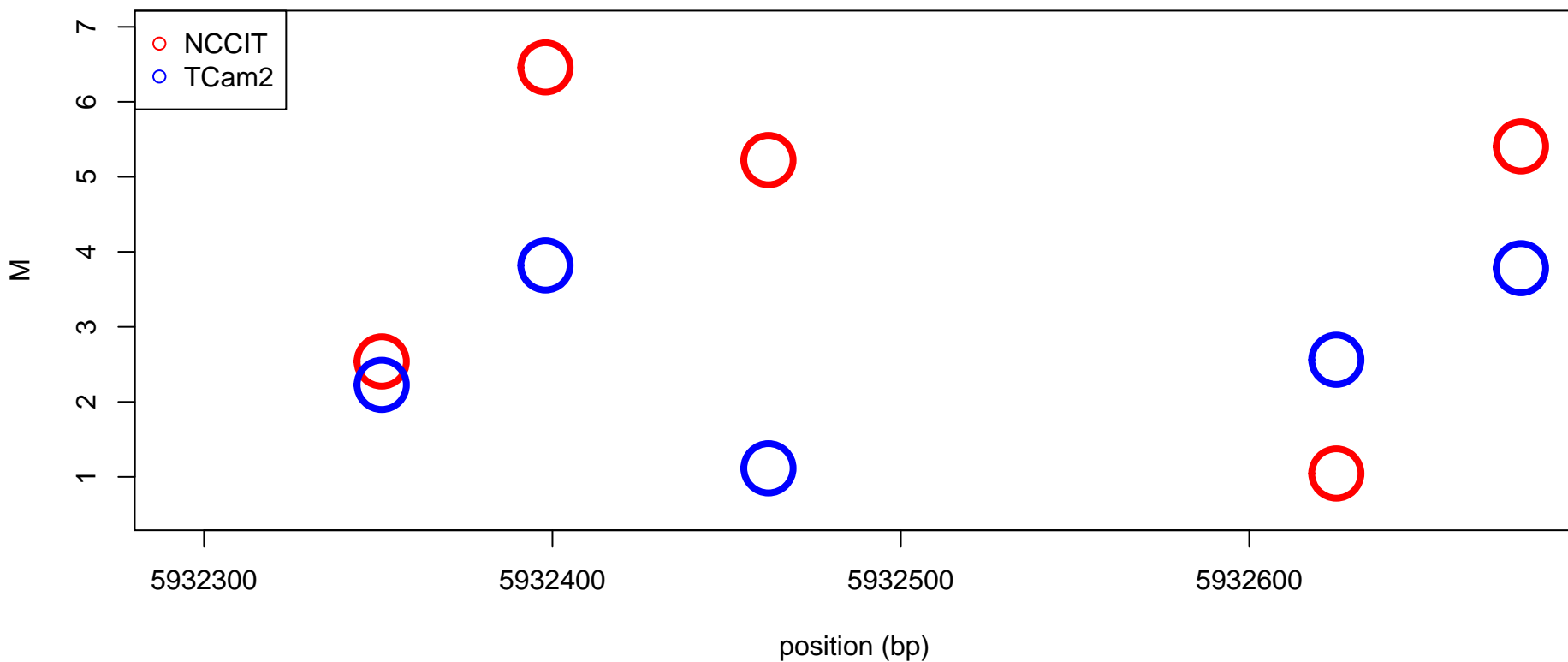

RegionID: 154, chr1:5932351–5932678–Beta\_values

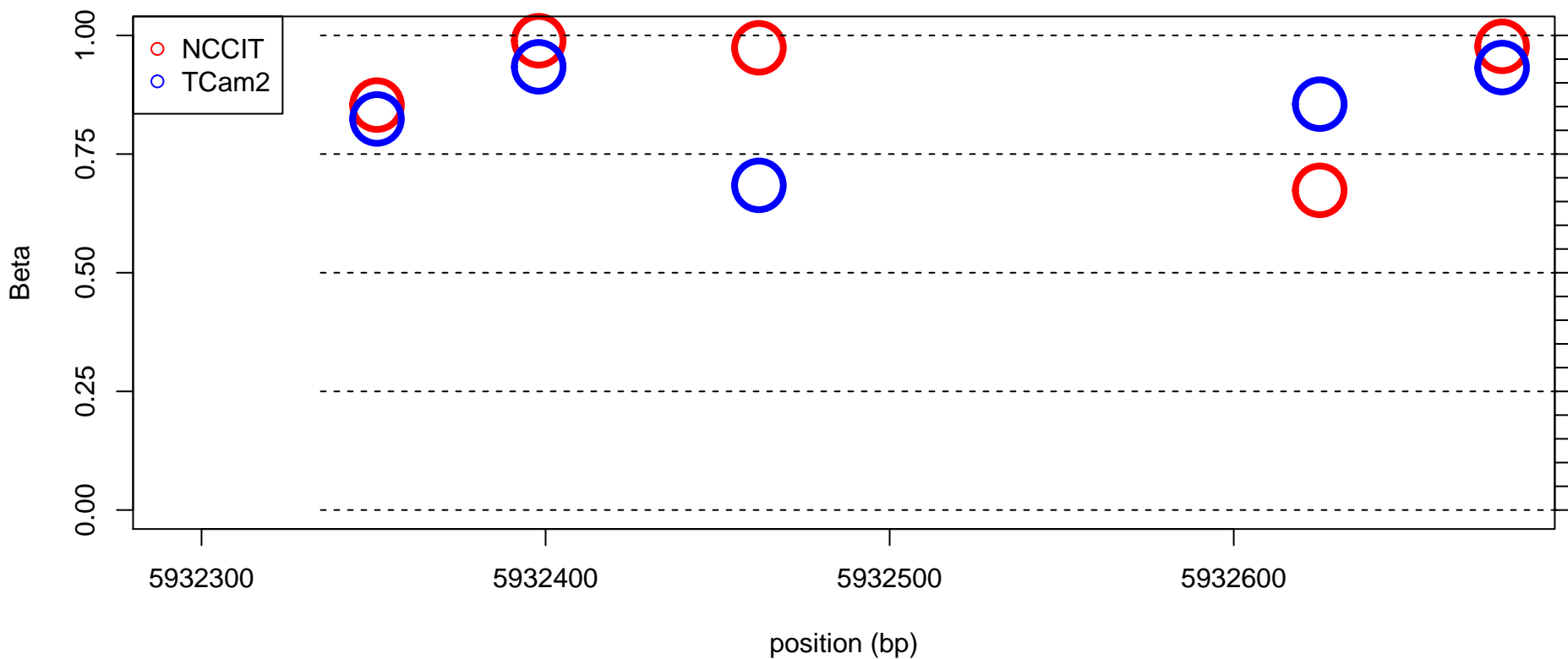

Supplement: File S1 — ZIP file containing DMRforPairs output for significant regions. Please start from the html files. (ZIP) [file pone.0098330.s008.zip › figures/154.pdf]

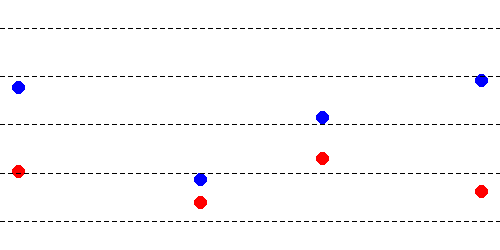

Supplement: File S1 — ZIP file containing DMRforPairs output for significant regions. Please start from the html files. (ZIP) [file pone.0098330.s008.zip › figures/164.png]

RegionID: 164, chr1:6304272–6304499–M\_values

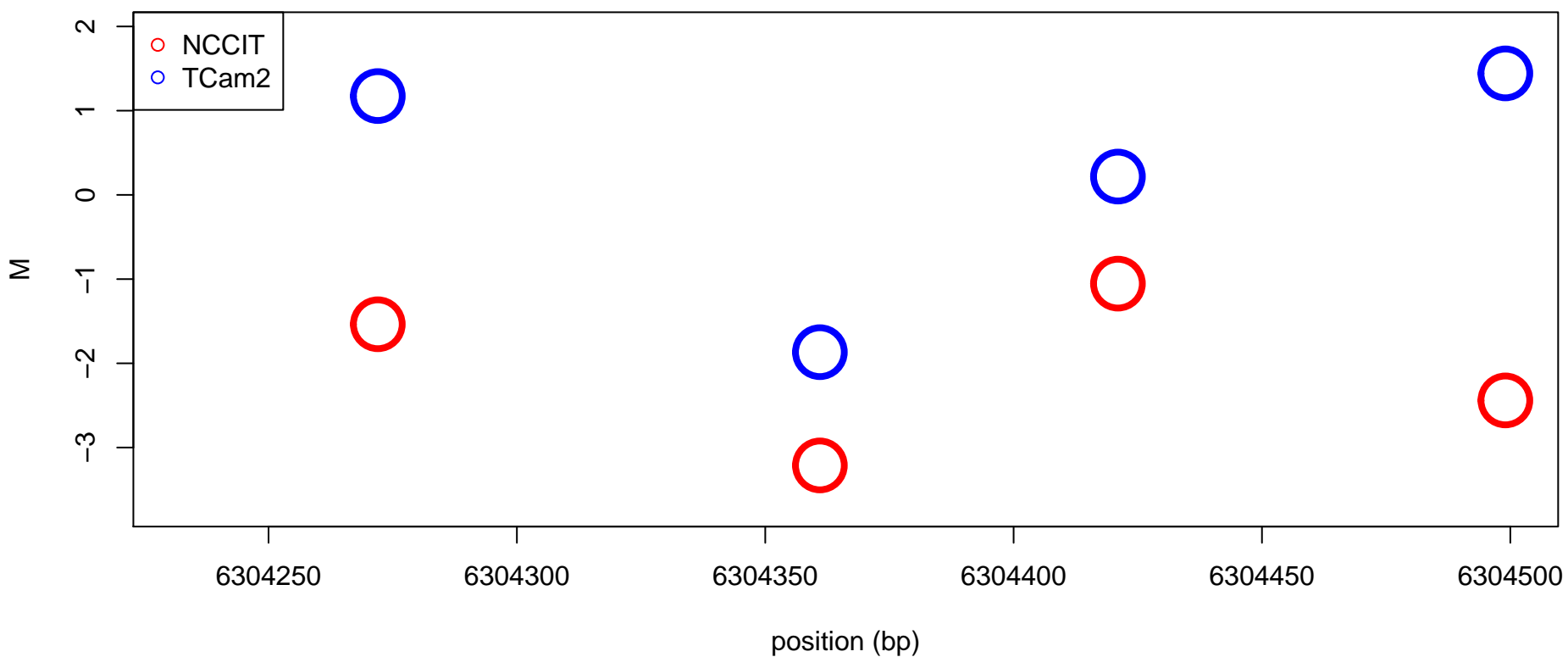

RegionID: 164, chr1:6304272–6304499–Beta\_values

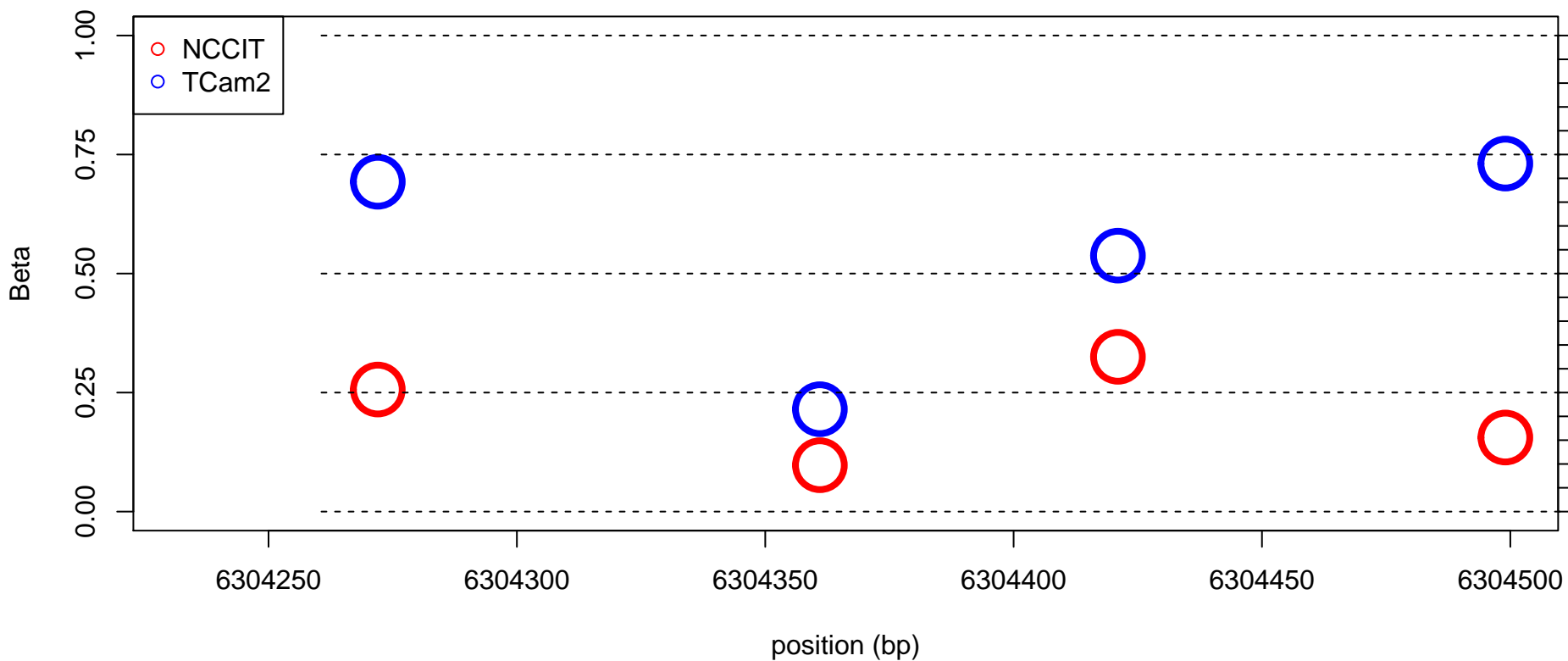

Supplement: File S1 — ZIP file containing DMRforPairs output for significant regions. Please start from the html files. (ZIP) [file pone.0098330.s008.zip › figures/164.pdf]

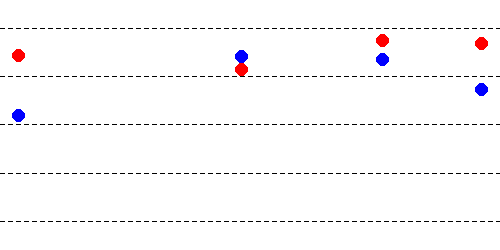

Supplement: File S1 — ZIP file containing DMRforPairs output for significant regions. Please start from the html files. (ZIP) [file pone.0098330.s008.zip › figures/166.png]

RegionID: 166, chr1:6341140–6341327–M\_values

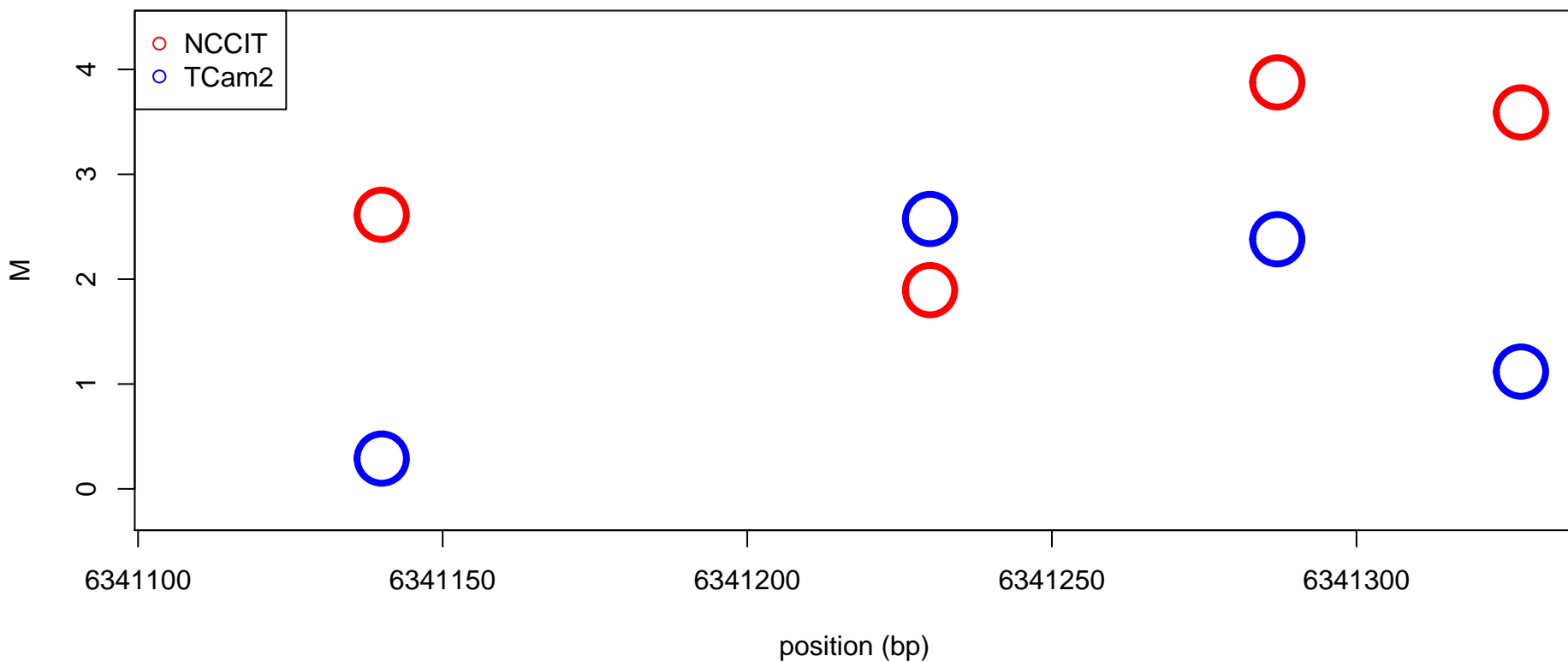

RegionID: 166, chr1:6341140–6341327–Beta\_values

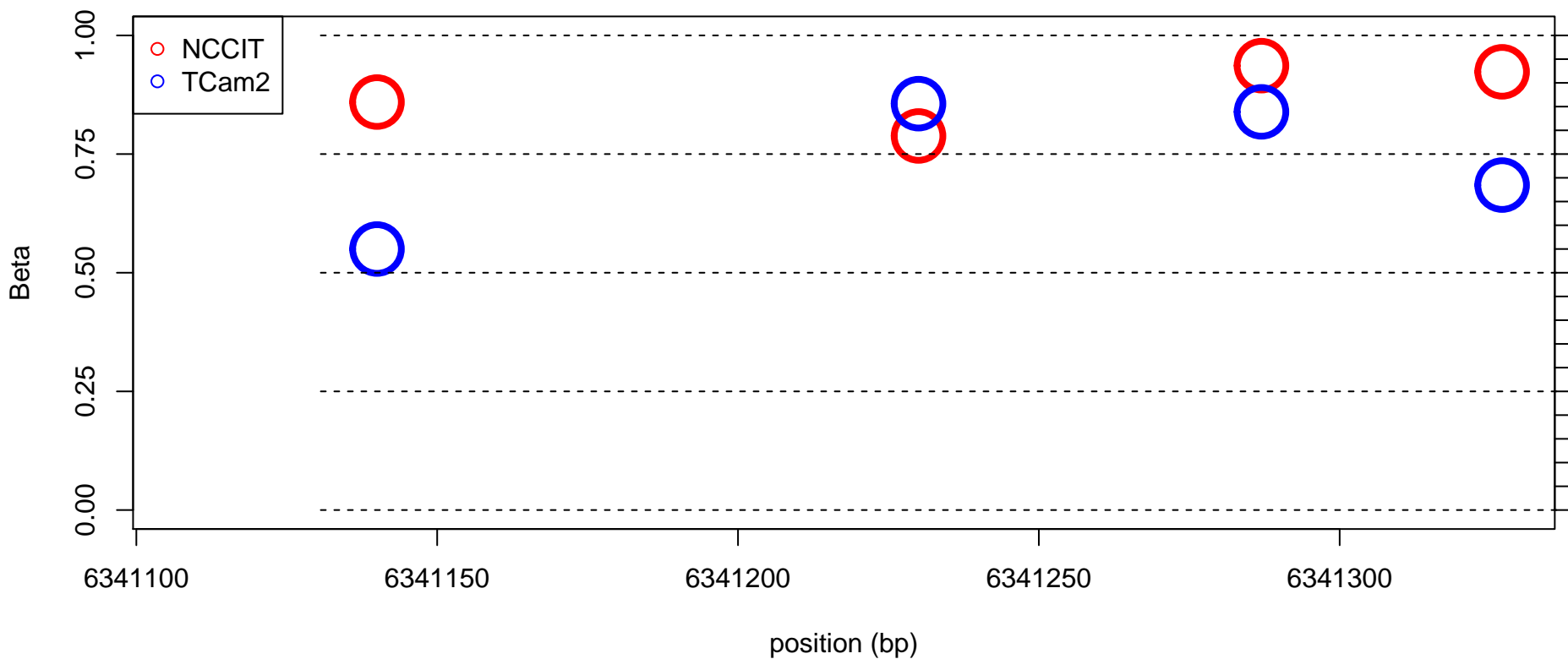

Supplement: File S1 — ZIP file containing DMRforPairs output for significant regions. Please start from the html files. (ZIP) [file pone.0098330.s008.zip › figures/166.pdf]

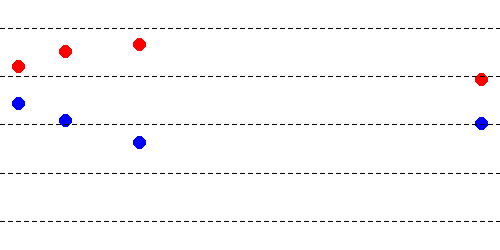

Supplement: File S1 — ZIP file containing DMRforPairs output for significant regions. Please start from the html files. (ZIP) [file pone.0098330.s008.zip › figures/172.png]

RegionID: 172, chr1:6515580–6515748–M\_values

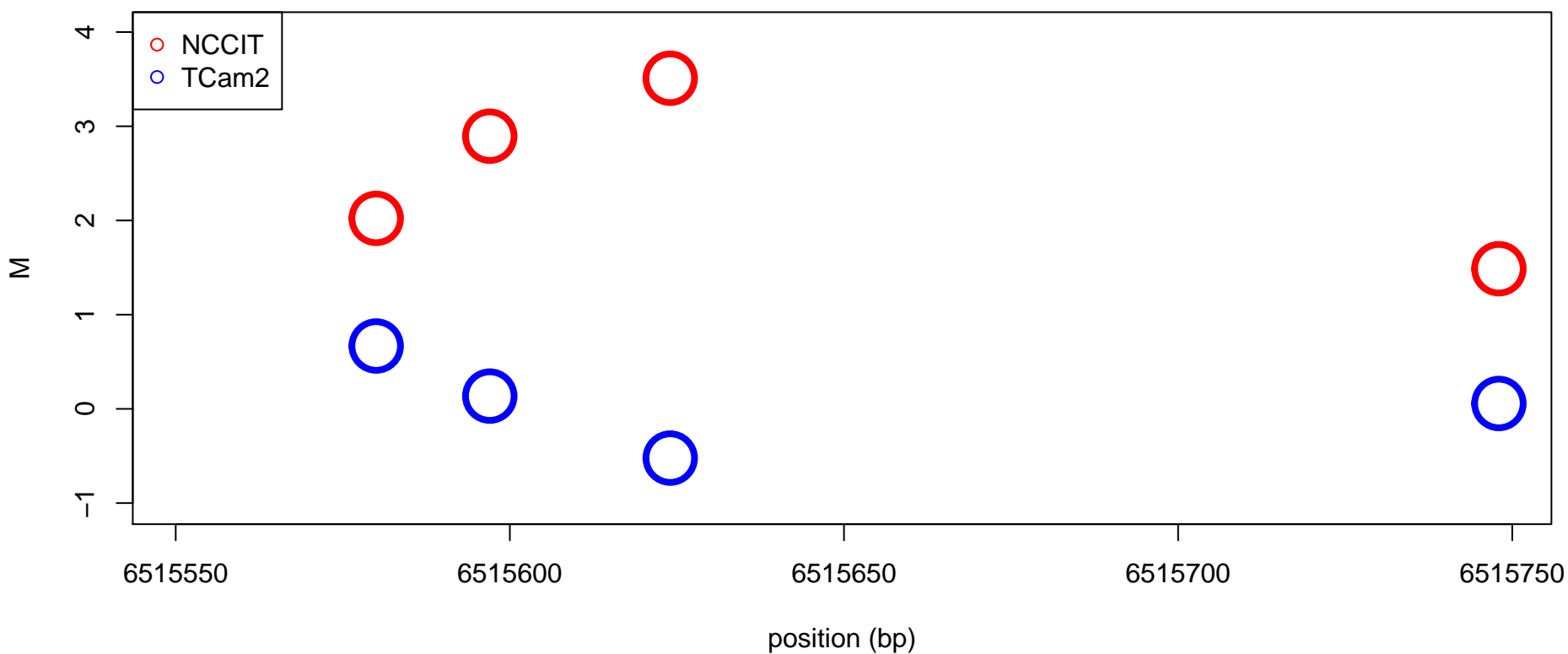

RegionID: 172, chr1:6515580–6515748–Beta\_values

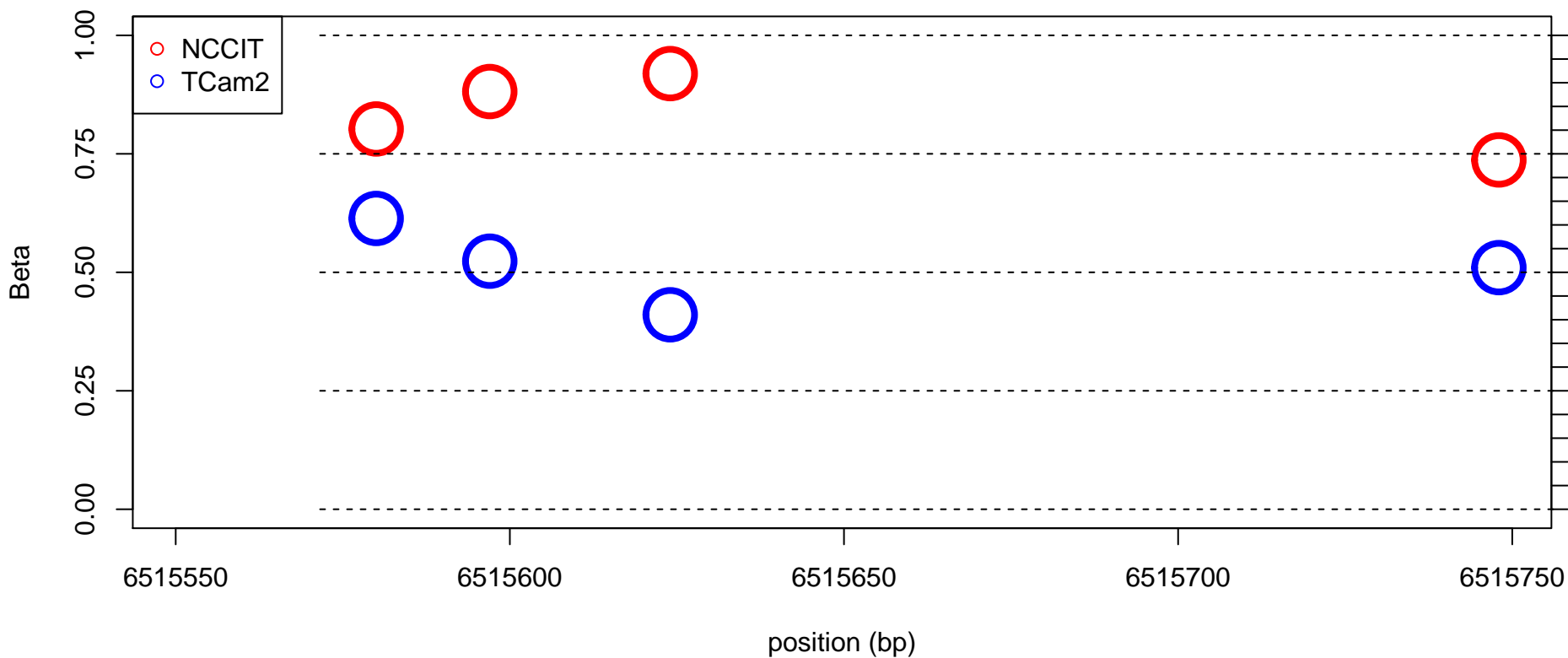

Supplement: File S1 — ZIP file containing DMRforPairs output for significant regions. Please start from the html files. (ZIP) [file pone.0098330.s008.zip › figures/172.pdf]

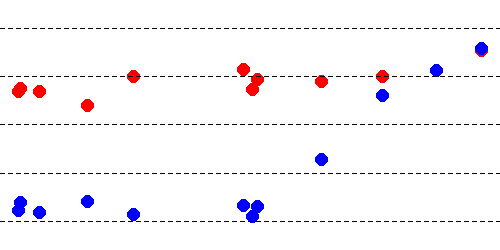

Supplement: File S1 — ZIP file containing DMRforPairs output for significant regions. Please start from the html files. (ZIP) [file pone.0098330.s008.zip › figures/173.png]

RegionID: 173, chr1:6526049–6526583–M\_values

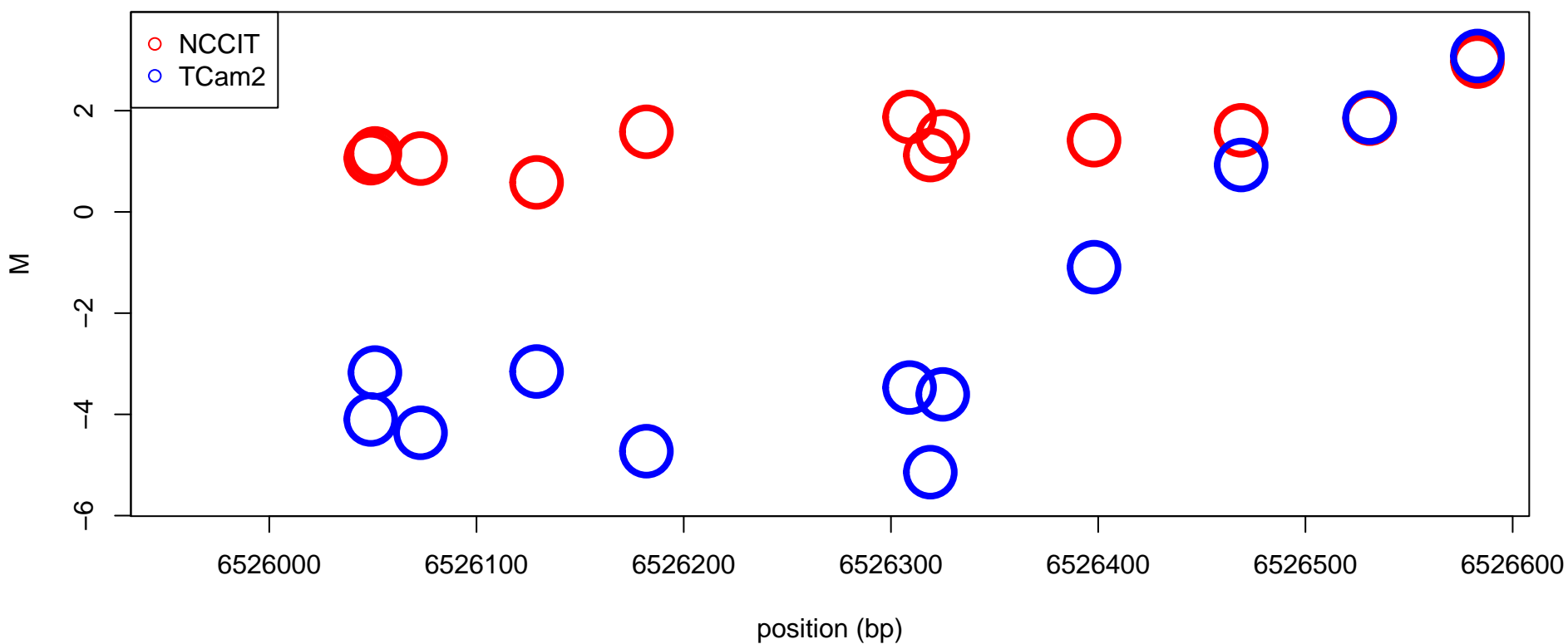

RegionID: 173, chr1:6526049–6526583–Beta\_values

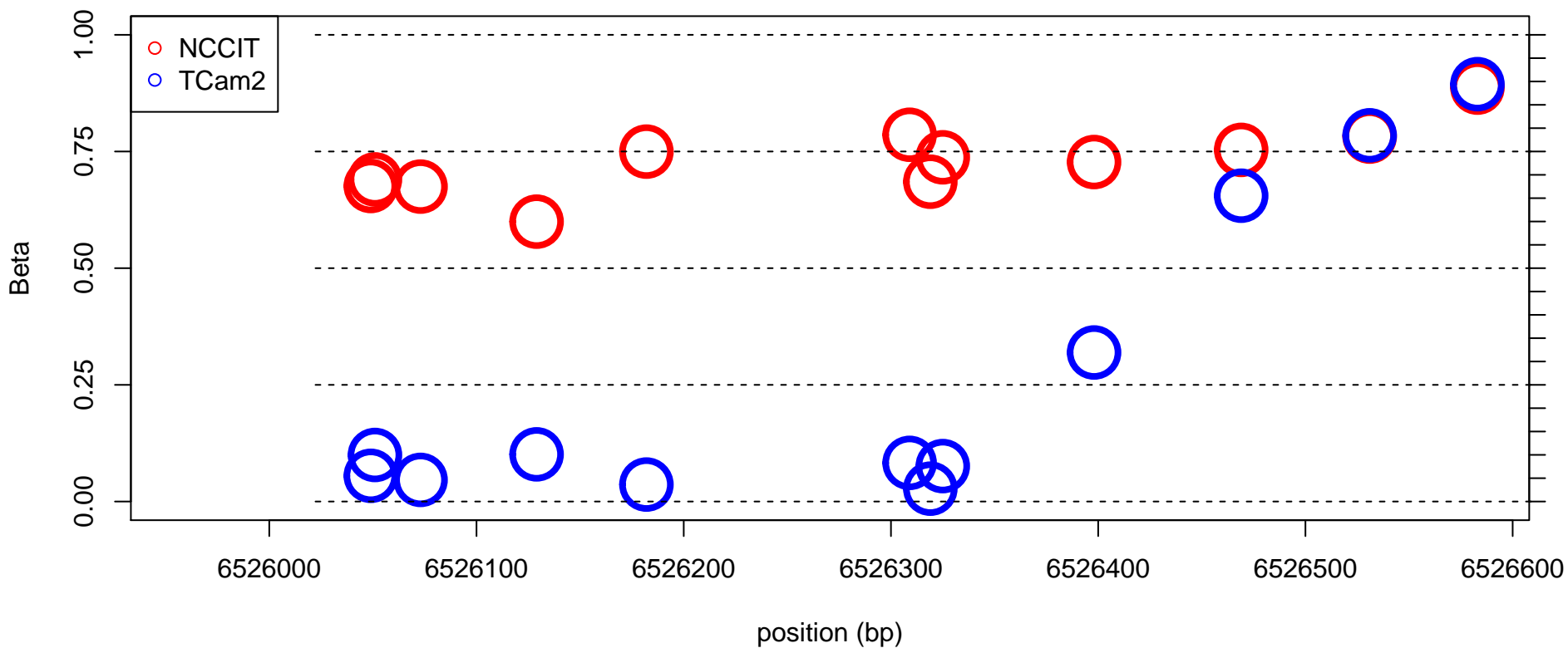

Supplement: File S1 — ZIP file containing DMRforPairs output for significant regions. Please start from the html files. (ZIP) [file pone.0098330.s008.zip › figures/173.pdf]

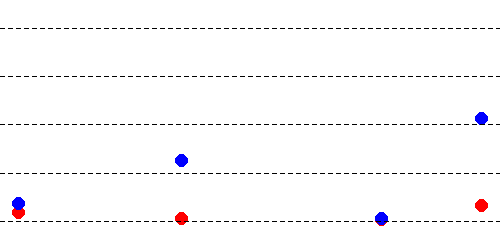

Supplement: File S1 — ZIP file containing DMRforPairs output for significant regions. Please start from the html files. (ZIP) [file pone.0098330.s008.zip › figures/183.png]

RegionID: 183, chr1:7023495–7023864–M\_values

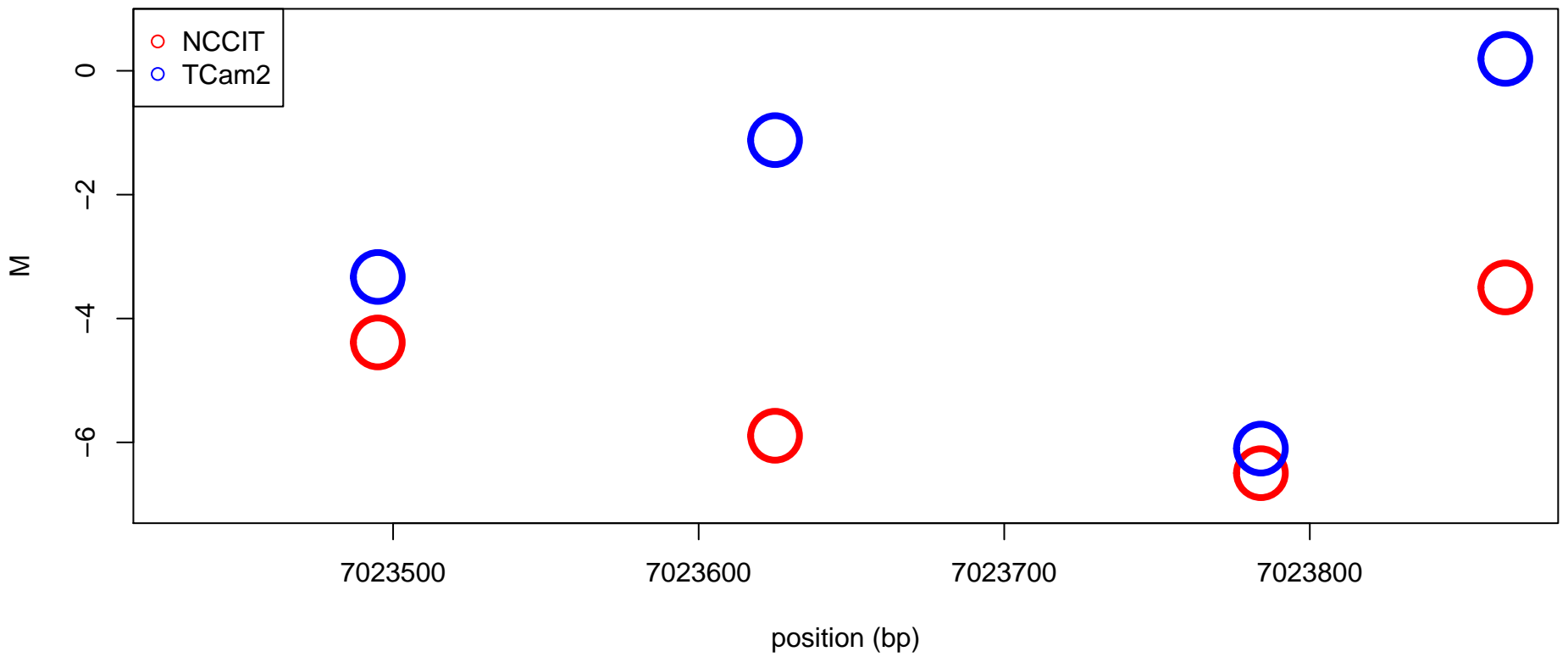

RegionID: 183, chr1:7023495–7023864–Beta\_values

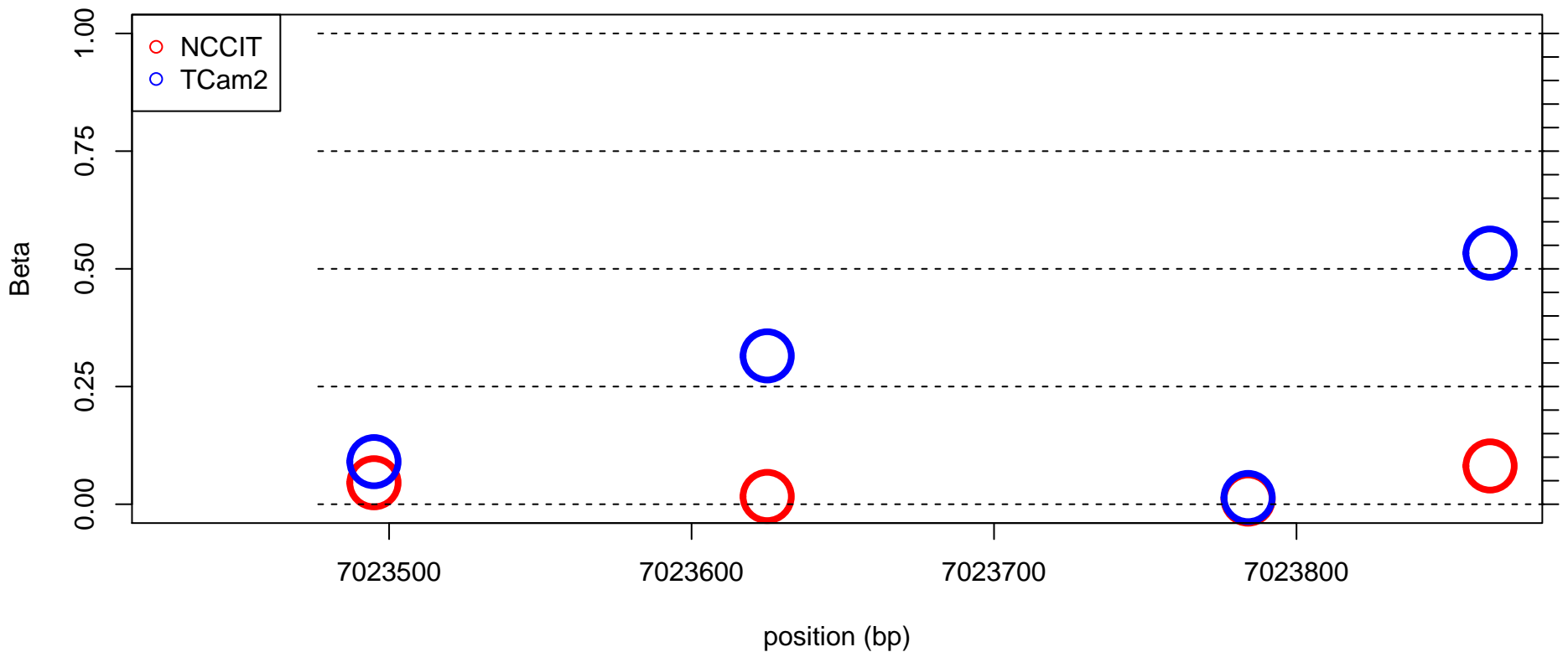

Supplement: File S1 — ZIP file containing DMRforPairs output for significant regions. Please start from the html files. (ZIP) [file pone.0098330.s008.zip › figures/183.pdf]

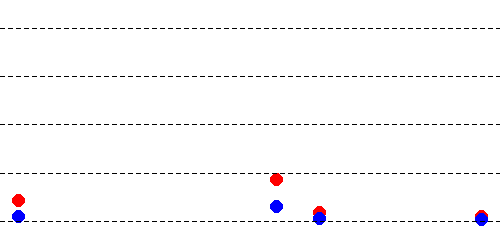

Supplement: File S1 — ZIP file containing DMRforPairs output for significant regions. Please start from the html files. (ZIP) [file pone.0098330.s008.zip › figures/186.png]

RegionID: 186, chr1:7740243-7740286-M\_values

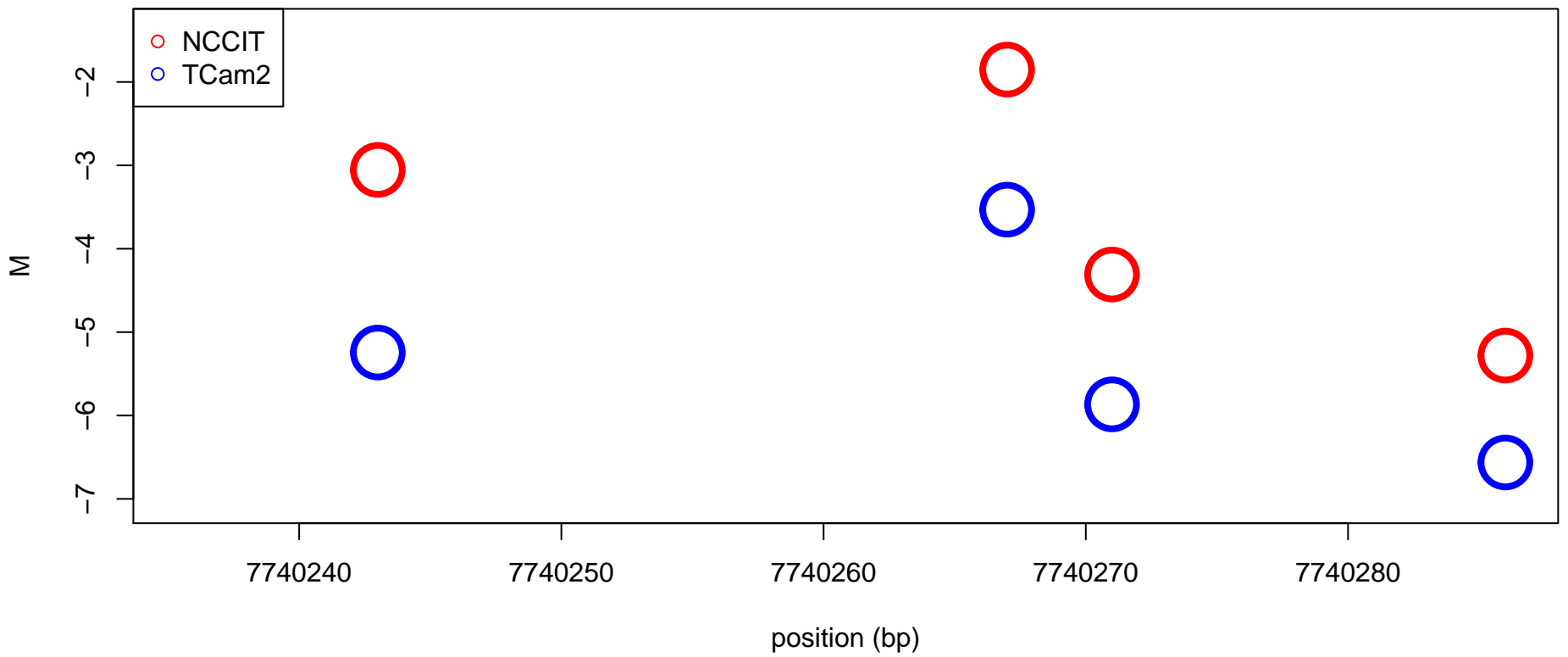

RegionID: 186, chr1:7740243-7740286-Beta\_values

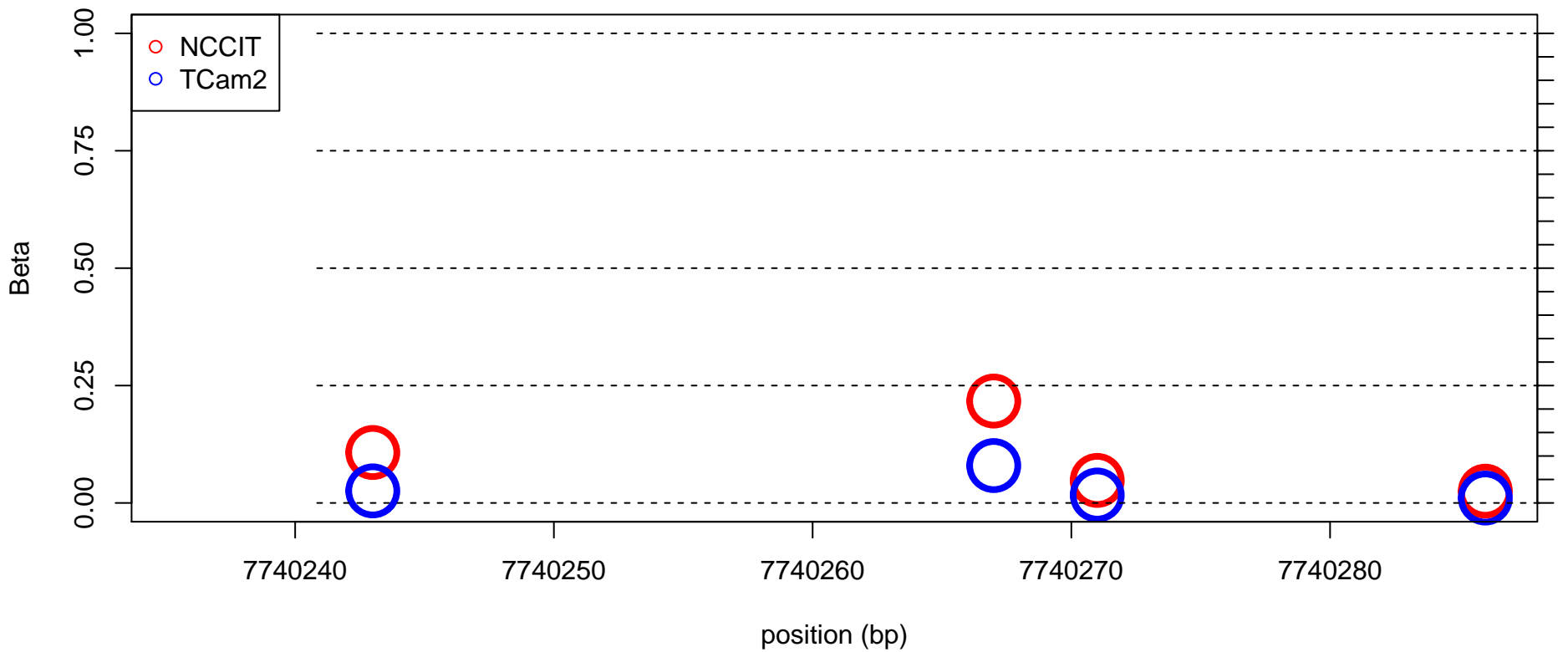

Supplement: File S1 — ZIP file containing DMRforPairs output for significant regions. Please start from the html files. (ZIP) [file pone.0098330.s008.zip › figures/186.pdf]

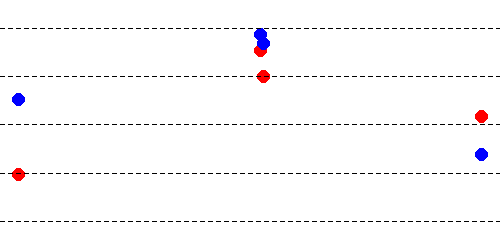

Supplement: File S1 — ZIP file containing DMRforPairs output for significant regions. Please start from the html files. (ZIP) [file pone.0098330.s008.zip › figures/198.png]

RegionID: 198, chr1:9129487-9129791-M\_values

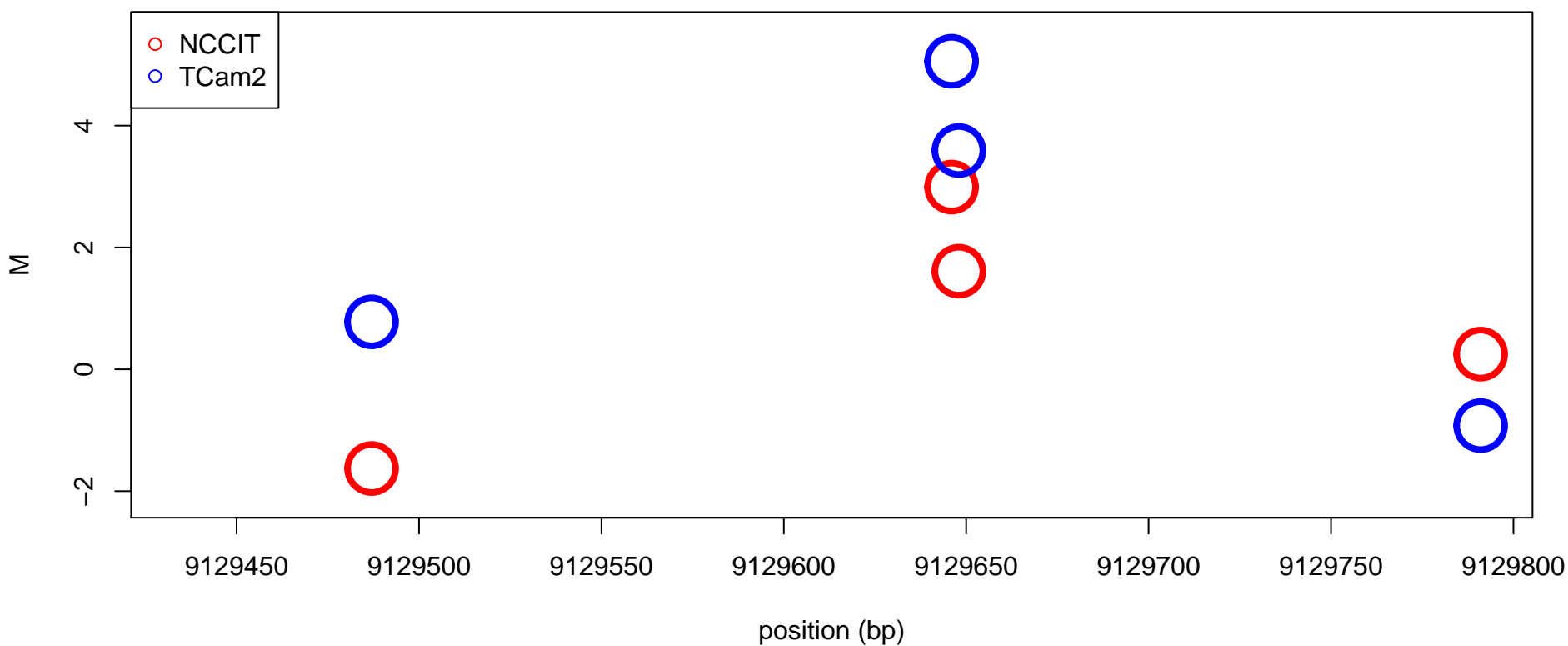

RegionID: 198, chr1:9129487-9129791-Beta\_values

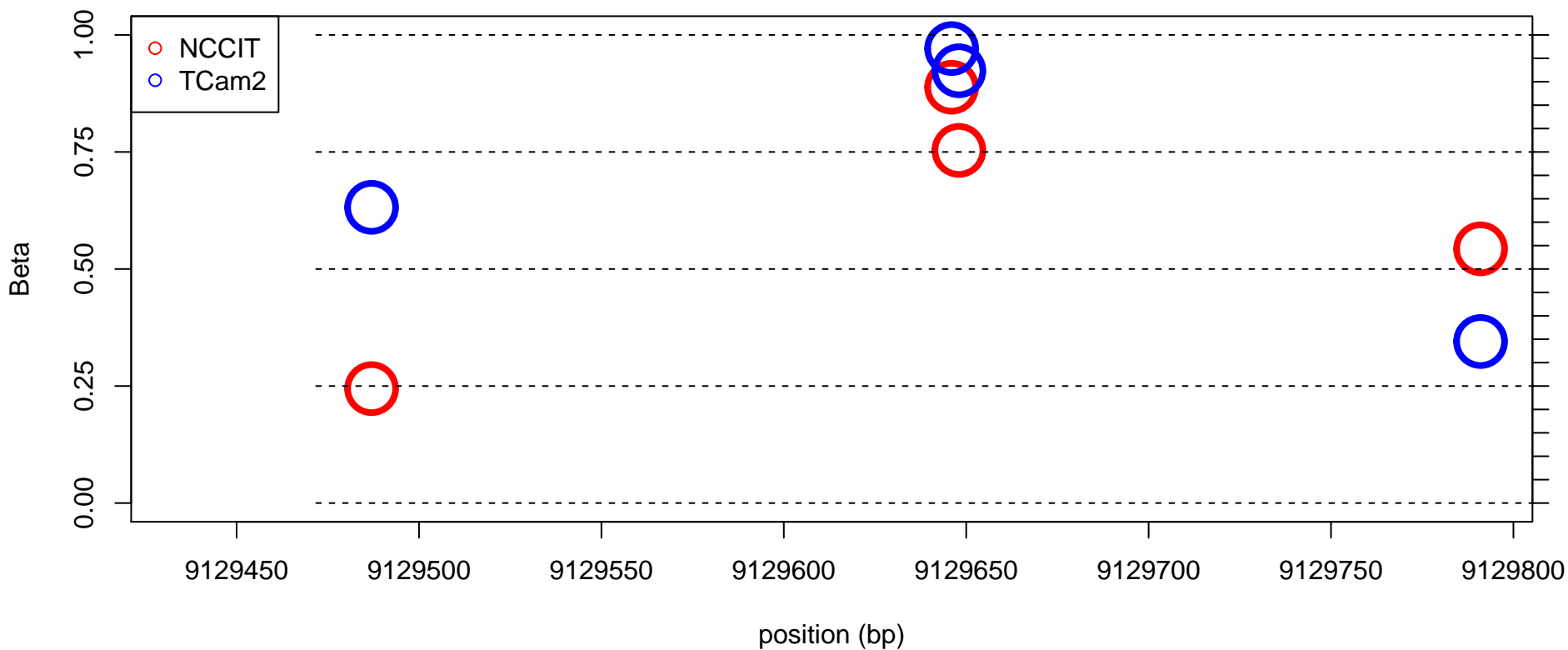

Supplement: File S1 — ZIP file containing DMRforPairs output for significant regions. Please start from the html files. (ZIP) [file pone.0098330.s008.zip › figures/198.pdf]

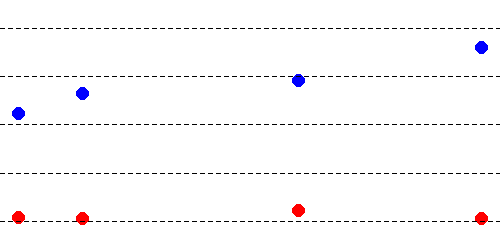

Supplement: File S1 — ZIP file containing DMRforPairs output for significant regions. Please start from the html files. (ZIP) [file pone.0098330.s008.zip › figures/225.png]

RegionID: 225, chr1:11752109–11752494–M\_values

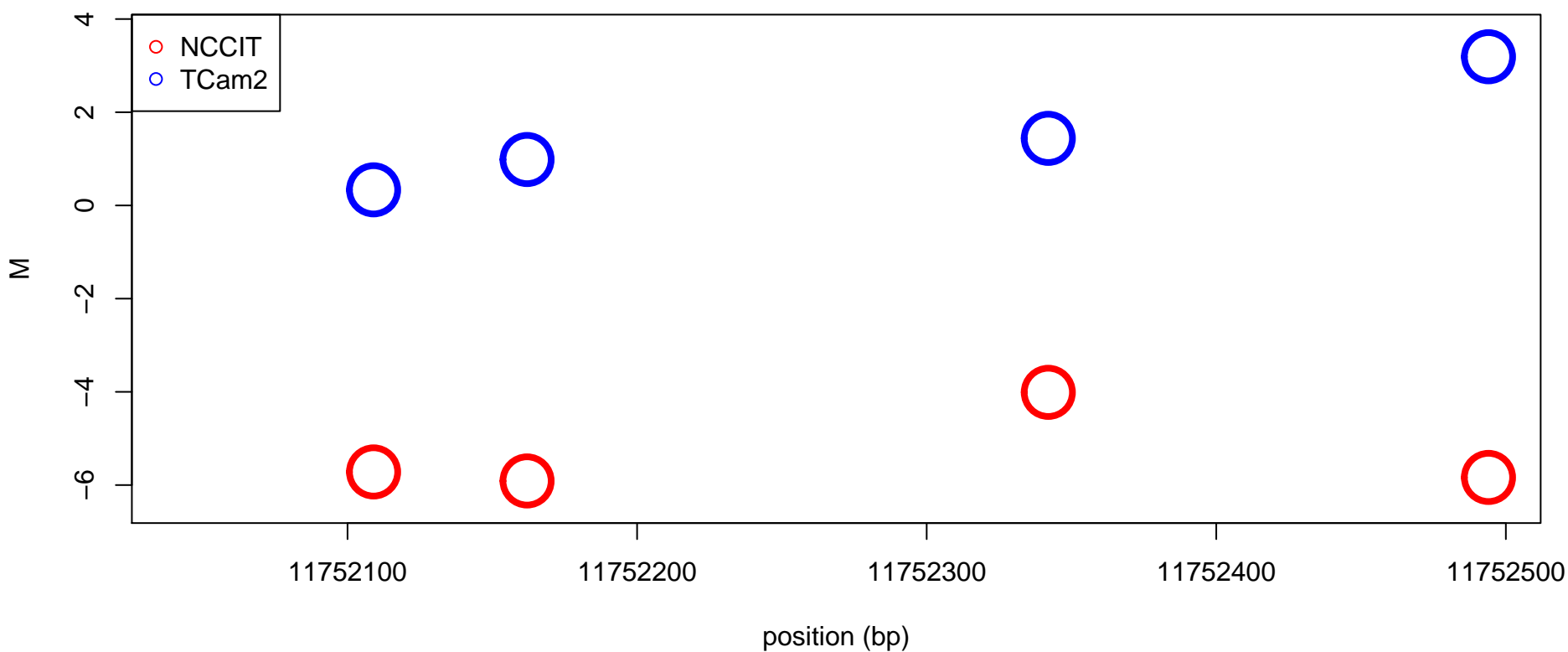

RegionID: 225, chr1:11752109–11752494–Beta\_values

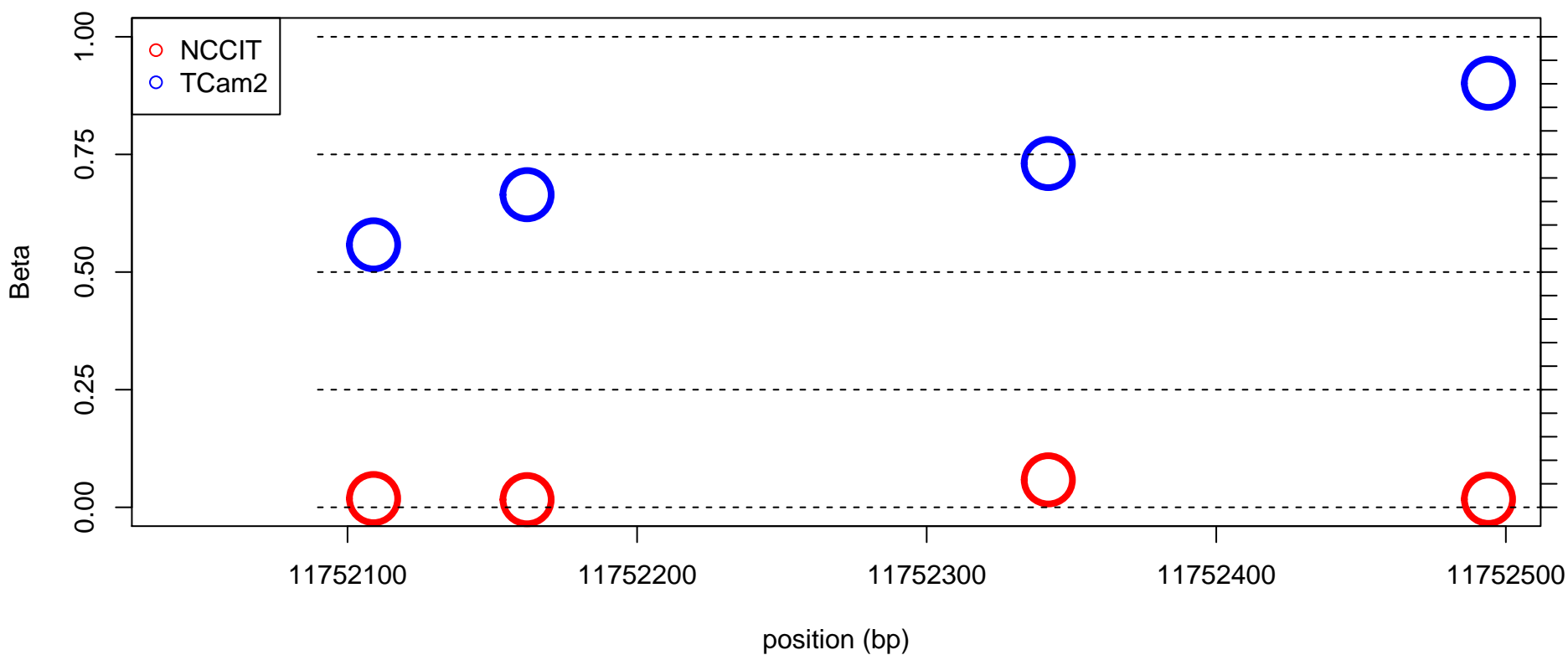

Supplement: File S1 — ZIP file containing DMRforPairs output for significant regions. Please start from the html files. (ZIP) [file pone.0098330.s008.zip › figures/225.pdf]

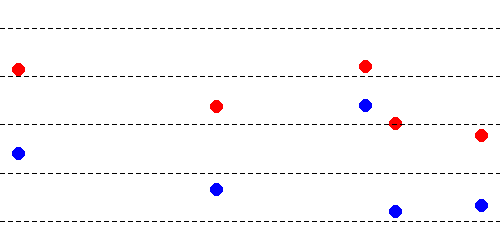

Supplement: File S1 — ZIP file containing DMRforPairs output for significant regions. Please start from the html files. (ZIP) [file pone.0098330.s008.zip › figures/227.png]

RegionID: 227, chr1:11898405–11898805–M\_values

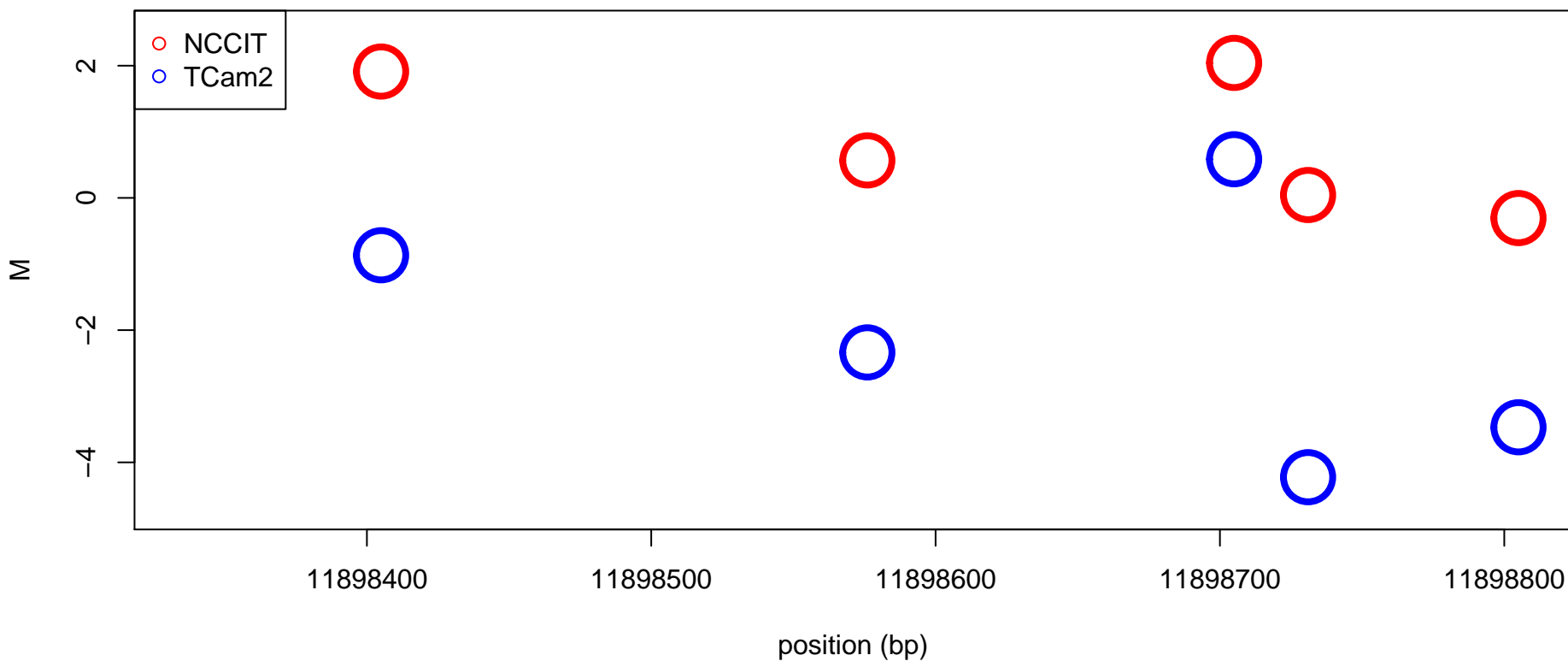

RegionID: 227, chr1:11898405–11898805–Beta\_values

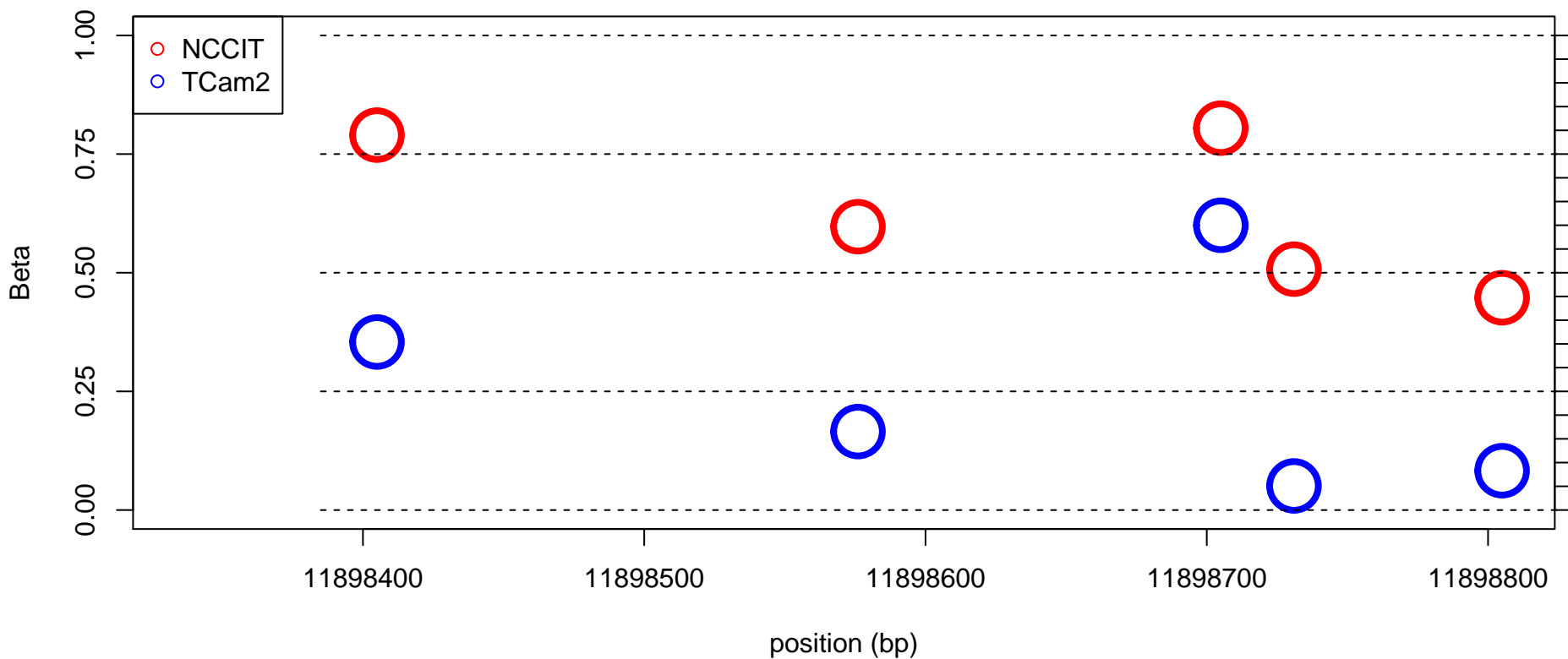

Supplement: File S1 — ZIP file containing DMRforPairs output for significant regions. Please start from the html files. (ZIP) [file pone.0098330.s008.zip › figures/227.pdf]

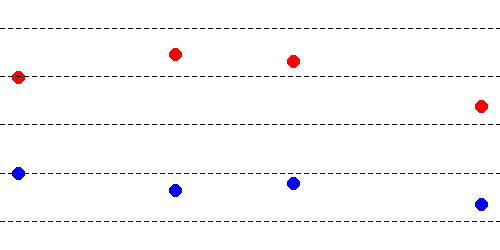

Supplement: File S1 — ZIP file containing DMRforPairs output for significant regions. Please start from the html files. (ZIP) [file pone.0098330.s008.zip › figures/232.png]

RegionID: 232, chr1:12538341–12538678–M\_values

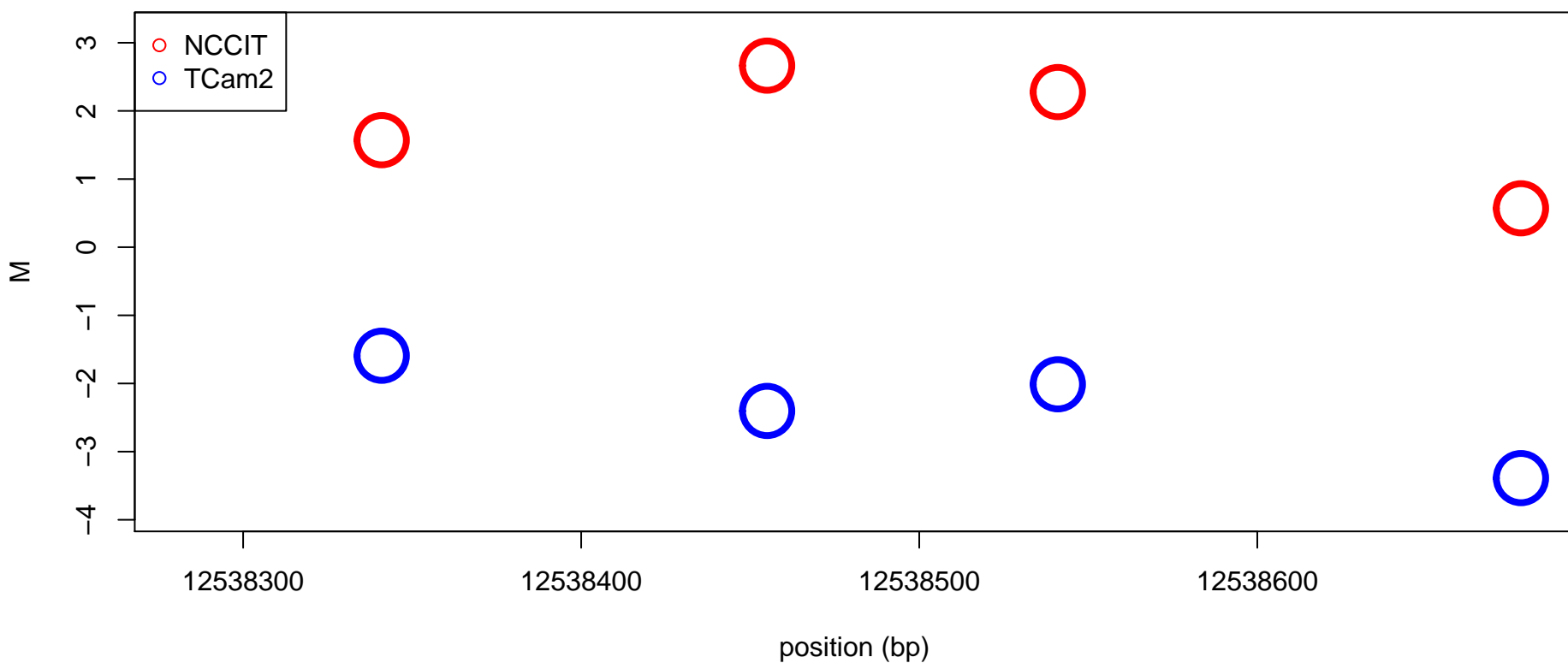

RegionID: 232, chr1:12538341–12538678–Beta\_values

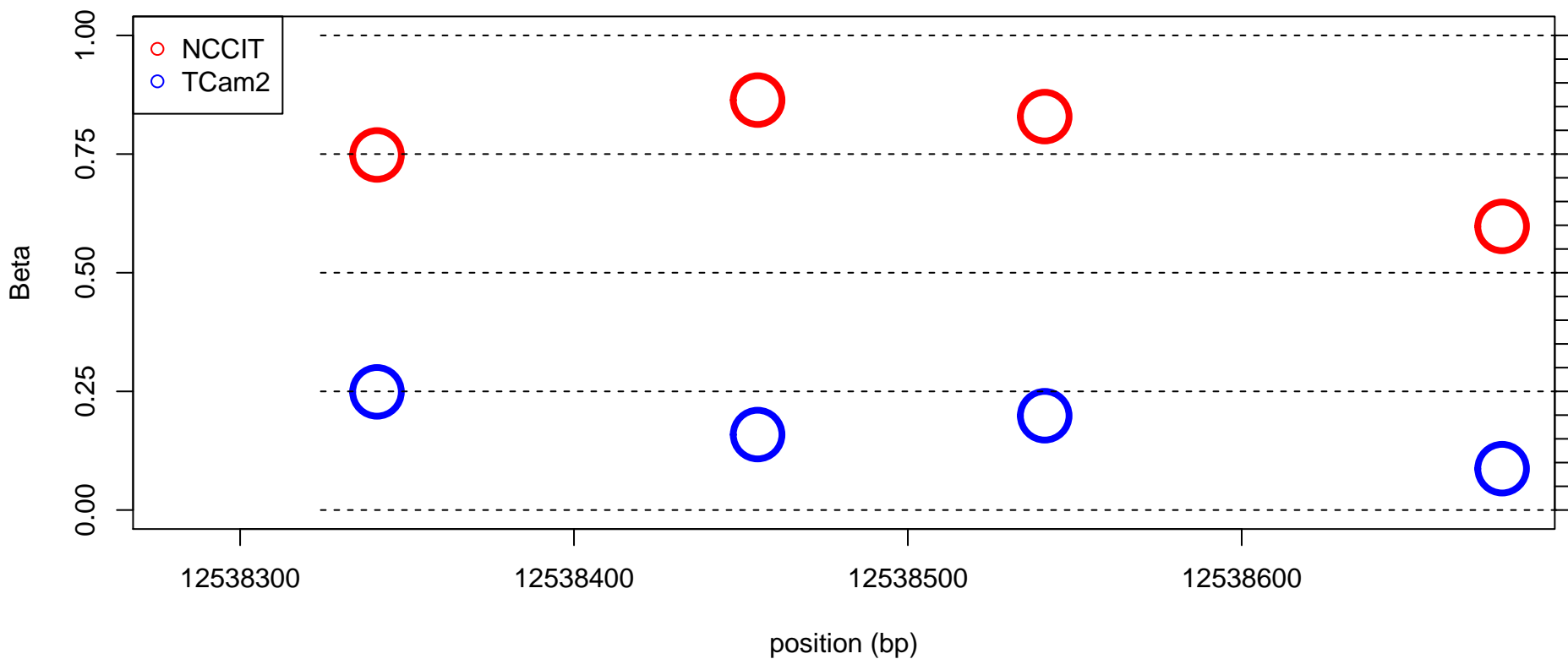

Supplement: File S1 — ZIP file containing DMRforPairs output for significant regions. Please start from the html files. (ZIP) [file pone.0098330.s008.zip › figures/232.pdf]

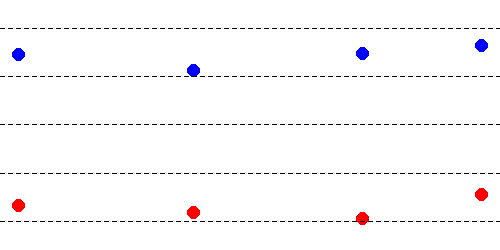

Supplement: File S1 — ZIP file containing DMRforPairs output for significant regions. Please start from the html files. (ZIP) [file pone.0098330.s008.zip › figures/233.png]

RegionID: 233, chr1:12655992–12656315–M\_values

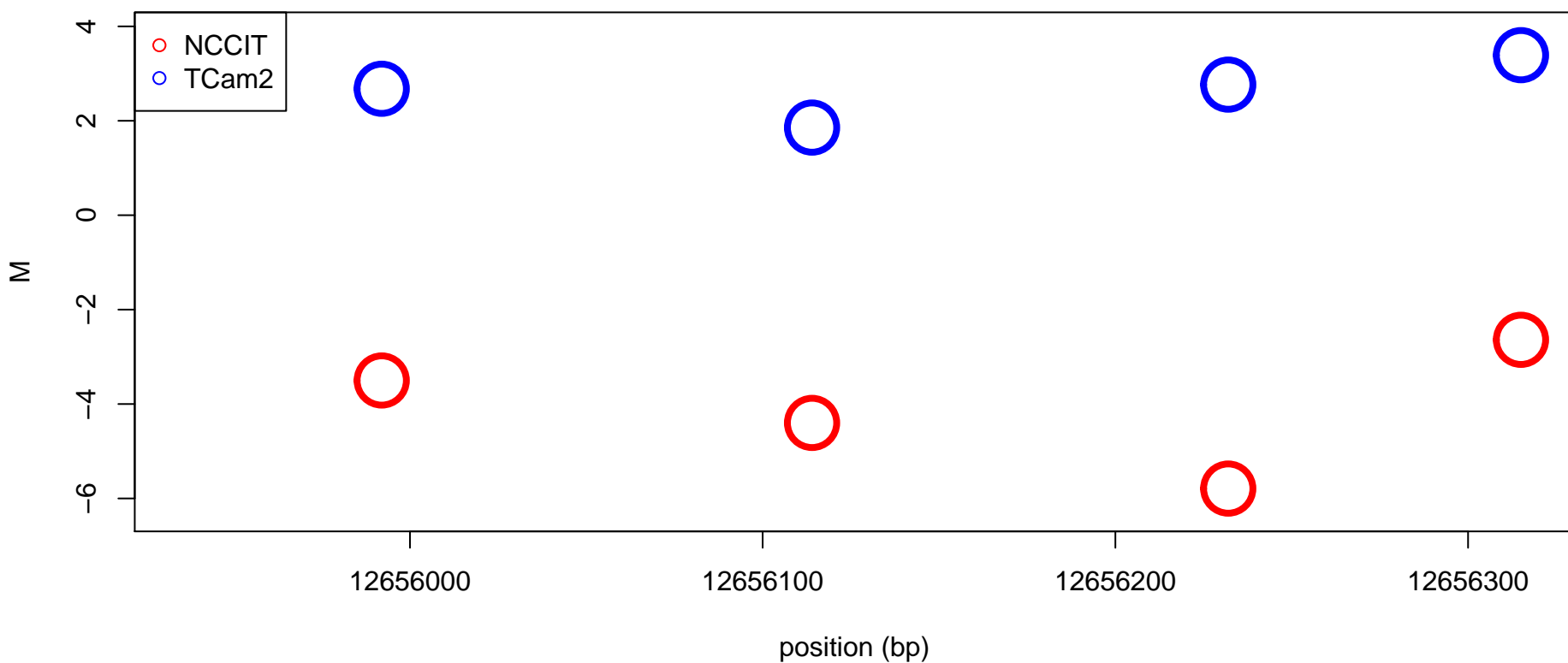

RegionID: 233, chr1:12655992–12656315–Beta\_values

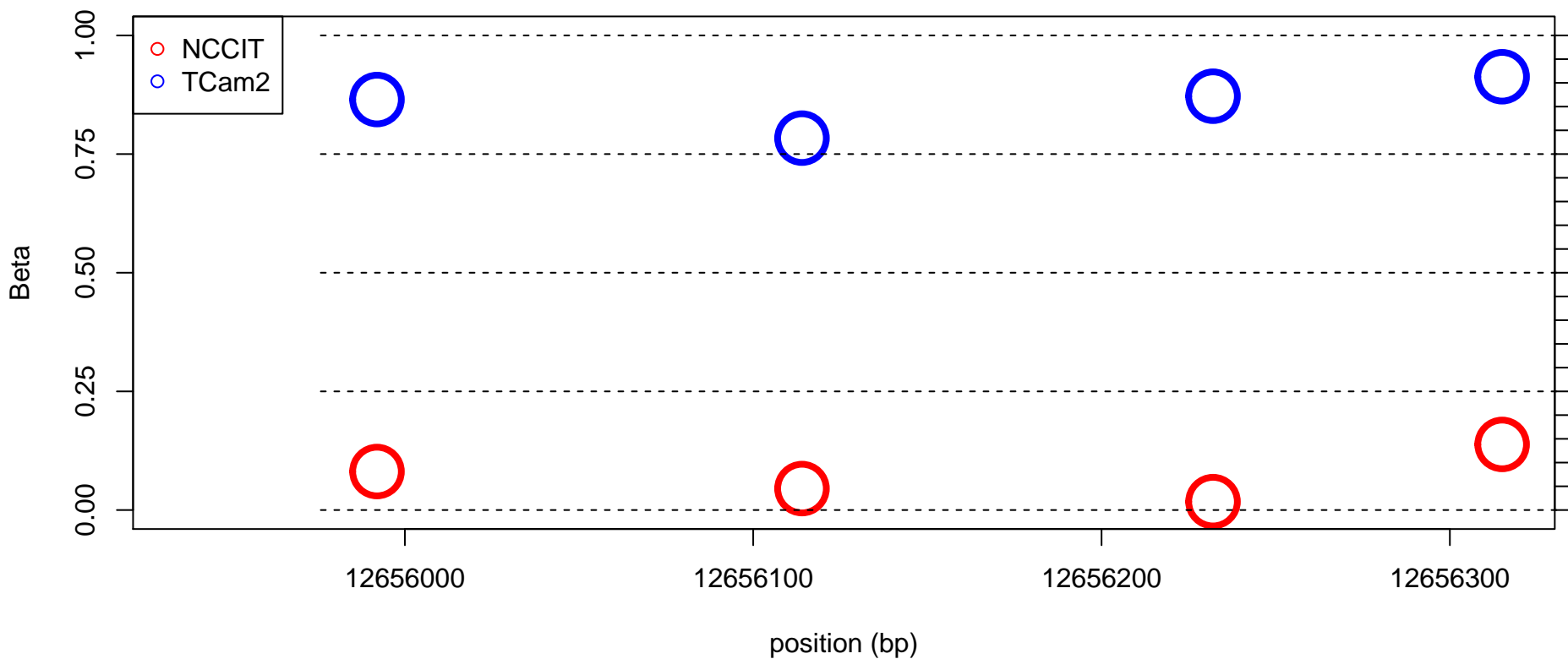

Supplement: File S1 — ZIP file containing DMRforPairs output for significant regions. Please start from the html files. (ZIP) [file pone.0098330.s008.zip › figures/233.pdf]

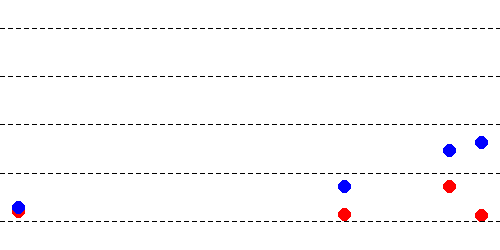

Supplement: File S1 — ZIP file containing DMRforPairs output for significant regions. Please start from the html files. (ZIP) [file pone.0098330.s008.zip › figures/235.png]

RegionID: 235, chr1:14027378–14027581–M\_values

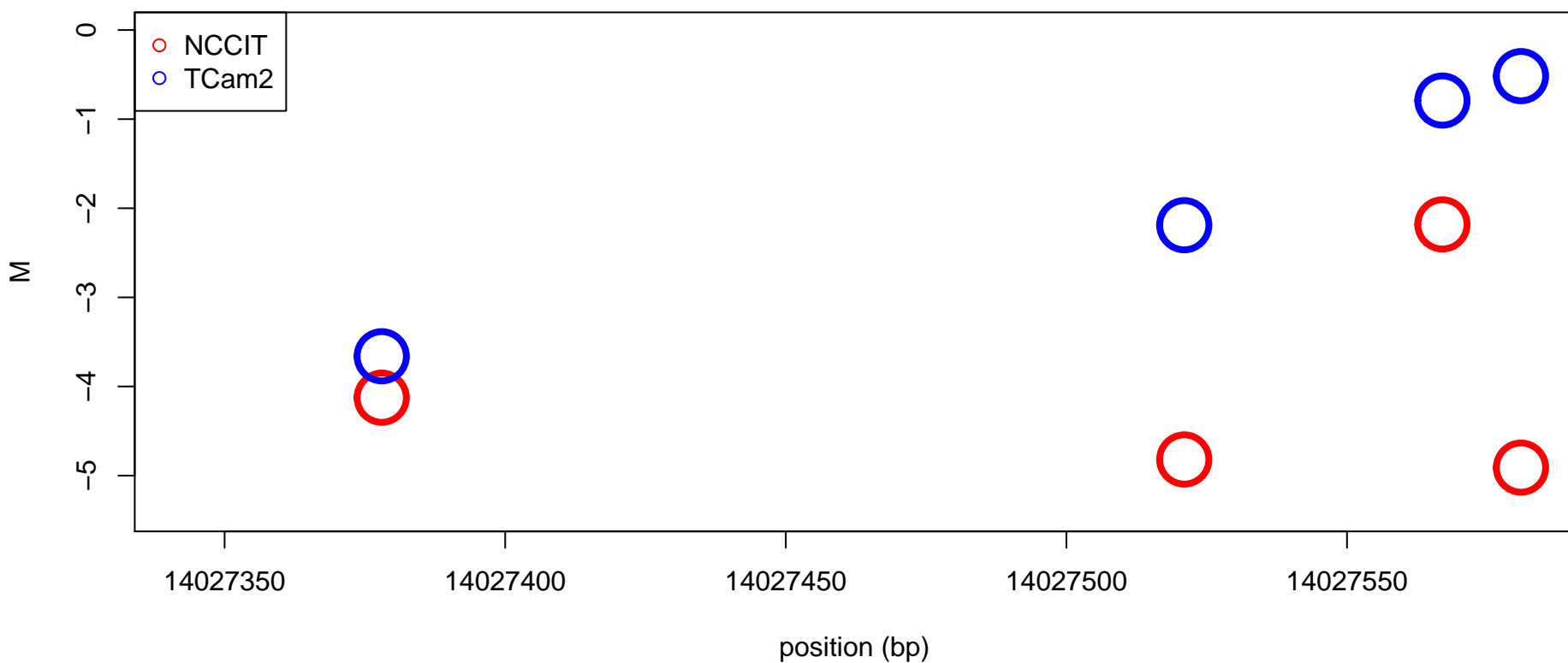

RegionID: 235, chr1:14027378–14027581–Beta\_values

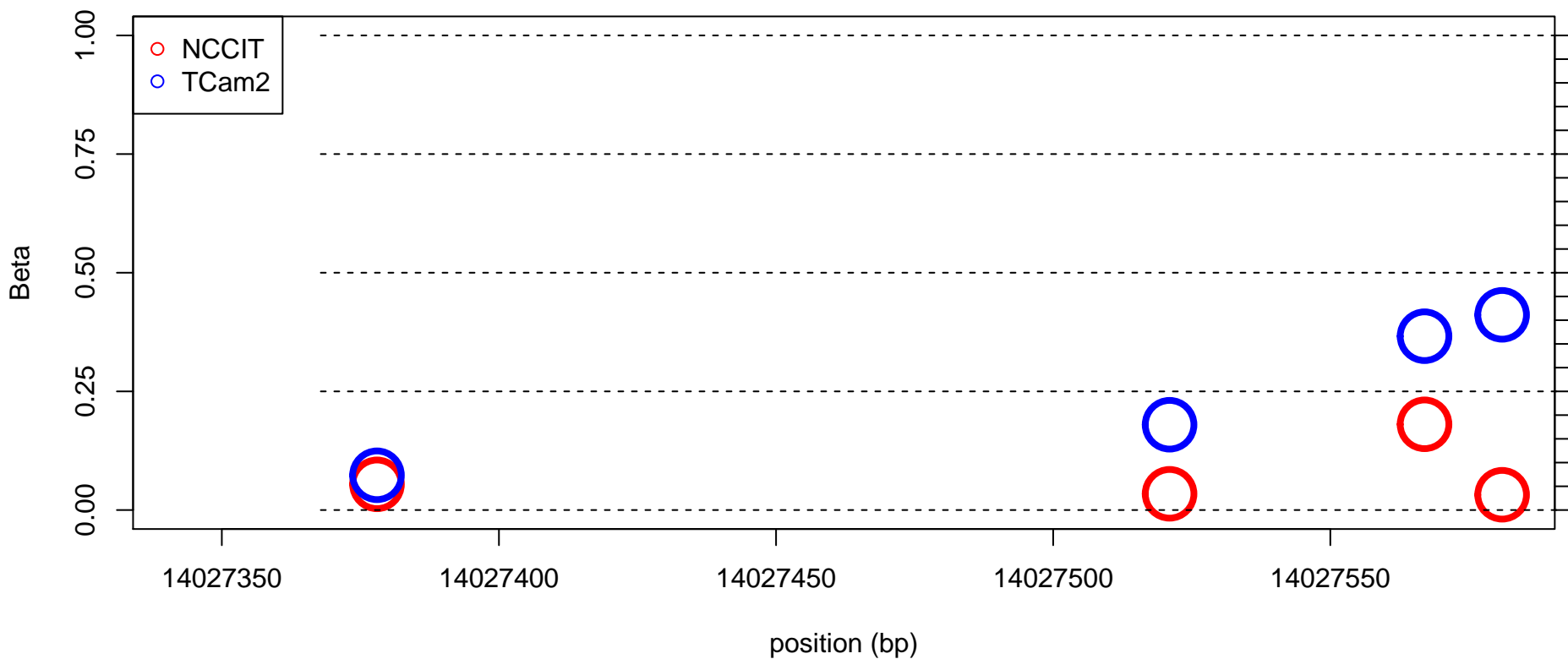

Supplement: File S1 — ZIP file containing DMRforPairs output for significant regions. Please start from the html files. (ZIP) [file pone.0098330.s008.zip › figures/235.pdf]

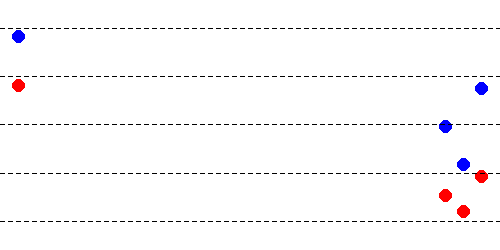

Supplement: File S1 — ZIP file containing DMRforPairs output for significant regions. Please start from the html files. (ZIP) [file pone.0098330.s008.zip › figures/238.png]

RegionID: 238, chr1:15250209–15250388–M\_values

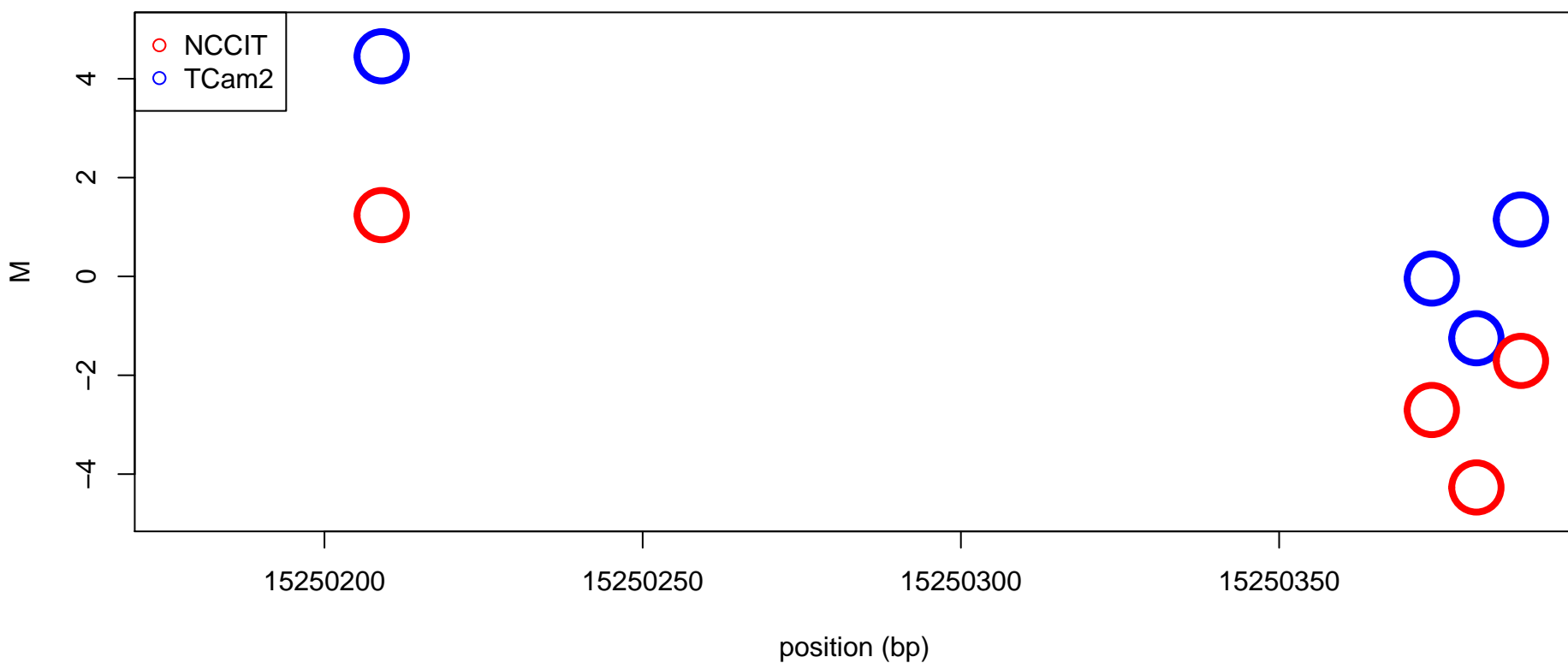

RegionID: 238, chr1:15250209–15250388–Beta\_values

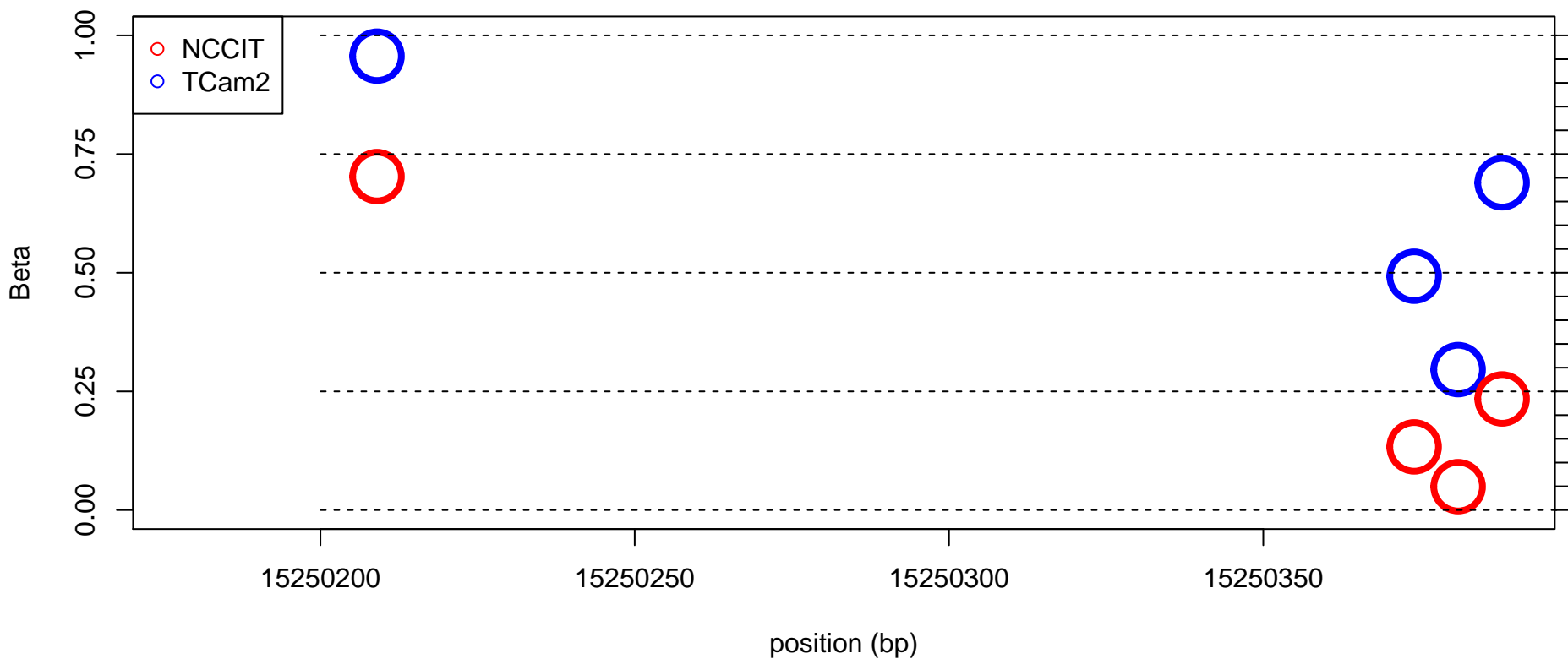

Supplement: File S1 — ZIP file containing DMRforPairs output for significant regions. Please start from the html files. (ZIP) [file pone.0098330.s008.zip › figures/238.pdf]

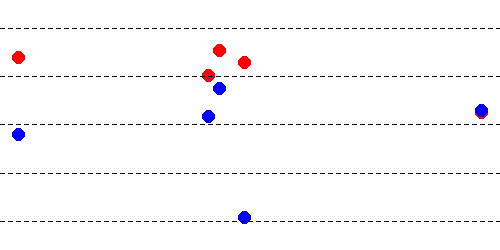

Supplement: File S1 — ZIP file containing DMRforPairs output for significant regions. Please start from the html files. (ZIP) [file pone.0098330.s008.zip › figures/239.png]

RegionID: 239, chr1:15256054–15256222–M\_values

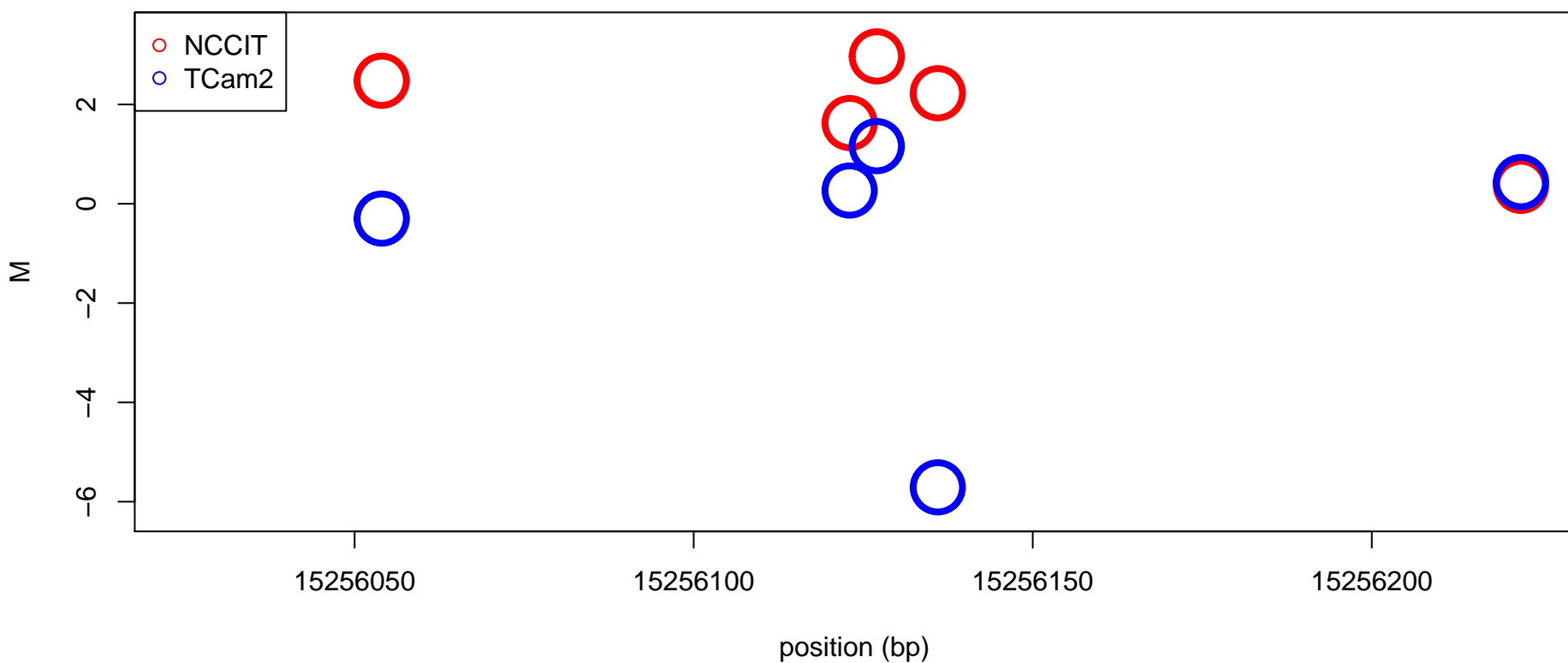

RegionID: 239, chr1:15256054–15256222–Beta\_values

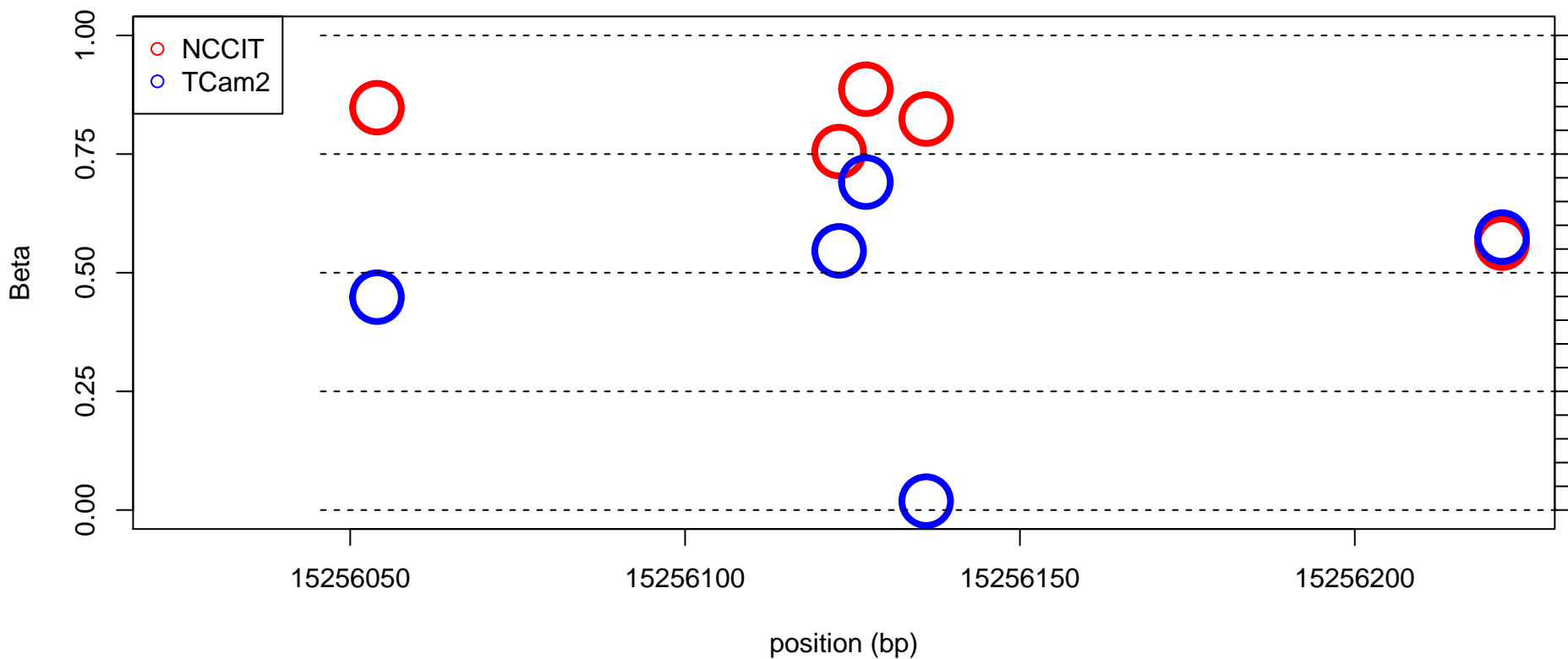

Supplement: File S1 — ZIP file containing DMRforPairs output for significant regions. Please start from the html files. (ZIP) [file pone.0098330.s008.zip › figures/239.pdf]

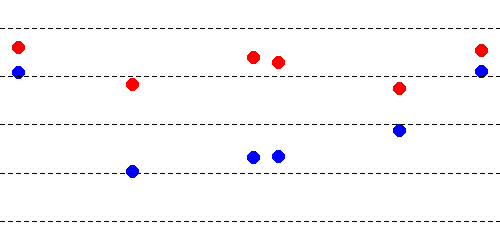

Supplement: File S1 — ZIP file containing DMRforPairs output for significant regions. Please start from the html files. (ZIP) [file pone.0098330.s008.zip › figures/240.png]

RegionID: 240, chr1:15271830–15272326–M\_values

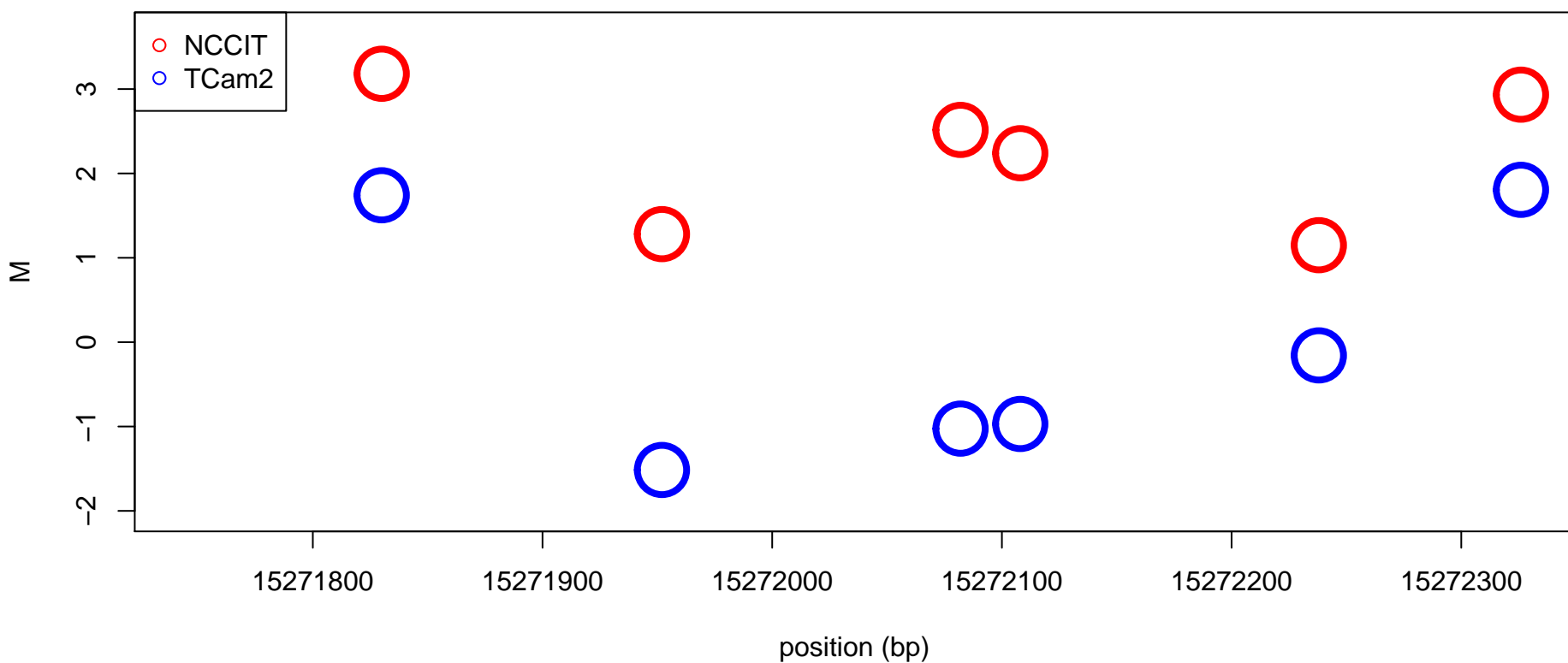

RegionID: 240, chr1:15271830–15272326–Beta\_values

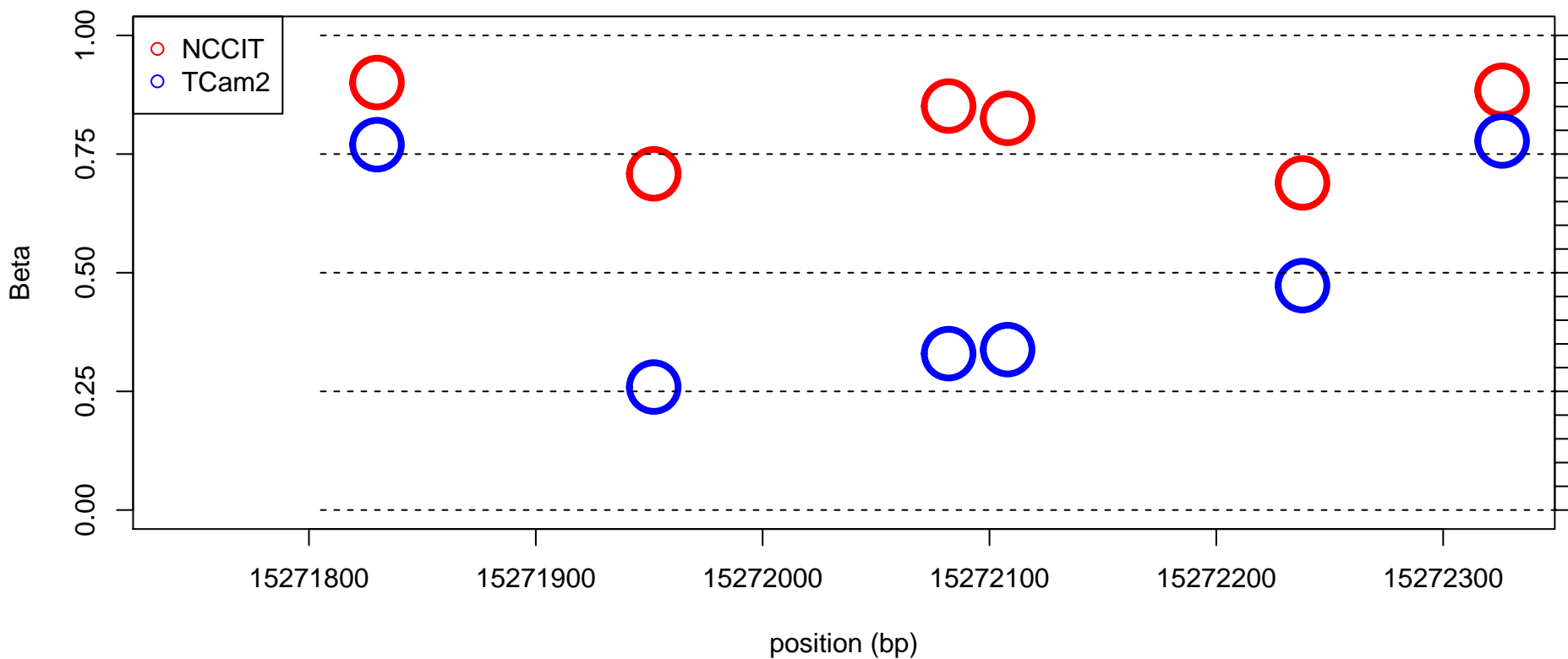

Supplement: File S1 — ZIP file containing DMRforPairs output for significant regions. Please start from the html files. (ZIP) [file pone.0098330.s008.zip › figures/240.pdf]

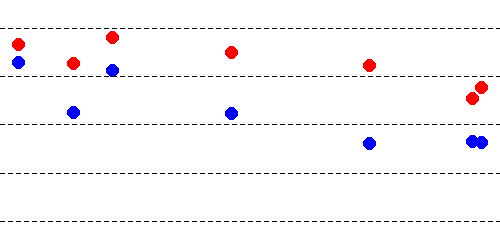

Supplement: File S1 — ZIP file containing DMRforPairs output for significant regions. Please start from the html files. (ZIP) [file pone.0098330.s008.zip › figures/251.png]

RegionID: 251, chr1:16163479–16164122–M\_values

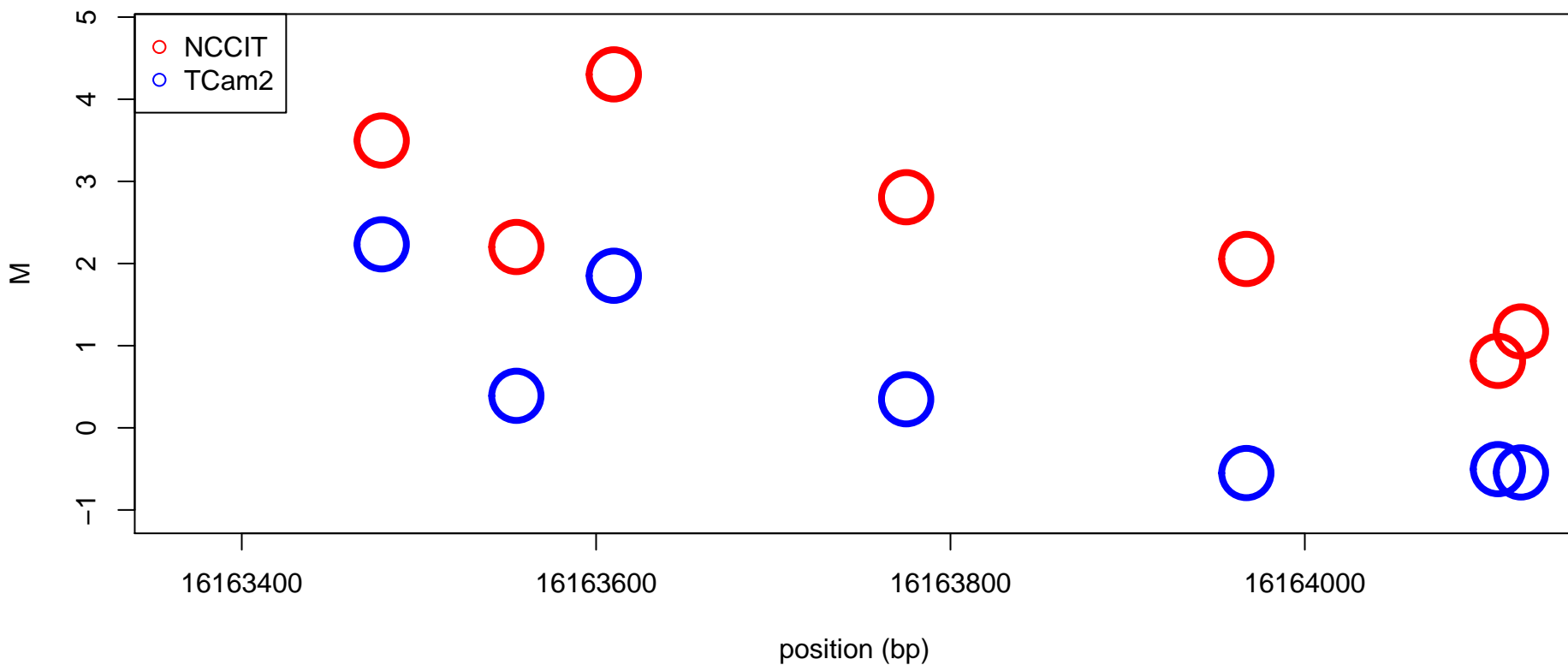

RegionID: 251, chr1:16163479–16164122–Beta\_values

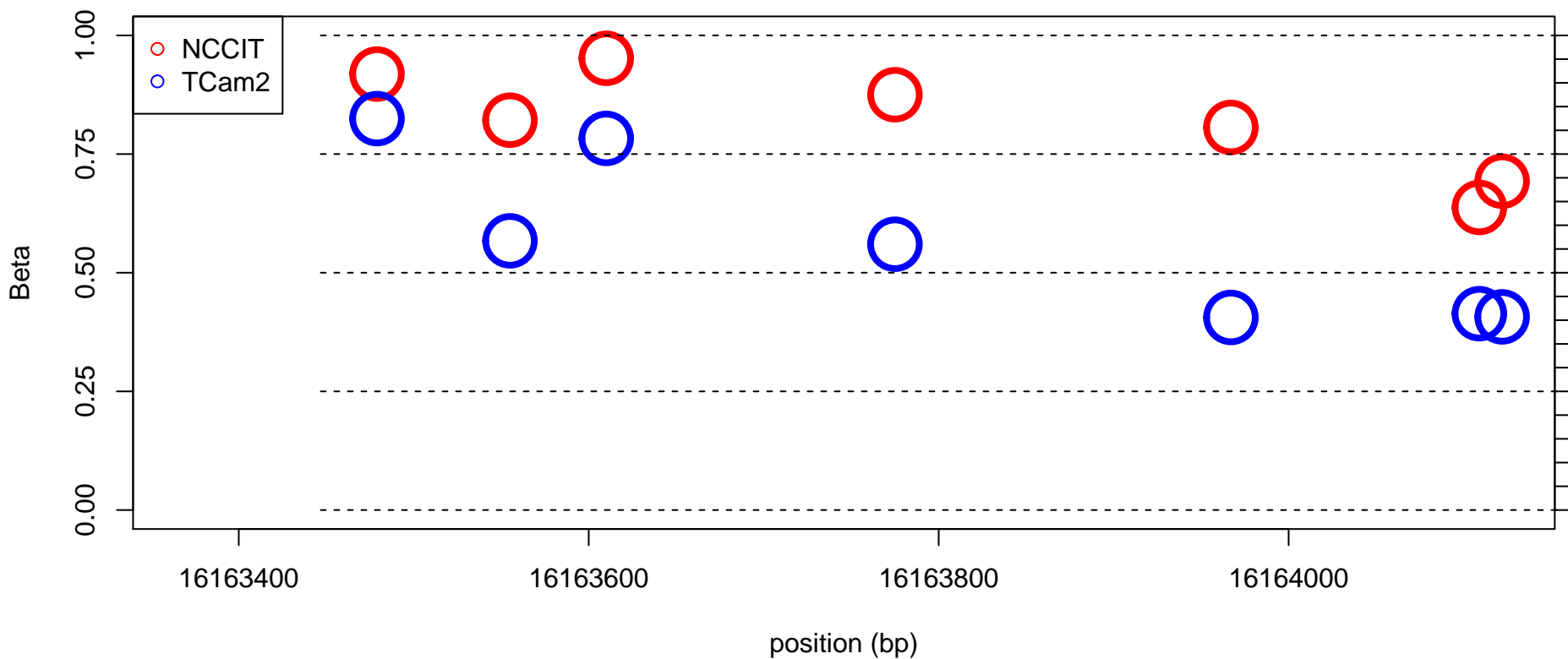

Supplement: File S1 — ZIP file containing DMRforPairs output for significant regions. Please start from the html files. (ZIP) [file pone.0098330.s008.zip › figures/251.pdf]

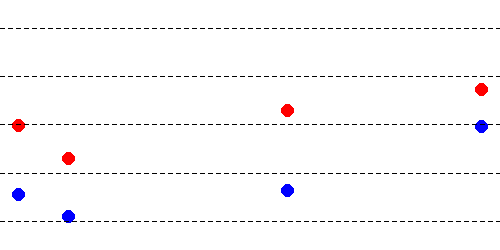

Supplement: File S1 — ZIP file containing DMRforPairs output for significant regions. Please start from the html files. (ZIP) [file pone.0098330.s008.zip › figures/260.png]

RegionID: 260, chr1:17026786–17027073–M\_values

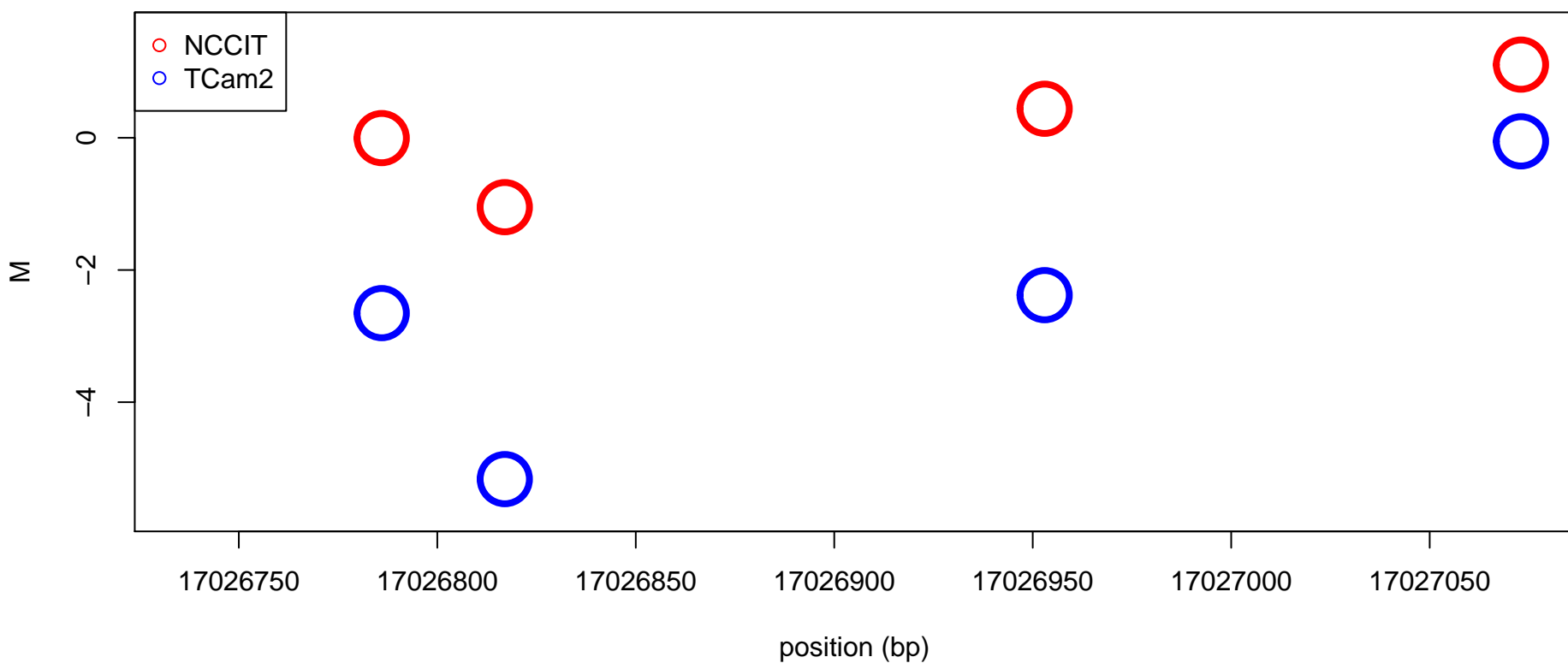

RegionID: 260, chr1:17026786–17027073–Beta\_values

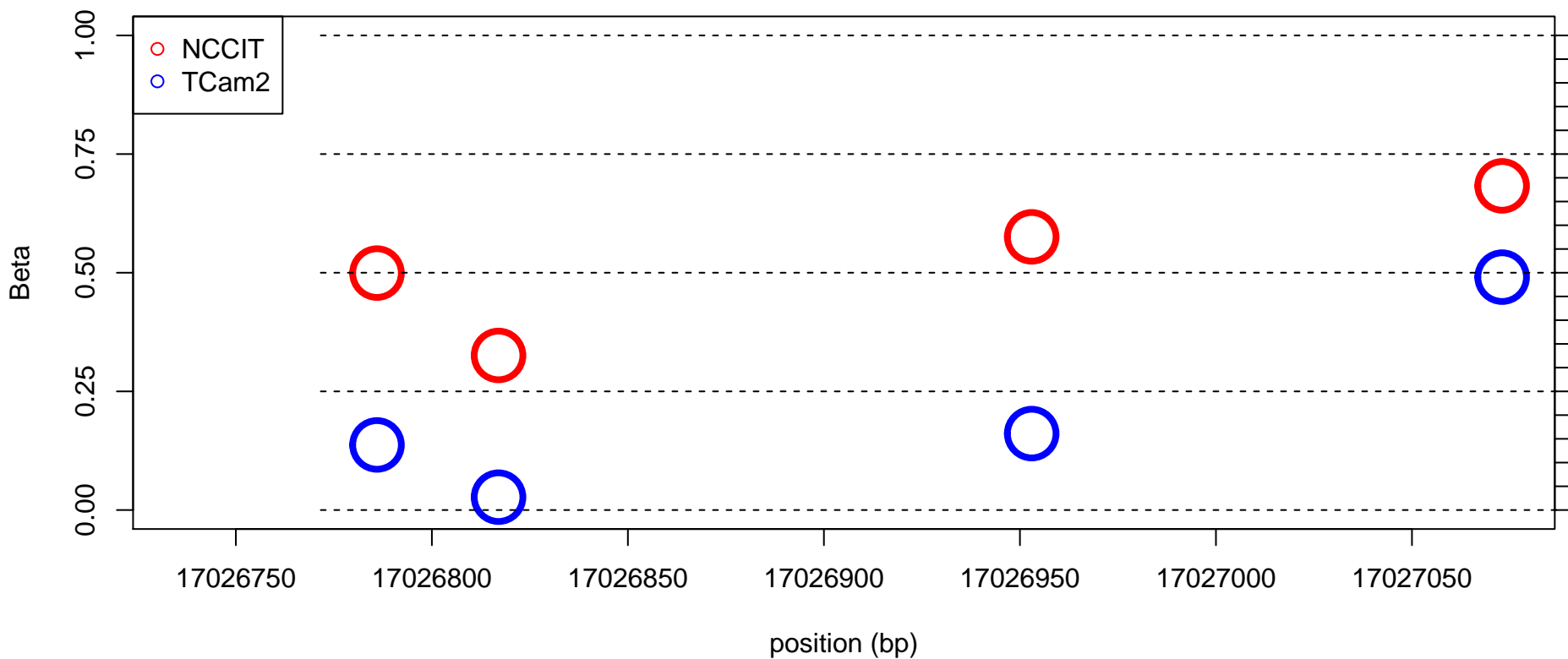

Supplement: File S1 — ZIP file containing DMRforPairs output for significant regions. Please start from the html files. (ZIP) [file pone.0098330.s008.zip › figures/260.pdf]

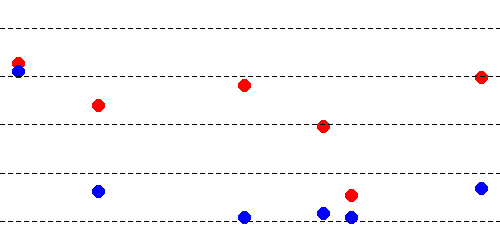

Supplement: File S1 — ZIP file containing DMRforPairs output for significant regions. Please start from the html files. (ZIP) [file pone.0098330.s008.zip › figures/265.png]

RegionID: 265, chr1:17746286–17746597–M\_values

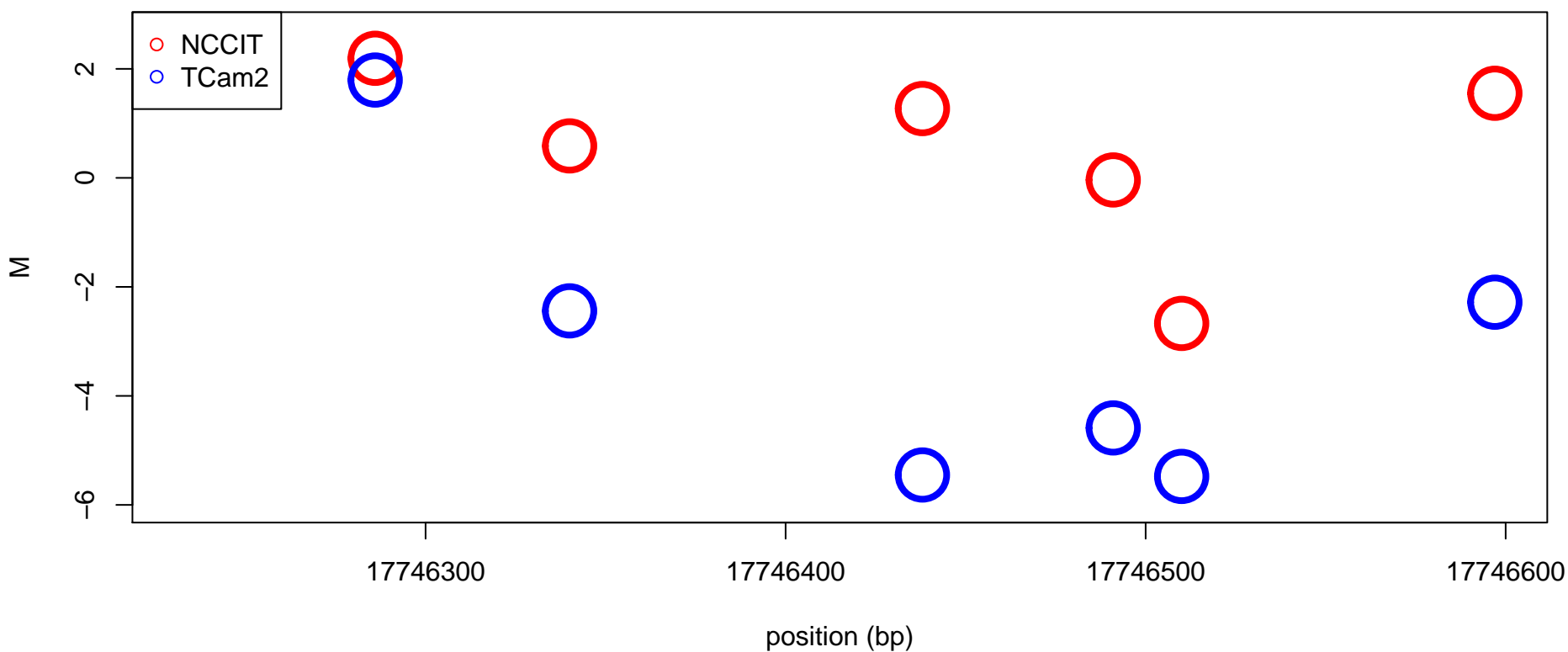

RegionID: 265, chr1:17746286–17746597–Beta\_values

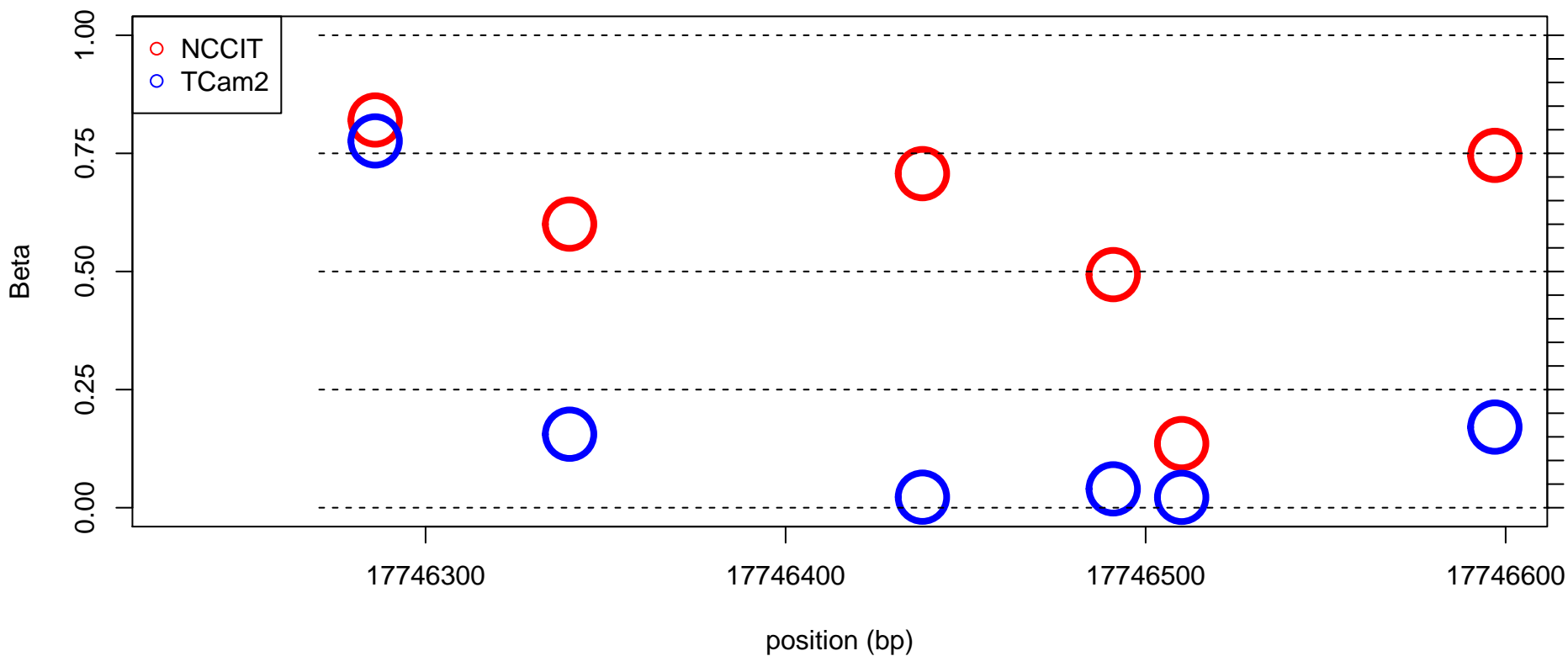

Supplement: File S1 — ZIP file containing DMRforPairs output for significant regions. Please start from the html files. (ZIP) [file pone.0098330.s008.zip › figures/265.pdf]

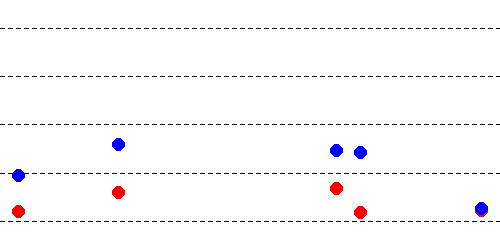

Supplement: File S1 — ZIP file containing DMRforPairs output for significant regions. Please start from the html files. (ZIP) [file pone.0098330.s008.zip › figures/274.png]

RegionID: 274, chr1:18972919–18973303–M\_values

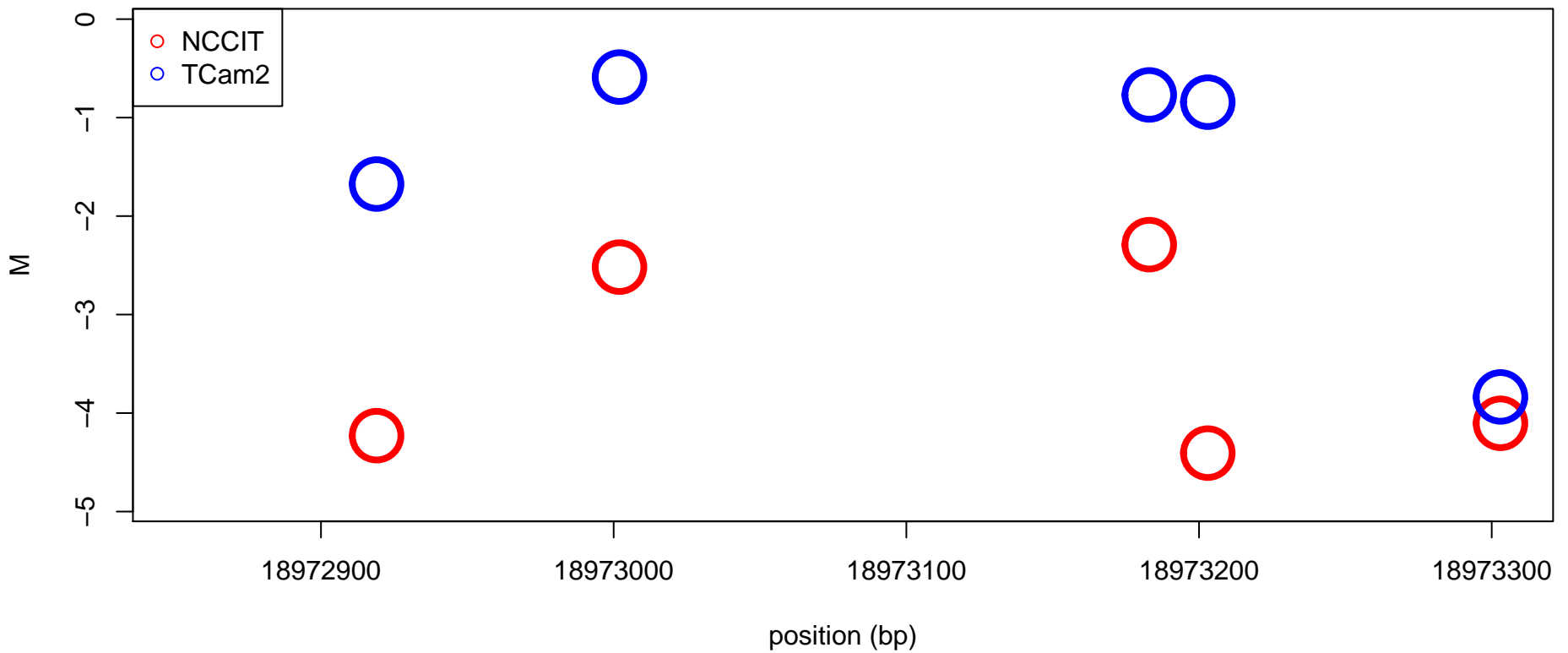

RegionID: 274, chr1:18972919–18973303–Beta\_values

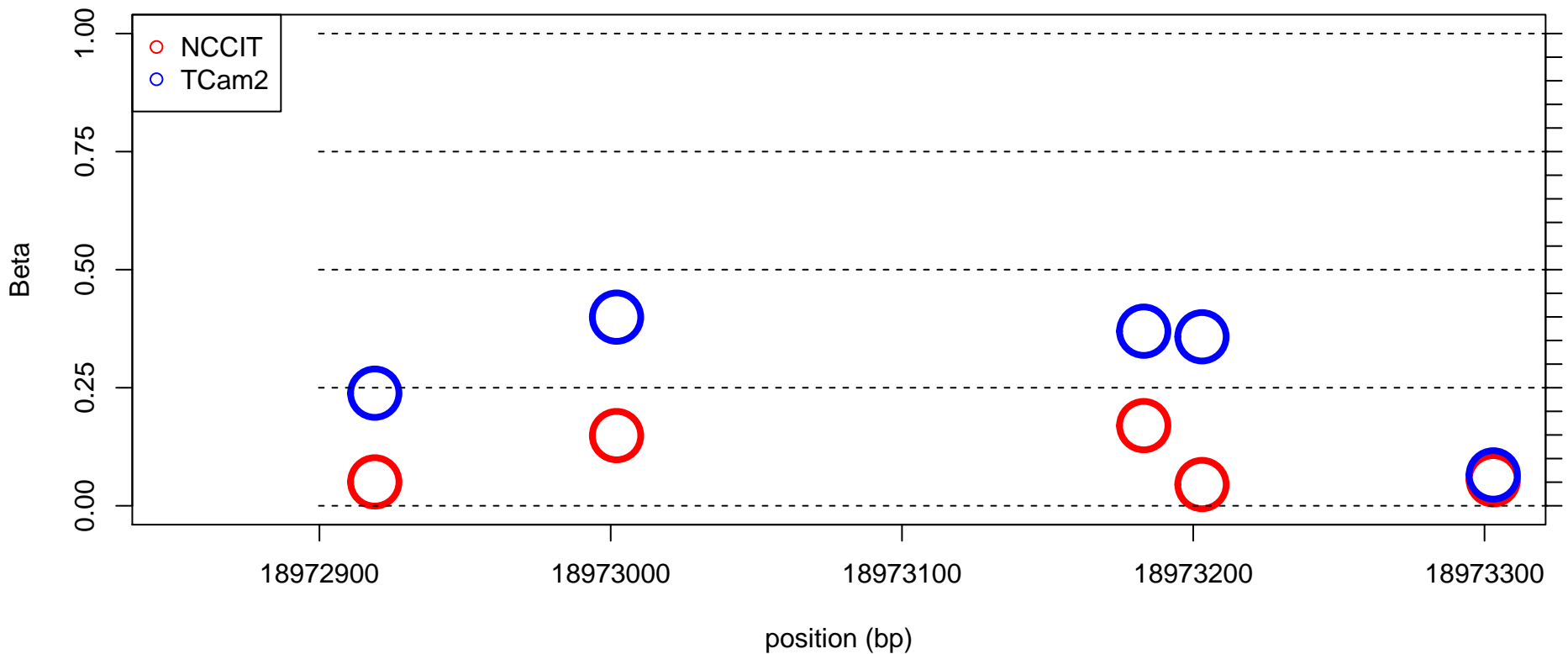

Supplement: File S1 — ZIP file containing DMRforPairs output for significant regions. Please start from the html files. (ZIP) [file pone.0098330.s008.zip › figures/274.pdf]

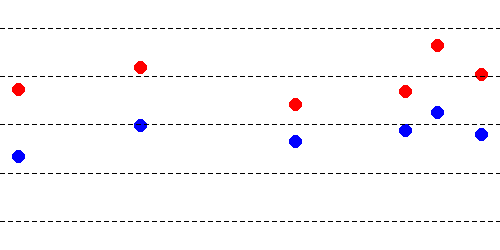

Supplement: File S1 — ZIP file containing DMRforPairs output for significant regions. Please start from the html files. (ZIP) [file pone.0098330.s008.zip › figures/275.png]

RegionID: 275, chr1:19181040–19181459–M\_values

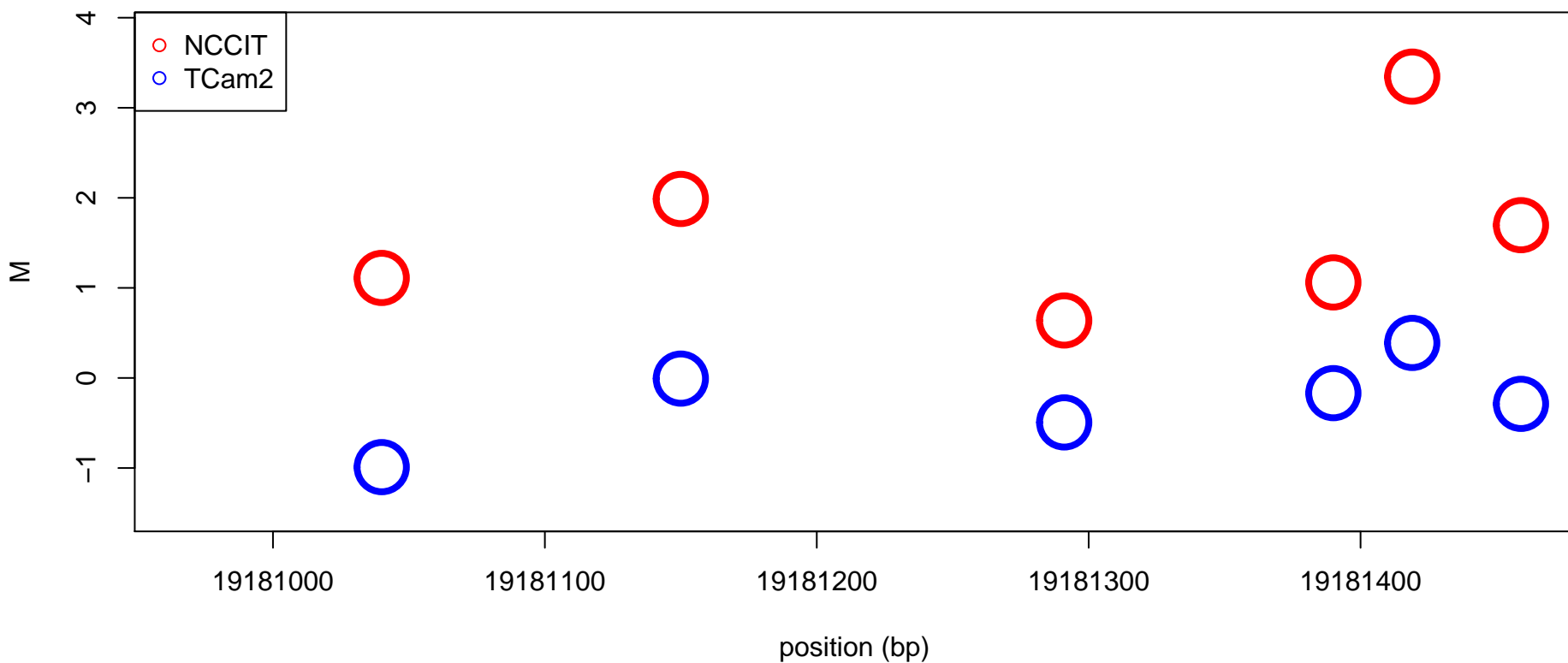

RegionID: 275, chr1:19181040–19181459–Beta\_values

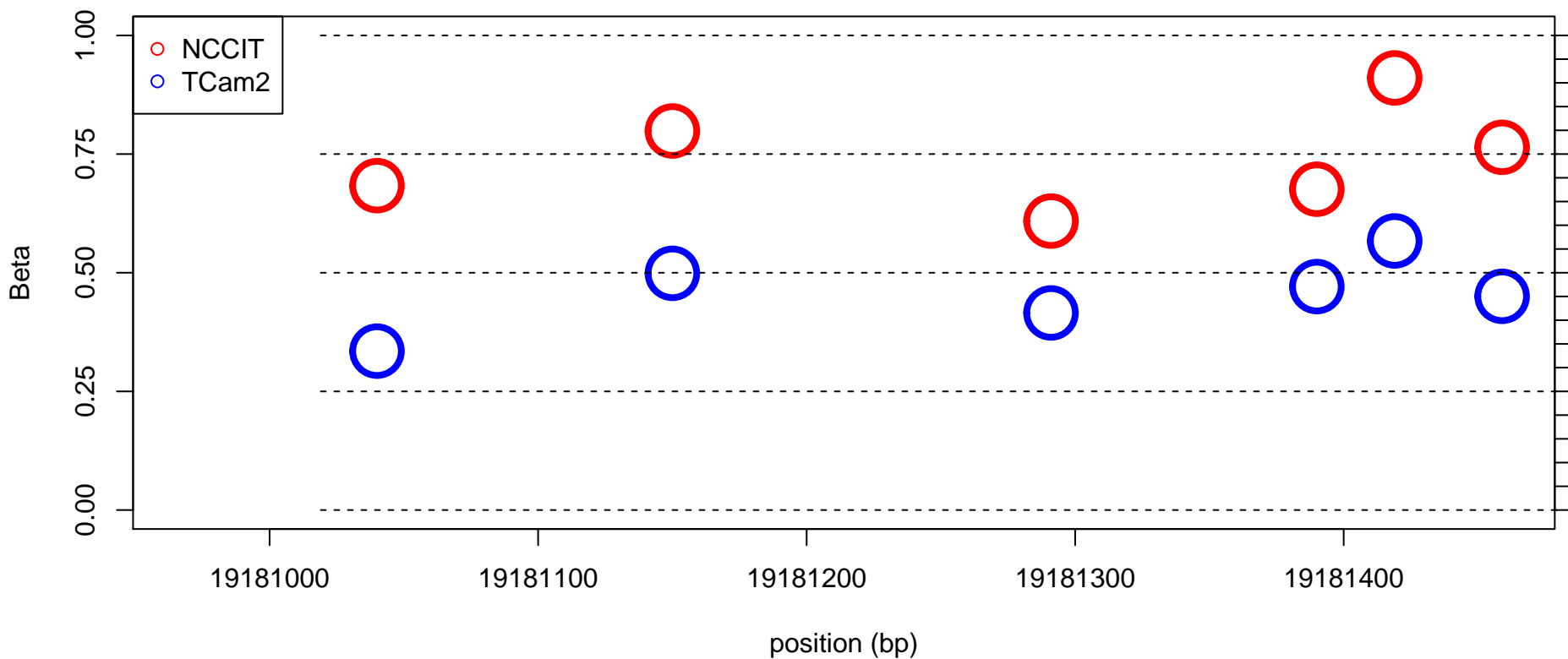

Supplement: File S1 — ZIP file containing DMRforPairs output for significant regions. Please start from the html files. (ZIP) [file pone.0098330.s008.zip › figures/275.pdf]

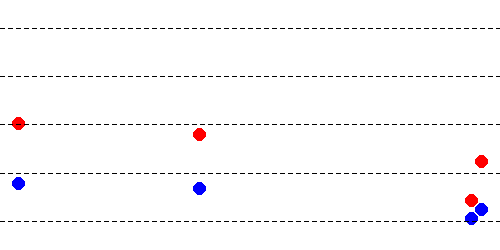

Supplement: File S1 — ZIP file containing DMRforPairs output for significant regions. Please start from the html files. (ZIP) [file pone.0098330.s008.zip › figures/283.png]

RegionID: 283, chr1:19970204–19970337–M\_values

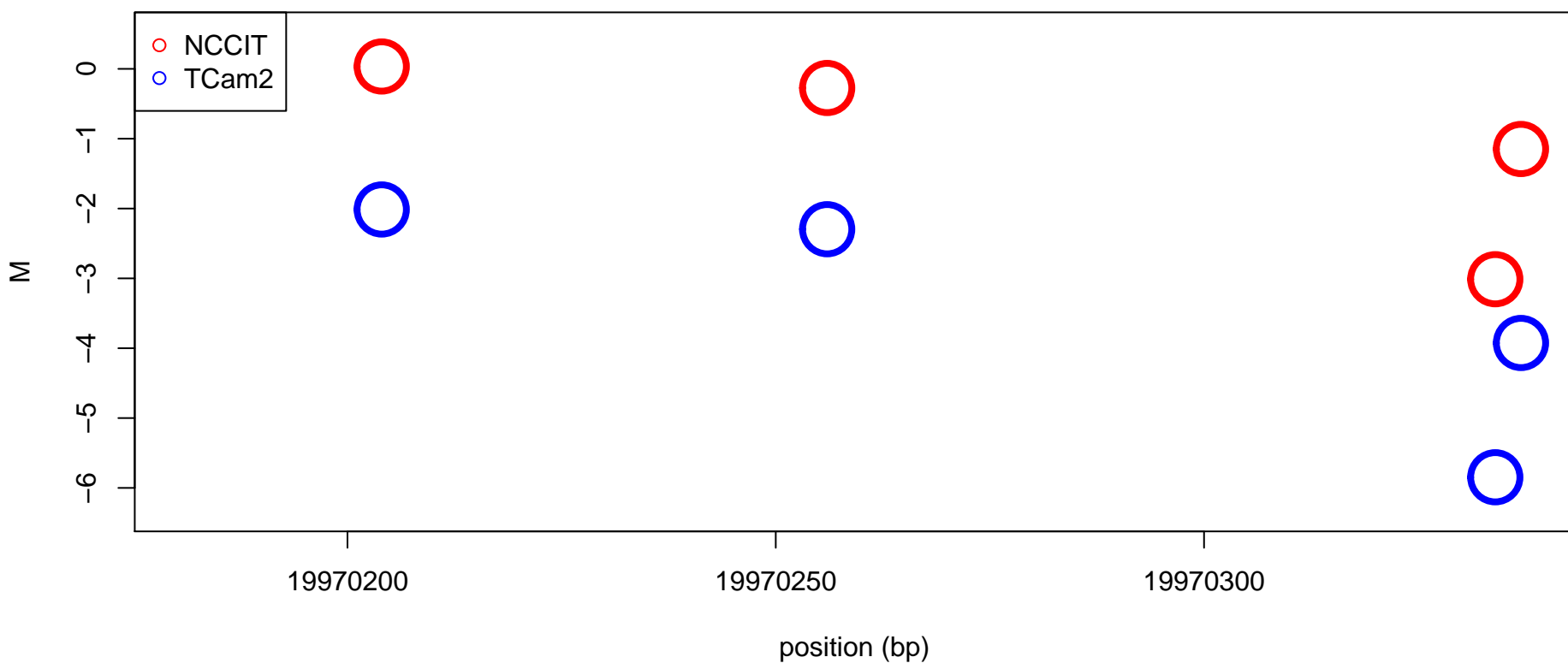

RegionID: 283, chr1:19970204–19970337–Beta\_values

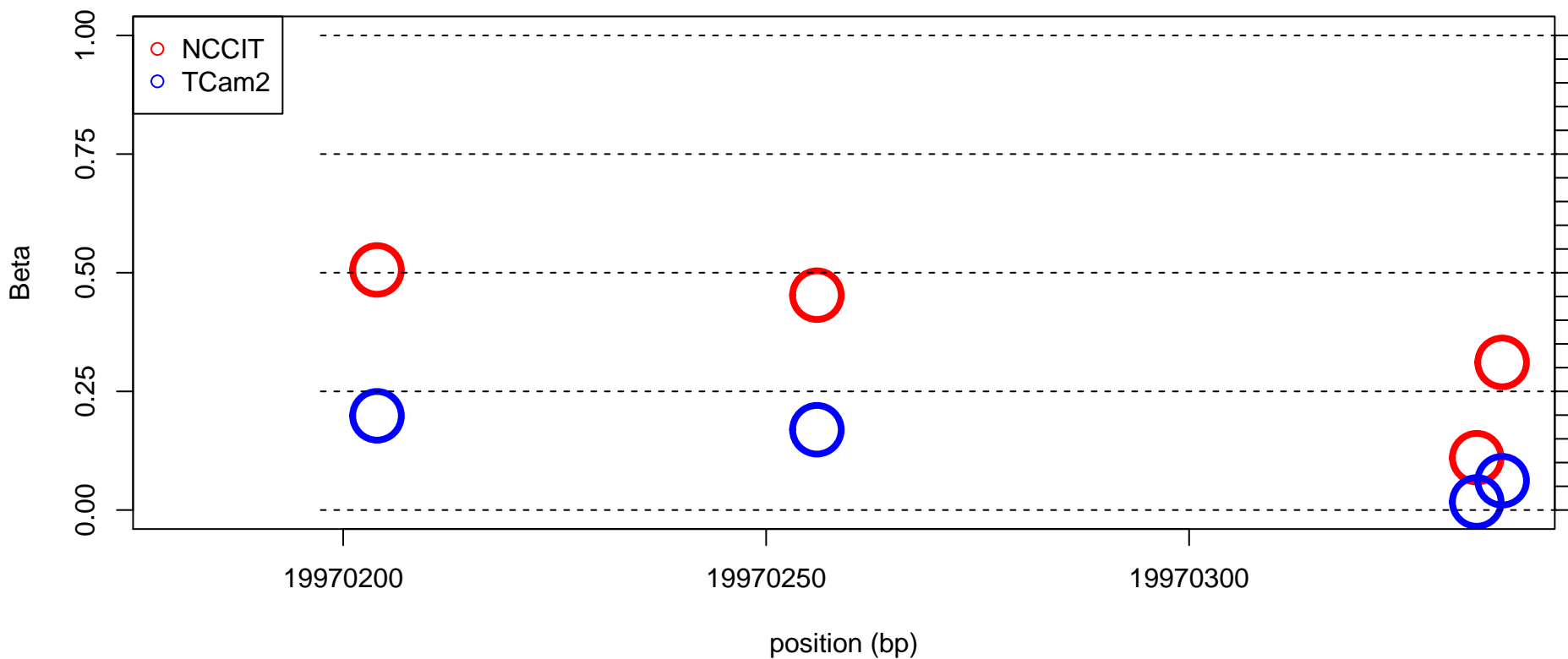

Supplement: File S1 — ZIP file containing DMRforPairs output for significant regions. Please start from the html files. (ZIP) [file pone.0098330.s008.zip › figures/283.pdf]

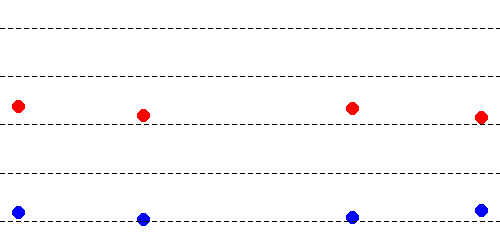

Supplement: File S1 — ZIP file containing DMRforPairs output for significant regions. Please start from the html files. (ZIP) [file pone.0098330.s008.zip › figures/286.png]

RegionID: 286, chr1:20669724–20669975–M\_values

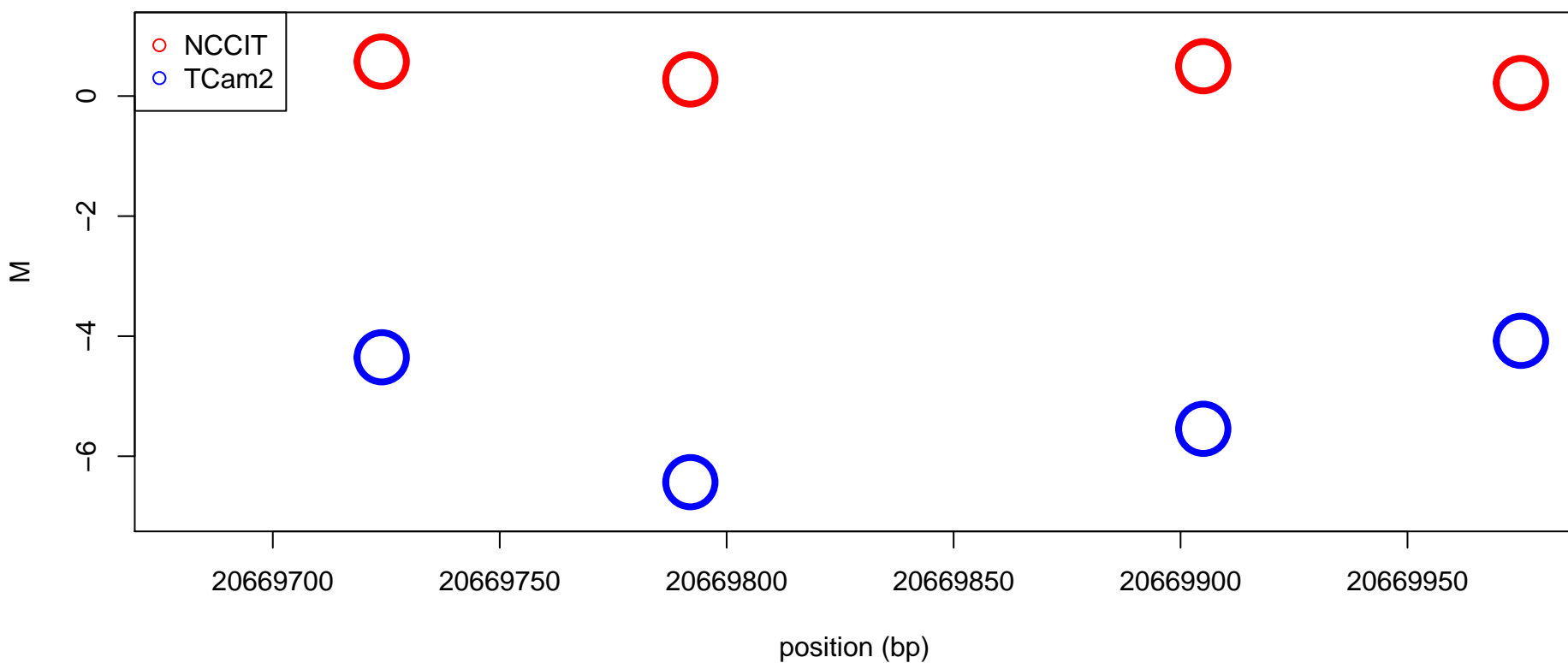

RegionID: 286, chr1:20669724–20669975–Beta\_values

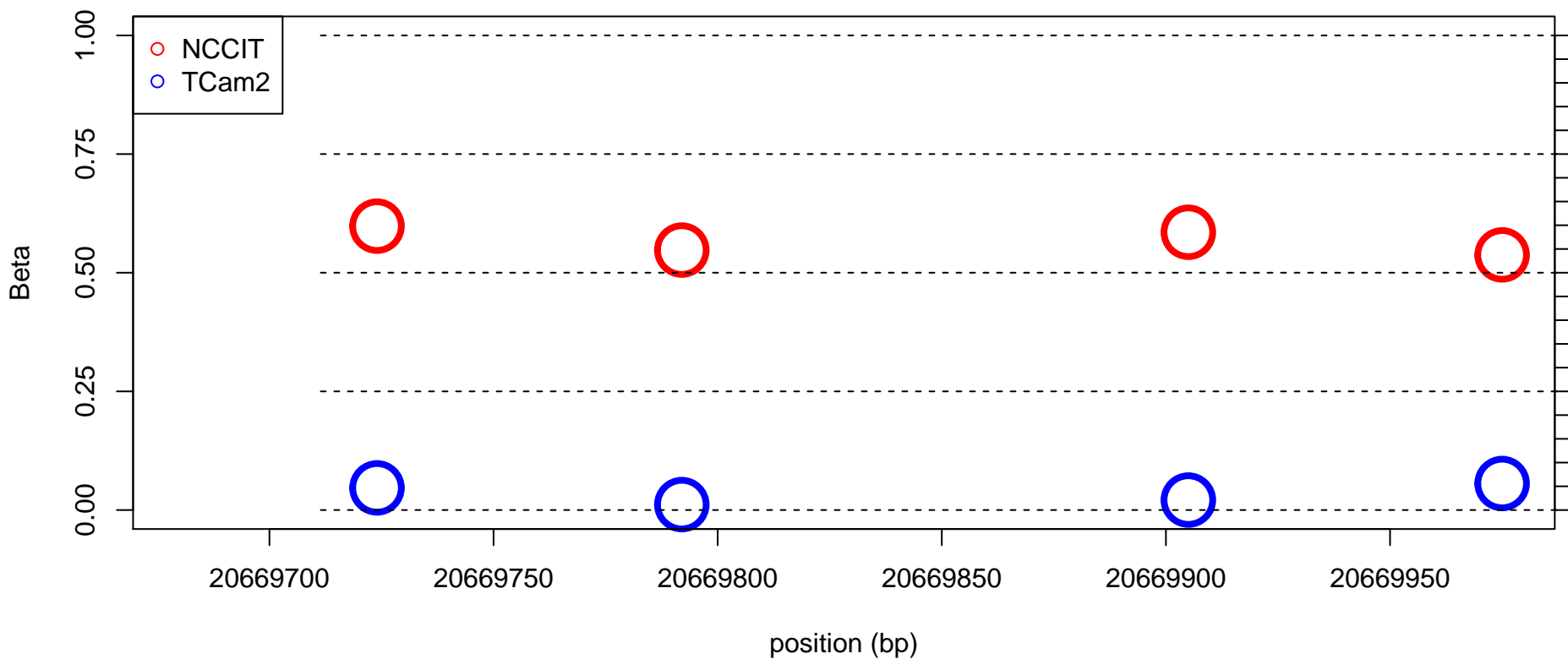

Supplement: File S1 — ZIP file containing DMRforPairs output for significant regions. Please start from the html files. (ZIP) [file pone.0098330.s008.zip › figures/286.pdf]

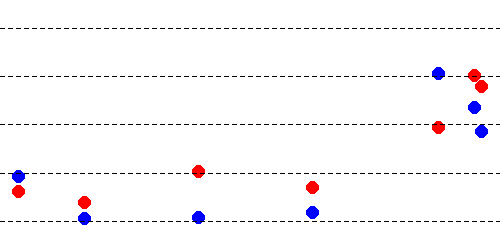

Supplement: File S1 — ZIP file containing DMRforPairs output for significant regions. Please start from the html files. (ZIP) [file pone.0098330.s008.zip › figures/300.png]

RegionID: 300, chr1:22140769–22141400–M\_values

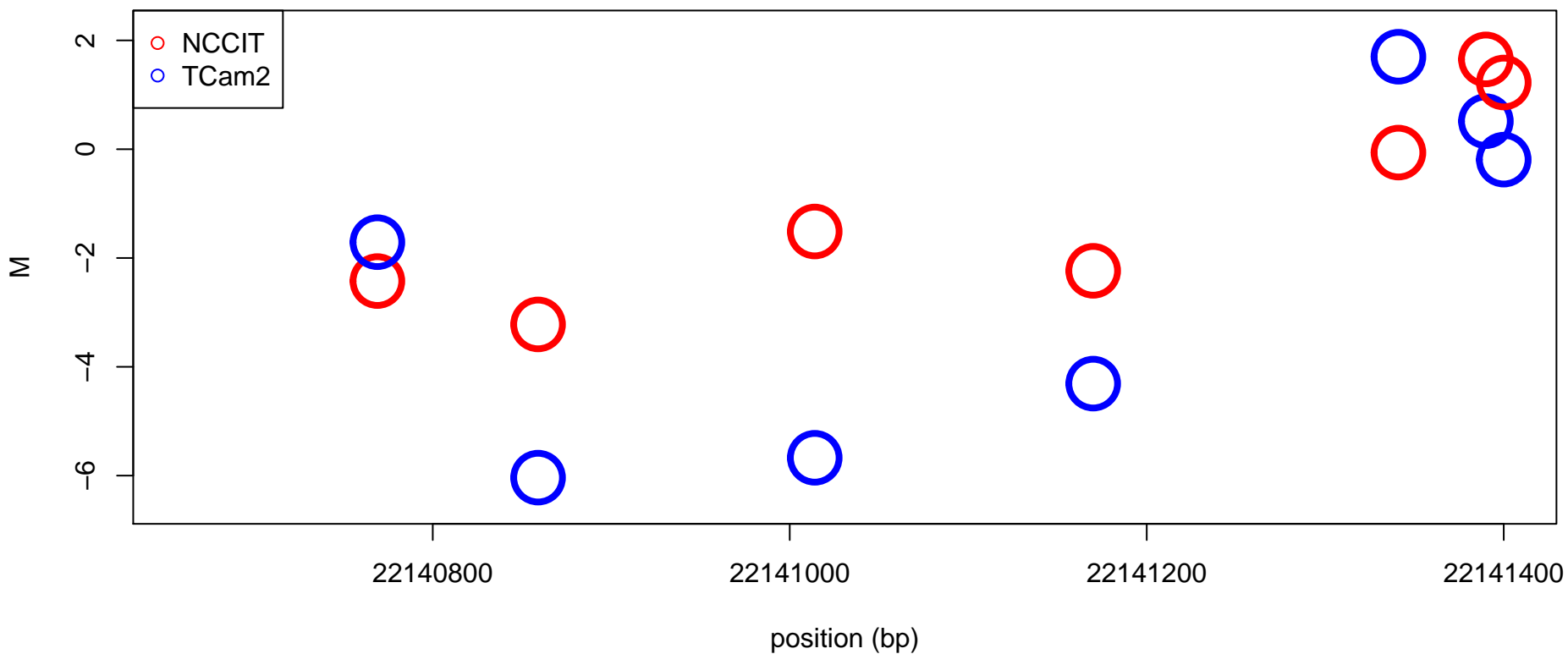

RegionID: 300, chr1:22140769–22141400–Beta\_values

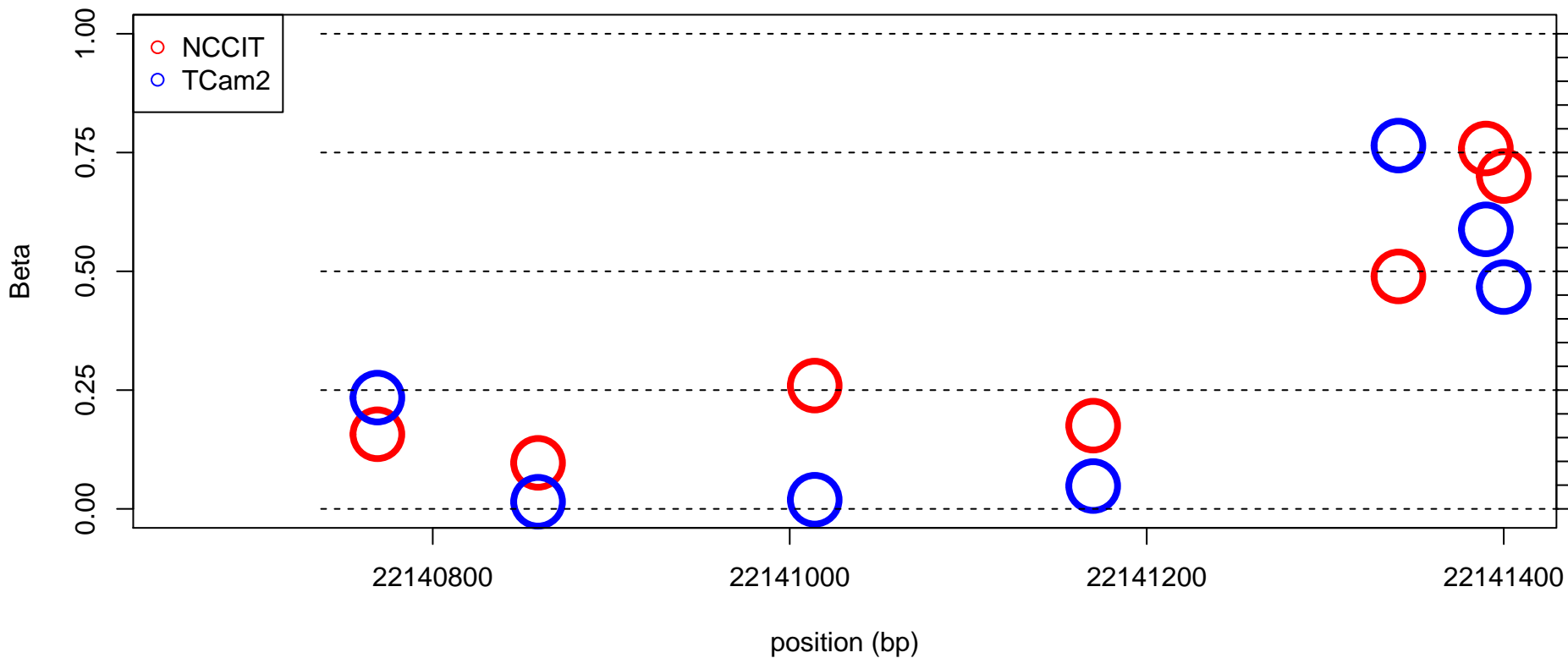

Supplement: File S1 — ZIP file containing DMRforPairs output for significant regions. Please start from the html files. (ZIP) [file pone.0098330.s008.zip › figures/300.pdf]

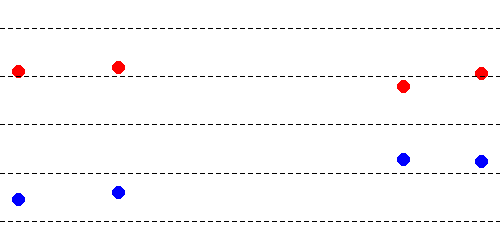

Supplement: File S1 — ZIP file containing DMRforPairs output for significant regions. Please start from the html files. (ZIP) [file pone.0098330.s008.zip › figures/306.png]

RegionID: 306, chr1:22919873–22920169–M\_values

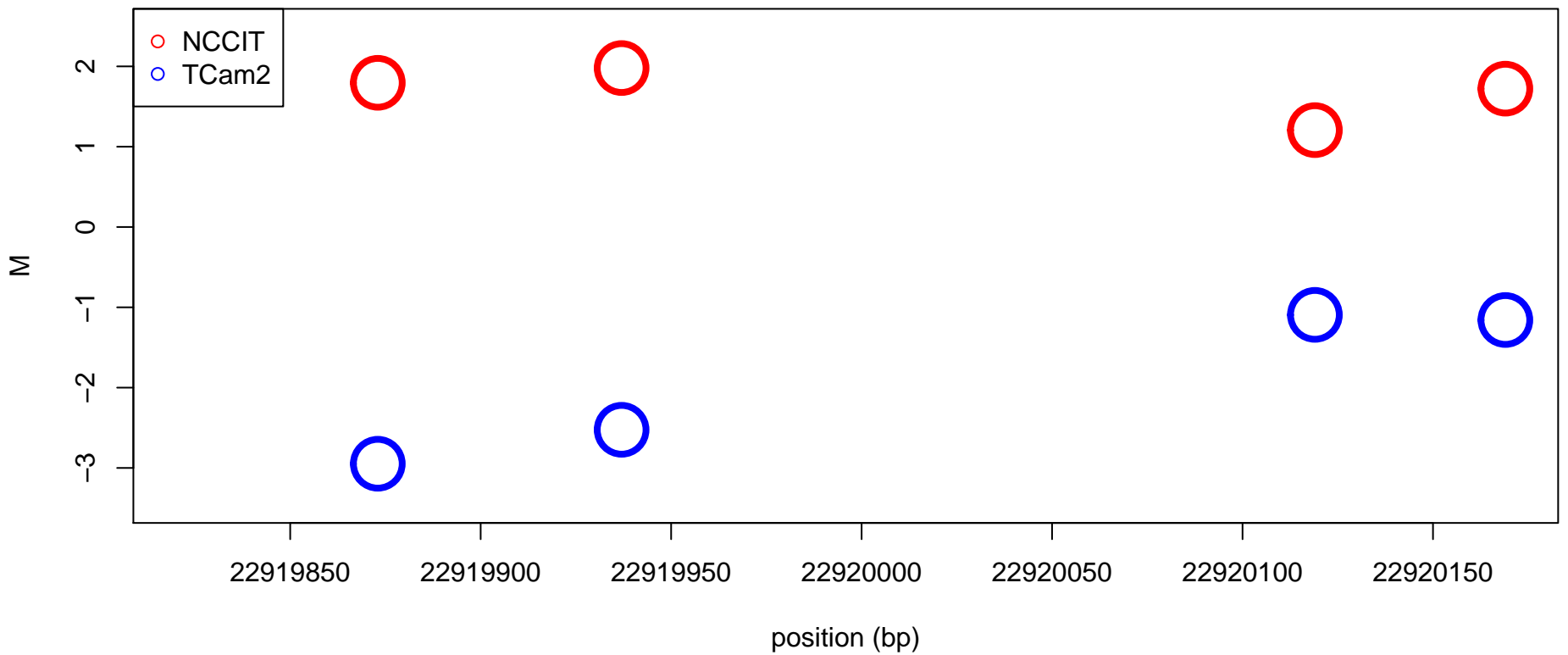

RegionID: 306, chr1:22919873–22920169–Beta\_values

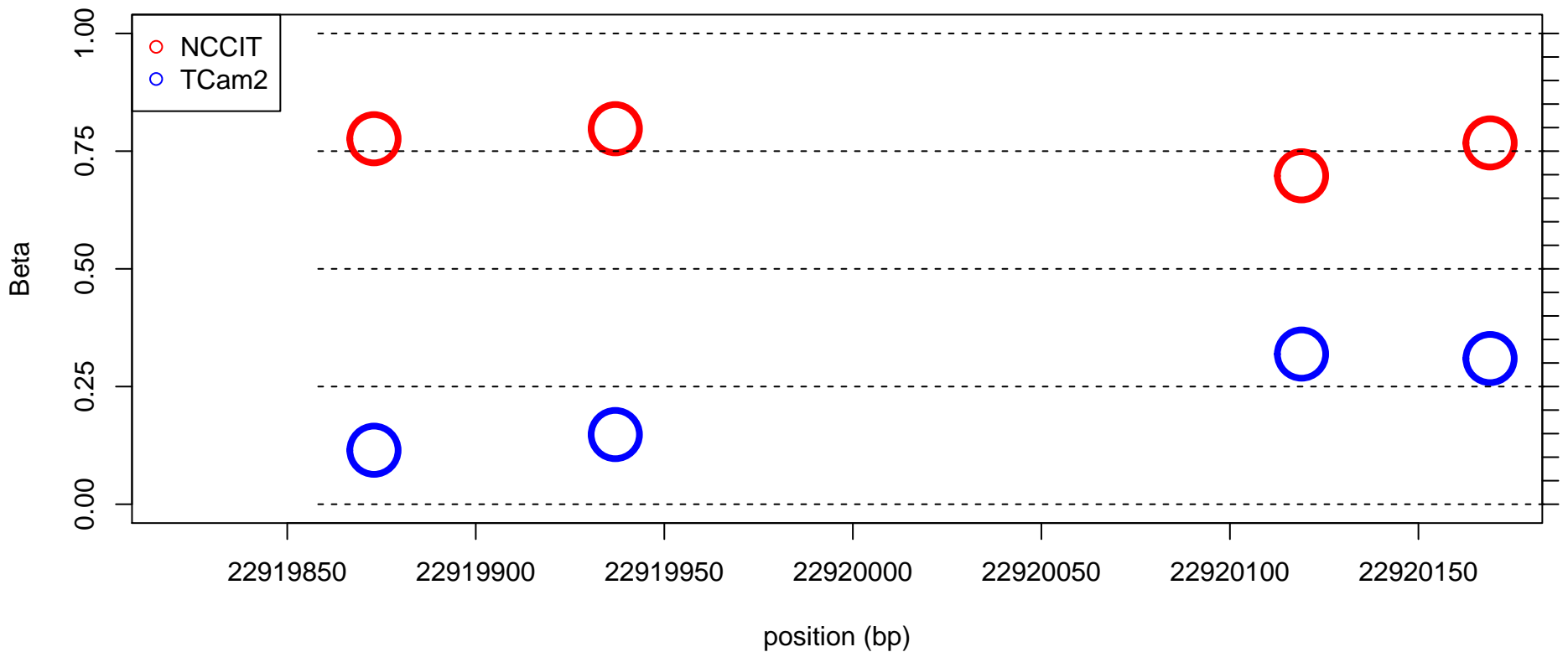

Supplement: File S1 — ZIP file containing DMRforPairs output for significant regions. Please start from the html files. (ZIP) [file pone.0098330.s008.zip › figures/306.pdf]
