# Supplementary material for: Seminoma and Embryonal Carcinoma Footprints Identified by Analysis of Integrated Genome-Wide Epigenetic and Expression Profiles of Germ Cell Cancer Cell Lines
Source: PLoS One. 2014 Jun 2;9(6):e98330. doi: 10.1371/journal.pone.0098330 (PMC4041891; doi:10.1371/journal.pone.0098330)
Supplement: Table S3 — Detailed results of motif enrichment analysis (HOMER) per cell line and per histone mark. (PDF) [file pone.0098330.s007.pdf]

Results homer motif enrichments (known motifs)

NCCIT h3k4me3

Homer Known Motif Enrichment Results

Homer *de novo* Motif Results
Gene Ontology Enrichment Results
Known Motif Enrichment Results (txt file)
Total Target Sequences = 19015, Total Background Sequences = 26126

Rank	Motif	Name	P-value	log P-pvalue	q-value (Benjamini)	# Target Sequences with Motif	% of Targets Sequences with Motif	# Background Sequences with Motif	% of Background Sequences with Motif	Motif File	PDF
1	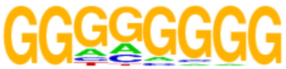	Maz(Zf)/HepG2-Maz-ChIP-Seq (GSE31477)/Homer	1e-401	-9.248e+02	0.0000	16119.0	84.77%	18698.3	71.57%	motif file (matrix)	pdf
2	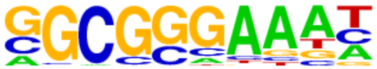	E2F4(E2F)/K562-E2F4-ChIP-Seq(GSE31477)/Homer	1e-202	-4.661e+02	0.0000	9320.0	49.01%	9969.5	38.16%	motif file (matrix)	pdf
3	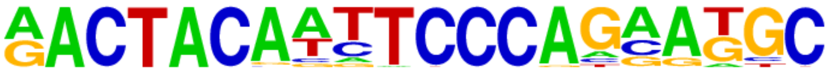	GFY-Staf/Promoters/Homer	1e-201	-4.637e+02	0.0000	1572.0	8.27%	922.4	3.53%	motif file (matrix)	pdf
4	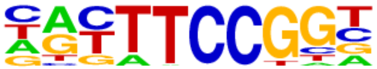	Elk4(ETS)/Hela-Elk4-ChIP-Seq (GSE31477)/Homer	1e-187	-4.323e+02	0.0000	9136.0	48.05%	9828.3	37.62%	motif file (matrix)	pdf
5	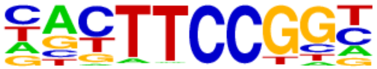	Elk1(ETS)/Hela-Elk1-ChIP-Seq (GSE31477)/Homer	1e-137	-3.170e+02	0.0000	8824.0	46.41%	9799.5	37.51%	motif file (matrix)	pdf
6	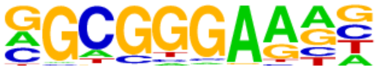	E2F6(E2F)/Hela-E2F6-ChIP-Seq(GSE31477)/Homer	1e-132	-3.053e+02	0.0000	10283.0	54.08%	11806.7	45.19%	motif file (matrix)	pdf
7	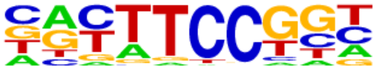	Fli1(ETS)/CD8-FLI-ChIP-Seq (GSE20898)/Homer	1e-120	-2.770e+02	0.0000	12460.0	65.53%	14952.1	57.23%	motif file (matrix)	pdf
8	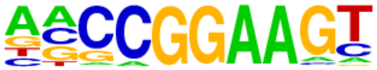	ELF1(ETS)/Jurkat-ELF1-ChIP-Seq/Homer	1e-110	-2.540e+02	0.0000	7972.0	41.92%	8912.4	34.11%	motif file (matrix)	pdf
9	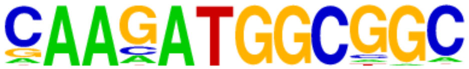	YY1(Zf)/Promoter/Homer	1e-105	-2.434e+02	0.0000	1847.0	9.71%	1487.0	5.69%	motif file (matrix)	pdf
10	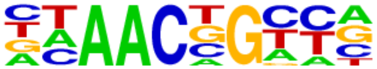	BMYB(HTH)/Hela-BMYB-ChIPSeq(GSE27030)/Homer	1e-103	-2.378e+02	0.0000	12992.0	68.33%	15876.7	60.77%	motif file (matrix)	pdf
11		Sp1(Zf)/Promoter/Homer	1e-95	-2.208e+02	0.0000	7766.0	40.84%	8777.9	33.60%	motif file (matrix)	pdf

	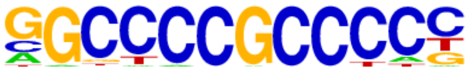										
12	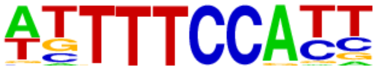	NFAT(RHD)/Jurkat-NFATC1-ChIP-Seq/Homer	1e-87	-2.009e+02	0.0000	8824.0	46.41%	10270.0	39.31%	motif file (matrix)	pdf
13	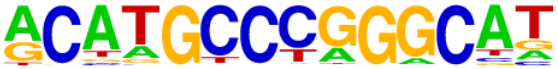	p53(p53)/mES-cMyc-ChIP-Seq/Homer	1e-84	-1.939e+02	0.0000	646.0	3.40%	372.9	1.43%	motif file (matrix)	pdf
14	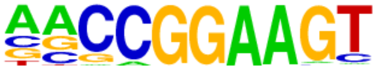	ETS(ETS)/Promoter/Homer	1e-78	-1.801e+02	0.0000	5114.0	26.89%	5529.7	21.17%	motif file (matrix)	pdf
15	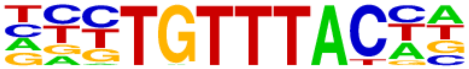	FOXP1(Forkhead)/H9-FOXP1-ChIP-Seq(GSE31006)/Homer	1e-70	-1.618e+02	0.0000	4728.0	24.86%	5117.4	19.59%	motif file (matrix)	pdf
16	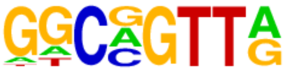	MYB(HTH)/ERMYB-Myb-ChIPSeq(GSE22095)/Homer	1e-65	-1.503e+02	0.0000	13988.0	73.56%	17729.9	67.86%	motif file (matrix)	pdf
17	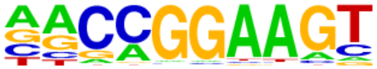	GABPA(ETS)/Jurkat-GABPa-ChIP-Seq/Homer	1e-63	-1.453e+02	0.0000	10078.0	53.00%	12253.5	46.90%	motif file (matrix)	pdf
18	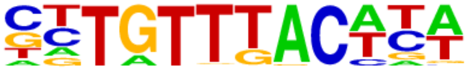	Foxa2(Forkhead)/Liver-Foxa2-ChIP-Seq/Homer	1e-54	-1.264e+02	0.0000	7025.0	36.94%	8252.5	31.59%	motif file (matrix)	pdf
19	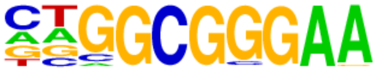	E2F1(E2F)/Hela-E2F1-ChIP-Seq/Hoemr	1e-54	-1.251e+02	0.0000	5950.0	31.29%	6851.1	26.22%	motif file (matrix)	pdf
20	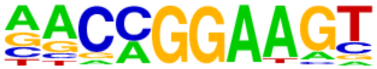	ETV1(ETS)/GIST48-ETV1-ChIP-Seq/Homer	1e-51	-1.176e+02	0.0000	13072.0	68.75%	16599.0	63.53%	motif file (matrix)	pdf
21	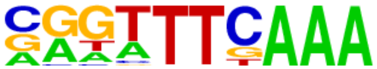	CHR/Cell-Cycle-Exp/Homer	1e-50	-1.168e+02	0.0000	6578.0	34.59%	7717.2	29.54%	motif file (matrix)	pdf
22	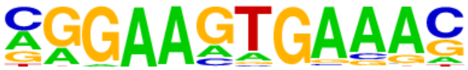	PU.1-IRF/Bcell-PU.1-ChIP-Seq/Homer	1e-50	-1.161e+02	0.0000	12451.0	65.48%	15727.1	60.20%	motif file (matrix)	pdf
23	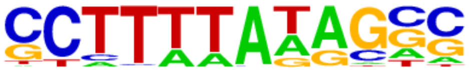	TATA-Box (TBP)/Promoter/Homer	1e-49	-1.131e+02	0.0000	11053.0	58.13%	13790.5	52.78%	motif file (matrix)	pdf
24		NFkB-p65(RHD)/GM12787-p65-ChIP-Seq/Homer	1e-46	-1.073e+02	0.0000	6861.0	36.08%	8145.8	31.18%	motif file (matrix)	pdf

	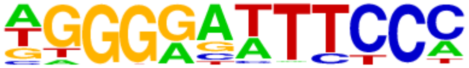										
25	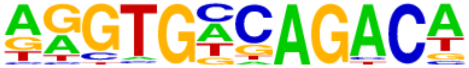	Tbox:Smad/ESCd5-Smad2_3-ChIP-Seq (GSE29422)/Homer	1e-45	-1.039e+02	0.0000	2485.0	13.07%	2578.1	9.87%	motif file (matrix)	pdf
26	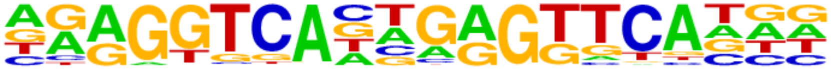	VDR(NR/DR3)/GM10855-VDR+vitD-ChIP-Seq/Homer	1e-41	-9.614e+01	0.0000	3064.0	16.11%	3320.1	12.71%	motif file (matrix)	pdf
27	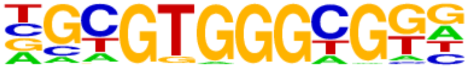	Egr2/Thymocytes-Egr2-ChIP-Seq(GSE34254)/Homer	1e-36	-8.325e+01	0.0000	4262.0	22.41%	4899.5	18.75%	motif file (matrix)	pdf
28	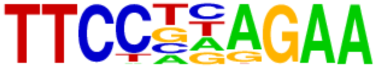	STAT6/Macrophage-Stat6-ChIP-Seq/Homer	1e-34	-8.028e+01	0.0000	5316.0	27.96%	6282.3	24.05%	motif file (matrix)	pdf
29	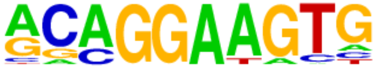	ETS1(ETS)/Jurkat-ETS1-ChIP-Seq/Homer	1e-33	-7.826e+01	0.0000	10676.0	56.15%	13511.5	51.72%	motif file (matrix)	pdf
30	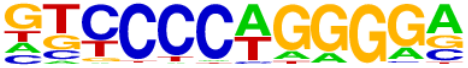	EBF1(EBF)/Near-E2A-ChIP-Seq/Homer	1e-33	-7.802e+01	0.0000	12284.0	64.60%	15752.6	60.29%	motif file (matrix)	pdf
31	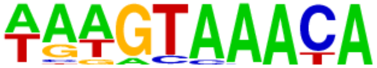	FOXA1(Forkhead)/LNCAP-FOXA1-ChIP-Seq/Homer	1e-31	-7.272e+01	0.0000	9676.0	50.89%	12179.9	46.62%	motif file (matrix)	pdf
32	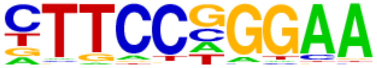	Stat3(Stat)/mES-Stat3-ChIP-Seq/Homer	1e-31	-7.260e+01	0.0000	5959.0	31.34%	7178.8	27.48%	motif file (matrix)	pdf
33	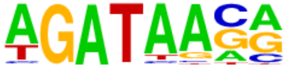	GATA3(Zf)/iTreg-Gata3-ChIP-Seq(GSE20898)/Homer	1e-30	-7.004e+01	0.0000	11025.0	57.98%	14059.9	53.82%	motif file (matrix)	pdf
34	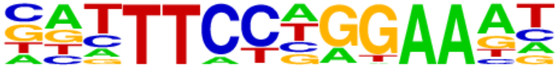	STAT1(Stat)/HelaS3-STAT1-ChIP-Seq/Homer	1e-30	-6.934e+01	0.0000	3197.0	16.81%	3619.9	13.86%	motif file (matrix)	pdf
35	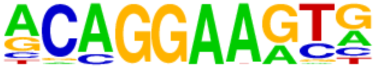	ERG(ETS)/VCaP-ERG-ChIP-Seq/Homer	1e-30	-6.912e+01	0.0000	13665.0	71.86%	17772.1	68.02%	motif file (matrix)	pdf
36	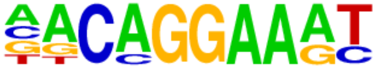	EWS:FLI1-fusion (ETS)/SK_N_MC-EWS:FLI1-ChIP-Seq/Homer	1e-28	-6.485e+01	0.0000	6529.0	34.34%	7991.2	30.59%	motif file (matrix)	pdf
37		Gata4(Zf)/Heart-Gata4-ChIP-Seq(GSE35151)/Homer	1e-28	-6.453e+01	0.0000	8161.0	42.92%	10184.1	38.98%	motif file (matrix)	pdf

	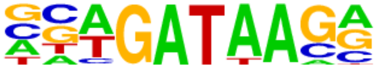										
38	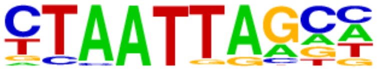	Lhx3(Homeobox)/Forebrain-p300-ChIP-Seq/Homer	1e-26	-6.212e+01	0.0000	8398.0	44.17%	10525.7	40.29%	motif file (matrix)	pdf
39	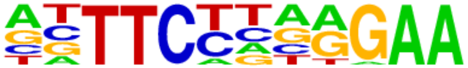	STAT6(Stat)/CD4-Stat6-ChIP-Seq/Homer	1e-26	-6.014e+01	0.0000	5241.0	27.56%	6322.4	24.20%	motif file (matrix)	pdf
40	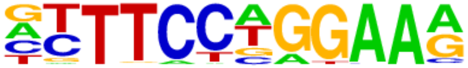	STAT4(Stat)/CD4-Stat4-ChIP-Seq/Homer	1e-25	-5.761e+01	0.0000	8619.0	45.33%	10864.8	41.59%	motif file (matrix)	pdf
41	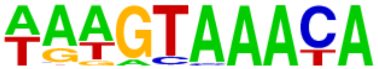	FOXA1(Forkhead)/MCF7-FOXA1-ChIP-Seq/Homer	1e-24	-5.607e+01	0.0000	8578.0	45.11%	10822.6	41.42%	motif file (matrix)	pdf
42	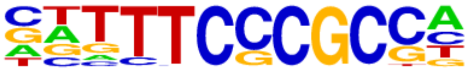	E2F7(E2F)/Hela-E2F7-ChIP-Seq(GSE32673)/Homer	1e-23	-5.506e+01	0.0000	3010.0	15.83%	3464.8	13.26%	motif file (matrix)	pdf
43	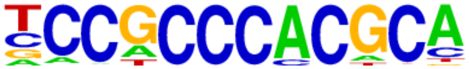	EGR(Zf)/K562-EGR1-ChIP-Seq/Homer	1e-23	-5.485e+01	0.0000	4543.0	23.89%	5446.1	20.85%	motif file (matrix)	pdf
44	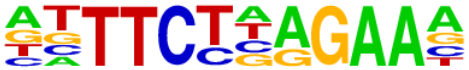	STAT5 (Stat)/mCD4+-Stat5a|b-ChIP-Seq/Homer	1e-23	-5.406e+01	0.0000	3472.0	18.26%	4062.6	15.55%	motif file (matrix)	pdf
45	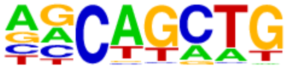	SCL/HPC7-Scl-ChIP-Seq/Homer	1e-22	-5.205e+01	0.0000	18524.0	97.42%	25109.9	96.11%	motif file (matrix)	pdf
46	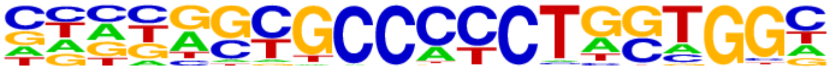	BORIS(Zf)/K562-CTCFL-ChIP-Seq/Homer	1e-21	-4.916e+01	0.0000	4027.0	21.18%	4815.5	18.43%	motif file (matrix)	pdf
47	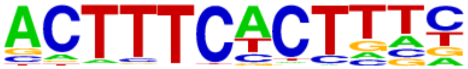	PRDM1/BMI1(Zf)/Hela-PRDM1-ChIP-Seq (GSE31477)/Homer	1e-21	-4.840e+01	0.0000	5910.0	31.08%	7302.6	27.95%	motif file (matrix)	pdf
48	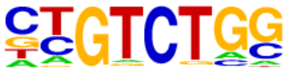	Smad2(MAD)/ES-SMAD2-ChIP-Seq(GSE29422)/Homer	1e-20	-4.815e+01	0.0000	13521.0	71.11%	17745.1	67.92%	motif file (matrix)	pdf
49	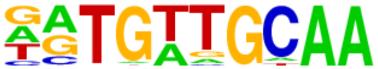	CEBP:AP1/ThioMac-CEBPb-ChIP-Seq/Homer	1e-20	-4.706e+01	0.0000	6469.0	34.02%	8060.9	30.85%	motif file (matrix)	pdf
50		AR-halfsite(NR)/LNCaP-AR-ChIP-Seq/Homer	1e-20	-4.682e+01	0.0000	17970.0	94.50%	24249.1	92.82%	motif file (matrix)	pdf

	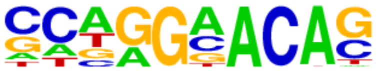										
51	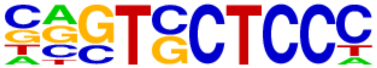	Znf263(Zf)/K562-Znf263-ChIP-Seq/Homer	1e-20	-4.665e+01	0.0000	15882.0	83.52%	21138.6	80.91%	motif file (matrix)	pdf
52	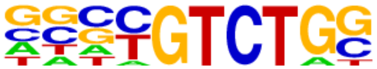	Smad4(MAD)/ESC-SMAD4-ChIP-Seq(GSE29422)/Homer	1e-20	-4.615e+01	0.0000	13758.0	72.35%	18099.8	69.28%	motif file (matrix)	pdf
53	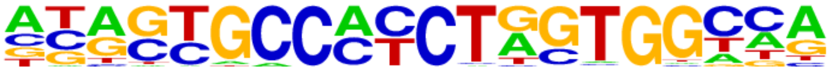	CTCF(Zf)/CD4+-CTCF-ChIP-Seq/Homer	1e-19	-4.526e+01	0.0000	1902.0	10.00%	2122.6	8.12%	motif file (matrix)	pdf
54	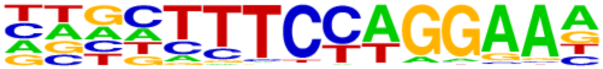	Bcl6(Zf)/Liver-Bcl6-ChIP-Seq (GSE31578)/Homer	1e-19	-4.435e+01	0.0000	11630.0	61.16%	15132.2	57.92%	motif file (matrix)	pdf
55	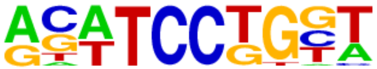	SPDEF(ETS)/VCaP-SPDEF-ChIP-Seq/Homer	1e-18	-4.333e+01	0.0000	9922.0	52.18%	12782.3	48.93%	motif file (matrix)	pdf
56	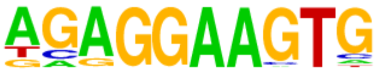	PU.1(ETS)/ThioMac-PU.1-ChIP-Seq/Homer	1e-18	-4.296e+01	0.0000	5264.0	27.68%	6492.3	24.85%	motif file (matrix)	pdf
57	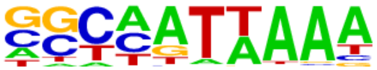	Unknown/Homeobox/Limb-p300-ChIP-Seq/Homer	1e-18	-4.276e+01	0.0000	6217.0	32.70%	7763.7	29.72%	motif file (matrix)	pdf
58	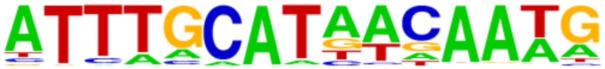	OCT4-SOX2-TCF-NANOG ((POU/Homeobox/HMG)/mES-ChIP-Seq/Homer	1e-18	-4.257e+01	0.0000	1883.0	9.90%	2113.9	8.09%	motif file (matrix)	pdf
59	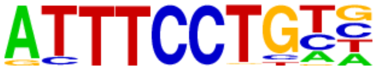	EWS:ERG-fusion (ETS)/CADO_ES1-EWS:ERG-ChIP-Seq/Homer	1e-17	-4.143e+01	0.0000	6963.0	36.62%	8777.4	33.60%	motif file (matrix)	pdf
60	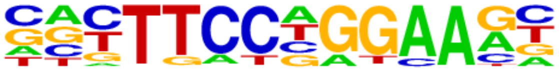	Stat3+il23(Stat)/CD4-Stat3-ChIP-Seq/Homer	1e-17	-3.918e+01	0.0000	7196.0	37.84%	9114.9	34.89%	motif file (matrix)	pdf
61	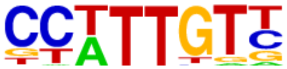	Sox3(HMG)/NPC-Sox3-ChIP-Seq(GSE33059)/Homer	1e-16	-3.814e+01	0.0000	12120.0	63.74%	15880.9	60.79%	motif file (matrix)	pdf
62	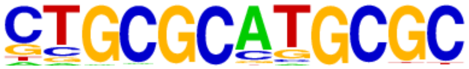	NRF1(NRF)/MCF7-NRF1-ChIP-Seq/Homer	1e-16	-3.757e+01	0.0000	3043.0	16.00%	3625.9	13.88%	motif file (matrix)	pdf
63		E2F(E2F)/Cell-Cycle-Exp/Homer	1e-16	-3.728e+01	0.0000	1252.0	6.58%	1358.8	5.20%	motif file (matrix)	pdf

	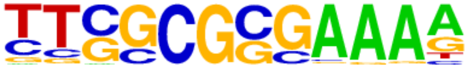										
64	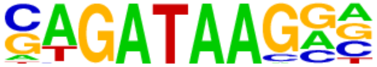	Gata1(Zf)/K562-GATA1-ChIP-Seq/Homer	1e-14	-3.454e+01	0.0000	5037.0	26.49%	6270.7	24.00%	motif file (matrix)	pdf
65	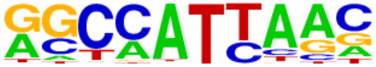	Nanog(Homeobox)/mES-Nanog-ChIP-Seq/Homer	1e-13	-3.195e+01	0.0000	18066.0	95.01%	24482.6	93.71%	motif file (matrix)	pdf
66	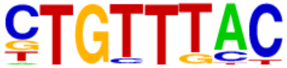	Foxo1(Forkhead)/RAW-Foxo1-ChIP-Seq/Homer	1e-13	-3.128e+01	0.0000	13639.0	71.73%	18085.2	69.22%	motif file (matrix)	pdf
67	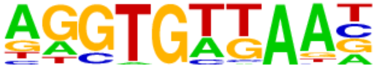	Eomes(T-box)/H9-Eomes-ChIP-Seq/Homer	1e-13	-3.106e+01	0.0000	17172.0	90.31%	23151.1	88.61%	motif file (matrix)	pdf
68	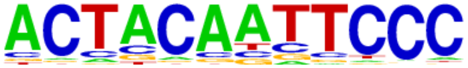	GFY(?)/Promoter/Homer	1e-12	-2.908e+01	0.0000	1134.0	5.96%	1254.3	4.80%	motif file (matrix)	pdf
69	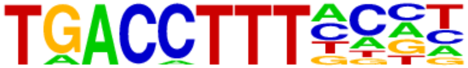	Nur77(NR)/K562-NR4A1-ChIP-Seq(GSE31363)/Homer	1e-12	-2.904e+01	0.0000	1722.0	9.06%	1992.5	7.63%	motif file (matrix)	pdf
70	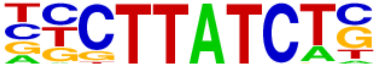	Gata2(Zf)/K562-GATA2-ChIP-Seq/Homer	1e-11	-2.602e+01	0.0000	5560.0	29.24%	7061.1	27.03%	motif file (matrix)	pdf
71	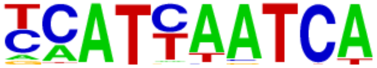	Pdx1(Homeobox)/Islet-Pdx1-ChIP-Seq/Homer	1e-11	-2.570e+01	0.0000	7085.0	37.26%	9120.4	34.91%	motif file (matrix)	pdf
72	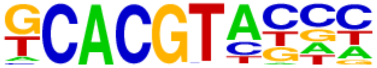	HIF2a(HLH)/O785-HIF2a-ChIP-Seq(GSE34871)/Homer	1e-10	-2.523e+01	0.0000	4833.0	25.42%	6098.5	23.34%	motif file (matrix)	pdf
73	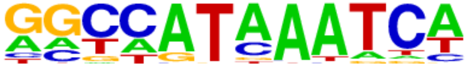	HOXA9/HSC-Hoxa9-ChIP-Seq (GSE33509)/Homer	1e-10	-2.504e+01	0.0000	5540.0	29.13%	7046.4	26.97%	motif file (matrix)	pdf
74	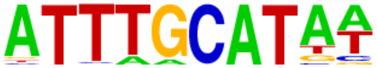	Oct4(POU/Homeobox)/mES-Oct4-ChIP-Seq/Homer	1e-10	-2.353e+01	0.0000	4216.0	22.17%	5296.3	20.27%	motif file (matrix)	pdf
75	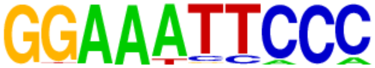	NFkB-p65-Rel(RHD)/LPS-exp/Homer	1e-10	-2.337e+01	0.0000	783.0	4.12%	851.7	3.26%	motif file (matrix)	pdf
76		Hnf1(Homeobox)/Liver-Foxa2-Chip-Seq/Homer	1e-9	-2.281e+01	0.0000	1523.0	8.01%	1782.7	6.82%	motif file (matrix)	pdf

	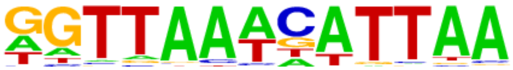										
77	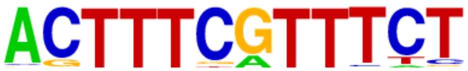	T1ISRE(IRF)/Ifnb-Exp/Homer	1e-9	-2.236e+01	0.0000	157.0	0.83%	125.6	0.48%	motif file (matrix)	pdf
78	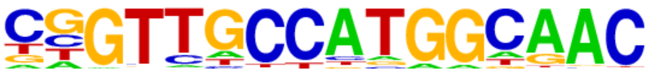	RFX(HTH)/K562-RFX3-ChIP-Seq/Homer	1e-9	-2.191e+01	0.0000	1125.0	5.92%	1284.6	4.92%	motif file (matrix)	pdf
79	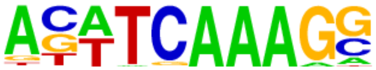	Tcf3(HMG)/mES-Tcf3-ChIP-Seq/Homer	1e-9	-2.135e+01	0.0000	2241.0	11.79%	2719.2	10.41%	motif file (matrix)	pdf
80	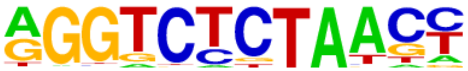	PRDM14(Zf)/H1-PRDM14-ChIP-Seq/Homer	1e-8	-2.007e+01	0.0000	3237.0	17.02%	4039.0	15.46%	motif file (matrix)	pdf
81	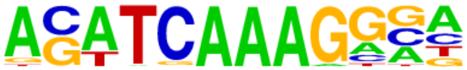	Tcf4(HMG)/Hct116-Tcf4-ChIP-Seq/Homer	1e-8	-1.973e+01	0.0000	3913.0	20.58%	4938.7	18.90%	motif file (matrix)	pdf
82	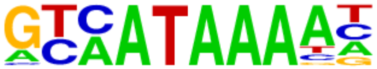	Cdx2(Homeobox)/mES-Cdx2-ChIP-Seq/Homer	1e-8	-1.946e+01	0.0000	6157.0	32.38%	7951.6	30.44%	motif file (matrix)	pdf
83	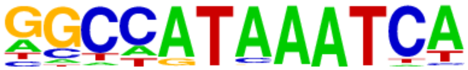	Hoxc9/Ainv15-Hoxc9-ChIP-Seq/Homer	1e-7	-1.837e+01	0.0000	4448.0	23.39%	5669.1	21.70%	motif file (matrix)	pdf
84	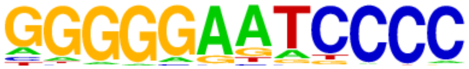	NFkB-p50,p52(RHD)/p50-ChIP-Chip/Homer	1e-7	-1.702e+01	0.0000	2335.0	12.28%	2884.5	11.04%	motif file (matrix)	pdf
85	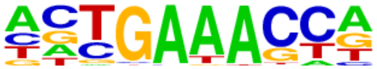	IRF4(IRF)/GM12878-IRF4-ChIP-Seq/Homer	1e-7	-1.653e+01	0.0000	4299.0	22.61%	5495.8	21.04%	motif file (matrix)	pdf
86	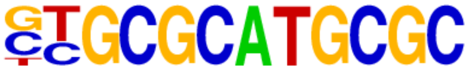	NRF1/Promoter/Homer	1e-6	-1.610e+01	0.0000	2960.0	15.57%	3718.6	14.23%	motif file (matrix)	pdf
87	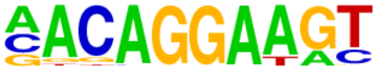	Ets1-distal(ETS)/CD4+-PolII-ChIP-Seq/Homer	1e-6	-1.567e+01	0.0000	2823.0	14.85%	3542.9	13.56%	motif file (matrix)	pdf
88	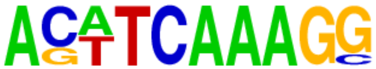	TCFL2(HMG)/K562-TCF7L2-ChIP-Seq(GSE29196)/Homer	1e-6	-1.534e+01	0.0000	709.0	3.73%	803.6	3.08%	motif file (matrix)	pdf
89		HNF6(Homeobox)/Liver-Hnf6-ChIP-Seq(ERP000394)	1e-6	-1.528e+01	0.0000	4744.0	24.95%	6110.4	23.39%	motif file (matrix)	pdf

	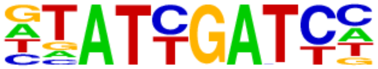										
90	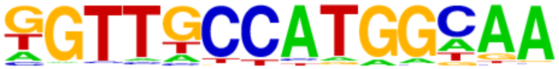	X-box(HTH)/NPC-H3K4me1-ChIP-Seq/Homer	1e-6	-1.479e+01	0.0000	1118.0	5.88%	1325.5	5.07%	motif file (matrix)	pdf
91	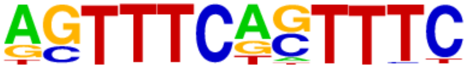	ISRE(IRF)/ThioMac-LPS-exp/HOMER	1e-6	-1.431e+01	0.0000	731.0	3.84%	837.3	3.20%	motif file (matrix)	pdf
92	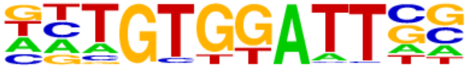	Foxh1(Forkhead)/hESC-FOXH1-ChIP-Seq (GSE29422)/Homer	1e-5	-1.270e+01	0.0000	5612.0	29.51%	7323.1	28.03%	motif file (matrix)	pdf
93	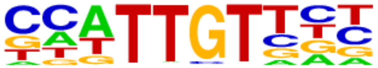	Sox6(HMG)/Myotubes-Sox6-ChIP-Seq(GSE32627)/Homer	1e-5	-1.265e+01	0.0000	11242.0	59.12%	15023.6	57.50%	motif file (matrix)	pdf
94	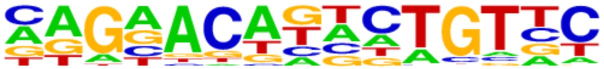	PR(NR)/T47D-PR-ChIP-Seq (GSE31130)/Homer	1e-4	-1.111e+01	0.0000	14084.0	74.07%	18999.4	72.72%	motif file (matrix)	pdf
95	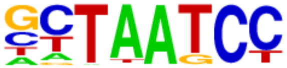	CRX(Homeobox)/Retina-Crx-ChIP-Seq/Homer	1e-4	-1.031e+01	0.0001	15702.0	82.58%	21281.9	81.46%	motif file (matrix)	pdf
96	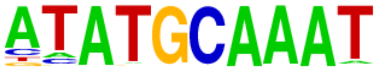	Oct2(POU/Homeobox)/Bcell-Oct2-ChIP-Seq/Homer	1e-4	-9.832e+00	0.0001	2820.0	14.83%	3618.4	13.85%	motif file (matrix)	pdf
97	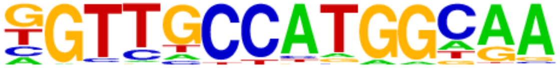	Rfx1(HTH)/NPC-Rfx1-ChIP-Seq/Homer	1e-4	-9.664e+00	0.0001	2215.0	11.65%	2815.6	10.78%	motif file (matrix)	pdf
98	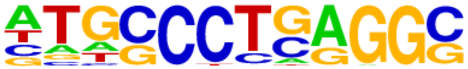	AP-2alpha(AP2)/Hela-AP2alpha-ChIP-Seq/Homer	1e-4	-9.365e+00	0.0002	11227.0	59.04%	15073.4	57.69%	motif file (matrix)	pdf
99	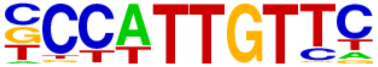	Sox2(HMG)/mES-Sox2-ChIP-Seq/Homer	1e-3	-8.476e+00	0.0005	7253.0	38.14%	9641.3	36.90%	motif file (matrix)	pdf
100	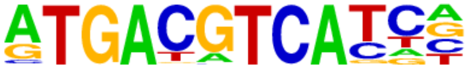	JunD(bZIP)/K562-JunD-ChIP-Seq/Homer	1e-3	-7.963e+00	0.0007	778.0	4.09%	946.9	3.62%	motif file (matrix)	pdf
101	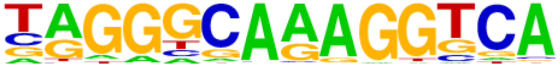	RXR(NR/DR1)/3T3L1-RXR-ChIP-Seq/Homer	1e-3	-7.907e+00	0.0008	11122.0	58.49%	14964.3	57.28%	motif file (matrix)	pdf
102		MyoD(HLH)/Myotube-MyoD-ChIP-Seq/Homer	1e-2	-6.326e+00	0.0038	8060.0	42.39%	10801.6	41.34%	motif file (matrix)	pdf

	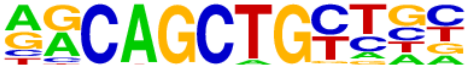										
103	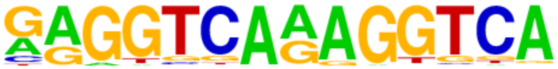	TR4(NR/DR1)/Hela-TR4-ChIP-Seq/Homer	1e-2	-6.129e+00	0.0045	1366.0	7.18%	1740.8	6.66%	motif file (matrix)	pdf
104	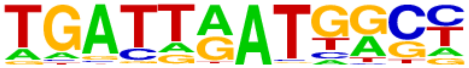	Hoxb4/ES-Hoxb4-ChIP-Seq (GSE34014)/Homer	1e-2	-5.559e+00	0.0080	1563.0	8.22%	2011.3	7.70%	motif file (matrix)	pdf
105	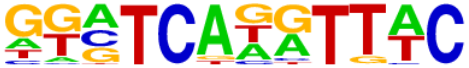	Six1(Homeobox)/Myoblast-Six1-ChIP-Chip (GSE20150)/Homer	1e-2	-5.464e+00	0.0087	2305.0	12.12%	3006.4	11.51%	motif file (matrix)	pdf

Results homer motif enrichments (known motifs)

NCCIT h3k27ac

Homer Known Motif Enrichment Results

Homer *de novo* Motif Results
Gene Ontology Enrichment Results
Known Motif Enrichment Results (txt file)
Total Target Sequences = 23765, Total Background Sequences = 24649

Rank	Motif	Name	P-value	log P-pvalue	q-value (Benjamini)	# Target Sequences with Motif	% of Targets Sequences with Motif	# Background Sequences with Motif	% of Background Sequences with Motif	Motif File	PDF
1	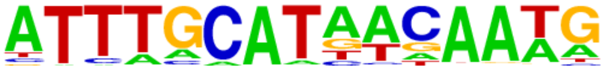	OCT4-SOX2-TCF-NANOG ((POU/Homeobox/HMG)/mES-ChIP-Seq/Homer	1e-339	-7.813e+02	0.0000	5454.0	22.95%	3325.1	13.49%	motif file (matrix)	pdf
2	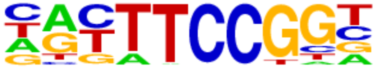	Elk4(ETS)/Hela-Elk4-ChIP-Seq (GSE31477)/Homer	1e-244	-5.634e+02	0.0000	8137.0	34.24%	6049.5	24.55%	motif file (matrix)	pdf
3	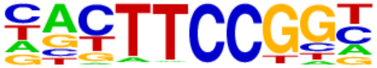	Elk1(ETS)/Hela-Elk1-ChIP-Seq (GSE31477)/Homer	1e-228	-5.263e+02	0.0000	8088.0	34.04%	6078.9	24.67%	motif file (matrix)	pdf
4	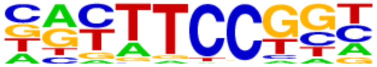	Fli1(ETS)/CD8-FLI-ChIP-Seq (GSE20898)/Homer	1e-227	-5.238e+02	0.0000	14162.0	59.60%	12115.4	49.17%	motif file (matrix)	pdf
5	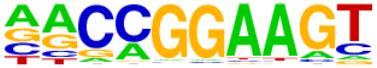	GABPA(ETS)/Jurkat-GABPa-ChIP-Seq/Homer	1e-177	-4.097e+02	0.0000	12045.0	50.69%	10229.3	41.51%	motif file (matrix)	pdf
6	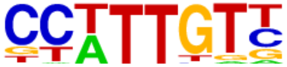	Sox3(HMG)/NPC-Sox3-ChIP-Seq(GSE33059)/Homer	1e-170	-3.934e+02	0.0000	18849.0	79.32%	17593.4	71.40%	motif file (matrix)	pdf
7	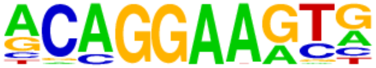	ERG(ETS)/VCaP-ERG-ChIP-Seq/Homer	1e-166	-3.836e+02	0.0000	17766.0	74.76%	16393.5	66.53%	motif file (matrix)	pdf
8	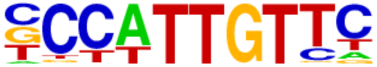	Sox2(HMG)/mES-Sox2-ChIP-Seq/Homer	1e-165	-3.801e+02	0.0000	12787.0	53.81%	11071.1	44.93%	motif file (matrix)	pdf
9	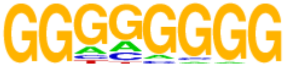	Maz(Zf)/HepG2-Maz-ChIP-Seq (GSE31477)/Homer	1e-164	-3.778e+02	0.0000	13925.0	58.60%	12260.0	49.76%	motif file (matrix)	pdf
10	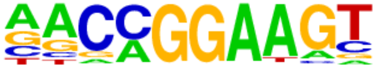	ETV1(ETS)/GIST48-ETV1-ChIP-Seq/Homer	1e-163	-3.774e+02	0.0000	15972.0	67.21%	14438.5	58.60%	motif file (matrix)	pdf
11		EWS:FLI1-fusion (ETS)/SK_N_MC-EWS:FLI1-ChIP-Seq/Homer	1e-162	-3.749e+02	0.0000	8751.0	36.83%	7057.5	28.64%	motif file (matrix)	pdf

	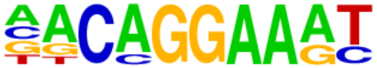										
12	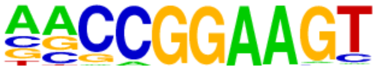	ETS(ETS)/Promoter/Homer	1e-158	-3.658e+02	0.0000	5145.0	21.65%	3715.5	15.08%	motif file (matrix)	pdf
13	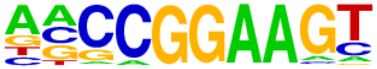	ELF1(ETS)/Jurkat-ELF1-ChIP-Seq/Homer	1e-154	-3.561e+02	0.0000	7405.0	31.16%	5818.7	23.61%	motif file (matrix)	pdf
14	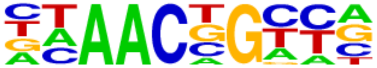	BMYB(HTH)/Hela-BMYB-ChIPSeq(GSE27030)/Homer	1e-142	-3.274e+02	0.0000	16931.0	71.25%	15633.9	63.45%	motif file (matrix)	pdf
15	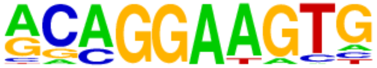	ETS1(ETS)/Jurkat-ETS1-ChIP-Seq/Homer	1e-141	-3.252e+02	0.0000	13458.0	56.63%	11931.5	48.42%	motif file (matrix)	pdf
16	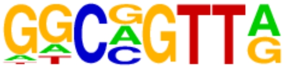	MYB(HTH)/ERMYB-Myb-ChIPSeq(GSE22095)/Homer	1e-127	-2.936e+02	0.0000	17972.0	75.63%	16891.0	68.55%	motif file (matrix)	pdf
17	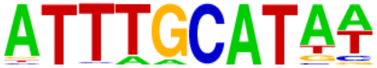	Oct4(POU/Homeobox)/mES-Oct4-ChIP-Seq/Homer	1e-122	-2.831e+02	0.0000	8890.0	37.41%	7449.1	30.23%	motif file (matrix)	pdf
18	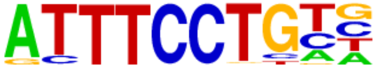	EWS:ERG-fusion (ETS)/CADO_ES1-EWS:ERG-ChIP-Seq/Homer	1e-106	-2.448e+02	0.0000	11190.0	47.09%	9870.3	40.06%	motif file (matrix)	pdf
19	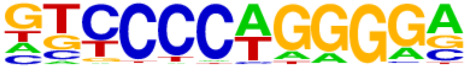	EBF1(EBF)/Near-E2A-ChIP-Seq/Homer	1e-105	-2.419e+02	0.0000	12778.0	53.77%	11507.0	46.70%	motif file (matrix)	pdf
20	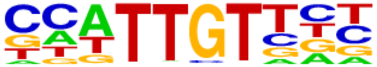	Sox6(HMG)/Myotubes-Sox6-ChIP-Seq(GSE32627)/Homer	1e-103	-2.391e+02	0.0000	17972.0	75.63%	17070.4	69.28%	motif file (matrix)	pdf
21	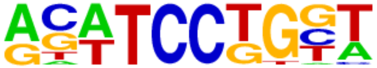	SPDEF(ETS)/VCaP-SPDEF-ChIP-Seq/Homer	1e-101	-2.329e+02	0.0000	13125.0	55.23%	11900.4	48.30%	motif file (matrix)	pdf
22	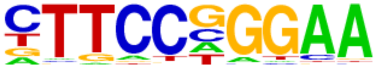	Stat3(Stat)/mES-Stat3-ChIP-Seq/Homer	1e-93	-2.161e+02	0.0000	7438.0	31.30%	6246.3	25.35%	motif file (matrix)	pdf
23	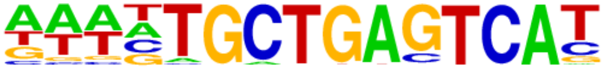	Bach1(bZIP)/K562-Bach1-ChIP-Seq(GSE31477)/Homer	1e-90	-2.074e+02	0.0000	1155.0	4.86%	627.6	2.55%	motif file (matrix)	pdf
24		Nrf2(bZIP)/Lymphoblast-Nrf2-ChIP-Seq(GSE37589)/Homer	1e-87	-2.014e+02	0.0000	1038.0	4.37%	547.3	2.22%	motif file (matrix)	pdf

	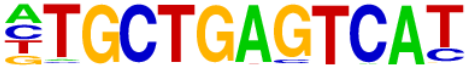										
25	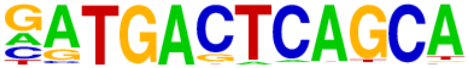	NF-E2(bZIP)/K562-NFE2-ChIP-Seq/Homer	1e-87	-2.008e+02	0.0000	1177.0	4.95%	651.3	2.64%	motif file (matrix)	pdf
26	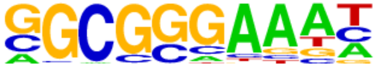	E2F4(E2F)/K562-E2F4-ChIP-Seq(GSE31477)/Homer	1e-81	-1.865e+02	0.0000	5586.0	23.51%	4567.5	18.54%	motif file (matrix)	pdf
27	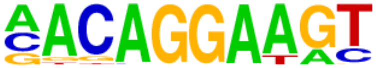	Ets1-distal(ETS)/CD4+-PolII-ChIP-Seq/Homer	1e-76	-1.768e+02	0.0000	4673.0	19.67%	3739.1	15.17%	motif file (matrix)	pdf
28	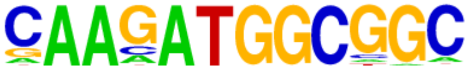	YY1(Zf)/Promoter/Homer	1e-76	-1.761e+02	0.0000	1515.0	6.38%	948.9	3.85%	motif file (matrix)	pdf
29	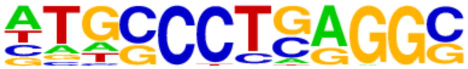	AP-2alpha(AP2)/Hela-AP2alpha-ChIP-Seq/Homer	1e-75	-1.738e+02	0.0000	10701.0	45.03%	9646.6	39.15%	motif file (matrix)	pdf
30	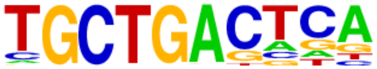	MafA(bZIP)/Islet-MafA-ChIP-Seq(GSE30298)/Homer	1e-70	-1.614e+02	0.0000	9799.0	41.24%	8787.8	35.66%	motif file (matrix)	pdf
31	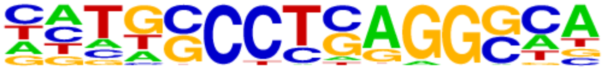	AP2gamma(AP2)/MCF7-TFAP2c-ChIP-Seq/Homer	1e-69	-1.609e+02	0.0000	11178.0	47.04%	10187.8	41.35%	motif file (matrix)	pdf
32	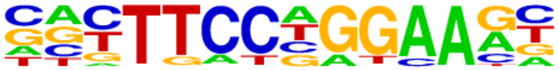	Stat3+il23(Stat)/CD4-Stat3-ChIP-Seq/Homer	1e-69	-1.607e+02	0.0000	9833.0	41.38%	8824.0	35.81%	motif file (matrix)	pdf
33	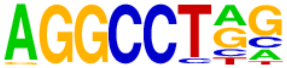	ZFX(Zf)/mES-Zfx-ChIP-Seq/Homer	1e-69	-1.601e+02	0.0000	14520.0	61.10%	13658.7	55.43%	motif file (matrix)	pdf
34	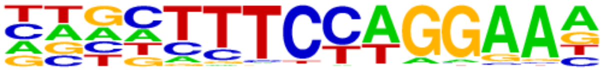	Bcl6(Zf)/Liver-Bcl6-ChIP-Seq (GSE31578)/Homer	1e-66	-1.542e+02	0.0000	16215.0	68.24%	15488.7	62.86%	motif file (matrix)	pdf
35	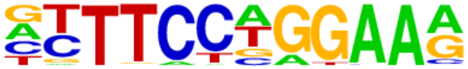	STAT4(Stat)/CD4-Stat4-ChIP-Seq/Homer	1e-65	-1.510e+02	0.0000	12929.0	54.41%	12035.2	48.84%	motif file (matrix)	pdf
36	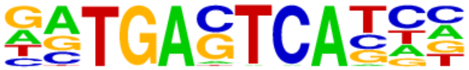	Jun-AP1(bZIP)/K562-cJun-ChIP-Seq/Homer	1e-64	-1.483e+02	0.0000	3616.0	15.22%	2844.1	11.54%	motif file (matrix)	pdf
37		NRF1(NRF)/MCF7-NRF1-ChIP-Seq/Homer	1e-63	-1.470e+02	0.0000	1485.0	6.25%	971.0	3.94%	motif file (matrix)	pdf

	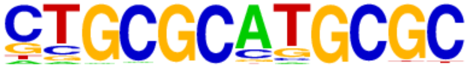										
38	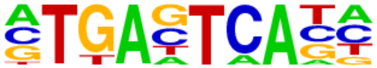	HIF1b(HLH)/O785-HIF1b-ChIP-Seq(GSE34871)/Homer	1e-61	-1.413e+02	0.0000	8173.0	34.39%	7249.0	29.42%	motif file (matrix)	pdf
39	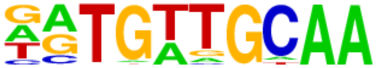	CEBP:AP1/ThioMac-CEBPb-ChIP-Seq/Homer	1e-57	-1.327e+02	0.0000	11131.0	46.84%	10269.1	41.68%	motif file (matrix)	pdf
40	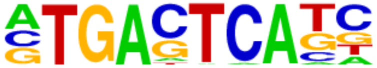	AP-1(bZIP)/ThioMac-PU.1-ChIP-Seq/Homer	1e-57	-1.327e+02	0.0000	9866.0	41.52%	8983.5	36.46%	motif file (matrix)	pdf
41	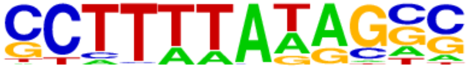	TATA-Box (TBP)/Promoter/Homer	1e-56	-1.308e+02	0.0000	16292.0	68.56%	15680.0	63.64%	motif file (matrix)	pdf
42	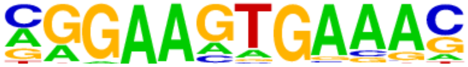	PU.1-IRF/Bcell-PU.1-ChIP-Seq/Homer	1e-54	-1.256e+02	0.0000	16603.0	69.87%	16039.6	65.09%	motif file (matrix)	pdf
43	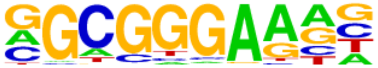	E2F6(E2F)/Hela-E2F6-ChIP-Seq(GSE31477)/Homer	1e-53	-1.239e+02	0.0000	6823.0	28.71%	5991.5	24.32%	motif file (matrix)	pdf
44	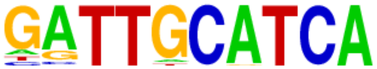	AARE(HLH)/mES-cMyc-ChIP-Seq/Homer	1e-49	-1.133e+02	0.0000	2132.0	8.97%	1596.9	6.48%	motif file (matrix)	pdf
45	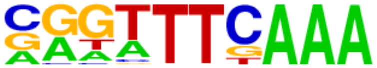	CHR/Cell-Cycle-Exp/Homer	1e-47	-1.092e+02	0.0000	10689.0	44.98%	9937.3	40.33%	motif file (matrix)	pdf
46	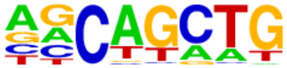	SCL/HPC7-Scl-ChIP-Seq/Homer	1e-47	-1.089e+02	0.0000	23203.0	97.64%	23635.8	95.92%	motif file (matrix)	pdf
47	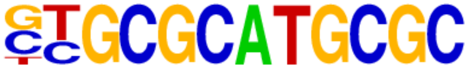	NRF1/Promoter/Homer	1e-46	-1.070e+02	0.0000	1850.0	7.79%	1362.7	5.53%	motif file (matrix)	pdf
48	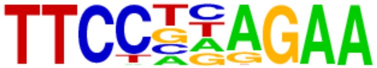	STAT6/Macrophage-Stat6-ChIP-Seq/Homer	1e-46	-1.067e+02	0.0000	8223.0	34.60%	7458.6	30.27%	motif file (matrix)	pdf
49	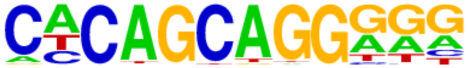	HEB?/mES-Nanog-ChIP-Seq/Homer	1e-44	-1.034e+02	0.0000	8116.0	34.15%	7368.0	29.90%	motif file (matrix)	pdf
50		STAT6(Stat)/CD4-Stat6-ChIP-Seq/Homer	1e-43	-1.013e+02	0.0000	8282.0	34.85%	7545.4	30.62%	motif file (matrix)	pdf

	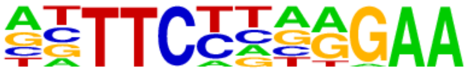										
51	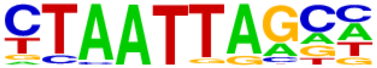	Lhx3(Homeobox)/Forebrain-p300-ChIP-Seq/Homer	1e-43	-1.004e+02	0.0000	14045.0	59.10%	13459.3	54.62%	motif file (matrix)	pdf
52	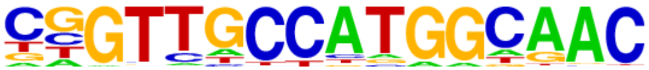	RFX(HTH)/K562-RFX3-ChIP-Seq/Homer	1e-42	-9.874e+01	0.0000	1233.0	5.19%	847.7	3.44%	motif file (matrix)	pdf
53	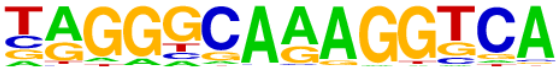	RXR(NR/DR1)/3T3L1-RXR-ChIP-Seq/Homer	1e-39	-9.101e+01	0.0000	13569.0	57.10%	13016.4	52.83%	motif file (matrix)	pdf
54	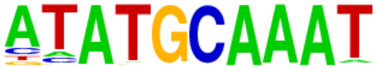	Oct2(POU/Homeobox)/Bcell-Oct2-ChIP-Seq/Homer	1e-39	-9.062e+01	0.0000	5927.0	24.94%	5264.4	21.36%	motif file (matrix)	pdf
55	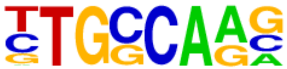	NF1-halfsite(CTF)/LNCaP-NF1-ChIP-Seq/Homer	1e-39	-9.028e+01	0.0000	17504.0	73.66%	17195.6	69.79%	motif file (matrix)	pdf
56	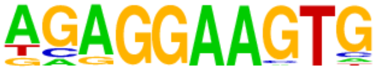	PU.1(ETS)/ThioMac-PU.1-ChIP-Seq/Homer	1e-38	-8.975e+01	0.0000	7309.0	30.76%	6634.5	26.93%	motif file (matrix)	pdf
57	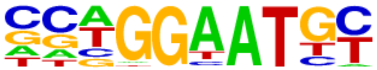	TEAD4(TEA)/Tropoblast-Tead4-ChIP-Seq (GSE37350)/Homer	1e-36	-8.312e+01	0.0000	11853.0	49.88%	11283.6	45.79%	motif file (matrix)	pdf
58	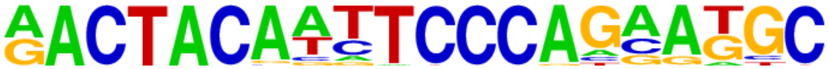	GFY-Staf/Promoters/Homer	1e-36	-8.305e+01	0.0000	933.0	3.93%	625.6	2.54%	motif file (matrix)	pdf
59	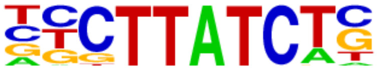	Gata2(Zf)/K562-GATA2-ChIP-Seq/Homer	1e-35	-8.196e+01	0.0000	9470.0	39.85%	8851.3	35.92%	motif file (matrix)	pdf
60	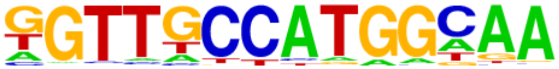	X-box(HTH)/NPC-H3K4me1-ChIP-Seq/Homer	1e-35	-8.172e+01	0.0000	1748.0	7.36%	1336.9	5.43%	motif file (matrix)	pdf
61	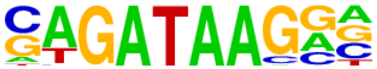	Gata1(Zf)/K562-GATA1-ChIP-Seq/Homer	1e-35	-8.171e+01	0.0000	8546.0	35.96%	7918.2	32.13%	motif file (matrix)	pdf
62	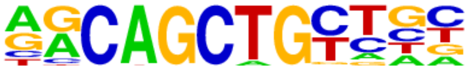	MyoD(HLH)/Myotube-MyoD-ChIP-Seq/Homer	1e-34	-7.832e+01	0.0000	9100.0	38.29%	8498.1	34.49%	motif file (matrix)	pdf
63		Rfx1(HTH)/NPC-Rfx1-ChIP-Seq/Homer	1e-33	-7.777e+01	0.0000	2997.0	12.61%	2499.3	10.14%	motif file (matrix)	pdf

	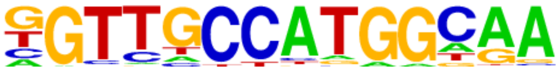										
64	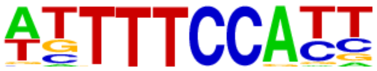	NFAT(RHD)/Jurkat-NFATC1-ChIP-Seq/Homer	1e-32	-7.499e+01	0.0000	12589.0	52.98%	12097.1	49.09%	motif file (matrix)	pdf
65	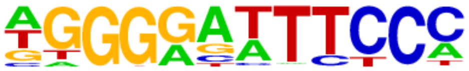	NFkB-p65(RHD)/GM12787-p65-ChIP-Seq/Homer	1e-31	-7.331e+01	0.0000	7880.0	33.16%	7298.0	29.62%	motif file (matrix)	pdf
66	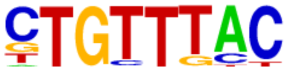	Foxo1(Forkhead)/RAW-Foxo1-ChIP-Seq/Homer	1e-31	-7.276e+01	0.0000	19235.0	80.95%	19177.0	77.83%	motif file (matrix)	pdf
67	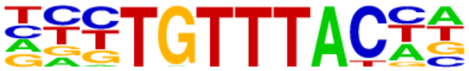	FOXP1(Forkhead)/H9-FOXP1-ChIP-Seq(GSE31006)/Homer	1e-29	-6.763e+01	0.0000	7250.0	30.51%	6701.3	27.20%	motif file (matrix)	pdf
68	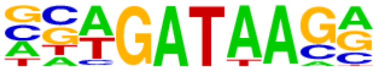	Gata4(Zf)/Heart-Gata4-ChIP-Seq(GSE35151)/Homer	1e-28	-6.601e+01	0.0000	13347.0	56.17%	12947.4	52.55%	motif file (matrix)	pdf
69	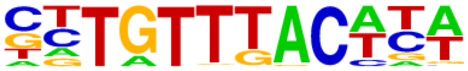	Foxa2(Forkhead)/Liver-Foxa2-ChIP-Seq/Homer	1e-28	-6.465e+01	0.0000	11066.0	46.57%	10595.6	43.00%	motif file (matrix)	pdf
70	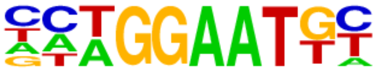	TEAD(TEA)/Fibroblast-PU.1-ChIP-Seq/Homer	1e-28	-6.463e+01	0.0000	10370.0	43.64%	9881.1	40.10%	motif file (matrix)	pdf
71	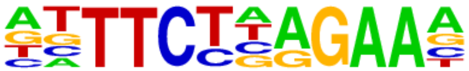	STAT5 (Stat)/mCD4+-Stat5a|b-ChIP-Seq/Homer	1e-28	-6.463e+01	0.0000	5476.0	23.04%	4956.3	20.11%	motif file (matrix)	pdf
72	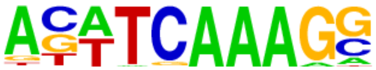	Tcf3(HMG)/mES-Tcf3-ChIP-Seq/Homer	1e-27	-6.312e+01	0.0000	4068.0	17.12%	3587.6	14.56%	motif file (matrix)	pdf
73	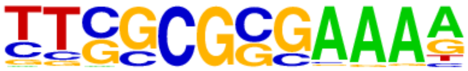	E2F(E2F)/Cell-Cycle-Exp/Homer	1e-27	-6.241e+01	0.0000	725.0	3.05%	490.6	1.99%	motif file (matrix)	pdf
74	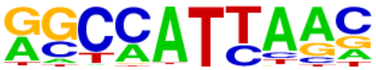	Nanog(Homeobox)/mES-Nanog-ChIP-Seq/Homer	1e-26	-6.197e+01	0.0000	23359.0	98.30%	23956.5	97.22%	motif file (matrix)	pdf
75	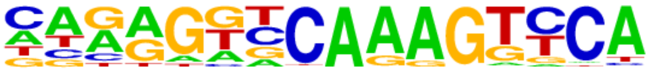	HNF4a(NR/DR1)/HepG2-HNF4a-ChIP-Seq/Homer	1e-26	-6.096e+01	0.0000	6555.0	27.58%	6048.4	24.55%	motif file (matrix)	pdf
76		EGR(Zf)/K562-EGR1-ChIP-Seq/Homer	1e-26	-5.987e+01	0.0000	2521.0	10.61%	2121.7	8.61%	motif file (matrix)	pdf

	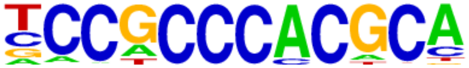										
77	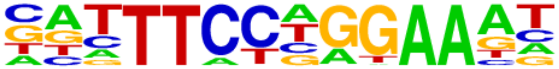	STAT1(Stat)/HelaS3-STAT1-ChIP-Seq/Homer	1e-25	-5.785e+01	0.0000	4933.0	20.76%	4460.8	18.10%	motif file (matrix)	pdf
78	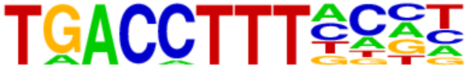	Nur77(NR)/K562-NR4A1-ChIP-Seq(GSE31363)/Homer	1e-24	-5.638e+01	0.0000	2952.0	12.42%	2545.3	10.33%	motif file (matrix)	pdf
79	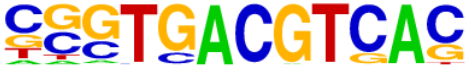	CRE(bZIP)/Promoter/Homer	1e-23	-5.439e+01	0.0000	2674.0	11.25%	2290.0	9.29%	motif file (matrix)	pdf
80	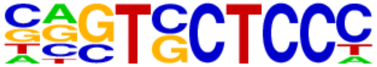	Znf263(Zf)/K562-Znf263-ChIP-Seq/Homer	1e-23	-5.439e+01	0.0000	17641.0	74.24%	17568.9	71.30%	motif file (matrix)	pdf
81	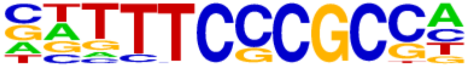	E2F7(E2F)/Hela-E2F7-ChIP-Seq(GSE32673)/Homer	1e-23	-5.408e+01	0.0000	1687.0	7.10%	1365.4	5.54%	motif file (matrix)	pdf
82	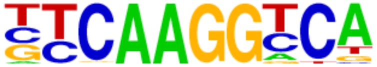	Nr5a2(NR)/mES-Nr5a2-ChIP-Seq/Homer	1e-23	-5.330e+01	0.0000	7085.0	29.82%	6629.1	26.90%	motif file (matrix)	pdf
83	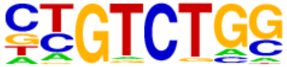	Smad2(MAD)/ES-SMAD2-ChIP-Seq(GSE29422)/Homer	1e-22	-5.265e+01	0.0000	15749.0	66.28%	15568.7	63.18%	motif file (matrix)	pdf
84	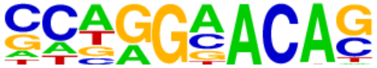	AR-halfsite(NR)/LNCaP-AR-ChIP-Seq/Homer	1e-22	-5.166e+01	0.0000	22739.0	95.69%	23227.6	94.27%	motif file (matrix)	pdf
85	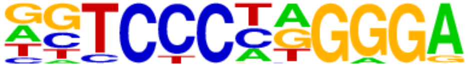	EBF(EBF)/proBcell-EBF-ChIP-Seq/Homer	1e-21	-4.855e+01	0.0000	3325.0	13.99%	2942.1	11.94%	motif file (matrix)	pdf
86	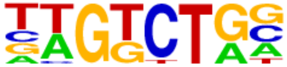	Smad3(MAD)/NPC-Smad3-ChIP-Seq(GSE36673)/Homer	1e-20	-4.827e+01	0.0000	21491.0	90.44%	21813.0	88.53%	motif file (matrix)	pdf
87	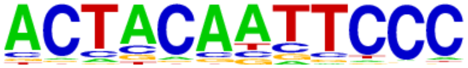	GFY(?)/Promoter/Homer	1e-20	-4.824e+01	0.0000	1153.0	4.85%	897.2	3.64%	motif file (matrix)	pdf
88	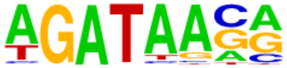	GATA3(Zf)/iTreg-Gata3-ChIP-Seq(GSE20898)/Homer	1e-20	-4.809e+01	0.0000	17237.0	72.54%	17183.5	69.74%	motif file (matrix)	pdf
89		JunD(bZIP)/K562-JunD-ChIP-Seq/Homer	1e-20	-4.783e+01	0.0000	1135.0	4.78%	882.5	3.58%	motif file (matrix)	pdf

	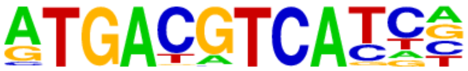										
90	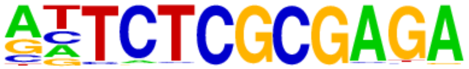	GFX(?)/Promoter/Homer	1e-20	-4.746e+01	0.0000	228.0	0.96%	118.6	0.48%	motif file (matrix)	pdf
91	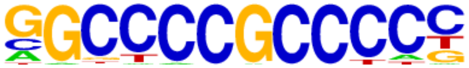	Sp1(Zf)/Promoter/Homer	1e-20	-4.736e+01	0.0000	4076.0	17.15%	3680.4	14.94%	motif file (matrix)	pdf
92	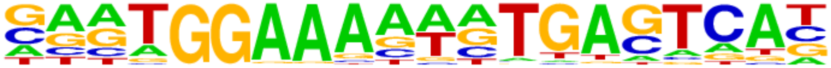	NFAT:AP1/Jurkat-NFATC1-ChIP-Seq/Homer	1e-19	-4.401e+01	0.0000	3111.0	13.09%	2759.7	11.20%	motif file (matrix)	pdf
93	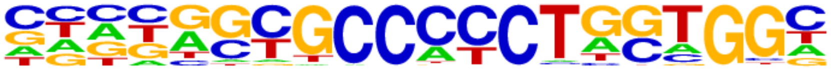	BORIS(Zf)/K562-CTCFL-ChIP-Seq/Homer	1e-18	-4.327e+01	0.0000	2607.0	10.97%	2277.2	9.24%	motif file (matrix)	pdf
94	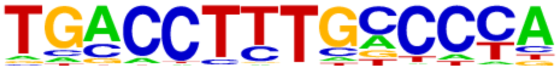	PPARE(NR/DR1)/3T3L1-Pparg-ChIP-Seq/Homer	1e-18	-4.295e+01	0.0000	12130.0	51.05%	11864.8	48.15%	motif file (matrix)	pdf
95	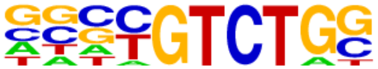	Smad4(MAD)/ESC-SMAD4-ChIP-Seq(GSE29422)/Homer	1e-18	-4.218e+01	0.0000	15832.0	66.62%	15741.7	63.89%	motif file (matrix)	pdf
96	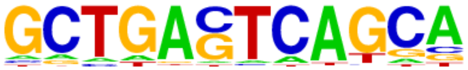	MafK(bZIP)/C2C12-MafK-ChIP-Seq(GSE36030)/Homer	1e-18	-4.200e+01	0.0000	3283.0	13.82%	2937.8	11.92%	motif file (matrix)	pdf
97	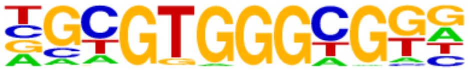	Egr2/Thymocytes-Egr2-ChIP-Seq(GSE34254)/Homer	1e-18	-4.172e+01	0.0000	2210.0	9.30%	1905.3	7.73%	motif file (matrix)	pdf
98	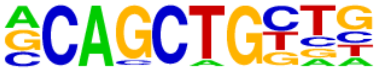	Tcf12(HLH)/GM12878-Tcf12-ChIP-Seq/Homer	1e-17	-3.956e+01	0.0000	10997.0	46.28%	10724.3	43.52%	motif file (matrix)	pdf
99	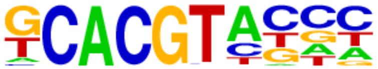	HIF2a(HLH)/O785-HIF2a-ChIP-Seq(GSE34871)/Homer	1e-16	-3.913e+01	0.0000	4669.0	19.65%	4317.4	17.52%	motif file (matrix)	pdf
100	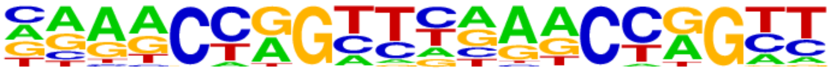	Tcfcp2l1(CP2)/mES-Tcfcp2l1-ChIP-Seq/Homer	1e-16	-3.878e+01	0.0000	1902.0	8.00%	1626.6	6.60%	motif file (matrix)	pdf
101	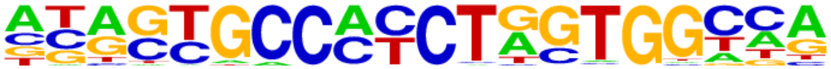	CTCF(Zf)/CD4+-CTCF-ChIP-Seq/Homer	1e-16	-3.877e+01	0.0000	1608.0	6.77%	1349.7	5.48%	motif file (matrix)	pdf
102		Hoxb4/ES-Hoxb4-ChIP-Seq (GSE34014)/Homer	1e-16	-3.852e+01	0.0000	3026.0	12.73%	2707.7	10.99%	motif file (matrix)	pdf

	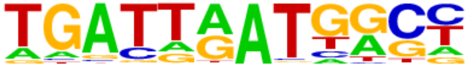										
103	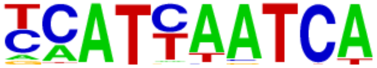	Pdx1(Homeobox)/Islet-Pdx1-ChIP-Seq/Homer	1e-16	-3.769e+01	0.0000	12506.0	52.63%	12302.7	49.93%	motif file (matrix)	pdf
104	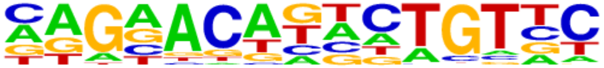	PR(NR)/T47D-PR-ChIP-Seq (GSE31130)/Homer	1e-16	-3.687e+01	0.0000	19440.0	81.81%	19635.3	79.69%	motif file (matrix)	pdf
105	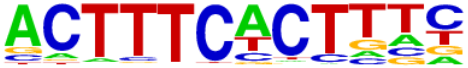	PRDM1/BMI1(Zf)/Hela-PRDM1-ChIP-Seq (GSE31477)/Homer	1e-15	-3.597e+01	0.0000	9034.0	38.02%	8743.0	35.48%	motif file (matrix)	pdf
106	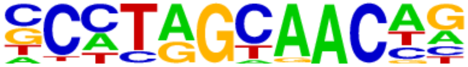	Rfx5(HTH)/GM12878-Rfx5-ChIP-Seq(GSE31477)/Homer	1e-14	-3.335e+01	0.0000	4657.0	19.60%	4347.2	17.64%	motif file (matrix)	pdf
107	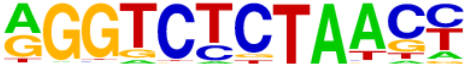	PRDM14(Zf)/H1-PRDM14-ChIP-Seq/Homer	1e-13	-3.150e+01	0.0000	4491.0	18.90%	4196.6	17.03%	motif file (matrix)	pdf
108	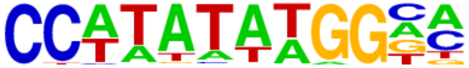	CArG(MADS)/PUER-Srf-ChIP-Seq/Homer	1e-13	-3.121e+01	0.0000	4869.0	20.49%	4575.7	18.57%	motif file (matrix)	pdf
109	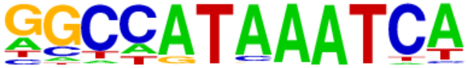	Hoxc9/Ainv15-Hoxc9-ChIP-Seq/Homer	1e-13	-3.005e+01	0.0000	8059.0	33.91%	7805.0	31.68%	motif file (matrix)	pdf
110	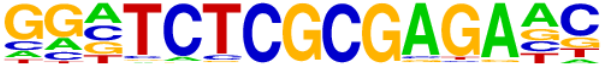	ZBTB33/GM12878-ZBTB33-ChIP-Seq/Homer	1e-12	-2.956e+01	0.0000	492.0	2.07%	361.2	1.47%	motif file (matrix)	pdf
111	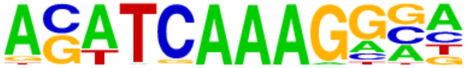	Tcf4(HMG)/Hct116-Tcf4-ChIP-Seq/Homer	1e-12	-2.872e+01	0.0000	6774.0	28.51%	6514.0	26.44%	motif file (matrix)	pdf
112	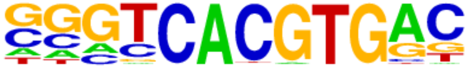	ATF3(bZIP)/K562-ATF3-ChIP-Seq/Homer	1e-12	-2.866e+01	0.0000	2166.0	9.12%	1930.6	7.83%	motif file (matrix)	pdf
113	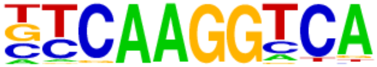	Nr5a2(NR)/Pancreas-LRH1-ChIP-Seq(GSE34295)/Homer	1e-12	-2.864e+01	0.0000	9206.0	38.74%	8991.1	36.49%	motif file (matrix)	pdf
114	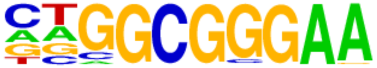	E2F1(E2F)/Hela-E2F1-ChIP-Seq/Hoemr	1e-12	-2.844e+01	0.0000	3188.0	13.42%	2929.8	11.89%	motif file (matrix)	pdf
115		NFY (CCAAT)/Promoter/Homer	1e-12	-2.799e+01	0.0000	9759.0	41.07%	9566.0	38.82%	motif file (matrix)	pdf

	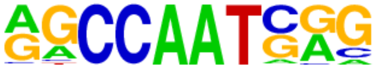										
116	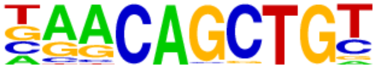	Myf5(bHLH)/GM-Myf5-ChIP-Seq(GSE24852)/Homer	1e-12	-2.770e+01	0.0000	8322.0	35.02%	8097.6	32.86%	motif file (matrix)	pdf
117	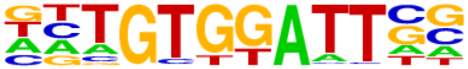	Foxh1(Forkhead)/hESC-FOXH1-ChIP-Seq (GSE29422)/Homer	1e-11	-2.673e+01	0.0000	9150.0	38.51%	8954.3	36.34%	motif file (matrix)	pdf
118	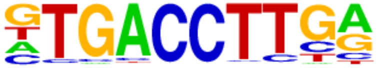	Esrrb(NR)/mES-Esrrb-ChIP-Seq/Homer	1e-11	-2.630e+01	0.0000	8957.0	37.69%	8761.8	35.56%	motif file (matrix)	pdf
119	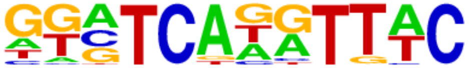	Six1(Homeobox)/Myoblast-Six1-ChIP-Chip (GSE20150)/Homer	1e-11	-2.625e+01	0.0000	3845.0	16.18%	3595.2	14.59%	motif file (matrix)	pdf
120	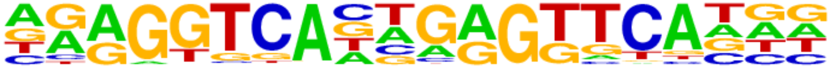	VDR(NR/DR3)/GM10855-VDR+vitD-ChIP-Seq/Homer	1e-11	-2.601e+01	0.0000	3214.0	13.53%	2972.3	12.06%	motif file (matrix)	pdf
121	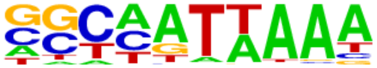	Unknown/Homeobox/Limb-p300-ChIP-Seq/Homer	1e-10	-2.510e+01	0.0000	10355.0	43.58%	10210.5	41.44%	motif file (matrix)	pdf
122	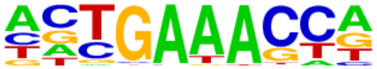	IRF4(IRF)/GM12878-IRF4-ChIP-Seq/Homer	1e-10	-2.489e+01	0.0000	6773.0	28.50%	6550.9	26.59%	motif file (matrix)	pdf
123	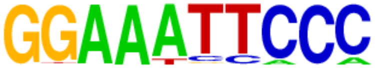	NFkB-p65-Rel(RHD)/LPS-exp/Homer	1e-10	-2.481e+01	0.0000	979.0	4.12%	818.5	3.32%	motif file (matrix)	pdf
124	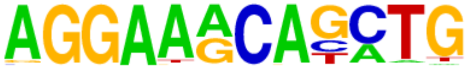	ETS:E-box/HPC7-Scl-ChIP-Seq/Homer	1e-10	-2.335e+01	0.0000	1434.0	6.03%	1255.8	5.10%	motif file (matrix)	pdf
125	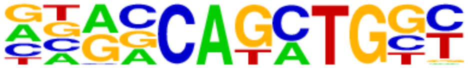	Atoh1(bHLH)/Cerebellum-Atoh1-ChIP-Seq/Homer	1e-9	-2.283e+01	0.0000	12396.0	52.17%	12347.3	50.11%	motif file (matrix)	pdf
126	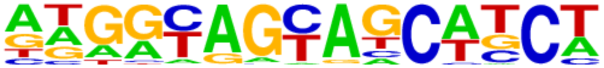	PRDM9(Zf)/Testis-DMC1-ChIP-Seq(GSE35498)/Homer	1e-7	-1.778e+01	0.0000	5538.0	23.31%	5376.0	21.82%	motif file (matrix)	pdf
127	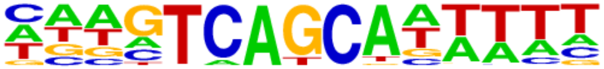	MafF(bZIP)/HepG2-MafF-ChIP-Seq(GSE31477)/Homer	1e-7	-1.705e+01	0.0000	5257.0	22.12%	5100.6	20.70%	motif file (matrix)	pdf
128		PAX3:FKHR-fusion (Paired/Homeobox)/Rh4-PAX3:FKHR-ChIP-Seq/Homer	1e-7	-1.672e+01	0.0000	3263.0	13.73%	3098.6	12.58%	motif file (matrix)	pdf

	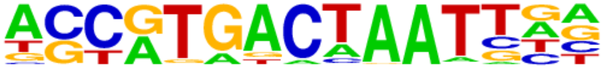										
129	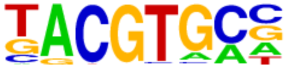	HIF-1a(HLH)/MCF7-HIF1a-ChIP-Seq/Homer	1e-7	-1.671e+01	0.0000	3417.0	14.38%	3252.8	13.20%	motif file (matrix)	pdf
130	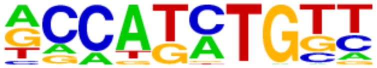	Olig2(bHLH)/Neuron-Olig2-ChIP-Seq(GSE30882)/Homer	1e-7	-1.623e+01	0.0000	18323.0	77.11%	18643.5	75.66%	motif file (matrix)	pdf
131	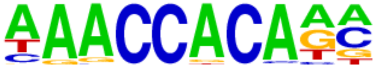	RUNX1(Runt)/Jurkat-RUNX1-ChIP-Seq/Homer	1e-7	-1.618e+01	0.0000	12353.0	51.98%	12392.7	50.29%	motif file (matrix)	pdf
132	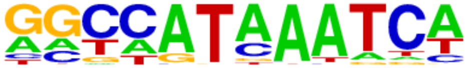	HOXA9/HSC-Hoxa9-ChIP-Seq (GSE33509)/Homer	1e-6	-1.489e+01	0.0000	9734.0	40.96%	9704.3	39.38%	motif file (matrix)	pdf
133	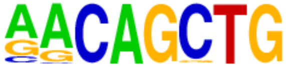	MyoG(HLH)/C2C12-MyoG-ChIP-Seq(GSE36024)/Homer	1e-6	-1.445e+01	0.0000	11546.0	48.59%	11582.4	47.01%	motif file (matrix)	pdf
134	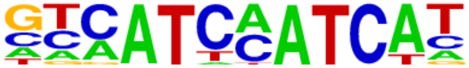	HOXA2(Homeobox)/mES-Hoxa2-ChIP-Seq/Homer	1e-6	-1.444e+01	0.0000	1525.0	6.42%	1397.4	5.67%	motif file (matrix)	pdf
135	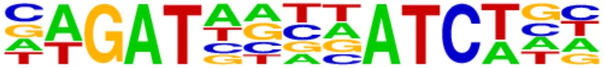	GATA-IR4(Zf)/iTreg-Gata3-ChIP-Seq(GSE20898)/Homer	1e-5	-1.220e+01	0.0000	1260.0	5.30%	1154.1	4.68%	motif file (matrix)	pdf
136	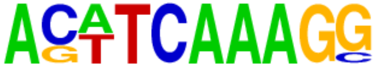	TCFL2(HMG)/K562-TCF7L2-ChIP-Seq(GSE29196)/Homer	1e-5	-1.198e+01	0.0000	1242.0	5.23%	1138.5	4.62%	motif file (matrix)	pdf
137	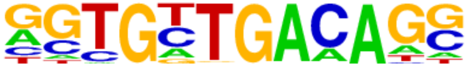	Tbx20(T-box)/Heart-Tbx20-ChIP-Seq(GSE29636)/Homer	1e-4	-1.107e+01	0.0000	2813.0	11.84%	2706.3	10.98%	motif file (matrix)	pdf
138	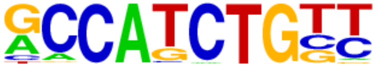	NeuroD1(bHLH)/Islet-NeuroD1-ChIP-Seq (GSE30298)/Homer	1e-4	-1.093e+01	0.0000	10114.0	42.56%	10161.3	41.24%	motif file (matrix)	pdf
139	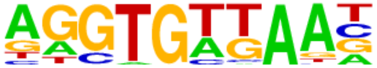	Eomes(T-box)/H9-Eomes-ChIP-Seq/Homer	1e-4	-1.051e+01	0.0000	22746.0	95.72%	23449.9	95.17%	motif file (matrix)	pdf
140	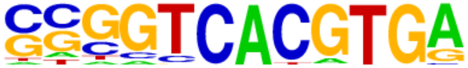	E-box(HLH)/Promoter/Homer	1e-4	-1.007e+01	0.0001	971.0	4.09%	887.4	3.60%	motif file (matrix)	pdf
141		GATA:SCL/Ter119-SCL-ChIP-Seq/Homer	1e-4	-9.642e+00	0.0001	1713.0	7.21%	1622.7	6.59%	motif file (matrix)	pdf

	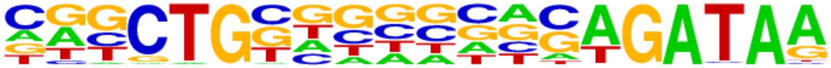										
142	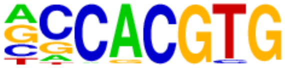	c-Myc(HLH)/LNCAP-cMyc-ChIP-Seq/Homer	1e-4	-9.430e+00	0.0001	5048.0	21.24%	4990.0	20.25%	motif file (matrix)	pdf
143	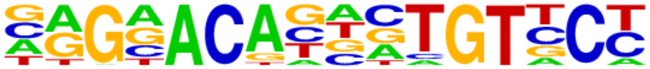	GRE(NR/IR3)/A549-GR-ChIP-Seq/Homer	1e-3	-8.980e+00	0.0002	1613.0	6.79%	1530.0	6.21%	motif file (matrix)	pdf
144	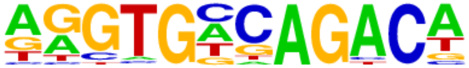	Tbox:Smad/ESCd5-Smad2_3-ChIP-Seq (GSE29422)/Homer	1e-3	-8.267e+00	0.0004	2783.0	11.71%	2710.8	11.00%	motif file (matrix)	pdf
145	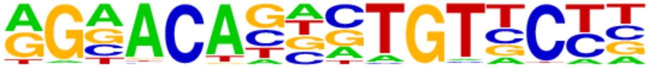	ARE(NR)/LNCAP-AR-ChIP-Seq/Homer	1e-3	-8.043e+00	0.0005	3075.0	12.94%	3008.1	12.21%	motif file (matrix)	pdf
146	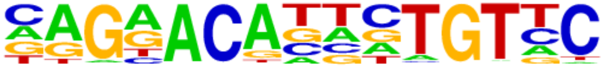	GRE/RAW264.7-GRE-ChIP-Seq/Homer	1e-3	-7.589e+00	0.0007	2945.0	12.39%	2883.4	11.70%	motif file (matrix)	pdf
147	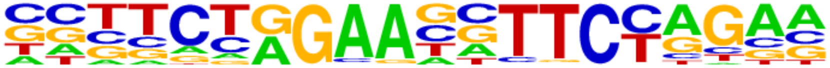	HRE(HSF)/HepG2-HSF1-ChIP-Seq/Homer	1e-3	-7.322e+00	0.0010	2021.0	8.50%	1955.4	7.94%	motif file (matrix)	pdf
148	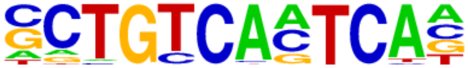	Pbx3(Homeobox)/GM12878-PBX3-ChIP-Seq/Homer	1e-3	-7.278e+00	0.0010	3065.0	12.90%	3009.4	12.21%	motif file (matrix)	pdf
149	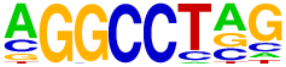	ZNF711(Zf)/SH-SY5Y-ZNF711-ChIP-Seq/Homer	1e-3	-7.047e+00	0.0013	17434.0	73.37%	17854.7	72.46%	motif file (matrix)	pdf
150	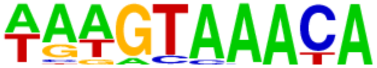	FOXA1(Forkhead)/LNCAP-FOXA1-ChIP-Seq/Homer	1e-2	-6.716e+00	0.0017	15275.0	64.28%	15605.9	63.33%	motif file (matrix)	pdf
151	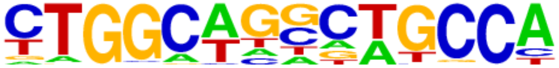	Tlx?/NPC-H3K4me1-ChIP-Seq/Homer	1e-2	-6.574e+00	0.0020	5555.0	23.38%	5559.9	22.56%	motif file (matrix)	pdf
152	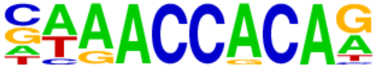	RUNX(Runt)/HPC7-Runx1-ChIP-Seq/Homer	1e-2	-6.417e+00	0.0023	8830.0	37.16%	8929.5	36.24%	motif file (matrix)	pdf
153	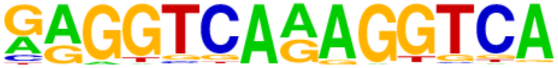	TR4(NR/DR1)/Hela-TR4-ChIP-Seq/Homer	1e-2	-6.188e+00	0.0029	1529.0	6.43%	1475.9	5.99%	motif file (matrix)	pdf
154		GLI3(Zf)/GLI3-ChIP-Chip/Homer	1e-2	-5.898e+00	0.0038	1816.0	7.64%	1767.4	7.17%	motif file (matrix)	pdf

	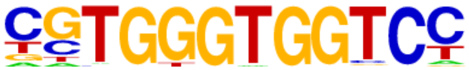										
155	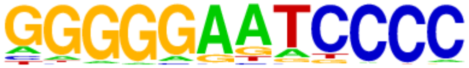	NFkB-p50,p52(RHD)/p50-ChIP-Chip/Homer	1e-2	-5.772e+00	0.0043	1716.0	7.22%	1668.6	6.77%	motif file (matrix)	pdf
156	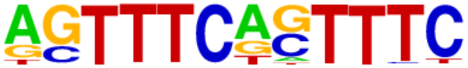	ISRE(IRF)/ThioMac-LPS-exp/HOMER	1e-2	-5.755e+00	0.0044	1069.0	4.50%	1020.6	4.14%	motif file (matrix)	pdf
157	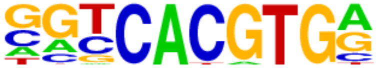	USF1(HLH)/GM12878-Usf1-ChIP-Seq/Homer	1e-2	-5.552e+00	0.0053	5523.0	23.24%	5549.0	22.52%	motif file (matrix)	pdf
158	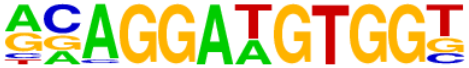	ETS:RUNX/Jurkat-RUNX1-ChIP-Seq/Homer	1e-2	-5.113e+00	0.0082	1450.0	6.10%	1409.7	5.72%	motif file (matrix)	pdf

Results homer motif enrichments (known motifs)

TCam-2 h3k4me3

Homer Known Motif Enrichment Results

Homer *de novo* Motif Results
Gene Ontology Enrichment Results
Known Motif Enrichment Results (txt file)
Total Target Sequences = 29429, Total Background Sequences = 25640

Rank	Motif	Name	P-value	log P-pvalue	q-value (Benjamini)	# Target Sequences with Motif	% of Targets Sequences with Motif	# Background Sequences with Motif	% of Background Sequences with Motif	Motif File	PDF
1	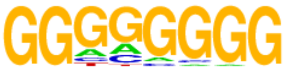	Maz(Zf)/HepG2-Maz-ChIP-Seq (GSE31477)/Homer	1e-416	-9.582e+02	0.0000	23161.0	78.70%	17229.8	67.20%	motif file (matrix)	pdf
2	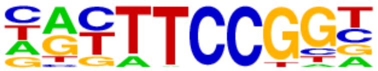	Elk4(ETS)/Hela-Elk4-ChIP-Seq (GSE31477)/Homer	1e-177	-4.083e+02	0.0000	12630.0	42.92%	8948.2	34.90%	motif file (matrix)	pdf
3	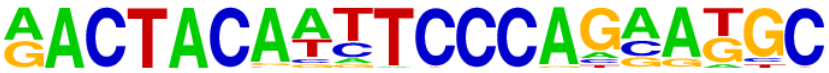	GFY-Staf/Promoters/Homer	1e-162	-3.733e+02	0.0000	1975.0	6.71%	886.1	3.46%	motif file (matrix)	pdf
4	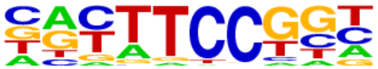	Fli1(ETS)/CD8-FLI-ChIP-Seq (GSE20898)/Homer	1e-156	-3.595e+02	0.0000	18676.0	63.46%	14309.3	55.81%	motif file (matrix)	pdf
5	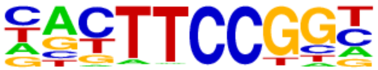	Elk1(ETS)/Hela-Elk1-ChIP-Seq (GSE31477)/Homer	1e-155	-3.577e+02	0.0000	12387.0	42.09%	8874.2	34.61%	motif file (matrix)	pdf
6	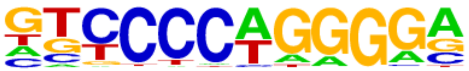	EBF1(EBF)/Near-E2A-ChIP-Seq/Homer	1e-136	-3.154e+02	0.0000	19273.0	65.49%	14973.0	58.39%	motif file (matrix)	pdf
7	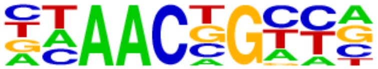	BMYB(HTH)/Hela-BMYB-ChIPSeq(GSE27030)/Homer	1e-124	-2.870e+02	0.0000	19924.0	67.70%	15646.5	61.02%	motif file (matrix)	pdf
8	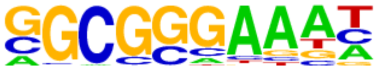	E2F4(E2F)/K562-E2F4-ChIP-Seq(GSE31477)/Homer	1e-121	-2.804e+02	0.0000	11943.0	40.58%	8718.4	34.00%	motif file (matrix)	pdf
9	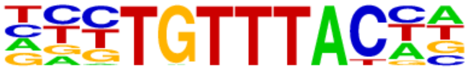	FOXP1(Forkhead)/H9-FOXP1-ChIP-Seq(GSE31006)/Homer	1e-114	-2.632e+02	0.0000	7775.0	26.42%	5350.5	20.87%	motif file (matrix)	pdf
10	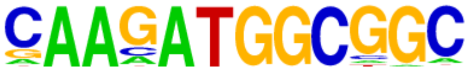	YY1(Zf)/Promoter/Homer	1e-111	-2.566e+02	0.0000	2388.0	8.11%	1285.9	5.02%	motif file (matrix)	pdf
11		Foxa2(Forkhead)/Liver-Foxa2-ChIP-Seq/Homer	1e-108	-2.500e+02	0.0000	11804.0	40.11%	8694.4	33.91%	motif file (matrix)	pdf

	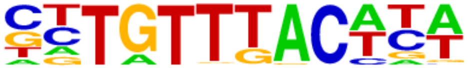										
12	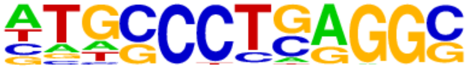	AP-2alpha(AP2)/Hela-AP2alpha-ChIP-Seq/Homer	1e-106	-2.441e+02	0.0000	17620.0	59.87%	13725.7	53.53%	motif file (matrix)	pdf
13	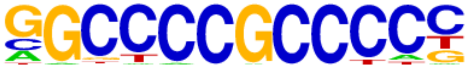	Sp1(Zf)/Promoter/Homer	1e-93	-2.157e+02	0.0000	10239.0	34.79%	7498.9	29.25%	motif file (matrix)	pdf
14	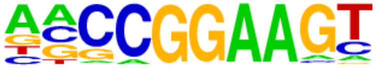	ELF1(ETS)/Jurkat-ELF1-ChIP-Seq/Homer	1e-93	-2.153e+02	0.0000	11147.0	37.88%	8256.3	32.20%	motif file (matrix)	pdf
15	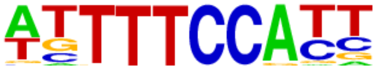	NFAT(RHD)/Jurkat-NFATC1-ChIP-Seq/Homer	1e-91	-2.102e+02	0.0000	13920.0	47.30%	10624.9	41.44%	motif file (matrix)	pdf
16	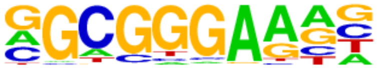	E2F6(E2F)/Hela-E2F6-ChIP-Seq(GSE31477)/Homer	1e-82	-1.903e+02	0.0000	13440.0	45.67%	10287.0	40.12%	motif file (matrix)	pdf
17	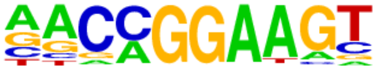	ETV1(ETS)/GIST48-ETV1-ChIP-Seq/Homer	1e-82	-1.896e+02	0.0000	20124.0	68.38%	16158.8	63.02%	motif file (matrix)	pdf
18	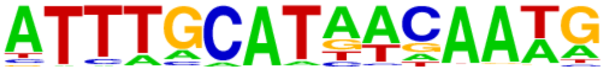	OCT4-SOX2-TCF-NANOG ((POU/Homeobox/HMG)/mES-ChIP-Seq/Homer	1e-81	-1.883e+02	0.0000	3732.0	12.68%	2377.7	9.27%	motif file (matrix)	pdf
19	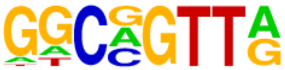	MYB(HTH)/ERMYB-Myb-ChIPSeq(GSE22095)/Homer	1e-80	-1.843e+02	0.0000	21495.0	73.04%	17423.6	67.95%	motif file (matrix)	pdf
20	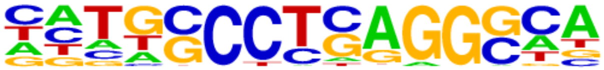	AP2gamma(AP2)/MCF7-TFAP2c-ChIP-Seq/Homer	1e-78	-1.818e+02	0.0000	17417.0	59.19%	13774.1	53.72%	motif file (matrix)	pdf
21	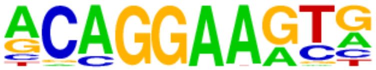	ERG(ETS)/VCaP-ERG-ChIP-Seq/Homer	1e-77	-1.779e+02	0.0000	21428.0	72.82%	17386.8	67.81%	motif file (matrix)	pdf
22	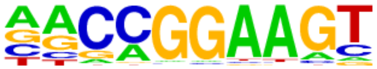	GABPA(ETS)/Jurkat-GABPa-ChIP-Seq/Homer	1e-77	-1.777e+02	0.0000	15246.0	51.81%	11891.0	46.38%	motif file (matrix)	pdf
23	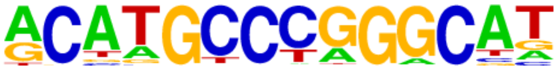	p53(p53)/mES-cMyc-ChIP-Seq/Homer	1e-76	-1.769e+02	0.0000	917.0	3.12%	406.4	1.59%	motif file (matrix)	pdf
24		NFkB-p65(RHD)/GM12787-p65-ChIP-Seq/Homer	1e-74	-1.727e+02	0.0000	10622.0	36.09%	7966.9	31.07%	motif file (matrix)	pdf

	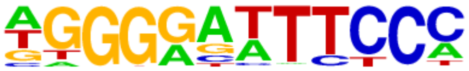										
25	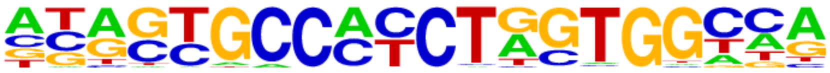	CTCF(Zf)/CD4+-CTCF-ChIP-Seq/Homer	1e-74	-1.724e+02	0.0000	3103.0	10.54%	1938.3	7.56%	motif file (matrix)	pdf
26	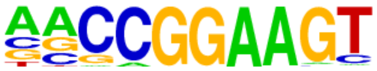	ETS(ETS)/Promoter/Homer	1e-74	-1.721e+02	0.0000	7104.0	24.14%	5069.8	19.77%	motif file (matrix)	pdf
27	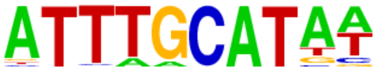	Oct4(POU/Homeobox)/mES-Oct4-ChIP-Seq/Homer	1e-73	-1.691e+02	0.0000	7927.0	26.94%	5748.6	22.42%	motif file (matrix)	pdf
28	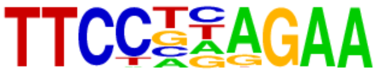	STAT6/Macrophage-Stat6-ChIP-Seq/Homer	1e-71	-1.650e+02	0.0000	8713.0	29.61%	6407.2	24.99%	motif file (matrix)	pdf
29	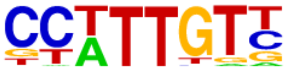	Sox3(HMG)/NPC-Sox3-ChIP-Seq(GSE33059)/Homer	1e-65	-1.511e+02	0.0000	19941.0	67.76%	16147.9	62.98%	motif file (matrix)	pdf
30	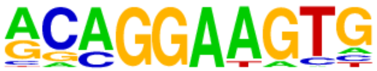	ETS1(ETS)/Jurkat-ETS1-ChIP-Seq/Homer	1e-64	-1.497e+02	0.0000	16495.0	56.05%	13097.5	51.08%	motif file (matrix)	pdf
31	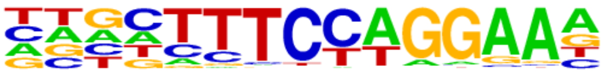	Bcl6(Zf)/Liver-Bcl6-ChIP-Seq (GSE31578)/Homer	1e-61	-1.422e+02	0.0000	18731.0	63.65%	15104.4	58.91%	motif file (matrix)	pdf
32	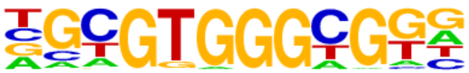	Egr2/Thymocytes-Egr2-ChIP-Seq(GSE34254)/Homer	1e-57	-1.313e+02	0.0000	5701.0	19.37%	4070.5	15.87%	motif file (matrix)	pdf
33	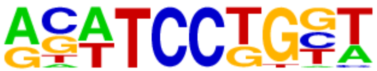	SPDEF(ETS)/VCaP-SPDEF-ChIP-Seq/Homer	1e-56	-1.305e+02	0.0000	15683.0	53.29%	12474.3	48.65%	motif file (matrix)	pdf
34	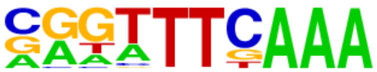	CHR/Cell-Cycle-Exp/Homer	1e-55	-1.277e+02	0.0000	10432.0	35.45%	7986.2	31.15%	motif file (matrix)	pdf
35	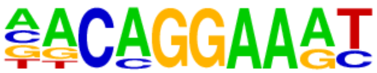	EWS:FLI1-fusion (ETS)/SK_N_MC-EWS:FLI1-ChIP-Seq/Homer	1e-52	-1.219e+02	0.0000	10174.0	34.57%	7794.1	30.40%	motif file (matrix)	pdf
36	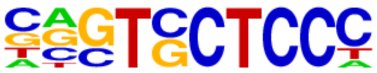	Znf263(Zf)/K562-Znf263-ChIP-Seq/Homer	1e-52	-1.212e+02	0.0000	24243.0	82.38%	20206.8	78.81%	motif file (matrix)	pdf
37		EWS:ERG-fusion (ETS)/CADO_ES1-EWS:ERG-ChIP-Seq/Homer	1e-51	-1.180e+02	0.0000	11640.0	39.55%	9052.8	35.31%	motif file (matrix)	pdf

	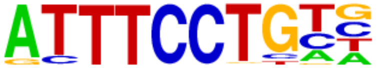										
38	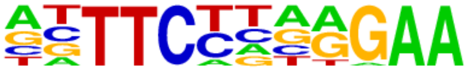	STAT6(Stat)/CD4-Stat6-ChIP-Seq/Homer	1e-51	-1.180e+02	0.0000	8660.0	29.43%	6544.8	25.53%	motif file (matrix)	pdf
39	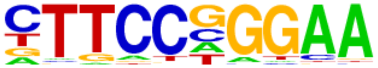	Stat3(Stat)/mES-Stat3-ChIP-Seq/Homer	1e-50	-1.169e+02	0.0000	9397.0	31.93%	7164.0	27.94%	motif file (matrix)	pdf
40	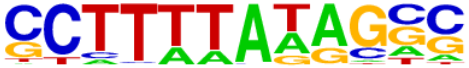	TATA-Box (TBP)/Promoter/Homer	1e-50	-1.152e+02	0.0000	17445.0	59.28%	14092.5	54.96%	motif file (matrix)	pdf
41	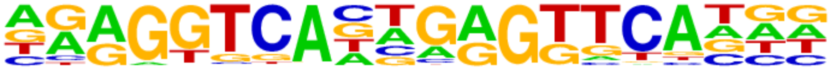	VDR(NR/DR3)/GM10855-VDR+vitD-ChIP-Seq/Homer	1e-49	-1.137e+02	0.0000	4527.0	15.38%	3189.5	12.44%	motif file (matrix)	pdf
42	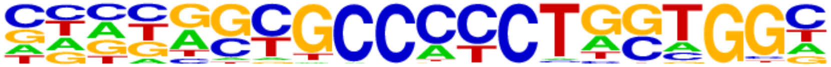	BORIS(Zf)/K562-CTCFL-ChIP-Seq/Homer	1e-43	-1.006e+02	0.0000	5897.0	20.04%	4339.6	16.92%	motif file (matrix)	pdf
43	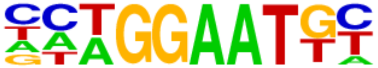	TEAD(TEA)/Fibroblast-PU.1-ChIP-Seq/Homer	1e-42	-9.810e+01	0.0000	10819.0	36.76%	8451.5	32.96%	motif file (matrix)	pdf
44	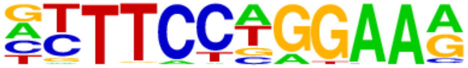	STAT4(Stat)/CD4-Stat4-ChIP-Seq/Homer	1e-42	-9.744e+01	0.0000	14043.0	47.72%	11216.4	43.74%	motif file (matrix)	pdf
45	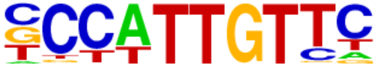	Sox2(HMG)/mES-Sox2-ChIP-Seq/Homer	1e-42	-9.710e+01	0.0000	12449.0	42.30%	9846.0	38.40%	motif file (matrix)	pdf
46	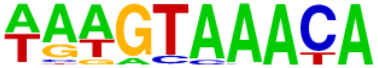	FOXA1(Forkhead)/LNCAP-FOXA1-ChIP-Seq/Homer	1e-41	-9.491e+01	0.0000	15854.0	53.87%	12803.4	49.93%	motif file (matrix)	pdf
47	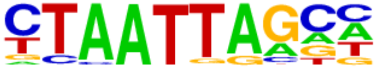	Lhx3(Homeobox)/Forebrain-p300-ChIP-Seq/Homer	1e-40	-9.417e+01	0.0000	13857.0	47.09%	11073.6	43.19%	motif file (matrix)	pdf
48	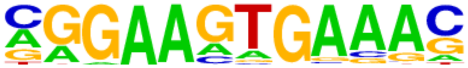	PU.1-IRF/Bcell-PU.1-ChIP-Seq/Homer	1e-39	-9.072e+01	0.0000	19245.0	65.40%	15814.4	61.68%	motif file (matrix)	pdf
49	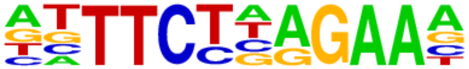	STAT5 (Stat)/mCD4+-Stat5a|b-ChIP-Seq/Homer	1e-38	-8.832e+01	0.0000	5796.0	19.70%	4306.2	16.79%	motif file (matrix)	pdf
50		Tbox:Smad/ESCd5-Smad2_3-ChIP-Seq (GSE29422)/Homer	1e-37	-8.683e+01	0.0000	3735.0	12.69%	2648.2	10.33%	motif file (matrix)	pdf

	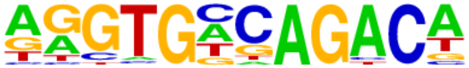										
51	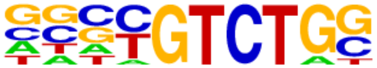	Smad4(MAD)/ESC-SMAD4-ChIP-Seq(GSE29422)/Homer	1e-37	-8.630e+01	0.0000	21122.0	71.78%	17517.9	68.32%	motif file (matrix)	pdf
52	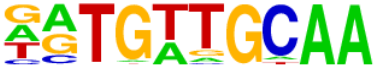	CEBP:AP1/ThioMac-CEBPb-ChIP-Seq/Homer	1e-37	-8.536e+01	0.0000	10716.0	36.41%	8430.0	32.88%	motif file (matrix)	pdf
53	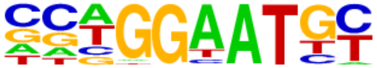	TEAD4(TEA)/Tropoblast-Tead4-ChIP-Seq (GSE37350)/Homer	1e-35	-8.276e+01	0.0000	13624.0	46.30%	10936.9	42.65%	motif file (matrix)	pdf
54	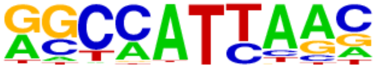	Nanog(Homeobox)/mES-Nanog-ChIP-Seq/Homer	1e-35	-8.133e+01	0.0000	28165.0	95.71%	24119.7	94.07%	motif file (matrix)	pdf
55	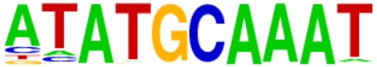	Oct2(POU/Homeobox)/Bcell-Oct2-ChIP-Seq/Homer	1e-35	-8.083e+01	0.0000	5345.0	18.16%	3969.8	15.48%	motif file (matrix)	pdf
56	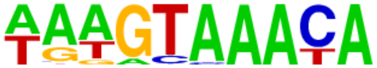	FOXA1(Forkhead)/MCF7-FOXA1-ChIP-Seq/Homer	1e-33	-7.633e+01	0.0000	14151.0	48.09%	11430.9	44.58%	motif file (matrix)	pdf
57	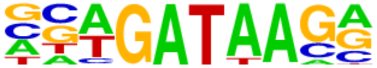	Gata4(Zf)/Heart-Gata4-ChIP-Seq(GSE35151)/Homer	1e-33	-7.606e+01	0.0000	13432.0	45.64%	10810.3	42.16%	motif file (matrix)	pdf
58	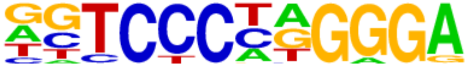	EBF(EBF)/proBcell-EBF-ChIP-Seq/Homer	1e-32	-7.411e+01	0.0000	5434.0	18.47%	4071.6	15.88%	motif file (matrix)	pdf
59	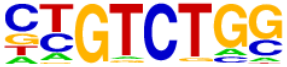	Smad2(MAD)/ES-SMAD2-ChIP-Seq(GSE29422)/Homer	1e-32	-7.385e+01	0.0000	20891.0	70.99%	17380.2	67.78%	motif file (matrix)	pdf
60	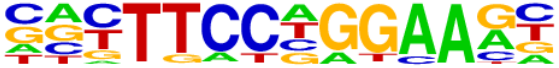	Stat3+il23(Stat)/CD4-Stat3-ChIP-Seq/Homer	1e-31	-7.211e+01	0.0000	11590.0	39.38%	9251.2	36.08%	motif file (matrix)	pdf
61	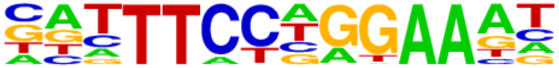	STAT1(Stat)/HelaS3-STAT1-ChIP-Seq/Homer	1e-31	-7.160e+01	0.0000	5203.0	17.68%	3893.2	15.18%	motif file (matrix)	pdf
62	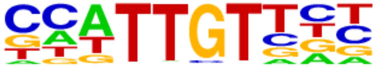	Sox6(HMG)/Myotubes-Sox6-ChIP-Seq(GSE32627)/Homer	1e-30	-7.019e+01	0.0000	18650.0	63.38%	15408.0	60.09%	motif file (matrix)	pdf
63		Foxo1(Forkhead)/RAW-Foxo1-ChIP-Seq/Homer	1e-30	-7.004e+01	0.0000	21812.0	74.12%	18230.9	71.10%	motif file (matrix)	pdf

	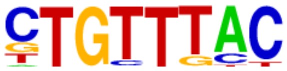										
64	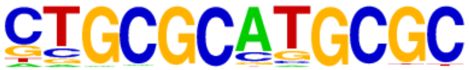	NRF1(NRF)/MCF7-NRF1-ChIP-Seq/Homer	1e-30	-6.934e+01	0.0000	3859.0	13.11%	2811.6	10.97%	motif file (matrix)	pdf
65	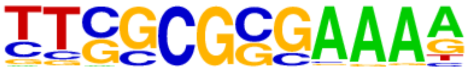	E2F(E2F)/Cell-Cycle-Exp/Homer	1e-28	-6.637e+01	0.0000	1590.0	5.40%	1038.9	4.05%	motif file (matrix)	pdf
66	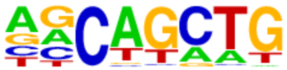	SCL/HPC7-Scl-ChIP-Seq/Homer	1e-27	-6.430e+01	0.0000	28763.0	97.74%	24780.1	96.64%	motif file (matrix)	pdf
67	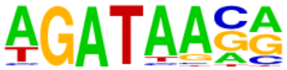	GATA3(Zf)/iTreg-Gata3-ChIP-Seq(GSE20898)/Homer	1e-27	-6.351e+01	0.0000	17885.0	60.78%	14775.4	57.62%	motif file (matrix)	pdf
68	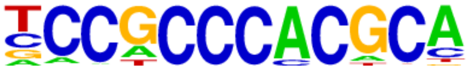	EGR(Zf)/K562-EGR1-ChIP-Seq/Homer	1e-27	-6.241e+01	0.0000	6032.0	20.50%	4620.3	18.02%	motif file (matrix)	pdf
69	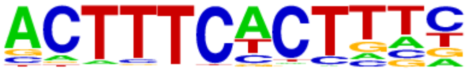	PRDM1/BMI1(Zf)/Hela-PRDM1-ChIP-Seq (GSE31477)/Homer	1e-26	-6.129e+01	0.0000	9328.0	31.70%	7391.3	28.83%	motif file (matrix)	pdf
70	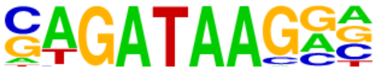	Gata1(Zf)/K562-GATA1-ChIP-Seq/Homer	1e-25	-5.949e+01	0.0000	8447.0	28.70%	6657.4	25.96%	motif file (matrix)	pdf
71	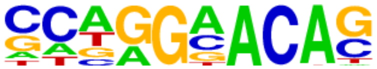	AR-halfsite(NR)/LNCaP-AR-ChIP-Seq/Homer	1e-25	-5.925e+01	0.0000	27990.0	95.11%	24016.3	93.66%	motif file (matrix)	pdf
72	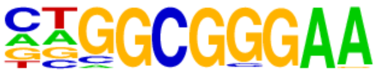	E2F1(E2F)/Hela-E2F1-ChIP-Seq/Hoemr	1e-24	-5.620e+01	0.0000	7460.0	25.35%	5846.5	22.80%	motif file (matrix)	pdf
73	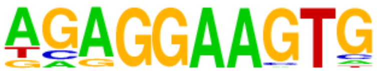	PU.1(ETS)/ThioMac-PU.1-ChIP-Seq/Homer	1e-24	-5.572e+01	0.0000	8320.0	28.27%	6573.4	25.64%	motif file (matrix)	pdf
74	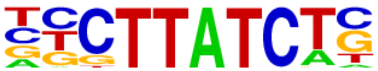	Gata2(Zf)/K562-GATA2-ChIP-Seq/Homer	1e-22	-5.236e+01	0.0000	9318.0	31.66%	7441.0	29.02%	motif file (matrix)	pdf
75	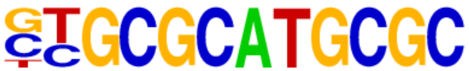	NRF1/Promoter/Homer	1e-21	-5.062e+01	0.0000	4034.0	13.71%	3034.7	11.84%	motif file (matrix)	pdf
76		HOXA9/HSC-Hoxa9-ChIP-Seq (GSE33509)/Homer	1e-20	-4.678e+01	0.0000	9275.0	31.52%	7442.9	29.03%	motif file (matrix)	pdf

	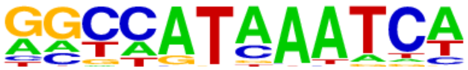										
77	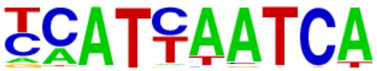	Pdx1(Homeobox)/Islet-Pdx1-ChIP-Seq/Homer	1e-19	-4.604e+01	0.0000	11941.0	40.58%	9729.8	37.95%	motif file (matrix)	pdf
78	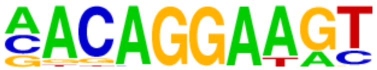	Ets1-distal(ETS)/CD4+-PolII-ChIP-Seq/Homer	1e-18	-4.239e+01	0.0000	4689.0	15.93%	3616.4	14.10%	motif file (matrix)	pdf
79	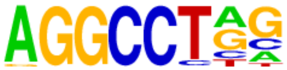	ZFX(Zf)/mES-Zfx-ChIP-Seq/Homer	1e-17	-4.118e+01	0.0000	19682.0	66.88%	16528.0	64.46%	motif file (matrix)	pdf
80	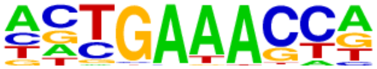	IRF4(IRF)/GM12878-IRF4-ChIP-Seq/Homer	1e-16	-3.846e+01	0.0000	6995.0	23.77%	5570.8	21.73%	motif file (matrix)	pdf
81	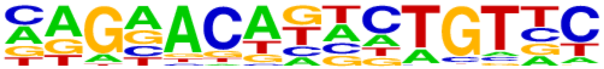	PR(NR)/T47D-PR-ChIP-Seq (GSE31130)/Homer	1e-16	-3.761e+01	0.0000	22526.0	76.55%	19089.3	74.45%	motif file (matrix)	pdf
82	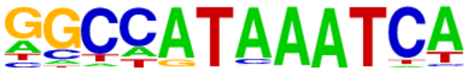	Hoxc9/Ainv15-Hoxc9-ChIP-Seq/Homer	1e-15	-3.679e+01	0.0000	7526.0	25.57%	6031.4	23.52%	motif file (matrix)	pdf
83	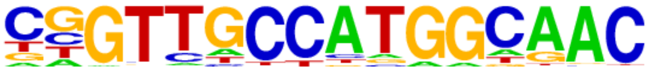	RFX(HTH)/K562-RFX3-ChIP-Seq/Homer	1e-15	-3.577e+01	0.0000	1654.0	5.62%	1179.8	4.60%	motif file (matrix)	pdf
84	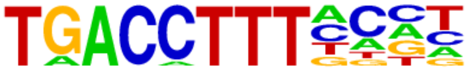	Nur77(NR)/K562-NR4A1-ChIP-Seq(GSE31363)/Homer	1e-15	-3.474e+01	0.0000	2810.0	9.55%	2113.6	8.24%	motif file (matrix)	pdf
85	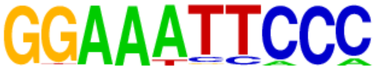	NFkB-p65-Rel(RHD)/LPS-exp/Homer	1e-14	-3.448e+01	0.0000	1260.0	4.28%	874.5	3.41%	motif file (matrix)	pdf
86	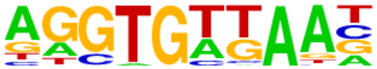	Eomes(T-box)/H9-Eomes-ChIP-Seq/Homer	1e-14	-3.380e+01	0.0000	26960.0	91.61%	23149.0	90.28%	motif file (matrix)	pdf
87	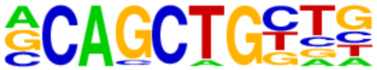	Tcf12(HLH)/GM12878-Tcf12-ChIP-Seq/Homer	1e-14	-3.358e+01	0.0000	15282.0	51.93%	12730.4	49.65%	motif file (matrix)	pdf
88	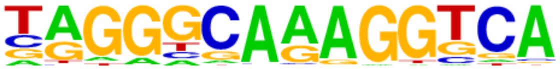	RXR(NR/DR1)/3T3L1-RXR-ChIP-Seq/Homer	1e-13	-3.179e+01	0.0000	17352.0	58.96%	14557.7	56.78%	motif file (matrix)	pdf
89		PRDM14(Zf)/H1-PRDM14-ChIP-Seq/Homer	1e-13	-3.150e+01	0.0000	5129.0	17.43%	4051.3	15.80%	motif file (matrix)	pdf

	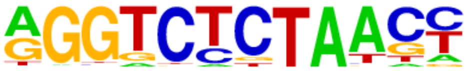										
90	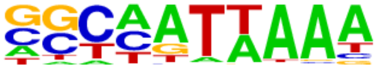	Unknown/Homeobox/Limb-p300-ChIP-Seq/Homer	1e-12	-2.931e+01	0.0000	10037.0	34.11%	8235.4	32.12%	motif file (matrix)	pdf
91	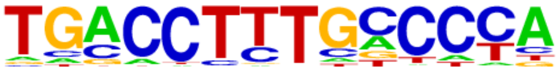	PPARE(NR/DR1)/3T3L1-Pparg-ChIP-Seq/Homer	1e-12	-2.878e+01	0.0000	15173.0	51.56%	12682.4	49.46%	motif file (matrix)	pdf
92	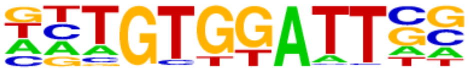	Foxh1(Forkhead)/hESC-FOXH1-ChIP-Seq (GSE29422)/Homer	1e-12	-2.816e+01	0.0000	9142.0	31.07%	7479.5	29.17%	motif file (matrix)	pdf
93	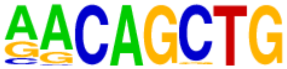	MyoG(HLH)/C2C12-MyoG-ChIP-Seq(GSE36024)/Homer	1e-11	-2.691e+01	0.0000	15873.0	53.94%	13312.9	51.92%	motif file (matrix)	pdf
94	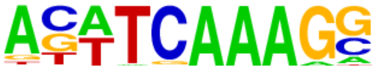	Tcf3(HMG)/mES-Tcf3-ChIP-Seq/Homer	1e-11	-2.657e+01	0.0000	3751.0	12.75%	2935.1	11.45%	motif file (matrix)	pdf
95	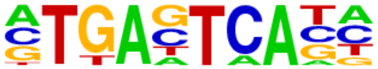	HIF1b(HLH)/O785-HIF1b-ChIP-Seq(GSE34871)/Homer	1e-11	-2.548e+01	0.0000	7605.0	25.84%	6192.9	24.15%	motif file (matrix)	pdf
96	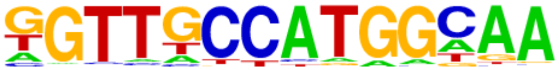	X-box(HTH)/NPC-H3K4me1-ChIP-Seq/Homer	1e-11	-2.547e+01	0.0000	1803.0	6.13%	1341.6	5.23%	motif file (matrix)	pdf
97	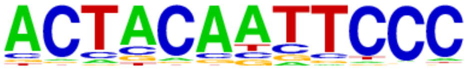	GFY(?)/Promoter/Homer	1e-10	-2.477e+01	0.0000	1562.0	5.31%	1150.1	4.49%	motif file (matrix)	pdf
98	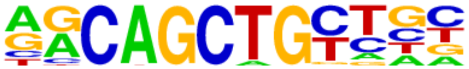	MyoD(HLH)/Myotube-MyoD-ChIP-Seq/Homer	1e-9	-2.198e+01	0.0000	12652.0	42.99%	10566.4	41.21%	motif file (matrix)	pdf
99	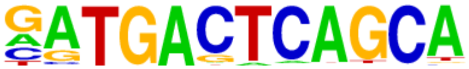	NF-E2(bZIP)/K562-NFE2-ChIP-Seq/Homer	1e-9	-2.190e+01	0.0000	799.0	2.72%	556.3	2.17%	motif file (matrix)	pdf
100	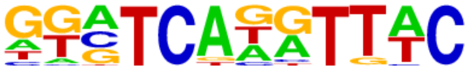	Six1(Homeobox)/Myoblast-Six1-ChIP-Chip (GSE20150)/Homer	1e-9	-2.151e+01	0.0000	3902.0	13.26%	3097.6	12.08%	motif file (matrix)	pdf
101	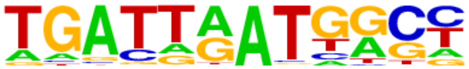	Hoxb4/ES-Hoxb4-ChIP-Seq (GSE34014)/Homer	1e-8	-2.028e+01	0.0000	2693.0	9.15%	2099.2	8.19%	motif file (matrix)	pdf
102		HIF2a(HLH)/O785-HIF2a-ChIP-Seq(GSE34871)/Homer	1e-8	-1.999e+01	0.0000	7006.0	23.81%	5735.9	22.37%	motif file (matrix)	pdf

	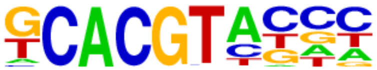										
103	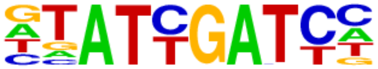	HNF6(Homeobox)/Liver-Hnf6-ChIP-Seq(ERP000394)	1e-7	-1.840e+01	0.0000	7710.0	26.20%	6353.6	24.78%	motif file (matrix)	pdf
104	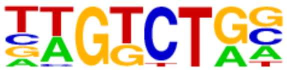	Smad3(MAD)/NPC-Smad3-ChIP-Seq(GSE36673)/Homer	1e-7	-1.814e+01	0.0000	26159.0	88.89%	22523.9	87.84%	motif file (matrix)	pdf
105	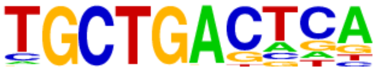	MafA(bZIP)/Islet-MafA-ChIP-Seq(GSE30298)/Homer	1e-7	-1.748e+01	0.0000	10730.0	36.46%	8959.8	34.94%	motif file (matrix)	pdf
106	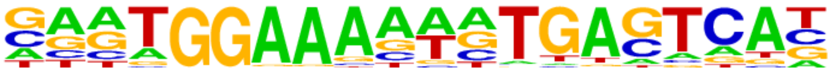	NFAT:AP1/Jurkat-NFATC1-ChIP-Seq/Homer	1e-7	-1.727e+01	0.0000	2986.0	10.15%	2364.7	9.22%	motif file (matrix)	pdf
107	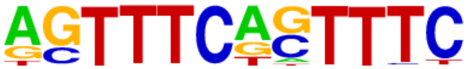	ISRE(IRF)/ThioMac-LPS-exp/HOMER	1e-7	-1.685e+01	0.0000	1185.0	4.03%	883.4	3.45%	motif file (matrix)	pdf
108	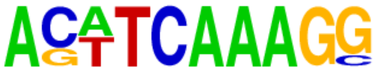	TCFL2(HMG)/K562-TCF7L2-ChIP-Seq(GSE29196)/Homer	1e-7	-1.671e+01	0.0000	1173.0	3.99%	874.0	3.41%	motif file (matrix)	pdf
109	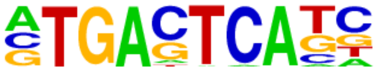	AP-1(bZIP)/ThioMac-PU.1-ChIP-Seq/Homer	1e-7	-1.636e+01	0.0000	9251.0	31.44%	7699.9	30.03%	motif file (matrix)	pdf
110	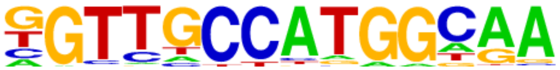	Rfx1(HTH)/NPC-Rfx1-ChIP-Seq/Homer	1e-6	-1.597e+01	0.0000	3493.0	11.87%	2799.3	10.92%	motif file (matrix)	pdf
111	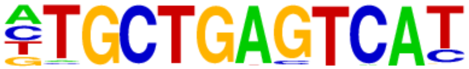	Nrf2(bZIP)/Lymphoblast-Nrf2-ChIP-Seq(GSE37589)/Homer	1e-6	-1.546e+01	0.0000	686.0	2.33%	490.4	1.91%	motif file (matrix)	pdf
112	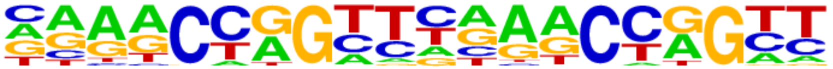	Tcfcp2l1(CP2)/mES-Tcfcp2l1-ChIP-Seq/Homer	1e-6	-1.534e+01	0.0000	2491.0	8.46%	1966.2	7.67%	motif file (matrix)	pdf
113	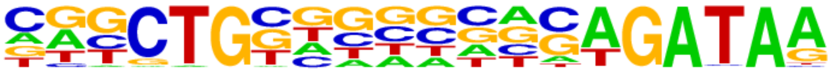	GATA:SCL/Ter119-SCL-ChIP-Seq/Homer	1e-6	-1.420e+01	0.0000	1915.0	6.51%	1496.9	5.84%	motif file (matrix)	pdf
114	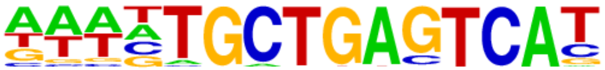	Bach1(bZIP)/K562-Bach1-ChIP-Seq(GSE31477)/Homer	1e-6	-1.404e+01	0.0000	703.0	2.39%	509.1	1.99%	motif file (matrix)	pdf
115		T1ISRE(IRF)/Ifnb-Exp/Homer	1e-5	-1.300e+01	0.0000	221.0	0.75%	139.4	0.54%	motif file (matrix)	pdf

	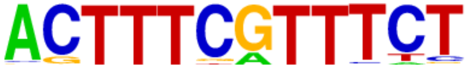										
116	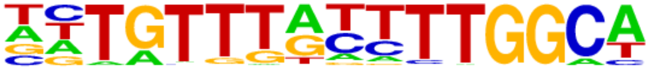	NF1:FOXA1/LNCAP-FOXA1-ChIP-Seq/Homer	1e-5	-1.294e+01	0.0000	771.0	2.62%	568.9	2.22%	motif file (matrix)	pdf
117	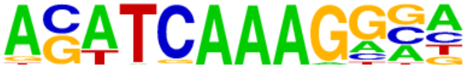	Tcf4(HMG)/Hct116-Tcf4-ChIP-Seq/Homer	1e-5	-1.270e+01	0.0000	6451.0	21.92%	5344.5	20.84%	motif file (matrix)	pdf
118	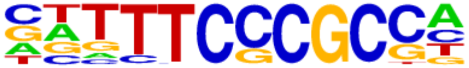	E2F7(E2F)/Hela-E2F7-ChIP-Seq(GSE32673)/Homer	1e-5	-1.234e+01	0.0000	3806.0	12.93%	3097.5	12.08%	motif file (matrix)	pdf
119	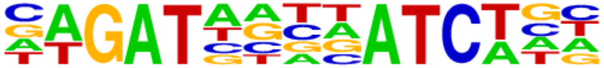	GATA-IR4(Zf)/iTreg-Gata3-ChIP-Seq(GSE20898)/Homer	1e-5	-1.192e+01	0.0000	1104.0	3.75%	844.0	3.29%	motif file (matrix)	pdf
120	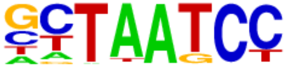	CRX(Homeobox)/Retina-Crx-ChIP-Seq/Homer	1e-4	-1.104e+01	0.0000	24796.0	84.26%	21374.2	83.36%	motif file (matrix)	pdf
121	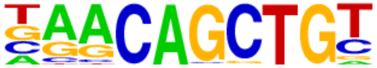	Myf5(bHLH)/GM-Myf5-ChIP-Seq(GSE24852)/Homer	1e-4	-1.078e+01	0.0000	10923.0	37.12%	9222.2	35.97%	motif file (matrix)	pdf
122	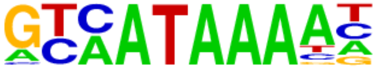	Cdx2(Homeobox)/mES-Cdx2-ChIP-Seq/Homer	1e-4	-9.755e+00	0.0001	10162.0	34.53%	8582.0	33.47%	motif file (matrix)	pdf
123	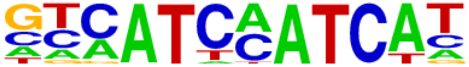	HOXA2(Homeobox)/mES-Hoxa2-ChIP-Seq/Homer	1e-3	-9.170e+00	0.0002	1377.0	4.68%	1086.5	4.24%	motif file (matrix)	pdf
124	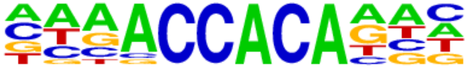	RUNX2(Runt)/PCa-RUNX2-ChIP-Seq(GSE33889)/Homer	1e-3	-8.475e+00	0.0004	11288.0	38.36%	9579.9	37.36%	motif file (matrix)	pdf
125	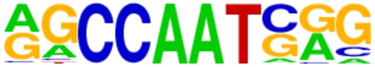	NFY (CCAAT)/Promoter/Homer	1e-3	-8.108e+00	0.0005	11116.0	37.77%	9437.6	36.81%	motif file (matrix)	pdf
126	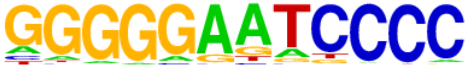	NFkB-p50,p52(RHD)/p50-ChIP-Chip/Homer	1e-3	-6.954e+00	0.0016	3244.0	11.02%	2683.8	10.47%	motif file (matrix)	pdf
127	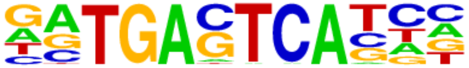	Jun-AP1(bZIP)/K562-cJun-ChIP-Seq/Homer	1e-2	-6.832e+00	0.0018	3134.0	10.65%	2591.0	10.11%	motif file (matrix)	pdf
128		CArG(MADS)/PUER-Srf-ChIP-Seq/Homer	1e-2	-6.639e+00	0.0022	4649.0	15.80%	3888.3	15.16%	motif file (matrix)	pdf

	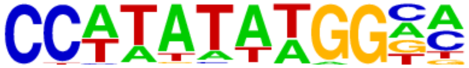										
129	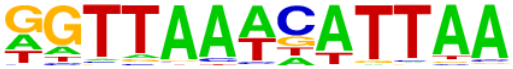	Hnf1(Homeobox)/Liver-Foxa2-Chip-Seq/Homer	1e-2	-6.536e+00	0.0024	2427.0	8.25%	1994.6	7.78%	motif file (matrix)	pdf
130	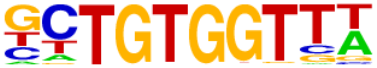	RUNX-AML (Runt)/CD4+-PolII-ChIP-Seq/Homer	1e-2	-6.446e+00	0.0026	10167.0	34.55%	8649.0	33.73%	motif file (matrix)	pdf
131	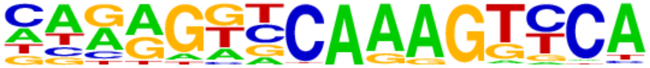	HNF4a(NR/DR1)/HepG2-HNF4a-ChIP-Seq/Homer	1e-2	-5.984e+00	0.0041	7257.0	24.66%	6143.9	23.96%	motif file (matrix)	pdf
132	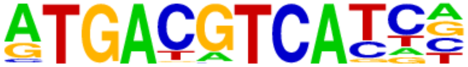	JunD(bZIP)/K562-JunD-ChIP-Seq/Homer	1e-2	-5.894e+00	0.0045	1183.0	4.02%	951.4	3.71%	motif file (matrix)	pdf
133	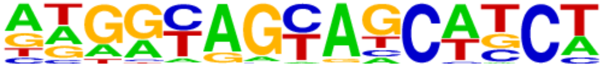	PRDM9(Zf)/Testis-DMC1-ChIP-Seq(GSE35498)/Homer	1e-2	-5.719e+00	0.0053	6911.0	23.48%	5850.1	22.82%	motif file (matrix)	pdf
134	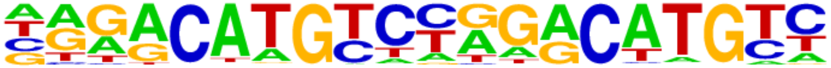	p53(p53)/p53-ChIP-Chip/Homer	1e-2	-5.446e+00	0.0069	121.0	0.41%	82.1	0.32%	motif file (matrix)	pdf
135	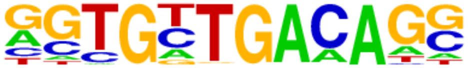	Tbx20(T-box)/Heart-Tbx20-ChIP-Seq(GSE29636)/Homer	1e-2	-5.409e+00	0.0071	3198.0	10.87%	2666.4	10.40%	motif file (matrix)	pdf
136	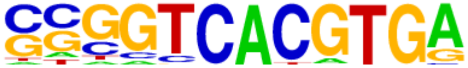	E-box(HLH)/Promoter/Homer	1e-2	-5.369e+00	0.0074	1493.0	5.07%	1217.8	4.75%	motif file (matrix)	pdf
137	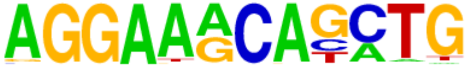	ETS:E-box/HPC7-Scl-ChIP-Seq/Homer	1e-2	-5.017e+00	0.0104	1583.0	5.38%	1297.3	5.06%	motif file (matrix)	pdf
138	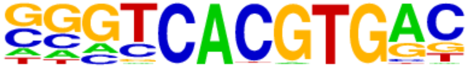	ATF3(bZIP)/K562-ATF3-ChIP-Seq/Homer	1e-2	-4.912e+00	0.0115	3073.0	10.44%	2567.4	10.01%	motif file (matrix)	pdf
139	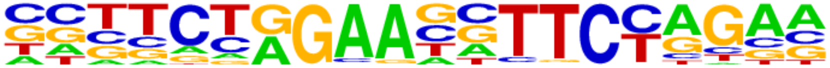	HRE(HSF)/HepG2-HSF1-ChIP-Seq/Homer	1e-2	-4.878e+00	0.0118	2474.0	8.41%	2056.8	8.02%	motif file (matrix)	pdf
140	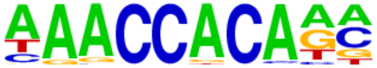	RUNX1(Runt)/Jurkat-RUNX1-ChIP-Seq/Homer	1e-2	-4.799e+00	0.0127	13503.0	45.88%	11586.9	45.19%	motif file (matrix)	pdf

Results homer motif enrichments (known motifs)

TCam-2 h3k27ac

Homer Known Motif Enrichment Results

Homer *de novo* Motif Results
Gene Ontology Enrichment Results
Known Motif Enrichment Results (txt file)
Total Target Sequences = 41569, Total Background Sequences = 39569

Rank	Motif	Name	P-value	log P-pvalue	q-value (Benjamini)	# Target Sequences with Motif	% of Targets Sequences with Motif	# Background Sequences with Motif	% of Background Sequences with Motif	Motif File	PDF
1	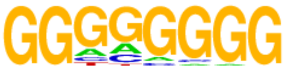	Maz(Zf)/HepG2-Maz-ChIP-Seq (GSE31477)/Homer	1e-275	-6.346e+02	0.0000	27868.0	67.04%	23175.4	58.57%	motif file (matrix)	pdf
2	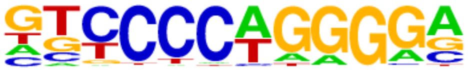	EBF1(EBF)/Near-E2A-ChIP-Seq/Homer	1e-234	-5.391e+02	0.0000	25935.0	62.39%	21547.2	54.46%	motif file (matrix)	pdf
3	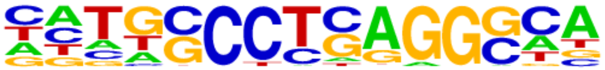	AP2gamma(AP2)/MCF7-TFAP2c-ChIP-Seq/Homer	1e-224	-5.179e+02	0.0000	23712.0	57.04%	19464.1	49.19%	motif file (matrix)	pdf
4	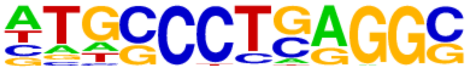	AP-2alpha(AP2)/Hela-AP2alpha-ChIP-Seq/Homer	1e-224	-5.179e+02	0.0000	22943.0	55.19%	18730.6	47.34%	motif file (matrix)	pdf
5	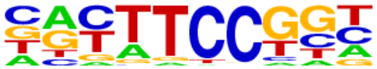	Fli1(ETS)/CD8-FLI-ChIP-Seq (GSE20898)/Homer	1e-222	-5.126e+02	0.0000	24983.0	60.10%	20703.1	52.32%	motif file (matrix)	pdf
6	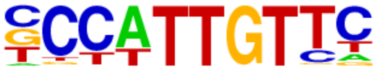	Sox2(HMG)/mES-Sox2-ChIP-Seq/Homer	1e-182	-4.210e+02	0.0000	20355.0	48.97%	16593.5	41.94%	motif file (matrix)	pdf
7	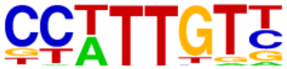	Sox3(HMG)/NPC-Sox3-ChIP-Seq(GSE33059)/Homer	1e-171	-3.960e+02	0.0000	30831.0	74.17%	26858.1	67.88%	motif file (matrix)	pdf
8	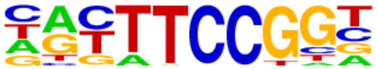	Elk4(ETS)/Hela-Elk4-ChIP-Seq (GSE31477)/Homer	1e-171	-3.939e+02	0.0000	14373.0	34.58%	11191.8	28.29%	motif file (matrix)	pdf
9	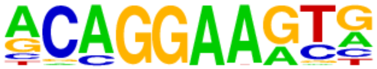	ERG(ETS)/VCaP-ERG-ChIP-Seq/Homer	1e-165	-3.820e+02	0.0000	31046.0	74.69%	27122.3	68.55%	motif file (matrix)	pdf
10	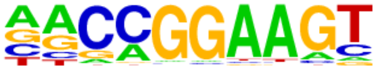	GABPA(ETS)/Jurkat-GABPa-ChIP-Seq/Homer	1e-163	-3.755e+02	0.0000	21184.0	50.96%	17527.9	44.30%	motif file (matrix)	pdf
11		ETV1(ETS)/GIST48-ETV1-ChIP-Seq/Homer	1e-158	-3.640e+02	0.0000	28252.0	67.96%	24390.6	61.64%	motif file (matrix)	pdf

	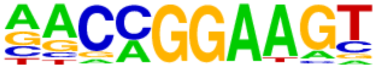										
12	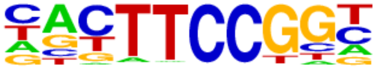	Elk1(ETS)/Hela-Elk1-ChIP-Seq (GSE31477)/Homer	1e-156	-3.595e+02	0.0000	14292.0	34.38%	11226.3	28.37%	motif file (matrix)	pdf
13	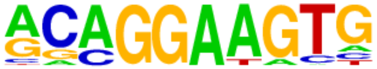	ETS1(ETS)/Jurkat-ETS1-ChIP-Seq/Homer	1e-147	-3.397e+02	0.0000	23618.0	56.82%	19971.2	50.47%	motif file (matrix)	pdf
14	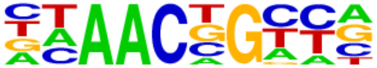	BMYB(HTH)/Hela-BMYB-ChIPSeq(GSE27030)/Homer	1e-145	-3.349e+02	0.0000	28066.0	67.52%	24312.5	61.45%	motif file (matrix)	pdf
15	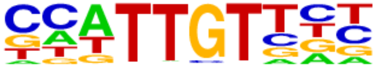	Sox6(HMG)/Myotubes-Sox6-ChIP-Seq(GSE32627)/Homer	1e-136	-3.144e+02	0.0000	29421.0	70.78%	25731.1	65.03%	motif file (matrix)	pdf
16	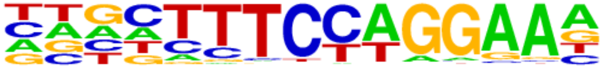	Bcl6(Zf)/Liver-Bcl6-ChIP-Seq (GSE31578)/Homer	1e-131	-3.030e+02	0.0000	28213.0	67.87%	24578.9	62.12%	motif file (matrix)	pdf
17	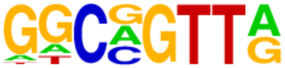	MYB(HTH)/ERMYB-Myb-ChIPSeq(GSE22095)/Homer	1e-121	-2.807e+02	0.0000	30287.0	72.86%	26724.4	67.54%	motif file (matrix)	pdf
18	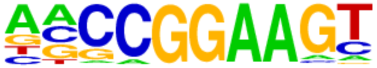	ELF1(ETS)/Jurkat-ELF1-ChIP-Seq/Homer	1e-119	-2.757e+02	0.0000	13366.0	32.15%	10677.3	26.98%	motif file (matrix)	pdf
19	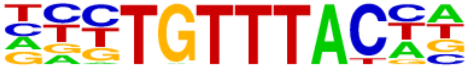	FOXP1(Forkhead)/H9-FOXP1-ChIP-Seq(GSE31006)/Homer	1e-118	-2.733e+02	0.0000	11849.0	28.50%	9324.9	23.57%	motif file (matrix)	pdf
20	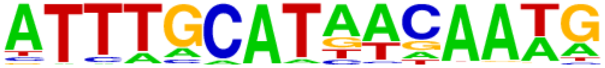	OCT4-SOX2-TCF-NANOG ((POU/Homeobox/HMG)/mES-ChIP-Seq/Homer	1e-116	-2.692e+02	0.0000	5868.0	14.12%	4152.7	10.50%	motif file (matrix)	pdf
21	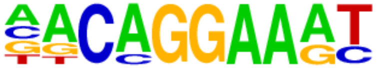	EWS:FLI1-fusion (ETS)/SK_N_MC-EWS:FLI1-ChIP-Seq/Homer	1e-115	-2.654e+02	0.0000	14724.0	35.42%	11947.7	30.20%	motif file (matrix)	pdf
22	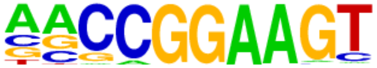	ETS(ETS)/Promoter/Homer	1e-113	-2.605e+02	0.0000	8885.0	21.37%	6752.7	17.07%	motif file (matrix)	pdf
23	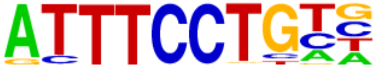	EWS:ERG-fusion (ETS)/CADO_ES1-EWS:ERG-ChIP-Seq/Homer	1e-110	-2.538e+02	0.0000	18273.0	43.96%	15265.8	38.58%	motif file (matrix)	pdf
24		TEAD4(TEA)/Tropoblast-Tead4-ChIP-Seq (GSE37350)/Homer	1e-109	-2.527e+02	0.0000	21097.0	50.75%	17921.5	45.29%	motif file (matrix)	pdf

	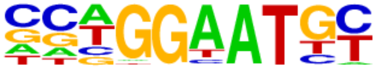										
25	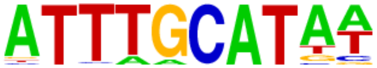	Oct4(POU/Homeobox)/mES-Oct4-ChIP-Seq/Homer	1e-107	-2.477e+02	0.0000	12575.0	30.25%	10067.7	25.44%	motif file (matrix)	pdf
26	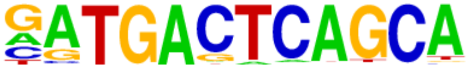	NF-E2(bZIP)/K562-NFE2-ChIP-Seq/Homer	1e-102	-2.359e+02	0.0000	1779.0	4.28%	978.4	2.47%	motif file (matrix)	pdf
27	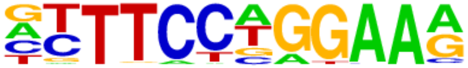	STAT4(Stat)/CD4-Stat4-ChIP-Seq/Homer	1e-100	-2.317e+02	0.0000	21618.0	52.01%	18507.6	46.77%	motif file (matrix)	pdf
28	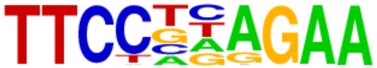	STAT6/Macrophage-Stat6-ChIP-Seq/Homer	1e-97	-2.245e+02	0.0000	13682.0	32.91%	11160.6	28.21%	motif file (matrix)	pdf
29	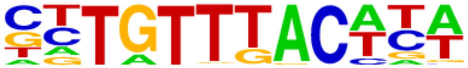	Foxa2(Forkhead)/Liver-Foxa2-ChIP-Seq/Homer	1e-96	-2.233e+02	0.0000	18099.0	43.54%	15236.0	38.51%	motif file (matrix)	pdf
30	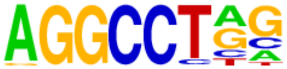	ZFX(Zf)/mES-Zfx-ChIP-Seq/Homer	1e-91	-2.099e+02	0.0000	28054.0	67.49%	24816.6	62.72%	motif file (matrix)	pdf
31	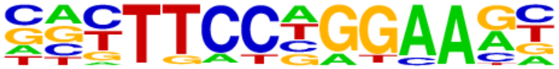	Stat3+il23(Stat)/CD4-Stat3-ChIP-Seq/Homer	1e-90	-2.075e+02	0.0000	17289.0	41.59%	14552.5	36.78%	motif file (matrix)	pdf
32	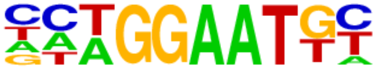	TEAD(TEA)/Fibroblast-PU.1-ChIP-Seq/Homer	1e-90	-2.074e+02	0.0000	17615.0	42.38%	14856.4	37.55%	motif file (matrix)	pdf
33	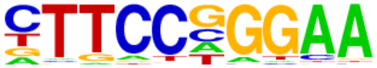	Stat3(Stat)/mES-Stat3-ChIP-Seq/Homer	1e-89	-2.068e+02	0.0000	13321.0	32.05%	10905.8	27.56%	motif file (matrix)	pdf
34	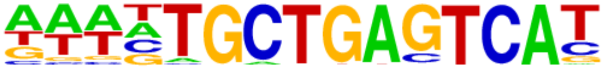	Bach1(bZIP)/K562-Bach1-ChIP-Seq(GSE31477)/Homer	1e-87	-2.019e+02	0.0000	1627.0	3.91%	912.8	2.31%	motif file (matrix)	pdf
35	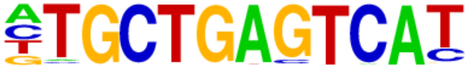	Nrf2(bZIP)/Lymphoblast-Nrf2-ChIP-Seq(GSE37589)/Homer	1e-84	-1.951e+02	0.0000	1521.0	3.66%	844.5	2.13%	motif file (matrix)	pdf
36	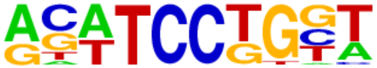	SPDEF(ETS)/VCaP-SPDEF-ChIP-Seq/Homer	1e-84	-1.937e+02	0.0000	22633.0	54.45%	19653.7	49.67%	motif file (matrix)	pdf
37		EBF(EBF)/proBcell-EBF-ChIP-Seq/Homer	1e-82	-1.911e+02	0.0000	7419.0	17.85%	5702.0	14.41%	motif file (matrix)	pdf

	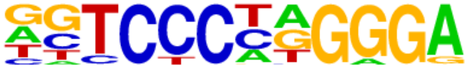										
38	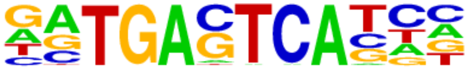	Jun-AP1(bZIP)/K562-cJun-ChIP-Seq/Homer	1e-82	-1.902e+02	0.0000	6165.0	14.83%	4621.9	11.68%	motif file (matrix)	pdf
39	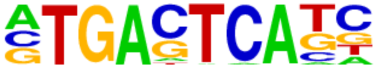	AP-1(bZIP)/ThioMac-PU.1-ChIP-Seq/Homer	1e-81	-1.867e+02	0.0000	16352.0	39.34%	13780.7	34.83%	motif file (matrix)	pdf
40	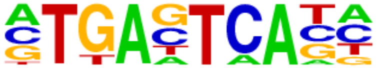	HIF1b(HLH)/O785-HIF1b-ChIP-Seq(GSE34871)/Homer	1e-80	-1.862e+02	0.0000	13525.0	32.54%	11181.9	28.26%	motif file (matrix)	pdf
41	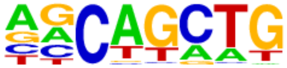	SCL/HPC7-Scl-ChIP-Seq/Homer	1e-72	-1.670e+02	0.0000	40765.0	98.07%	38215.6	96.58%	motif file (matrix)	pdf
42	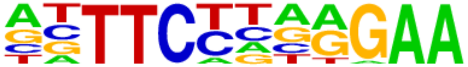	STAT6(Stat)/CD4-Stat6-ChIP-Seq/Homer	1e-70	-1.627e+02	0.0000	13459.0	32.38%	11230.4	28.38%	motif file (matrix)	pdf
43	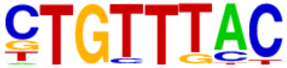	Foxo1(Forkhead)/RAW-Foxo1-ChIP-Seq/Homer	1e-68	-1.577e+02	0.0000	33079.0	79.58%	30057.5	75.96%	motif file (matrix)	pdf
44	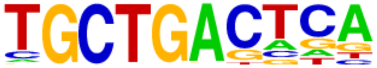	MafA(bZIP)/Islet-MafA-ChIP-Seq(GSE30298)/Homer	1e-67	-1.548e+02	0.0000	17043.0	41.00%	14584.0	36.86%	motif file (matrix)	pdf
45	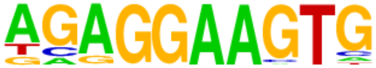	PU.1(ETS)/ThioMac-PU.1-ChIP-Seq/Homer	1e-66	-1.525e+02	0.0000	12787.0	30.76%	10665.0	26.95%	motif file (matrix)	pdf
46	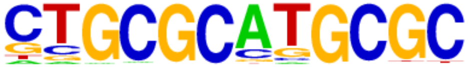	NRF1(NRF)/MCF7-NRF1-ChIP-Seq/Homer	1e-65	-1.514e+02	0.0000	2897.0	6.97%	1988.5	5.03%	motif file (matrix)	pdf
47	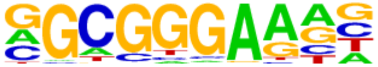	E2F6(E2F)/Hela-E2F6-ChIP-Seq(GSE31477)/Homer	1e-64	-1.493e+02	0.0000	13830.0	33.27%	11637.9	29.41%	motif file (matrix)	pdf
48	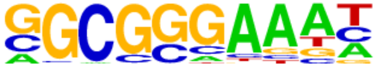	E2F4(E2F)/K562-E2F4-ChIP-Seq(GSE31477)/Homer	1e-64	-1.477e+02	0.0000	10909.0	26.24%	8980.6	22.70%	motif file (matrix)	pdf
49	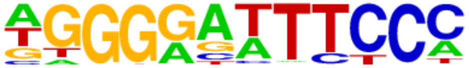	NFkB-p65(RHD)/GM12787-p65-ChIP-Seq/Homer	1e-60	-1.400e+02	0.0000	14838.0	35.69%	12615.7	31.88%	motif file (matrix)	pdf
50		Znf263(Zf)/K562-Znf263-ChIP-Seq/Homer	1e-59	-1.364e+02	0.0000	33373.0	80.28%	30458.6	76.98%	motif file (matrix)	pdf

	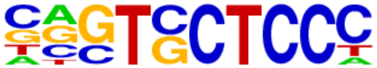										
51	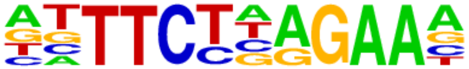	STAT5 (Stat)/mCD4+-Stat5a|b-ChIP-Seq/Homer	1e-58	-1.350e+02	0.0000	9121.0	21.94%	7428.9	18.78%	motif file (matrix)	pdf
52	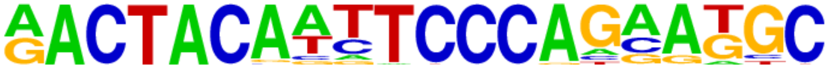	GFY-Staf/Promoters/Homer	1e-57	-1.325e+02	0.0000	1592.0	3.83%	991.2	2.51%	motif file (matrix)	pdf
53	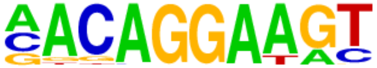	Ets1-distal(ETS)/CD4+-PolII-ChIP-Seq/Homer	1e-57	-1.315e+02	0.0000	7726.0	18.59%	6199.7	15.67%	motif file (matrix)	pdf
54	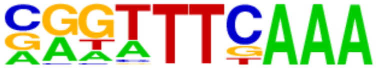	CHR/Cell-Cycle-Exp/Homer	1e-55	-1.280e+02	0.0000	15989.0	38.46%	13750.8	34.75%	motif file (matrix)	pdf
55	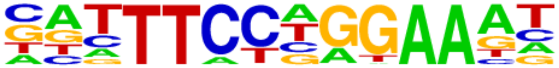	STAT1(Stat)/HelaS3-STAT1-ChIP-Seq/Homer	1e-55	-1.274e+02	0.0000	8168.0	19.65%	6610.7	16.71%	motif file (matrix)	pdf
56	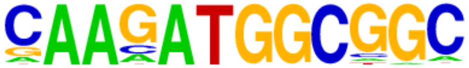	YY1(Zf)/Promoter/Homer	1e-51	-1.189e+02	0.0000	2256.0	5.43%	1544.6	3.90%	motif file (matrix)	pdf
57	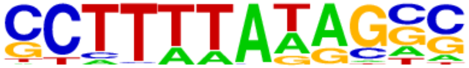	TATA-Box (TBP)/Promoter/Homer	1e-49	-1.149e+02	0.0000	26190.0	63.00%	23514.1	59.43%	motif file (matrix)	pdf
58	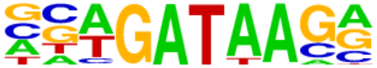	Gata4(Zf)/Heart-Gata4-ChIP-Seq(GSE35151)/Homer	1e-48	-1.109e+02	0.0000	21199.0	51.00%	18757.2	47.41%	motif file (matrix)	pdf
59	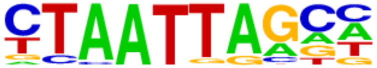	Lhx3(Homeobox)/Forebrain-p300-ChIP-Seq/Homer	1e-46	-1.064e+02	0.0000	21457.0	51.62%	19032.6	48.10%	motif file (matrix)	pdf
60	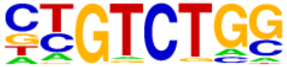	Smad2(MAD)/ES-SMAD2-ChIP-Seq(GSE29422)/Homer	1e-41	-9.571e+01	0.0000	29203.0	70.25%	26569.5	67.15%	motif file (matrix)	pdf
61	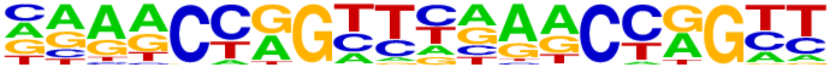	Tcfcp2l1(CP2)/mES-Tcfcp2l1-ChIP-Seq/Homer	1e-41	-9.563e+01	0.0000	3753.0	9.03%	2865.8	7.24%	motif file (matrix)	pdf
62	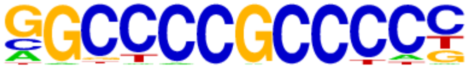	Sp1(Zf)/Promoter/Homer	1e-41	-9.443e+01	0.0000	8664.0	20.84%	7218.9	18.24%	motif file (matrix)	pdf
63		Gata2(Zf)/K562-GATA2-ChIP-Seq/Homer	1e-40	-9.434e+01	0.0000	15036.0	36.17%	13072.9	33.04%	motif file (matrix)	pdf

	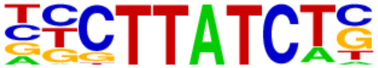										
64	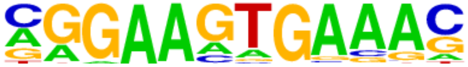	PU.1-IRF/Bcell-PU.1-ChIP-Seq/Homer	1e-40	-9.371e+01	0.0000	27971.0	67.29%	25382.9	64.15%	motif file (matrix)	pdf
65	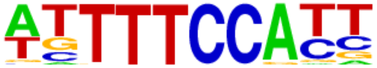	NFAT(RHD)/Jurkat-NFATC1-ChIP-Seq/Homer	1e-40	-9.276e+01	0.0000	20350.0	48.95%	18076.1	45.68%	motif file (matrix)	pdf
66	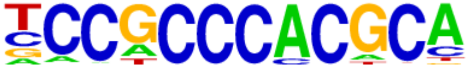	EGR(Zf)/K562-EGR1-ChIP-Seq/Homer	1e-40	-9.211e+01	0.0000	5698.0	13.71%	4576.1	11.57%	motif file (matrix)	pdf
67	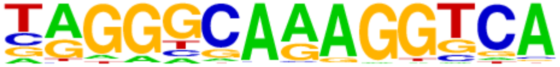	RXR(NR/DR1)/3T3L1-RXR-ChIP-Seq/Homer	1e-38	-8.931e+01	0.0000	25156.0	60.52%	22691.8	57.35%	motif file (matrix)	pdf
68	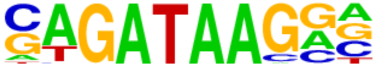	Gata1(Zf)/K562-GATA1-ChIP-Seq/Homer	1e-36	-8.343e+01	0.0000	13513.0	32.51%	11731.4	29.65%	motif file (matrix)	pdf
69	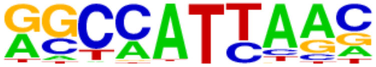	Nanog(Homeobox)/mES-Nanog-ChIP-Seq/Homer	1e-34	-8.045e+01	0.0000	40506.0	97.44%	38127.5	96.36%	motif file (matrix)	pdf
70	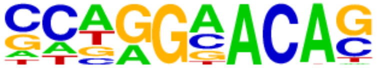	AR-halfsite(NR)/LNCaP-AR-ChIP-Seq/Homer	1e-34	-7.988e+01	0.0000	39987.0	96.19%	37556.8	94.92%	motif file (matrix)	pdf
71	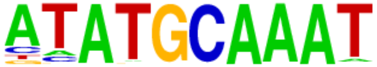	Oct2(POU/Homeobox)/Bcell-Oct2-ChIP-Seq/Homer	1e-32	-7.585e+01	0.0000	8304.0	19.98%	6998.6	17.69%	motif file (matrix)	pdf
72	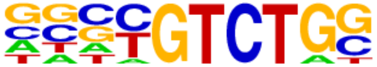	Smad4(MAD)/ESC-SMAD4-ChIP-Seq(GSE29422)/Homer	1e-32	-7.463e+01	0.0000	29389.0	70.70%	26901.2	67.99%	motif file (matrix)	pdf
73	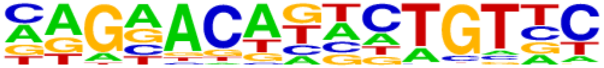	PR(NR)/T47D-PR-ChIP-Seq (GSE31130)/Homer	1e-32	-7.461e+01	0.0000	33815.0	81.35%	31256.9	79.00%	motif file (matrix)	pdf
74	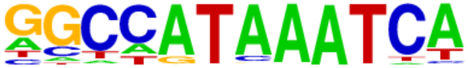	Hoxc9/Ainv15-Hoxc9-ChIP-Seq/Homer	1e-31	-7.281e+01	0.0000	12157.0	29.25%	10550.6	26.66%	motif file (matrix)	pdf
75	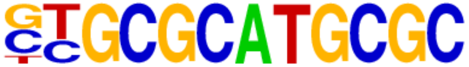	NRF1/Promoter/Homer	1e-31	-7.273e+01	0.0000	3546.0	8.53%	2773.8	7.01%	motif file (matrix)	pdf
76		HOXA9/HSC-Hoxa9-ChIP-Seq (GSE33509)/Homer	1e-31	-7.221e+01	0.0000	15133.0	36.40%	13321.7	33.67%	motif file (matrix)	pdf

	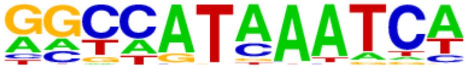										
77	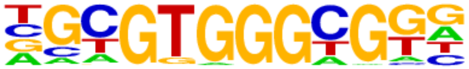	Egr2/Thymocytes-Egr2-ChIP-Seq(GSE34254)/Homer	1e-29	-6.857e+01	0.0000	5032.0	12.11%	4098.4	10.36%	motif file (matrix)	pdf
78	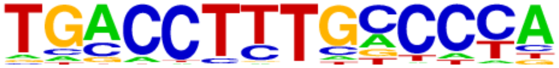	PPARE(NR/DR1)/3T3L1-Pparg-ChIP-Seq/Homer	1e-28	-6.591e+01	0.0000	22428.0	53.95%	20263.5	51.21%	motif file (matrix)	pdf
79	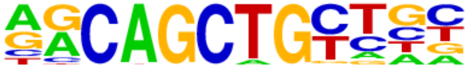	MyoD(HLH)/Myotube-MyoD-ChIP-Seq/Homer	1e-28	-6.569e+01	0.0000	17566.0	42.26%	15656.7	39.57%	motif file (matrix)	pdf
80	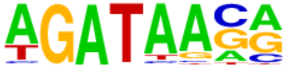	GATA3(Zf)/iTreg-Gata3-ChIP-Seq(GSE20898)/Homer	1e-28	-6.454e+01	0.0000	27694.0	66.62%	25335.5	64.03%	motif file (matrix)	pdf
81	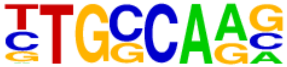	NF1-halfsite(CTF)/LNCaP-NF1-ChIP-Seq/Homer	1e-27	-6.238e+01	0.0000	31007.0	74.59%	28577.9	72.23%	motif file (matrix)	pdf
82	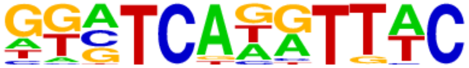	Six1(Homeobox)/Myoblast-Six1-ChIP-Chip (GSE20150)/Homer	1e-26	-6.163e+01	0.0000	6358.0	15.30%	5323.8	13.45%	motif file (matrix)	pdf
83	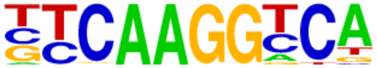	Nr5a2(NR)/mES-Nr5a2-ChIP-Seq/Homer	1e-25	-5.868e+01	0.0000	13133.0	31.59%	11564.4	29.23%	motif file (matrix)	pdf
84	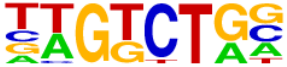	Smad3(MAD)/NPC-Smad3-ChIP-Seq(GSE36673)/Homer	1e-25	-5.764e+01	0.0000	37819.0	90.98%	35389.5	89.44%	motif file (matrix)	pdf
85	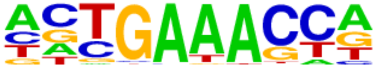	IRF4(IRF)/GM12878-IRF4-ChIP-Seq/Homer	1e-24	-5.669e+01	0.0000	10965.0	26.38%	9569.2	24.18%	motif file (matrix)	pdf
86	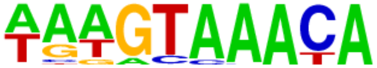	FOXA1(Forkhead)/LNCAP-FOXA1-ChIP-Seq/Homer	1e-24	-5.555e+01	0.0000	24644.0	59.28%	22476.4	56.80%	motif file (matrix)	pdf
87	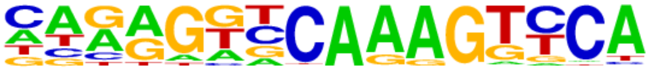	HNF4a(NR/DR1)/HepG2-HNF4a-ChIP-Seq/Homer	1e-23	-5.490e+01	0.0000	11575.0	27.85%	10148.1	25.65%	motif file (matrix)	pdf
88	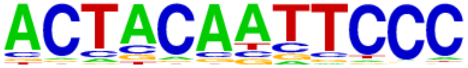	GFY(?)/Promoter/Homer	1e-23	-5.317e+01	0.0000	1998.0	4.81%	1515.7	3.83%	motif file (matrix)	pdf
89		Foxh1(Forkhead)/hESC-FOXH1-ChIP-Seq (GSE29422)/Homer	1e-22	-5.192e+01	0.0000	14582.0	35.08%	12975.8	32.79%	motif file (matrix)	pdf

	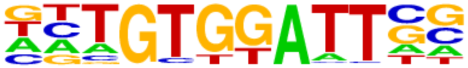										
90	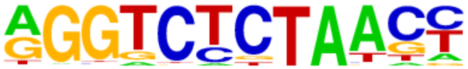	PRDM14(Zf)/H1-PRDM14-ChIP-Seq/Homer	1e-21	-4.935e+01	0.0000	7830.0	18.84%	6742.5	17.04%	motif file (matrix)	pdf
91	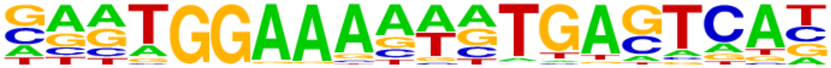	NFAT:AP1/Jurkat-NFATC1-ChIP-Seq/Homer	1e-20	-4.785e+01	0.0000	4929.0	11.86%	4120.8	10.41%	motif file (matrix)	pdf
92	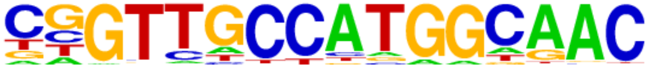	RFX(HTH)/K562-RFX3-ChIP-Seq/Homer	1e-20	-4.776e+01	0.0000	1941.0	4.67%	1487.0	3.76%	motif file (matrix)	pdf
93	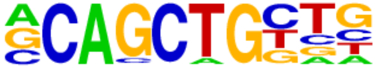	Tcf12(HLH)/GM12878-Tcf12-ChIP-Seq/Homer	1e-20	-4.751e+01	0.0000	21322.0	51.29%	19381.1	48.98%	motif file (matrix)	pdf
94	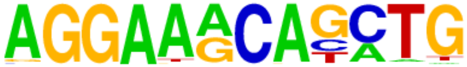	ETS:E-box/HPC7-Scl-ChIP-Seq/Homer	1e-20	-4.687e+01	0.0000	2649.0	6.37%	2102.7	5.31%	motif file (matrix)	pdf
95	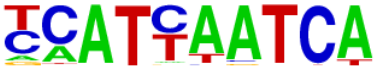	Pdx1(Homeobox)/Islet-Pdx1-ChIP-Seq/Homer	1e-20	-4.679e+01	0.0000	19327.0	46.49%	17494.8	44.21%	motif file (matrix)	pdf
96	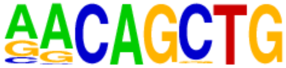	MyoG(HLH)/C2C12-MyoG-ChIP-Seq(GSE36024)/Homer	1e-20	-4.615e+01	0.0000	22509.0	54.15%	20526.2	51.88%	motif file (matrix)	pdf
97	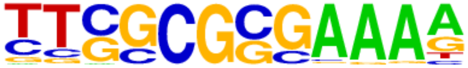	E2F(E2F)/Cell-Cycle-Exp/Homer	1e-20	-4.615e+01	0.0000	1343.0	3				
