# Supplementary figures and images for: Seminoma and Embryonal Carcinoma Footprints Identified by Analysis of Integrated Genome-Wide Epigenetic and Expression Profiles of Germ Cell Cancer Cell Lines (part 2 of 2)
Source: PLoS One. 2014 Jun 2;9(6):e98330. doi: 10.1371/journal.pone.0098330 (PMC4041891; doi:10.1371/journal.pone.0098330)

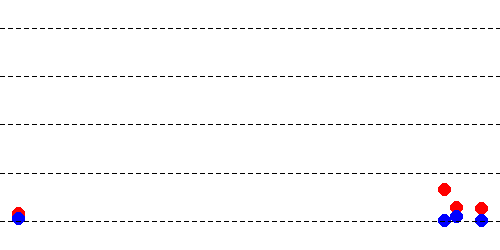

Supplement: File S1 — ZIP file containing DMRforPairs output for significant regions. Please start from the html files. (ZIP) [file pone.0098330.s008.zip › figures/311.png]

RegionID: 311, chr1:23696021-23696209-M\_values

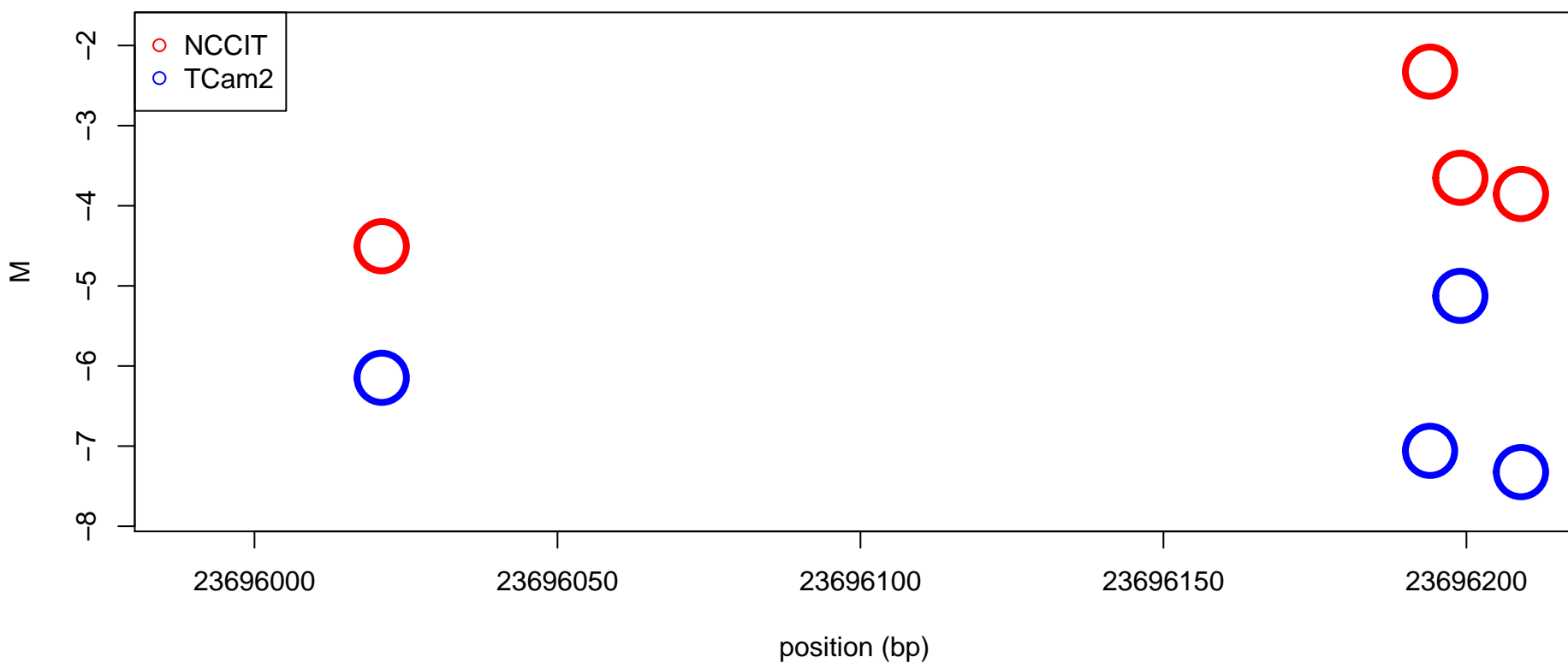

RegionID: 311, chr1:23696021-23696209-Beta\_values

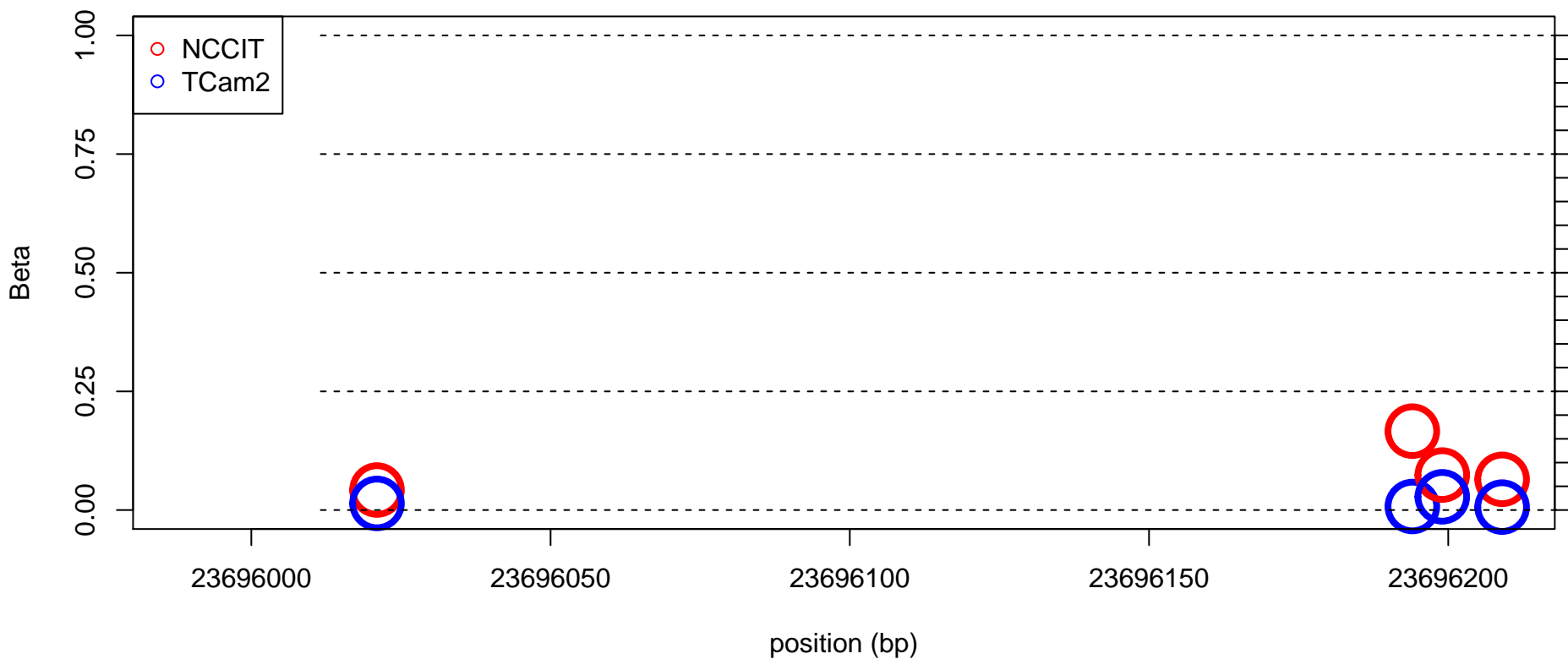

Supplement: File S1 — ZIP file containing DMRforPairs output for significant regions. Please start from the html files. (ZIP) [file pone.0098330.s008.zip › figures/311.pdf]

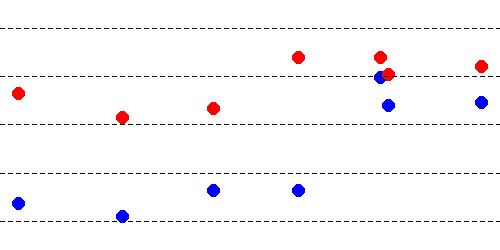

Supplement: File S1 — ZIP file containing DMRforPairs output for significant regions. Please start from the html files. (ZIP) [file pone.0098330.s008.zip › figures/313.png]

RegionID: 313, chr1:23763081-23763959-M\_values

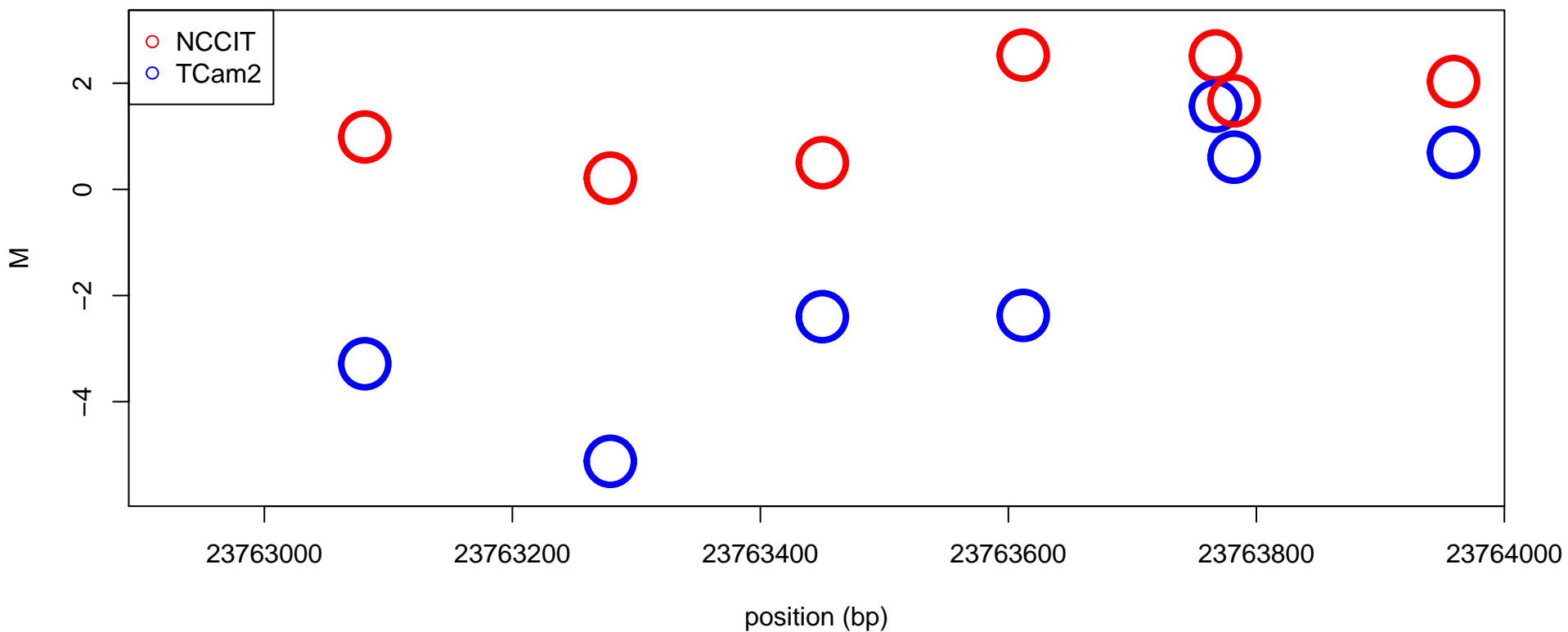

RegionID: 313, chr1:23763081-23763959-Beta\_values

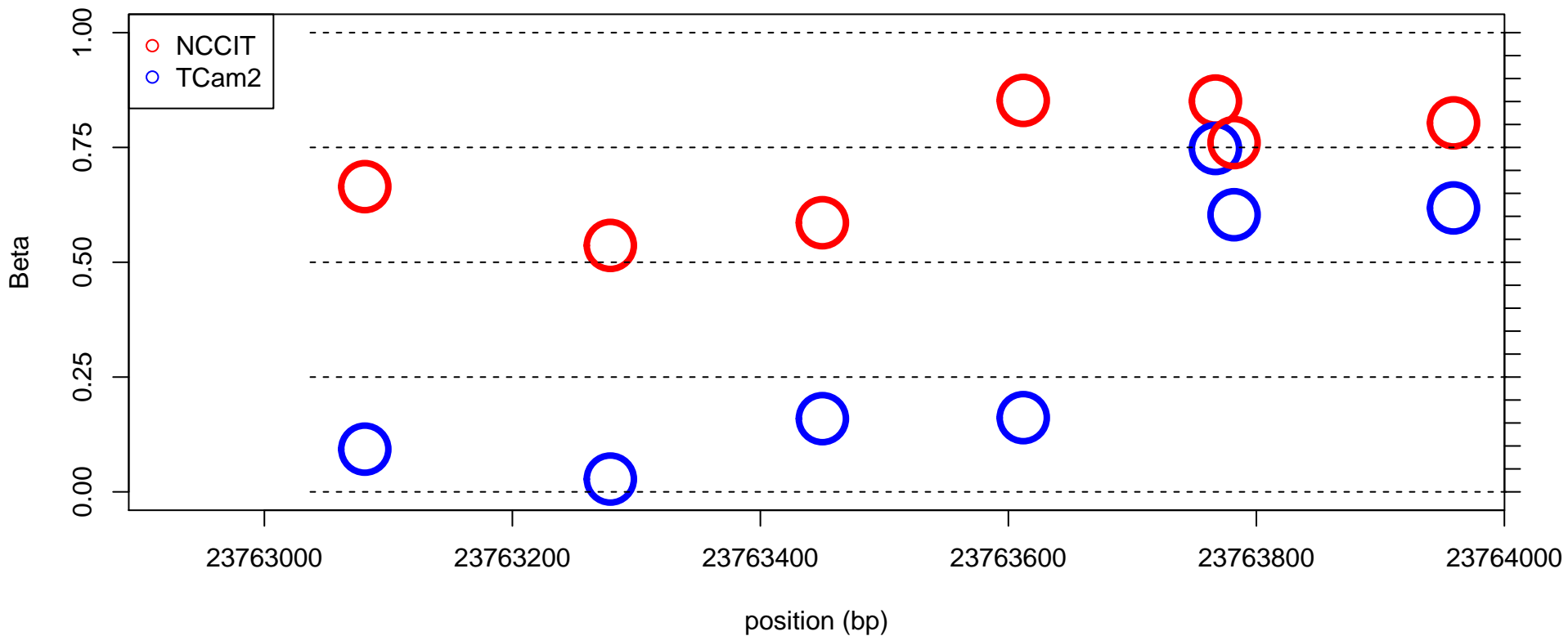

Supplement: File S1 — ZIP file containing DMRforPairs output for significant regions. Please start from the html files. (ZIP) [file pone.0098330.s008.zip › figures/313.pdf]

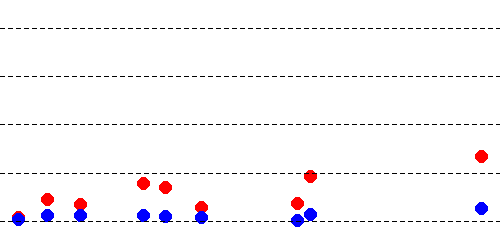

Supplement: File S1 — ZIP file containing DMRforPairs output for significant regions. Please start from the html files. (ZIP) [file pone.0098330.s008.zip › figures/325.png]

RegionID: 325, chr1:24645885–24646392–M\_values

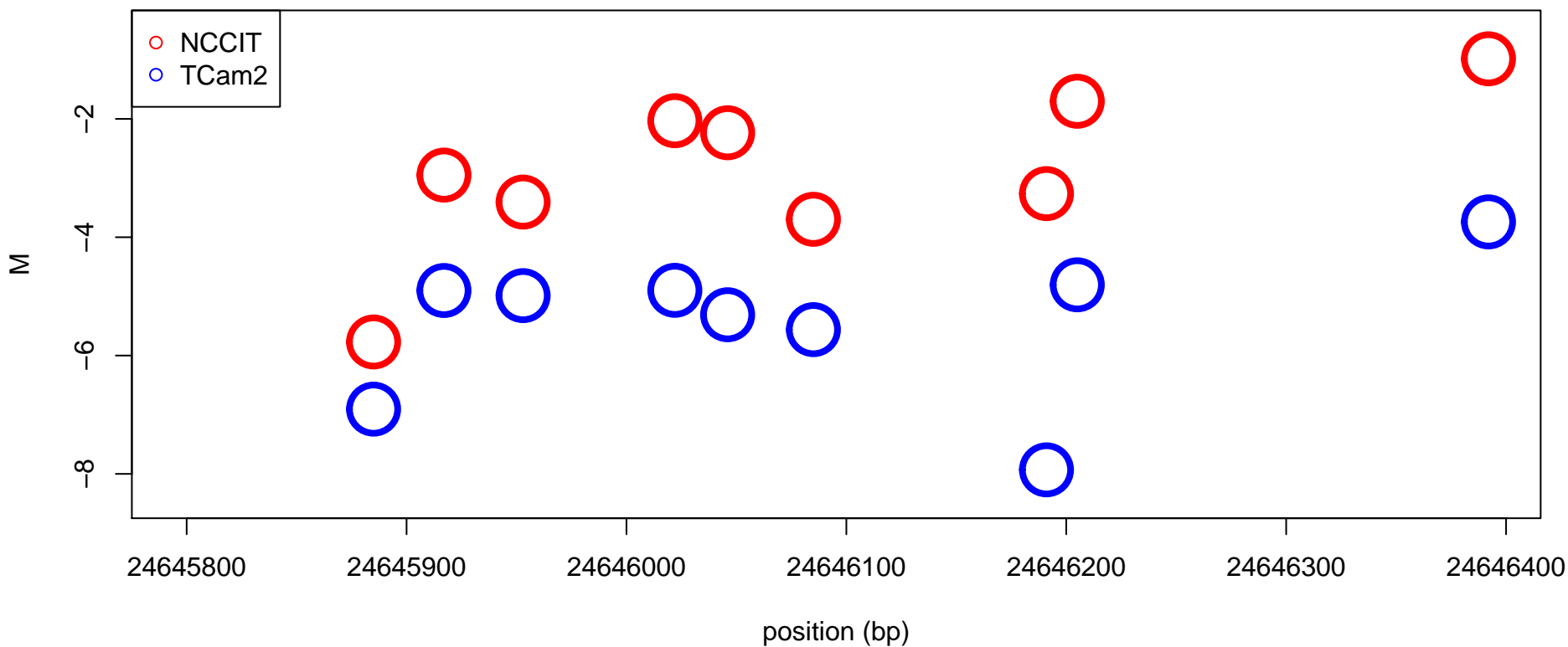

RegionID: 325, chr1:24645885–24646392–Beta\_values

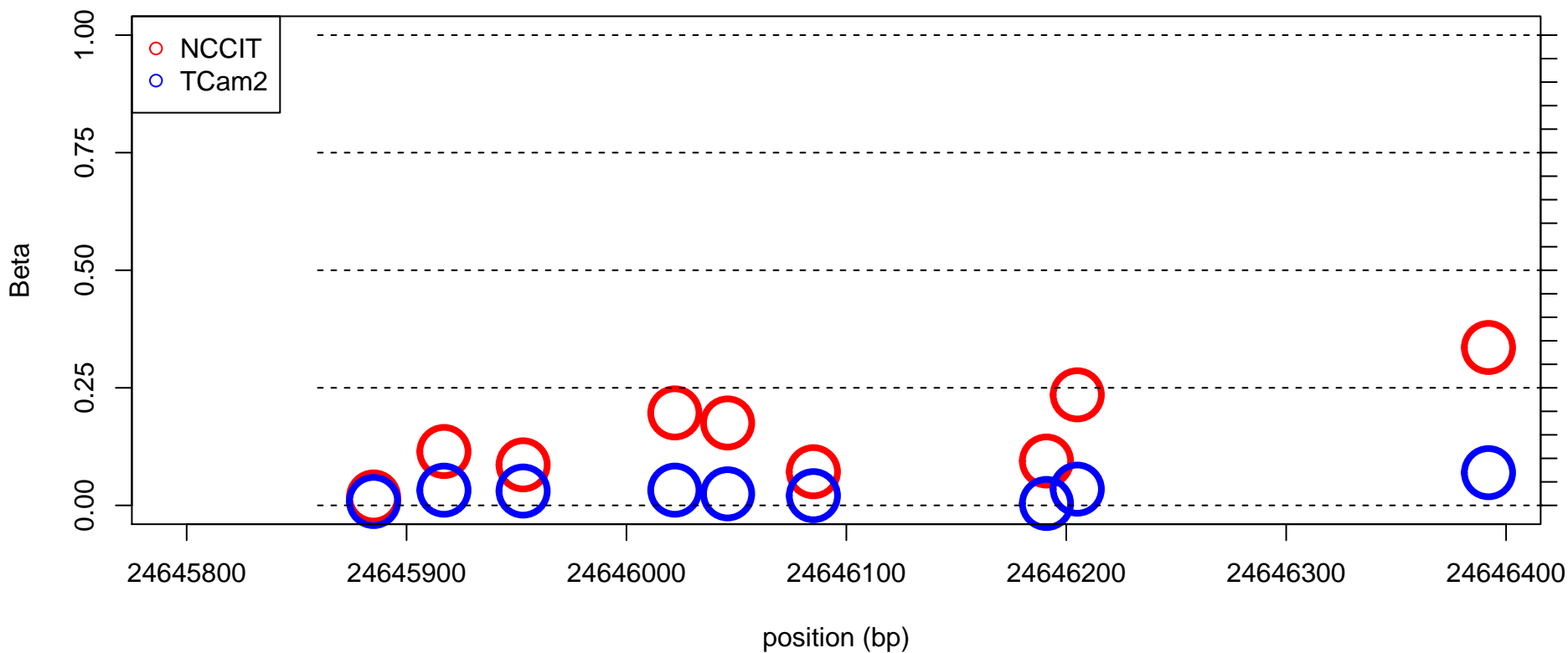

Supplement: File S1 — ZIP file containing DMRforPairs output for significant regions. Please start from the html files. (ZIP) [file pone.0098330.s008.zip › figures/325.pdf]

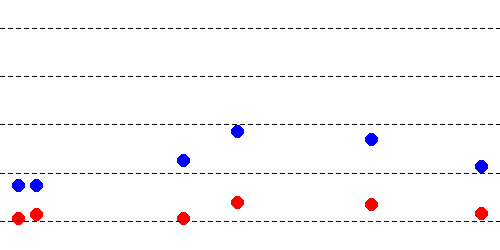

Supplement: File S1 — ZIP file containing DMRforPairs output for significant regions. Please start from the html files. (ZIP) [file pone.0098330.s008.zip › figures/329.png]

RegionID: 329, chr1:25256939–25257191–M\_values

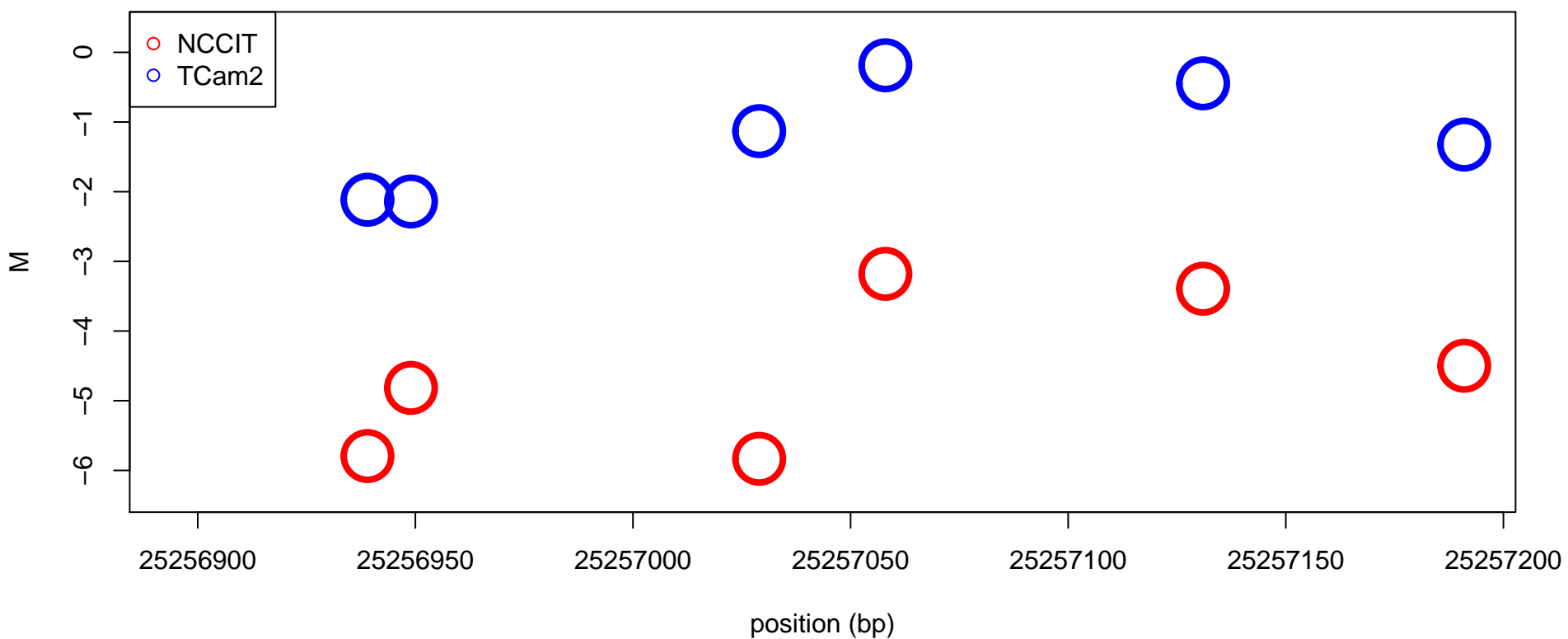

RegionID: 329, chr1:25256939–25257191–Beta\_values

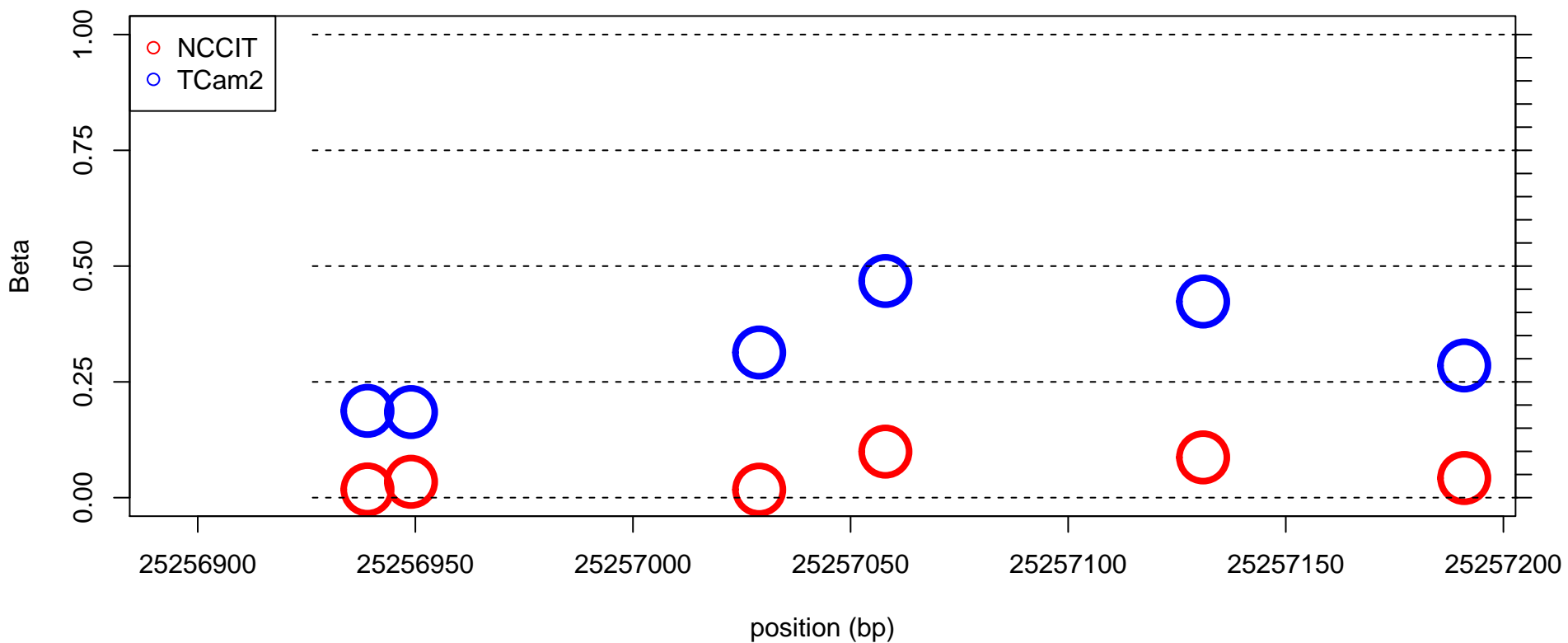

Supplement: File S1 — ZIP file containing DMRforPairs output for significant regions. Please start from the html files. (ZIP) [file pone.0098330.s008.zip › figures/329.pdf]

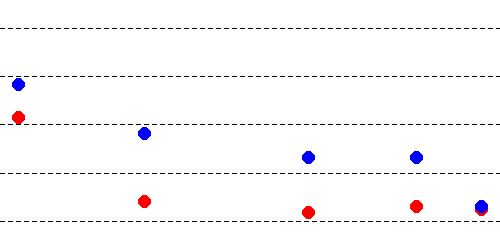

Supplement: File S1 — ZIP file containing DMRforPairs output for significant regions. Please start from the html files. (ZIP) [file pone.0098330.s008.zip › figures/336.png]

RegionID: 336, chr1:26487591–26488129–M\_values

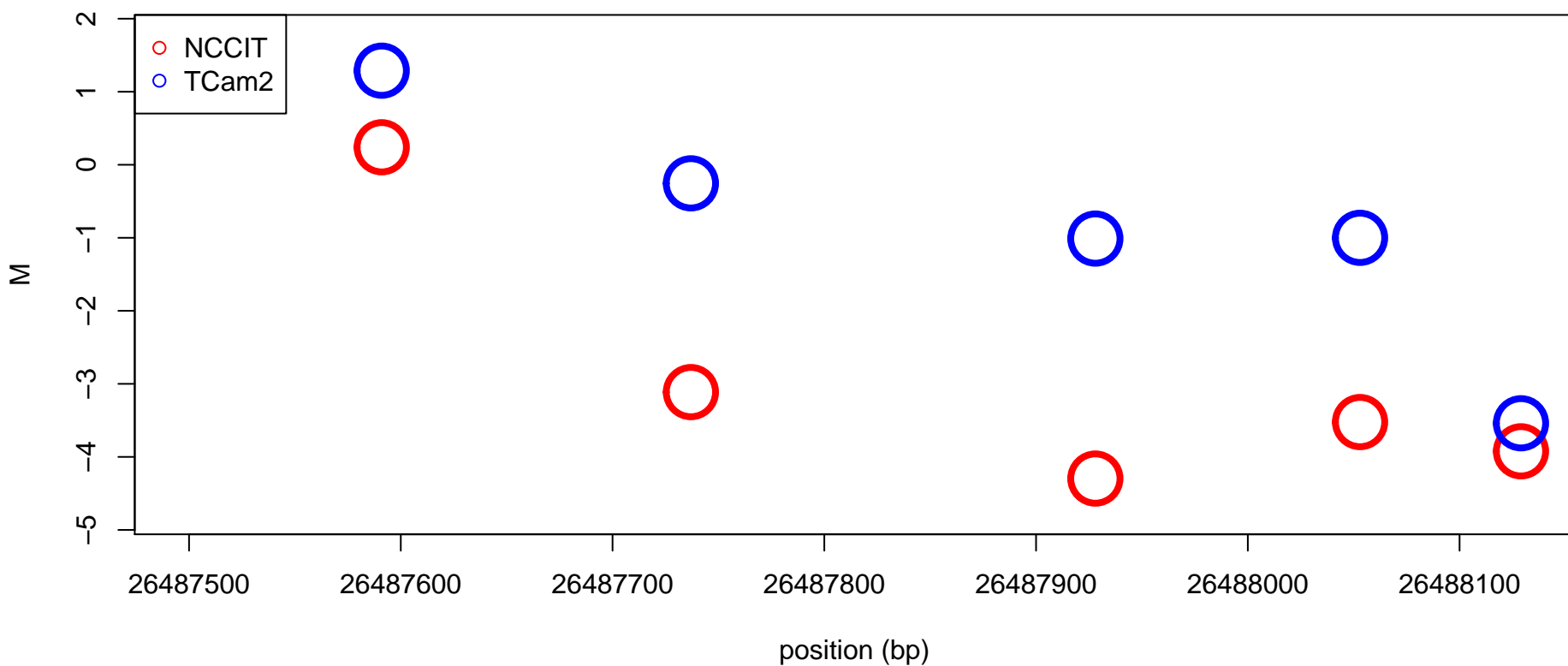

RegionID: 336, chr1:26487591–26488129–Beta\_values

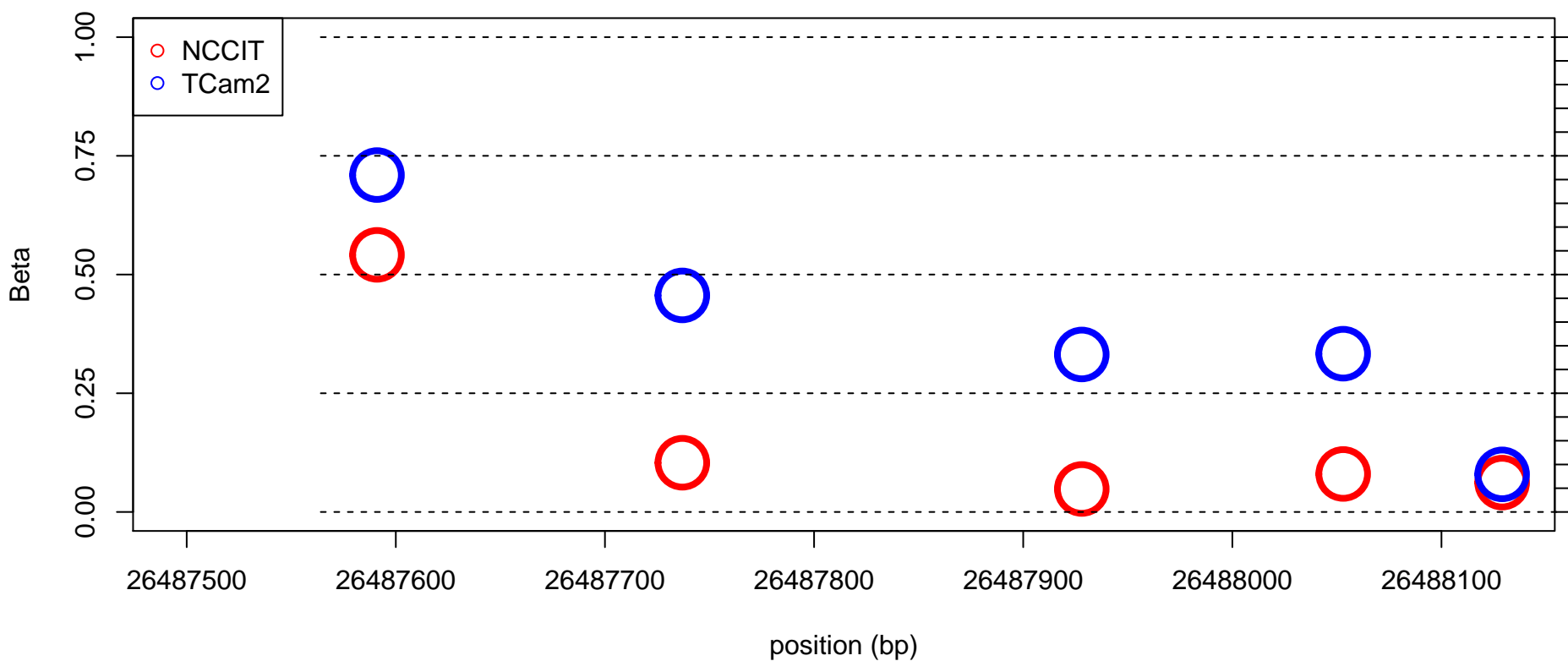

Supplement: File S1 — ZIP file containing DMRforPairs output for significant regions. Please start from the html files. (ZIP) [file pone.0098330.s008.zip › figures/336.pdf]

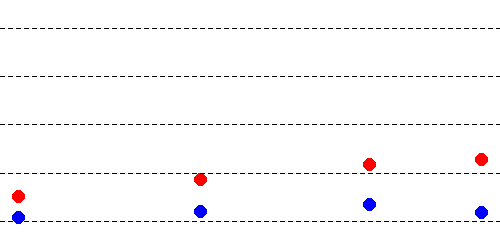

Supplement: File S1 — ZIP file containing DMRforPairs output for significant regions. Please start from the html files. (ZIP) [file pone.0098330.s008.zip › figures/337.png]

RegionID: 337, chr1:26606846–26606912–M\_values

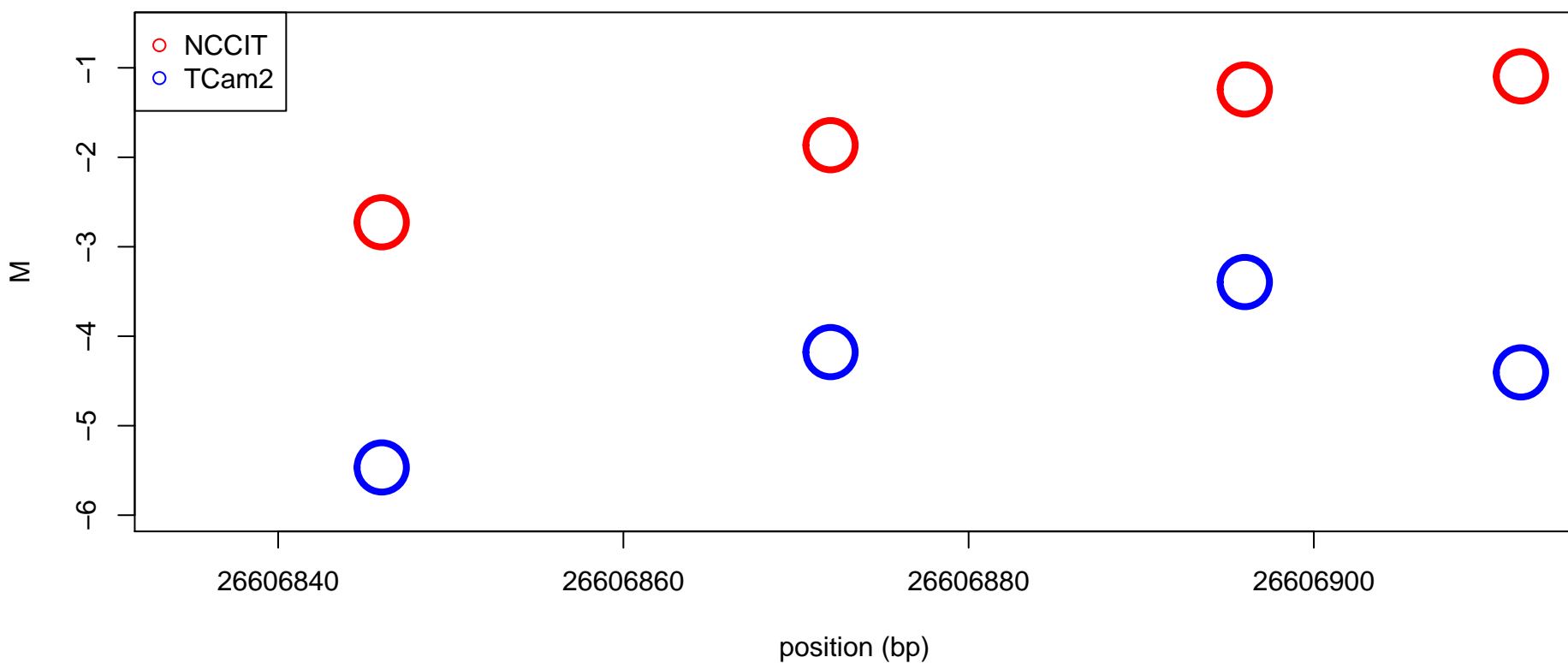

RegionID: 337, chr1:26606846–26606912–Beta\_values

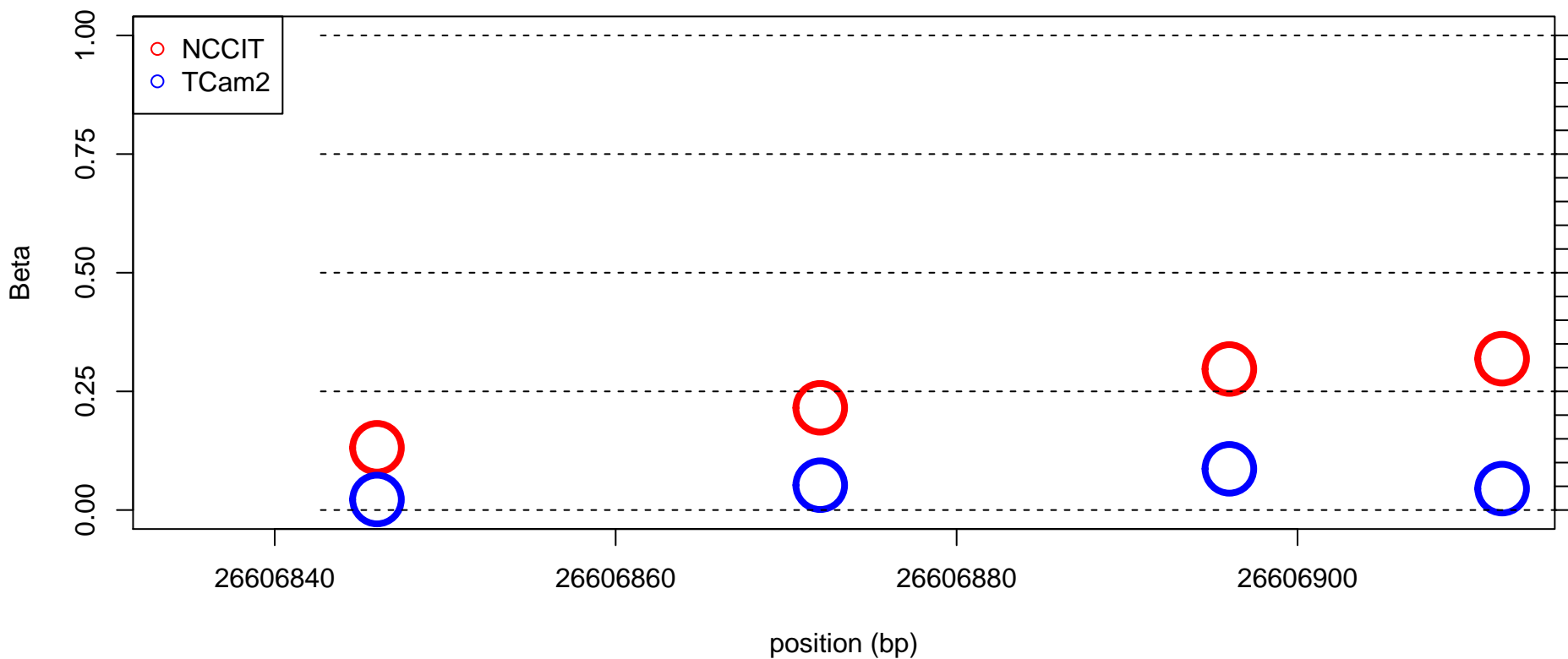

Supplement: File S1 — ZIP file containing DMRforPairs output for significant regions. Please start from the html files. (ZIP) [file pone.0098330.s008.zip › figures/337.pdf]

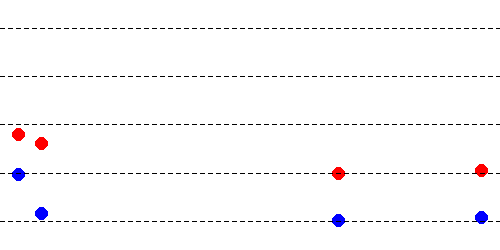

Supplement: File S1 — ZIP file containing DMRforPairs output for significant regions. Please start from the html files. (ZIP) [file pone.0098330.s008.zip › figures/341.png]

RegionID: 341, chr1:26856408–26856648–M\_values

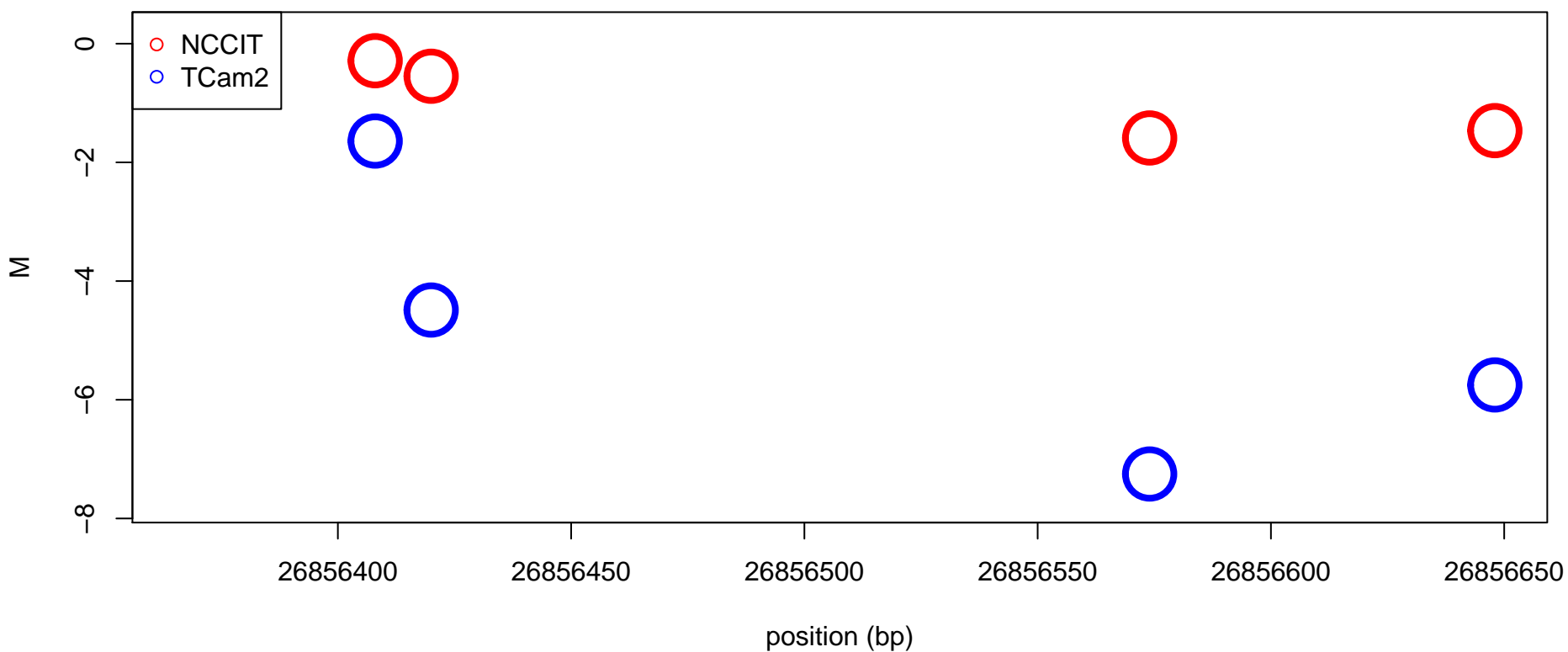

RegionID: 341, chr1:26856408–26856648–Beta\_values

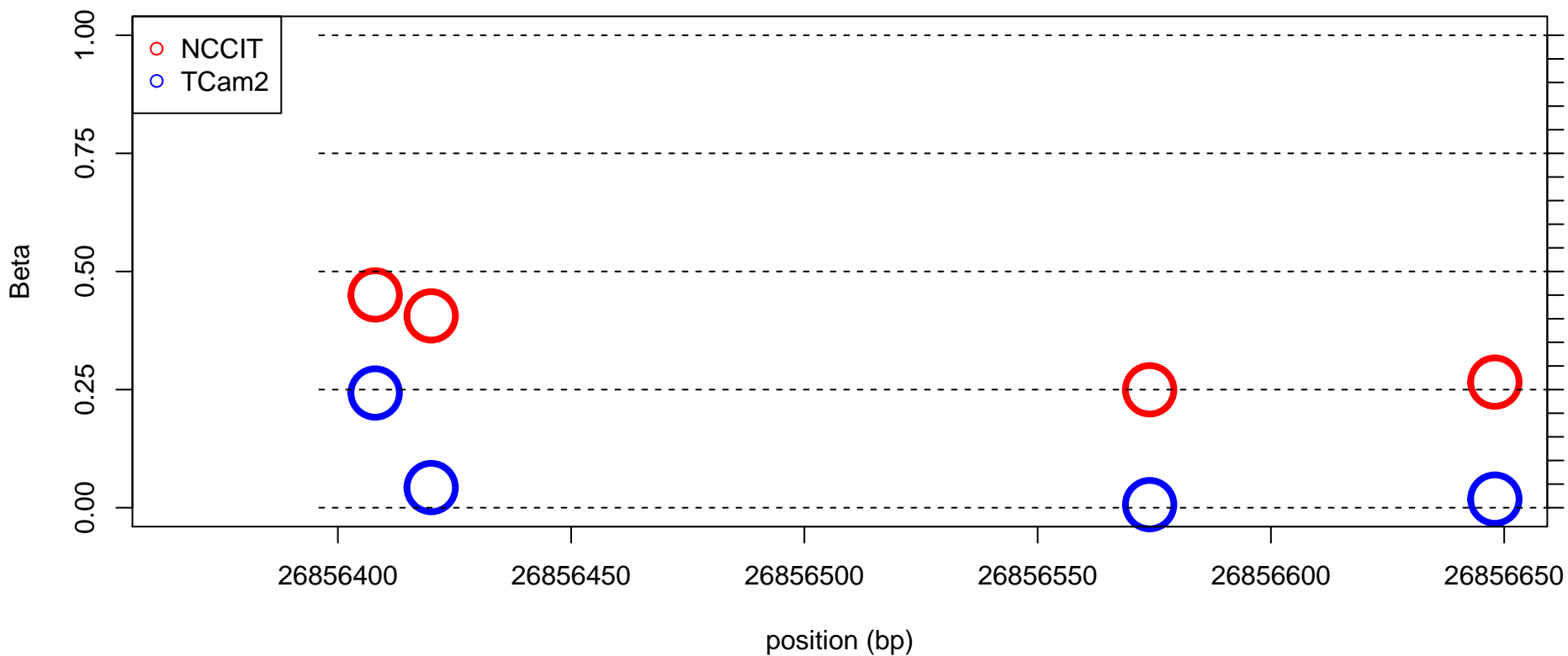

Supplement: File S1 — ZIP file containing DMRforPairs output for significant regions. Please start from the html files. (ZIP) [file pone.0098330.s008.zip › figures/341.pdf]

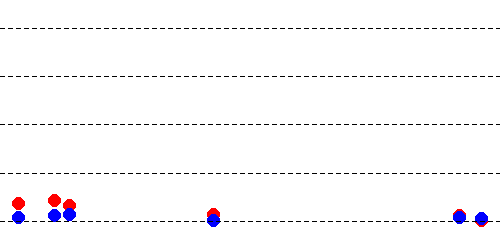

Supplement: File S1 — ZIP file containing DMRforPairs output for significant regions. Please start from the html files. (ZIP) [file pone.0098330.s008.zip › figures/345.png]

RegionID: 345, chr1:27216732-27216796-M\_values

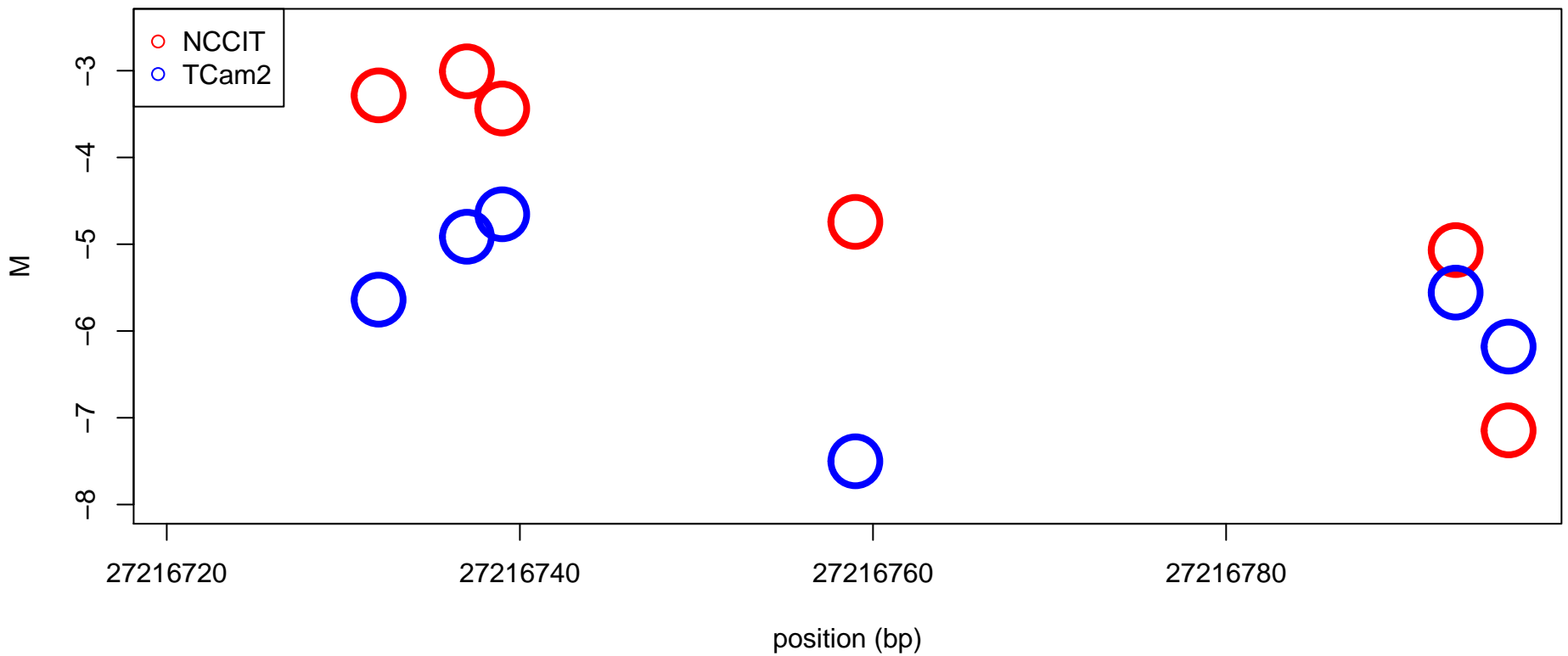

RegionID: 345, chr1:27216732-27216796-Beta\_values

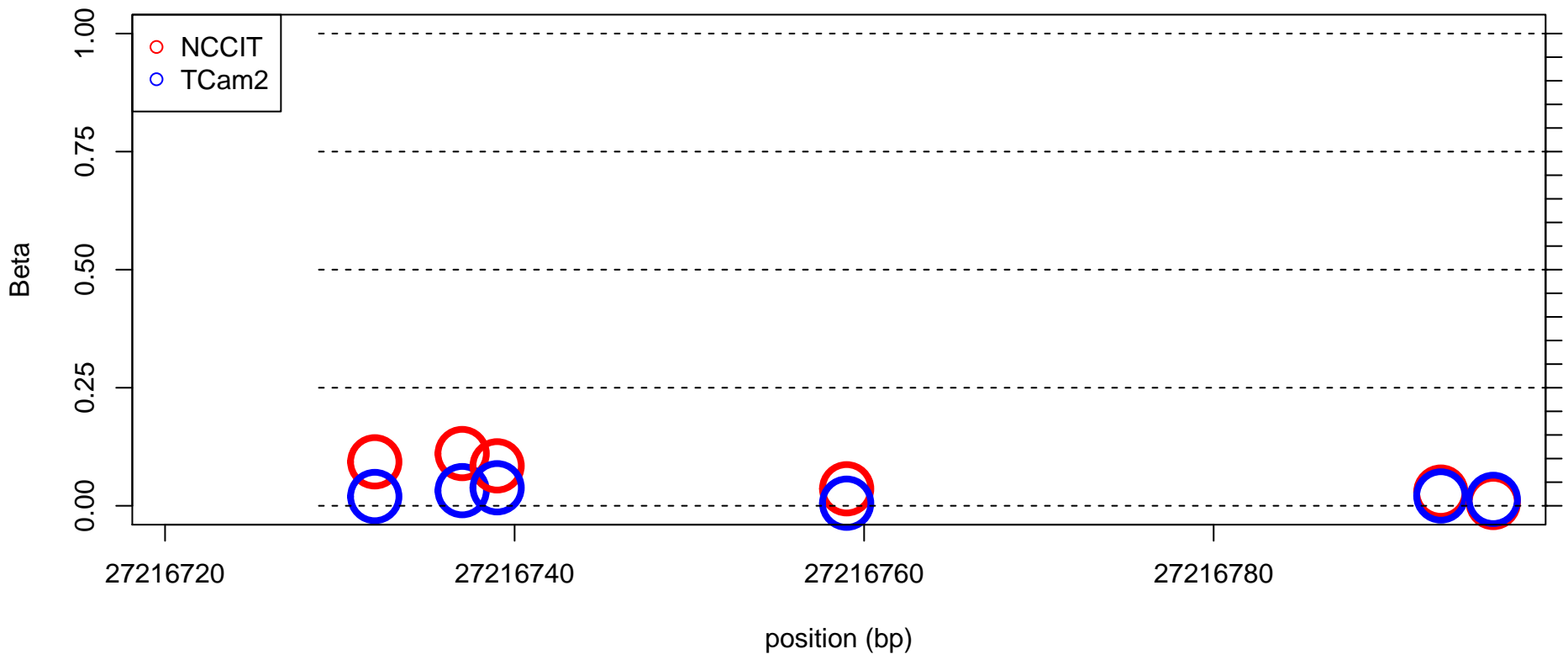

Supplement: File S1 — ZIP file containing DMRforPairs output for significant regions. Please start from the html files. (ZIP) [file pone.0098330.s008.zip › figures/345.pdf]

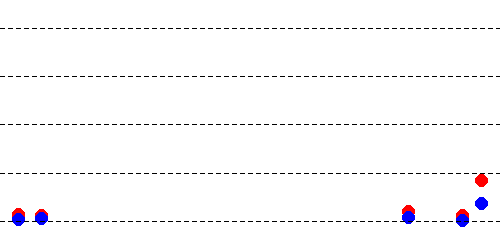

Supplement: File S1 — ZIP file containing DMRforPairs output for significant regions. Please start from the html files. (ZIP) [file pone.0098330.s008.zip › figures/362.png]

RegionID: 362, chr1:28414998–28415118–M\_values

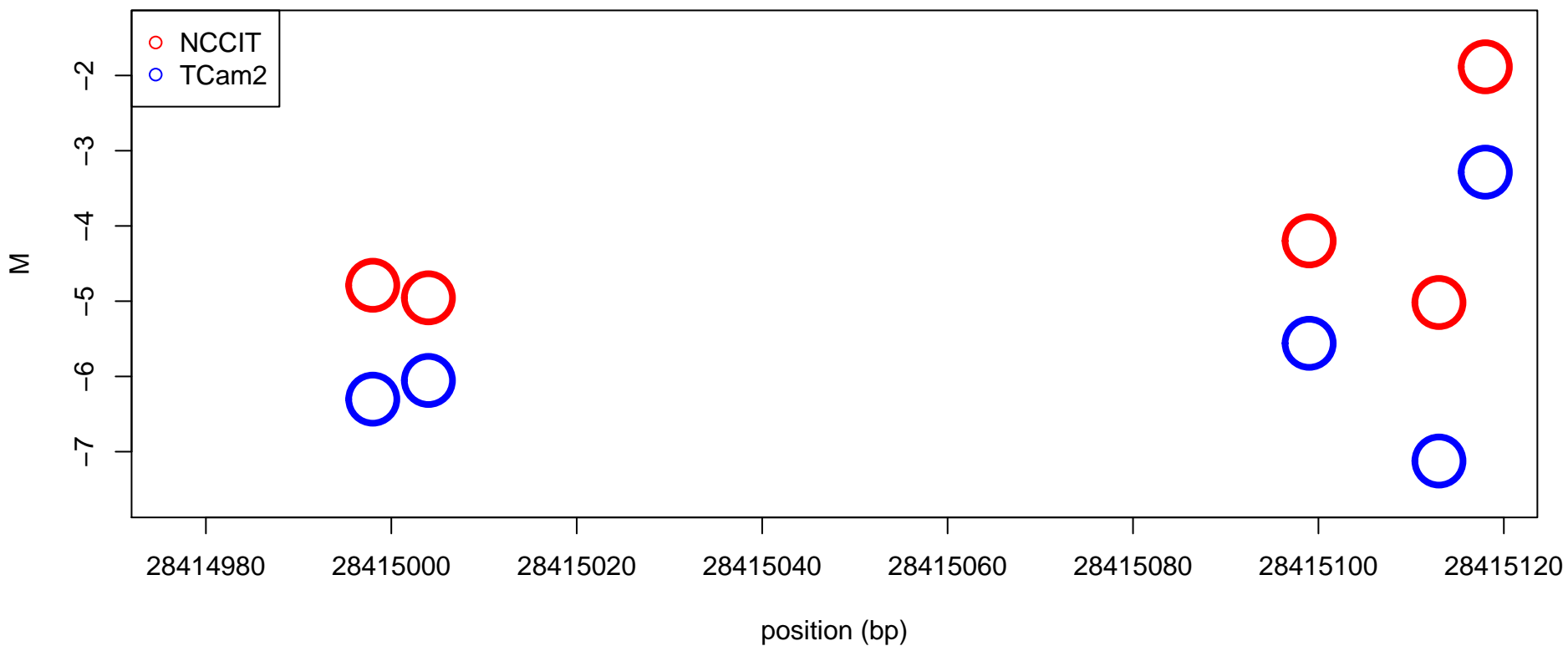

RegionID: 362, chr1:28414998–28415118–Beta\_values

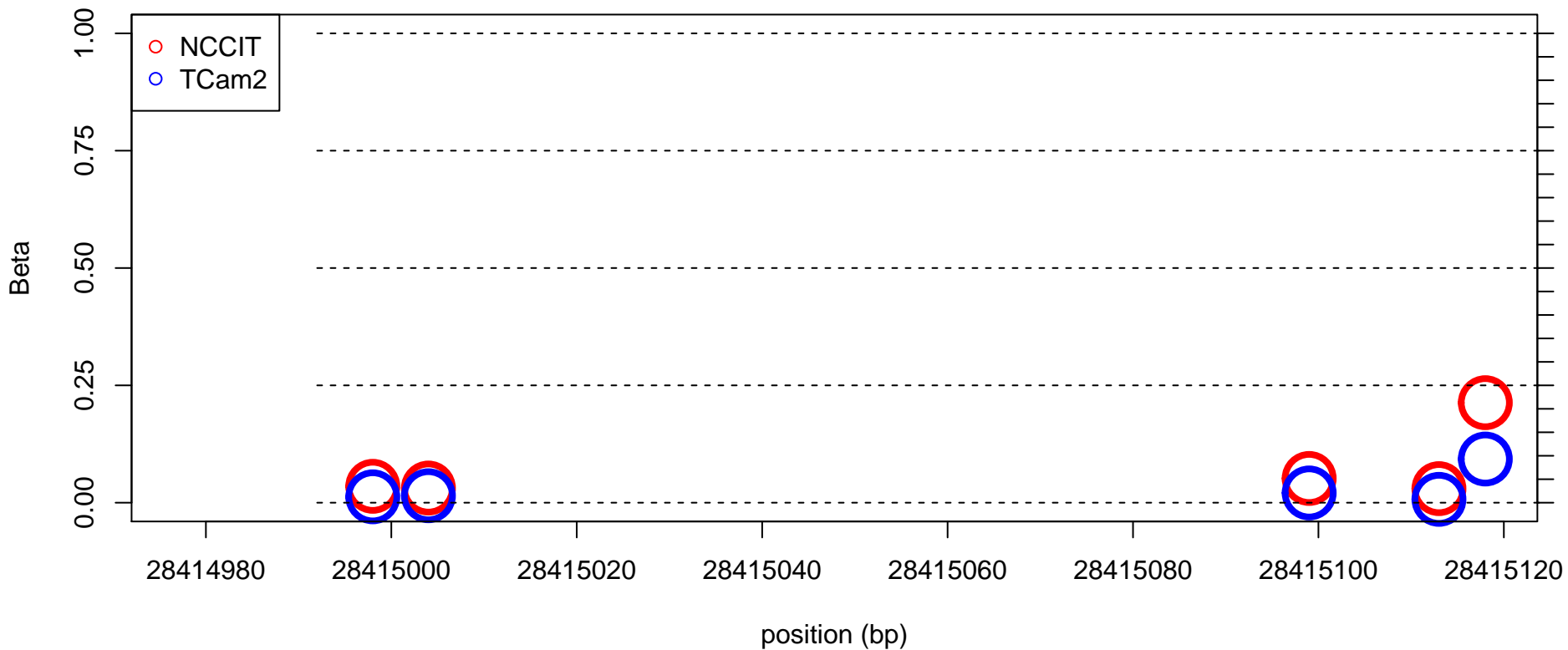

Supplement: File S1 — ZIP file containing DMRforPairs output for significant regions. Please start from the html files. (ZIP) [file pone.0098330.s008.zip › figures/362.pdf]

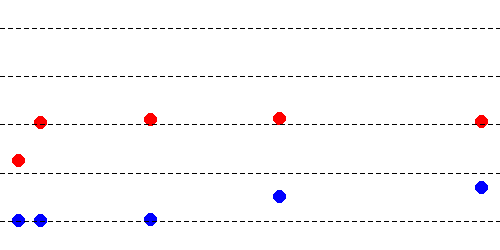

Supplement: File S1 — ZIP file containing DMRforPairs output for significant regions. Please start from the html files. (ZIP) [file pone.0098330.s008.zip › figures/370.png]

RegionID: 370, chr1:29138919–29139278–M\_values

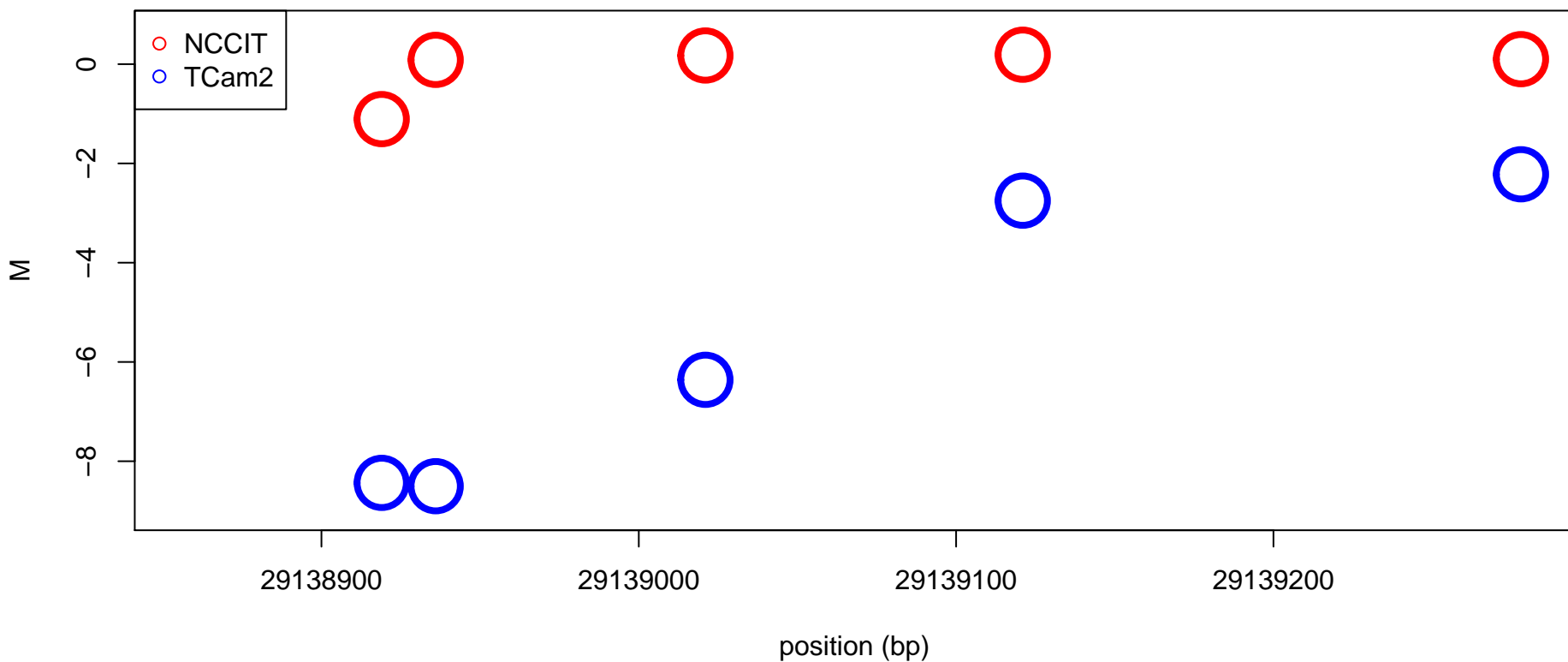

RegionID: 370, chr1:29138919–29139278–Beta\_values

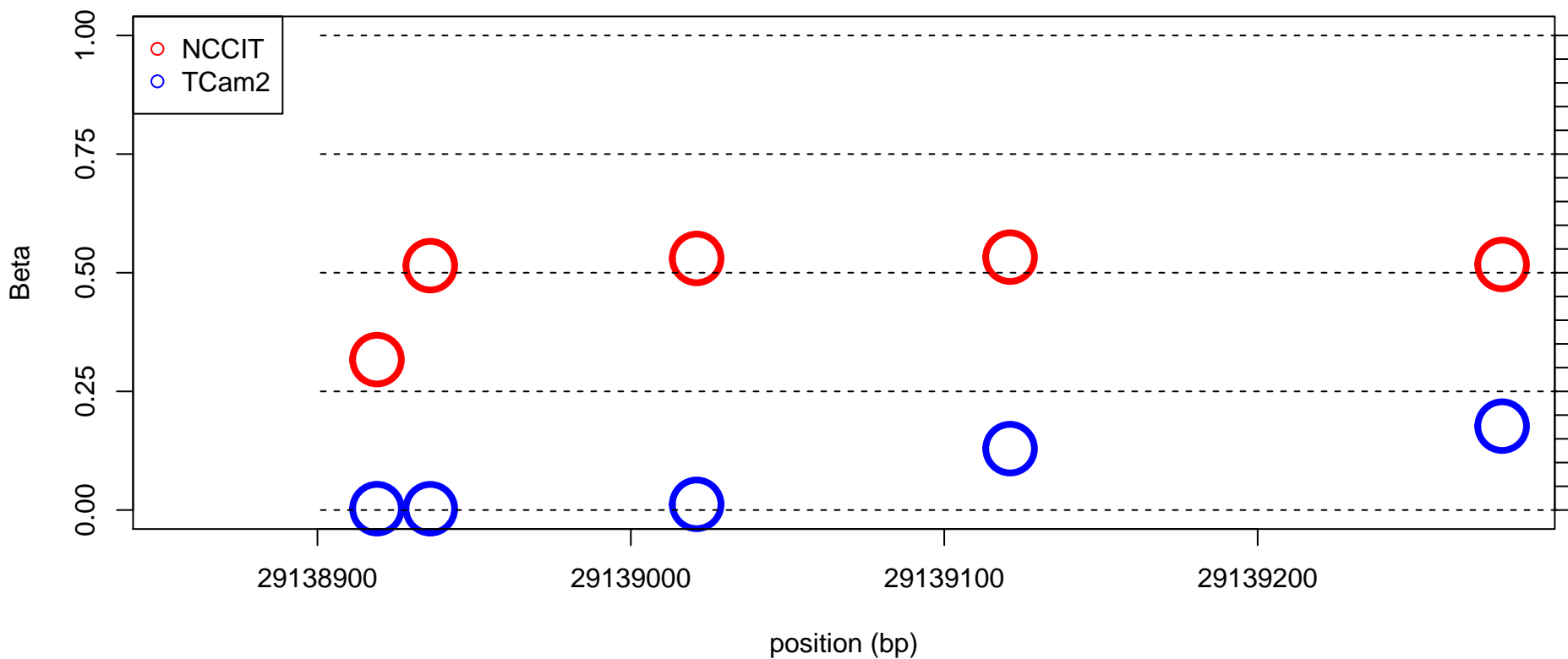

Supplement: File S1 — ZIP file containing DMRforPairs output for significant regions. Please start from the html files. (ZIP) [file pone.0098330.s008.zip › figures/370.pdf]

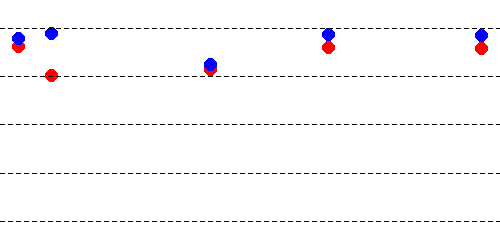

Supplement: File S1 — ZIP file containing DMRforPairs output for significant regions. Please start from the html files. (ZIP) [file pone.0098330.s008.zip › figures/376.png]

RegionID: 376, chr1:29587088–29587490–M\_values

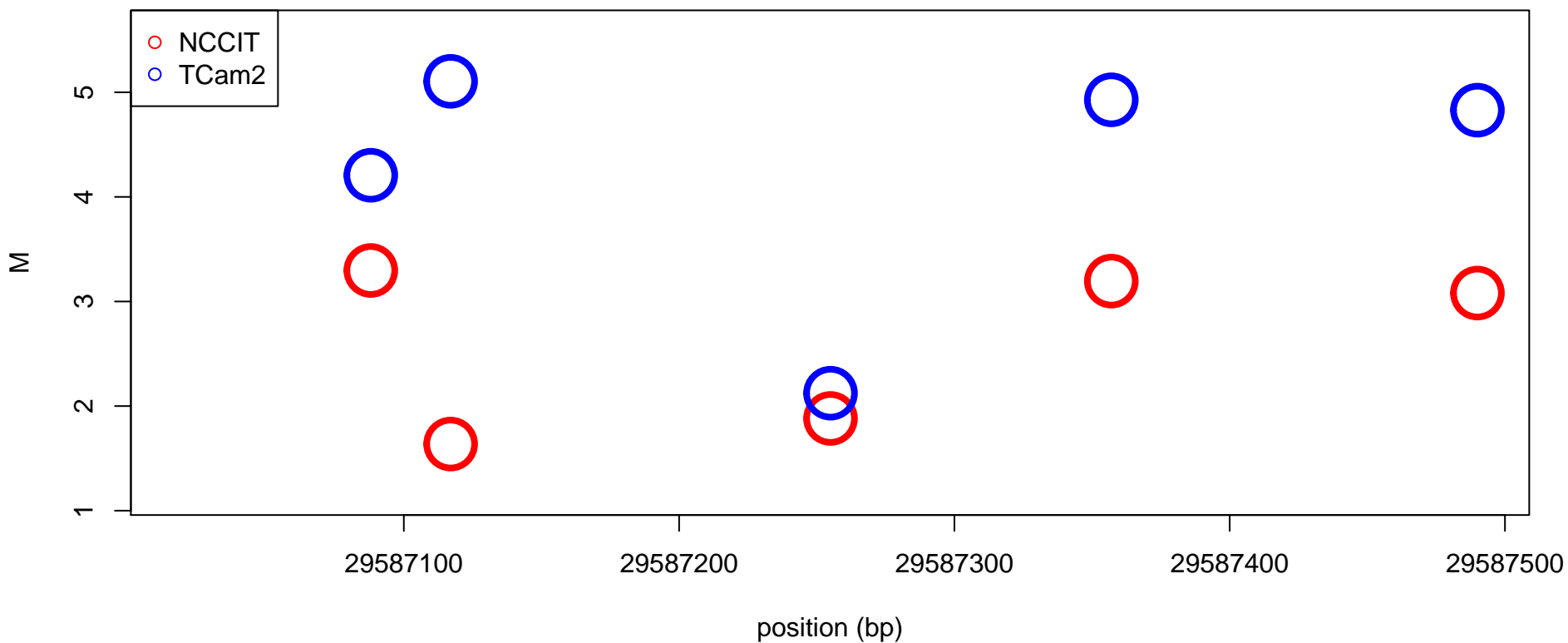

RegionID: 376, chr1:29587088–29587490–Beta\_values

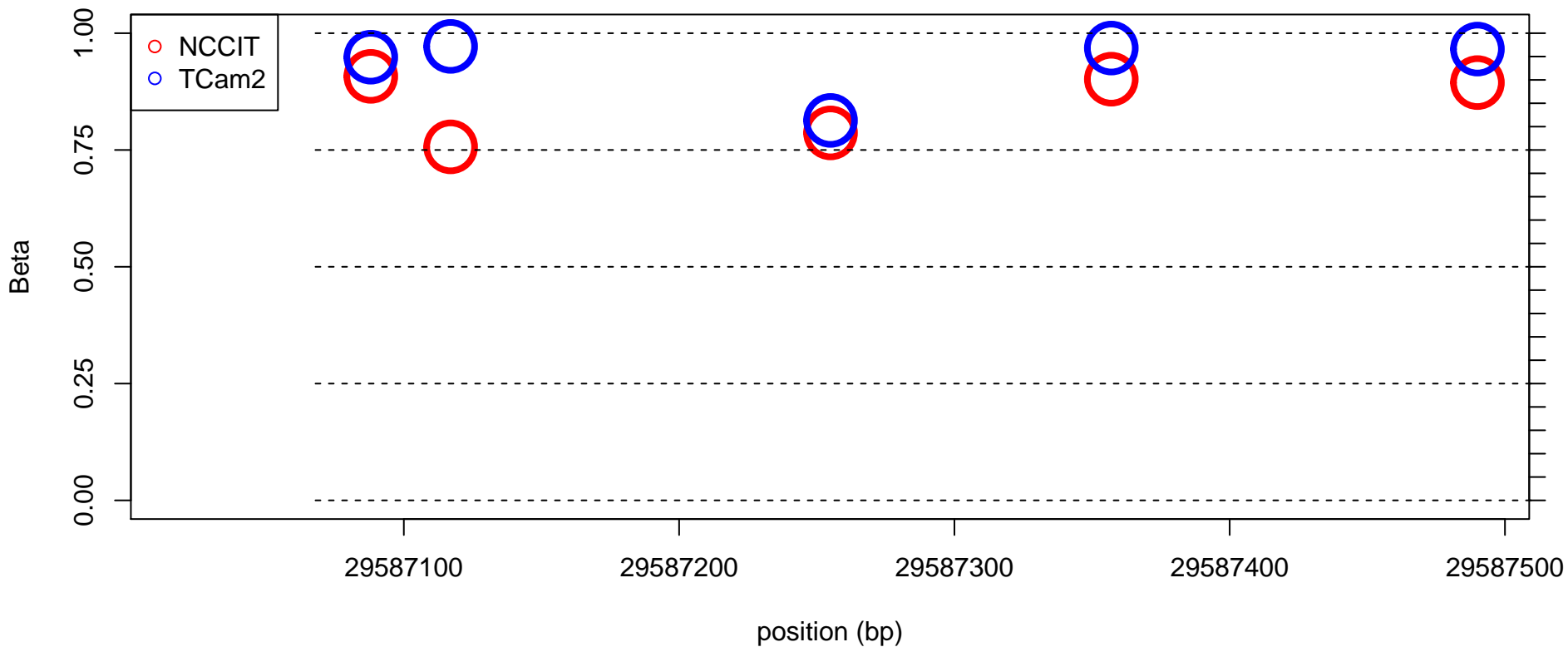

Supplement: File S1 — ZIP file containing DMRforPairs output for significant regions. Please start from the html files. (ZIP) [file pone.0098330.s008.zip › figures/376.pdf]

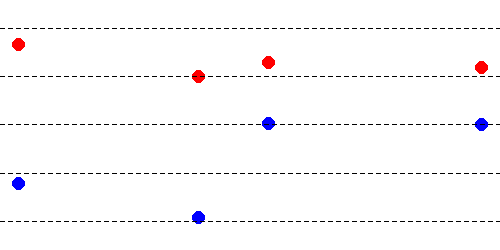

Supplement: File S1 — ZIP file containing DMRforPairs output for significant regions. Please start from the html files. (ZIP) [file pone.0098330.s008.zip › figures/392.png]

RegionID: 392, chr1:32741454–32741619–M\_values

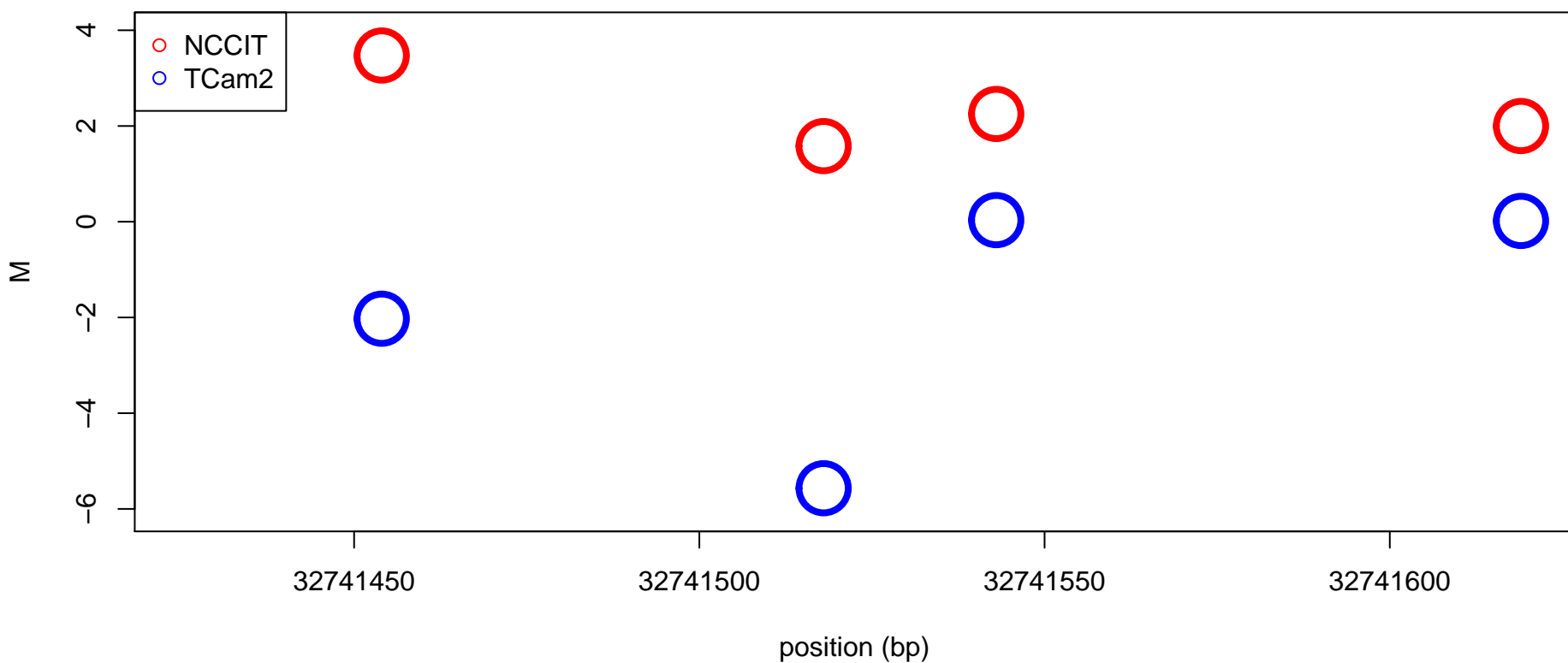

RegionID: 392, chr1:32741454–32741619–Beta\_values

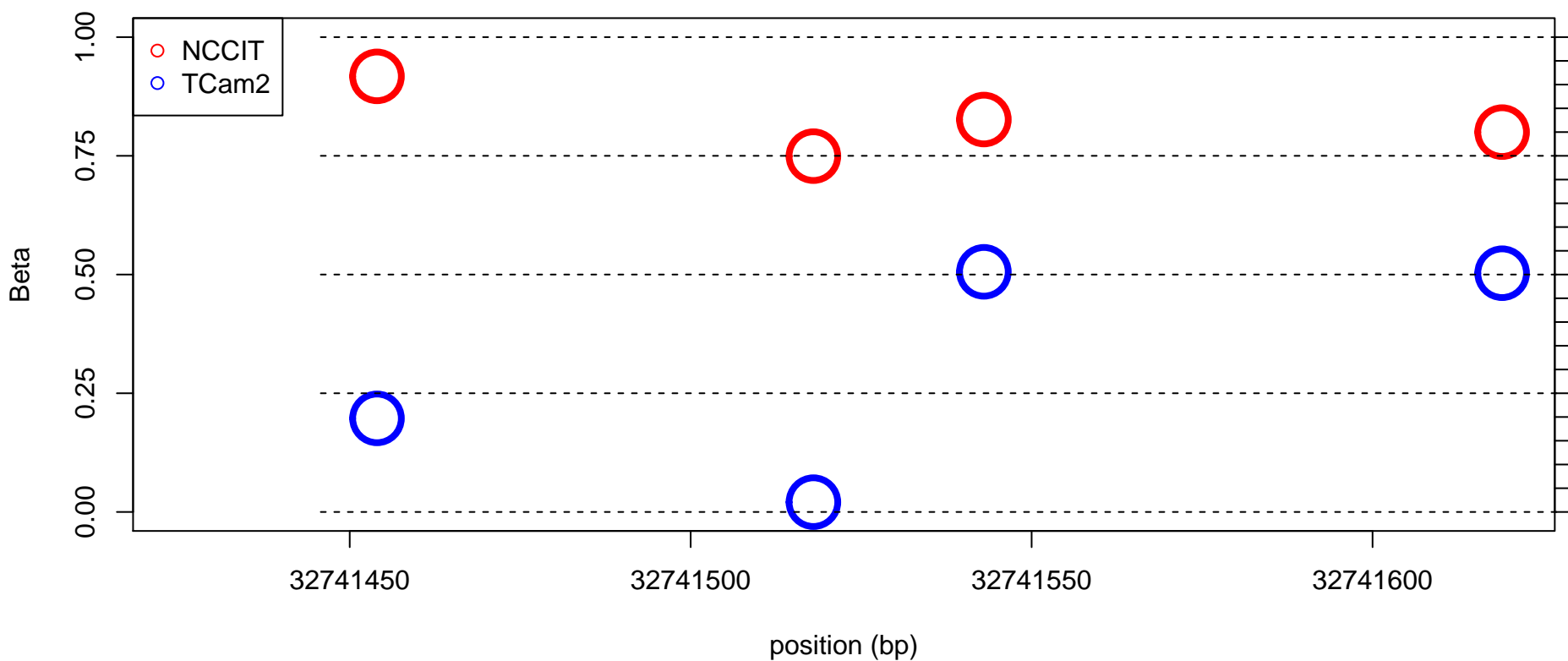

Supplement: File S1 — ZIP file containing DMRforPairs output for significant regions. Please start from the html files. (ZIP) [file pone.0098330.s008.zip › figures/392.pdf]

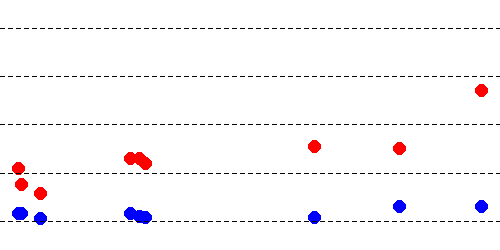

Supplement: File S1 — ZIP file containing DMRforPairs output for significant regions. Please start from the html files. (ZIP) [file pone.0098330.s008.zip › figures/394.png]

RegionID: 394, chr1:32827707-32828191-M\_values

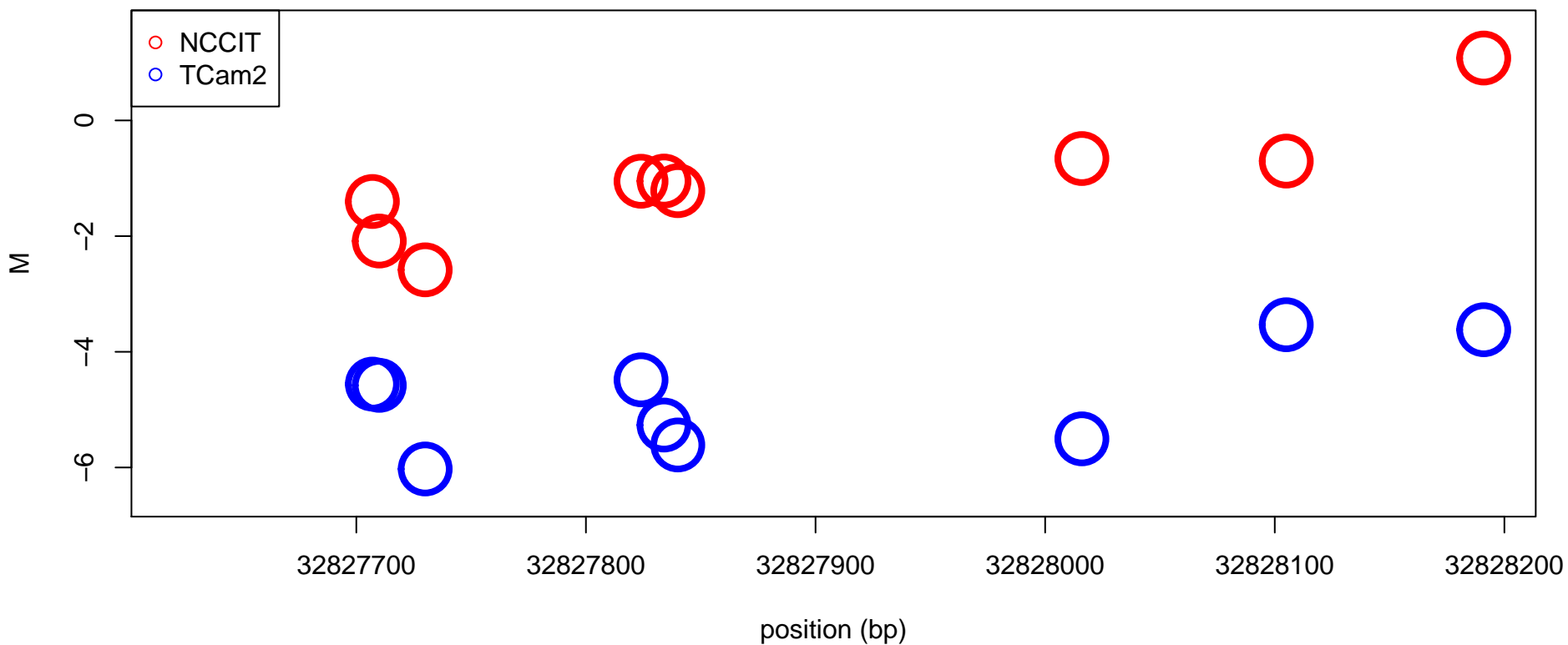

RegionID: 394, chr1:32827707-32828191-Beta\_values

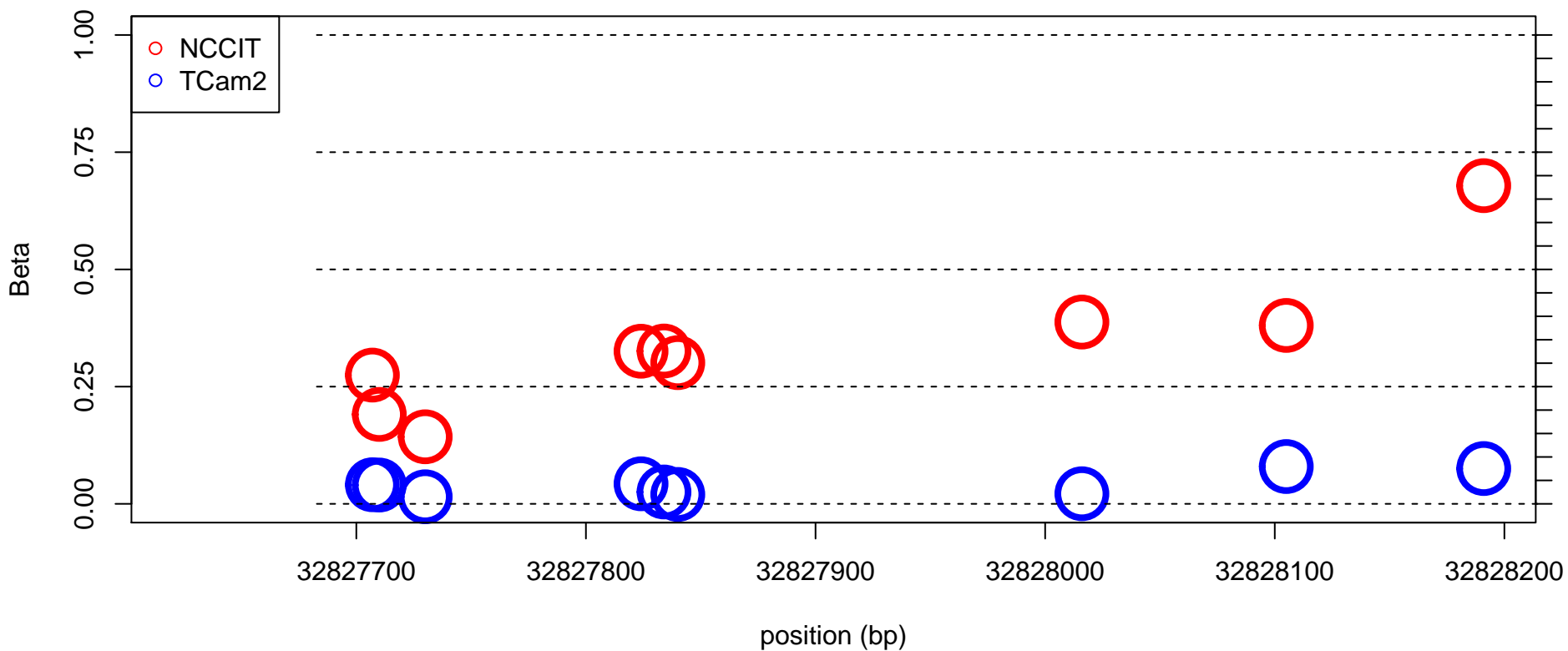

Supplement: File S1 — ZIP file containing DMRforPairs output for significant regions. Please start from the html files. (ZIP) [file pone.0098330.s008.zip › figures/394.pdf]

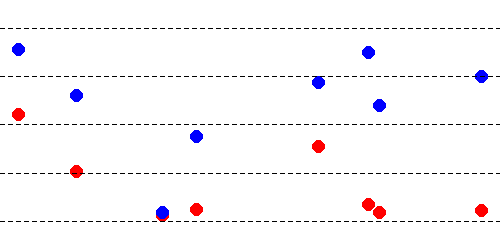

Supplement: File S1 — ZIP file containing DMRforPairs output for significant regions. Please start from the html files. (ZIP) [file pone.0098330.s008.zip › figures/398.png]

RegionID: 398, chr1:33231070–33231382–M\_values

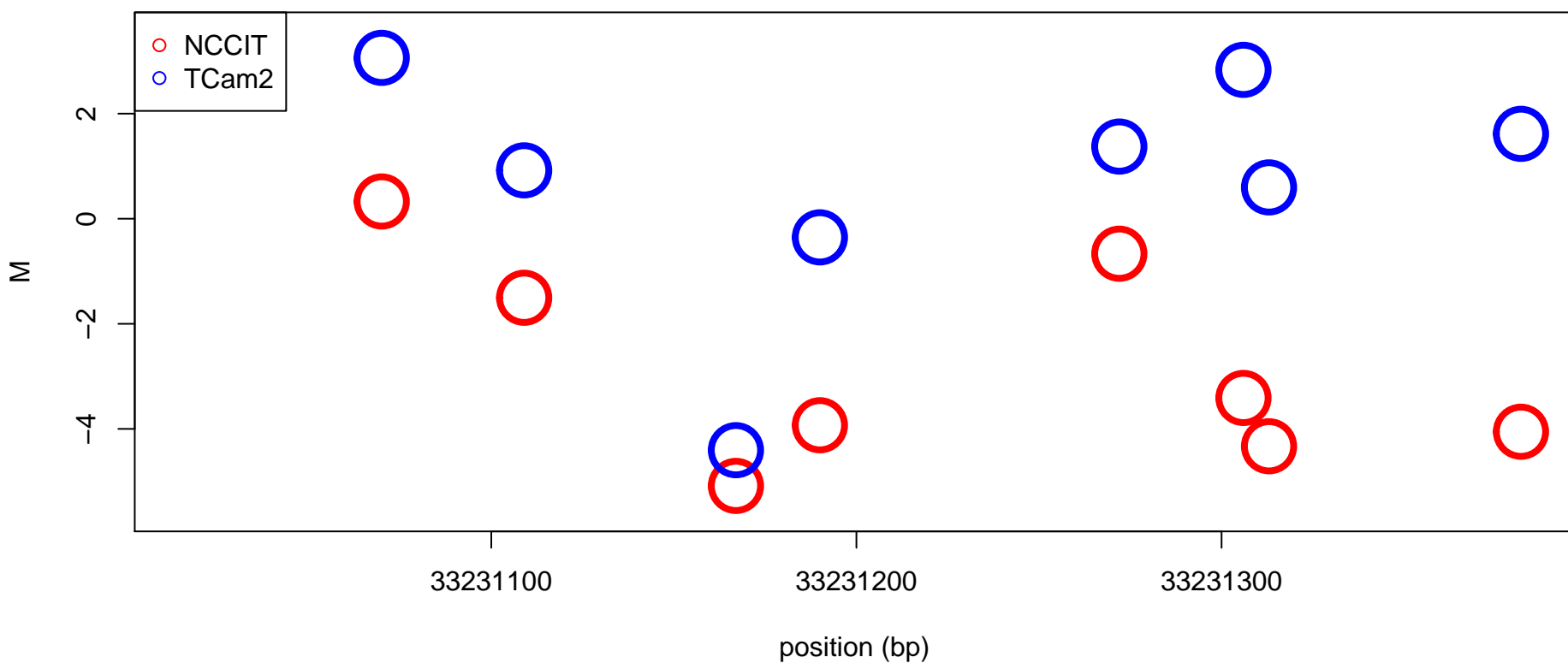

RegionID: 398, chr1:33231070–33231382–Beta\_values

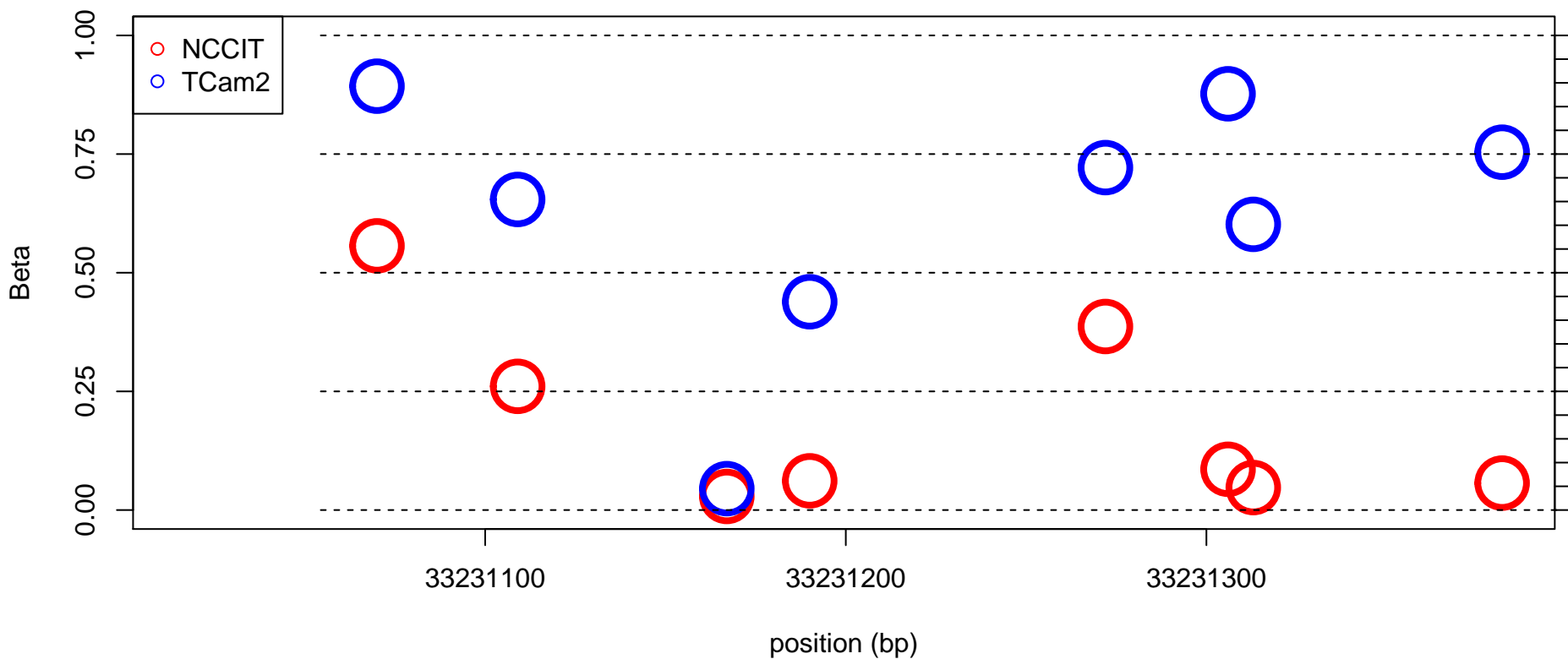

Supplement: File S1 — ZIP file containing DMRforPairs output for significant regions. Please start from the html files. (ZIP) [file pone.0098330.s008.zip › figures/398.pdf]

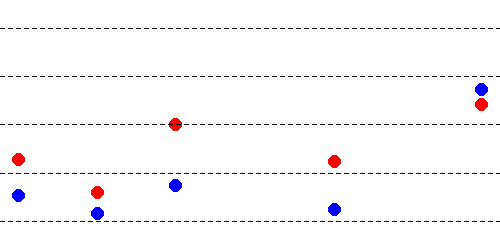

Supplement: File S1 — ZIP file containing DMRforPairs output for significant regions. Please start from the html files. (ZIP) [file pone.0098330.s008.zip › figures/404.png]

RegionID: 404, chr1:34090664–34090941–M\_values

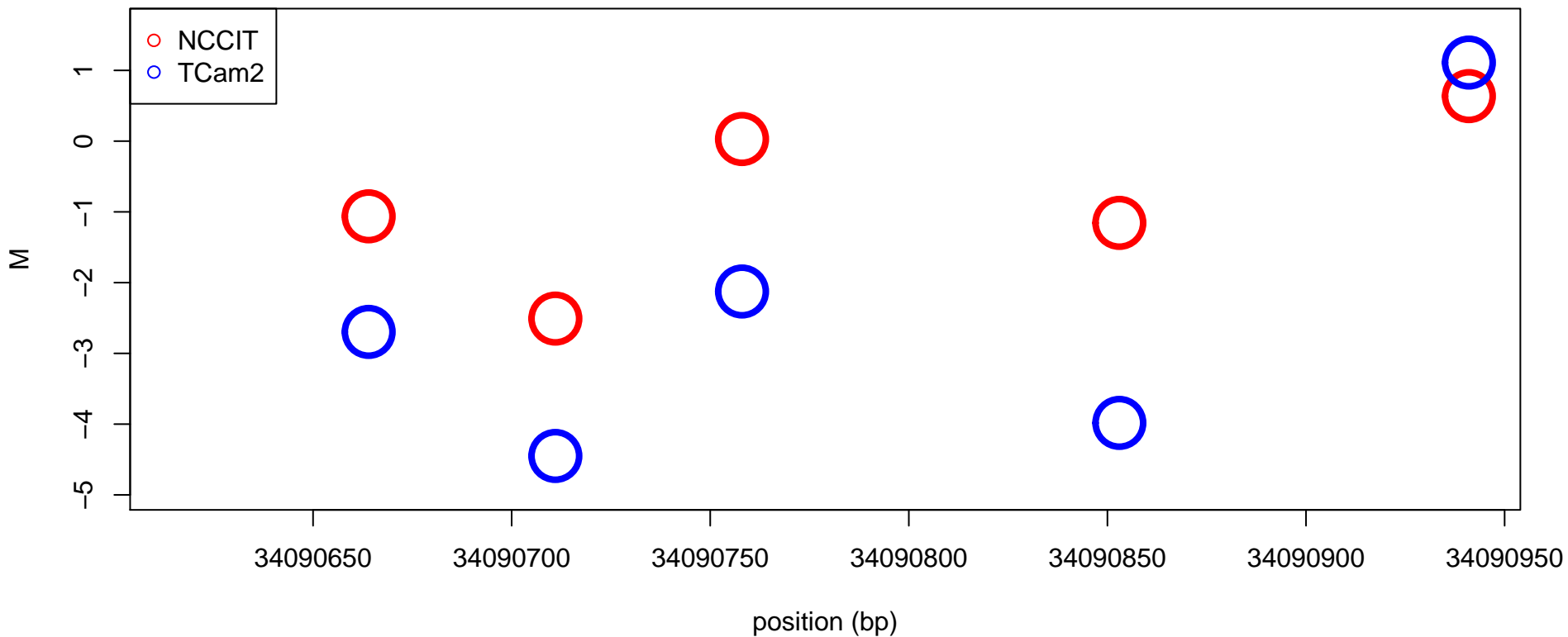

RegionID: 404, chr1:34090664–34090941–Beta\_values

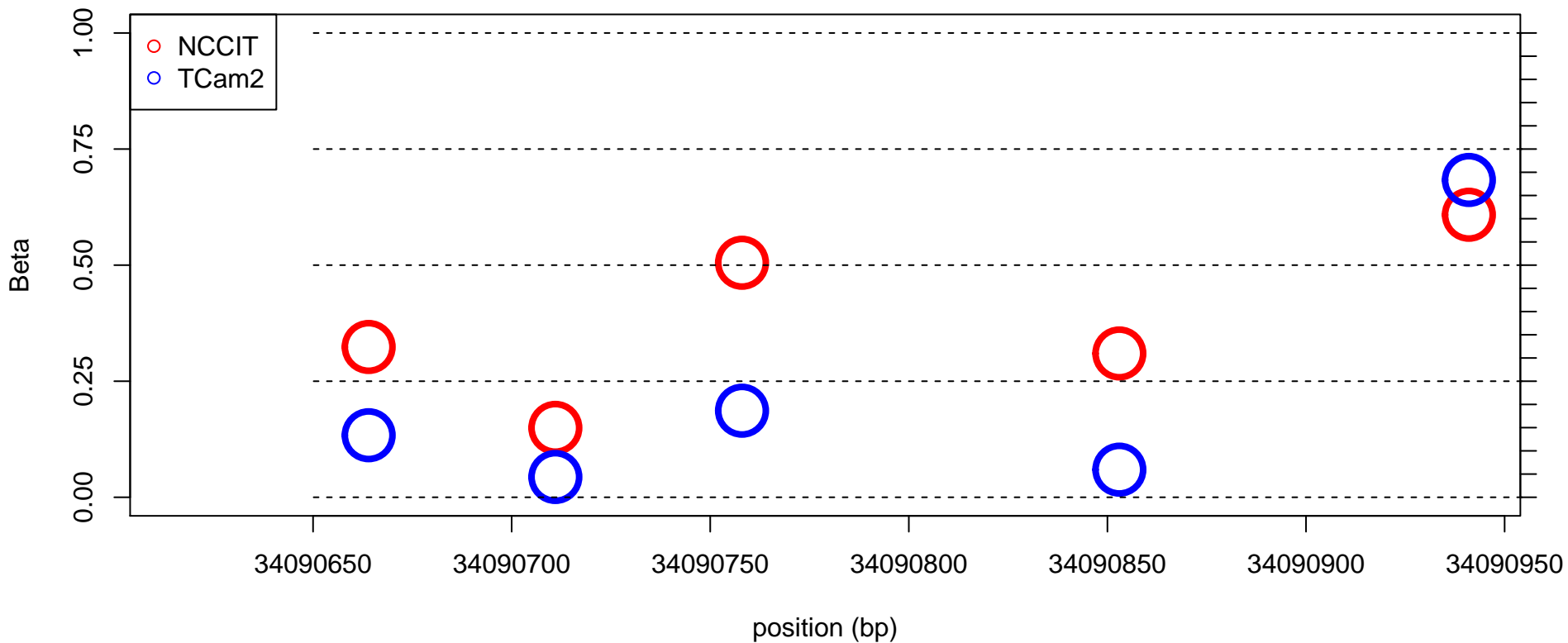

Supplement: File S1 — ZIP file containing DMRforPairs output for significant regions. Please start from the html files. (ZIP) [file pone.0098330.s008.zip › figures/404.pdf]

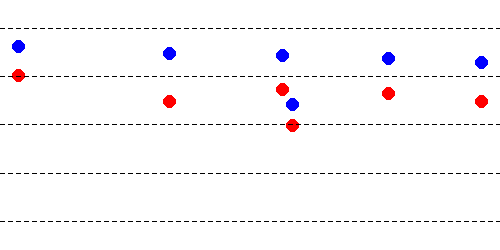

Supplement: File S1 — ZIP file containing DMRforPairs output for significant regions. Please start from the html files. (ZIP) [file pone.0098330.s008.zip › figures/411.png]

RegionID: 411, chr1:35226707-35227272-M\_values

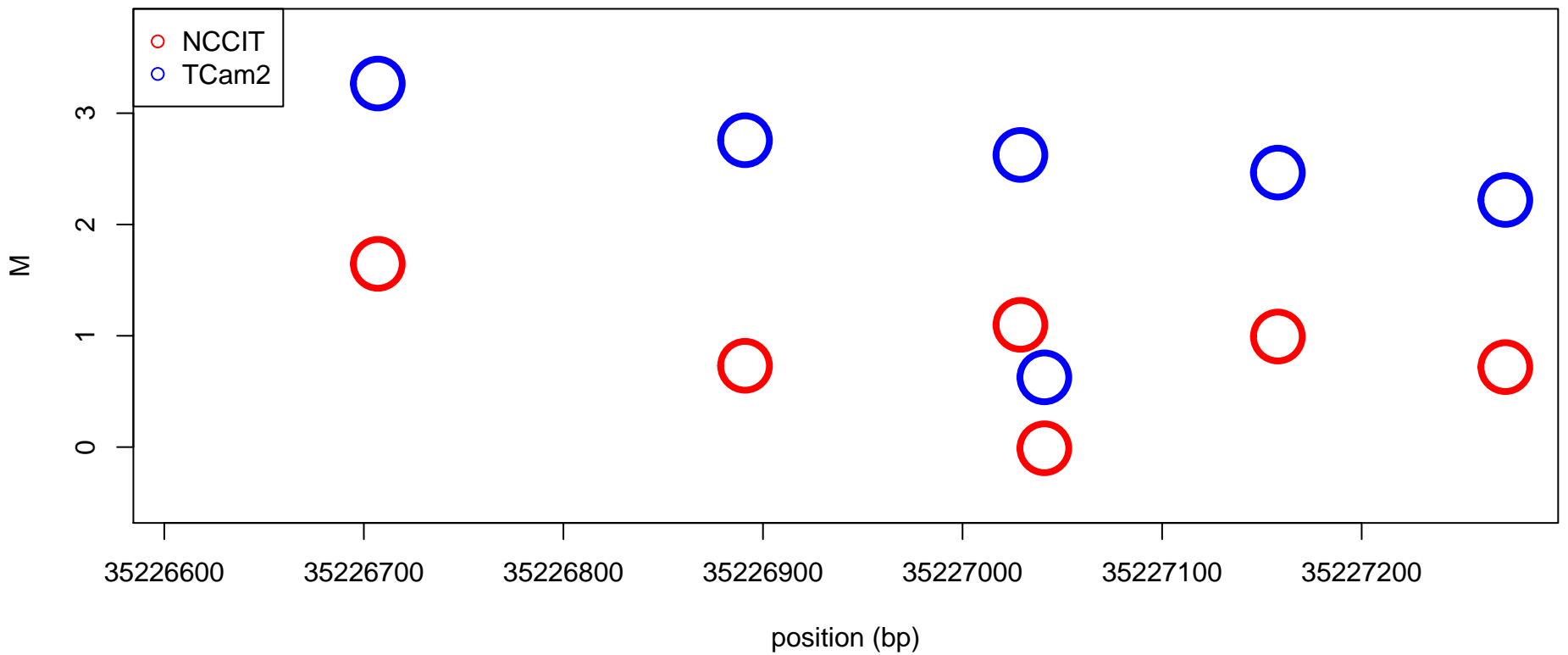

RegionID: 411, chr1:35226707-35227272-Beta\_values

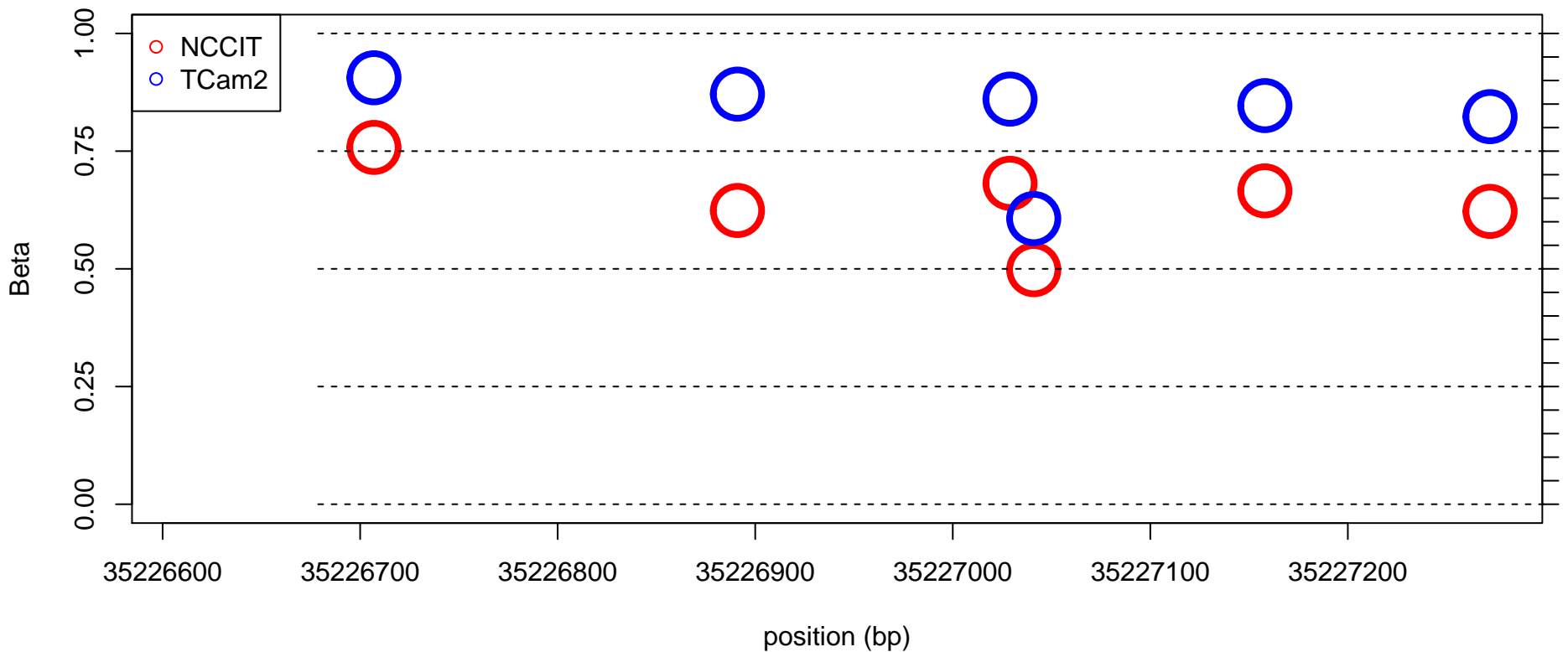

Supplement: File S1 — ZIP file containing DMRforPairs output for significant regions. Please start from the html files. (ZIP) [file pone.0098330.s008.zip › figures/411.pdf]

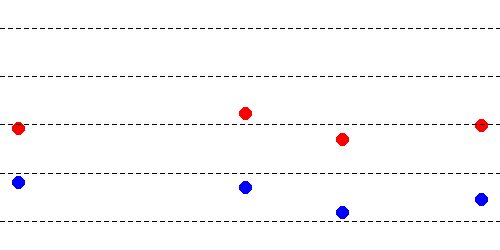

Supplement: File S1 — ZIP file containing DMRforPairs output for significant regions. Please start from the html files. (ZIP) [file pone.0098330.s008.zip › figures/430.png]

RegionID: 430, chr1:37499309–37499649–M\_values

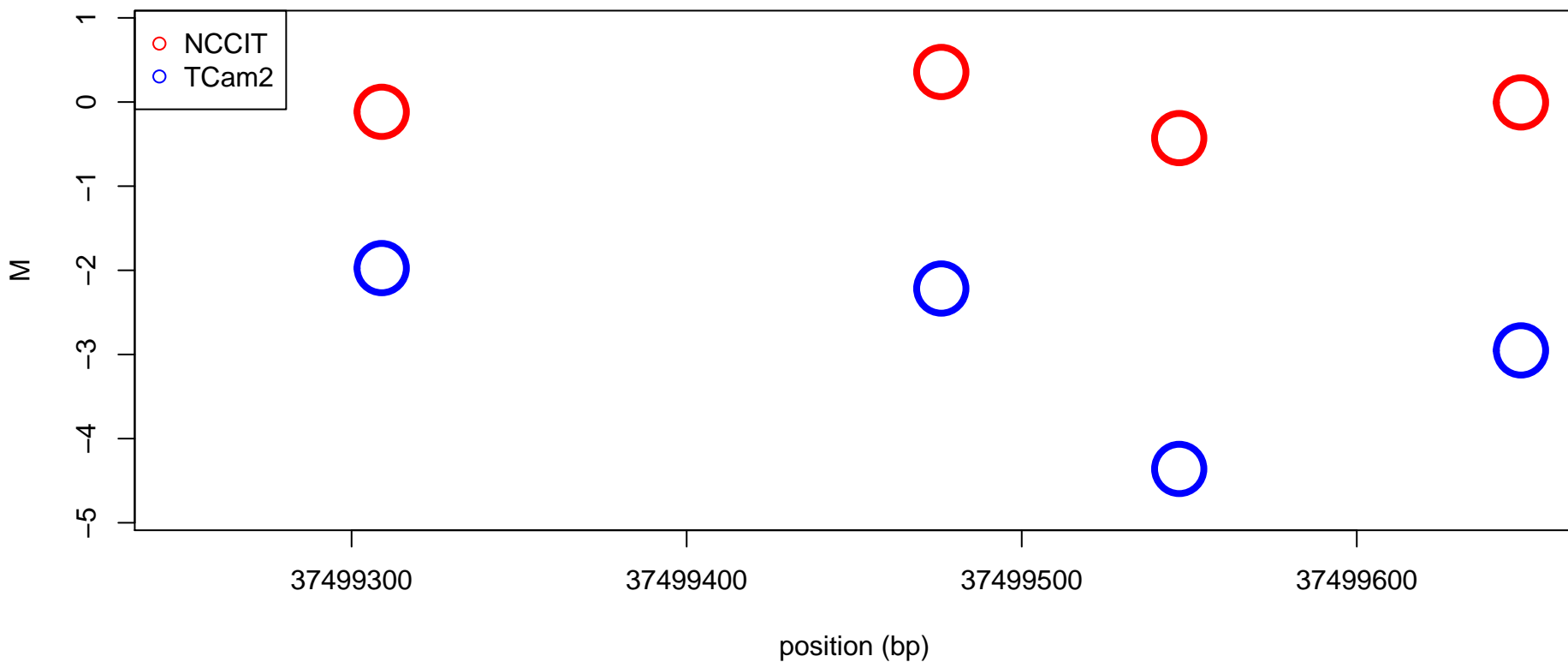

RegionID: 430, chr1:37499309–37499649–Beta\_values

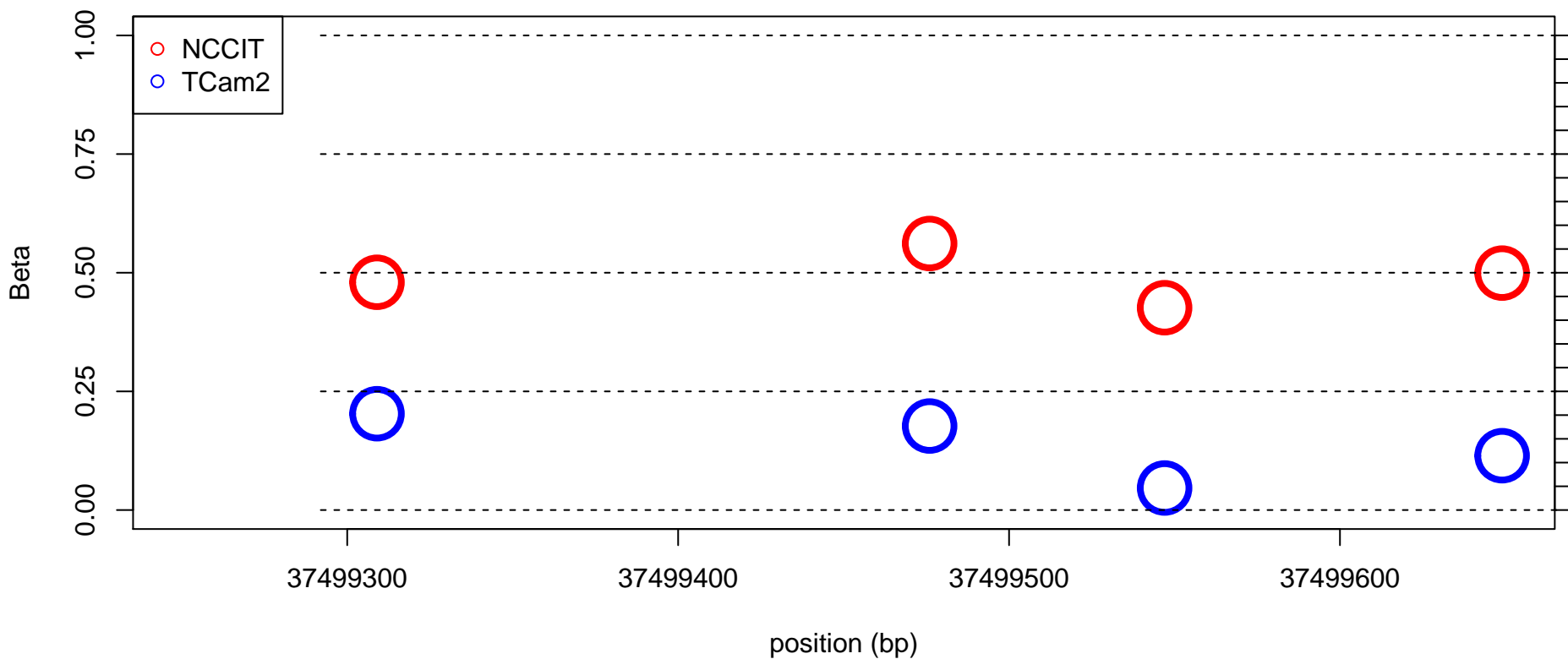

Supplement: File S1 — ZIP file containing DMRforPairs output for significant regions. Please start from the html files. (ZIP) [file pone.0098330.s008.zip › figures/430.pdf]

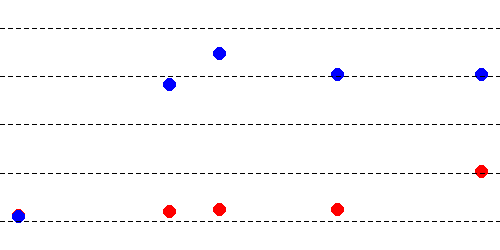

Supplement: File S1 — ZIP file containing DMRforPairs output for significant regions. Please start from the html files. (ZIP) [file pone.0098330.s008.zip › figures/433.png]

RegionID: 433, chr1:38022522-38022718-M\_values

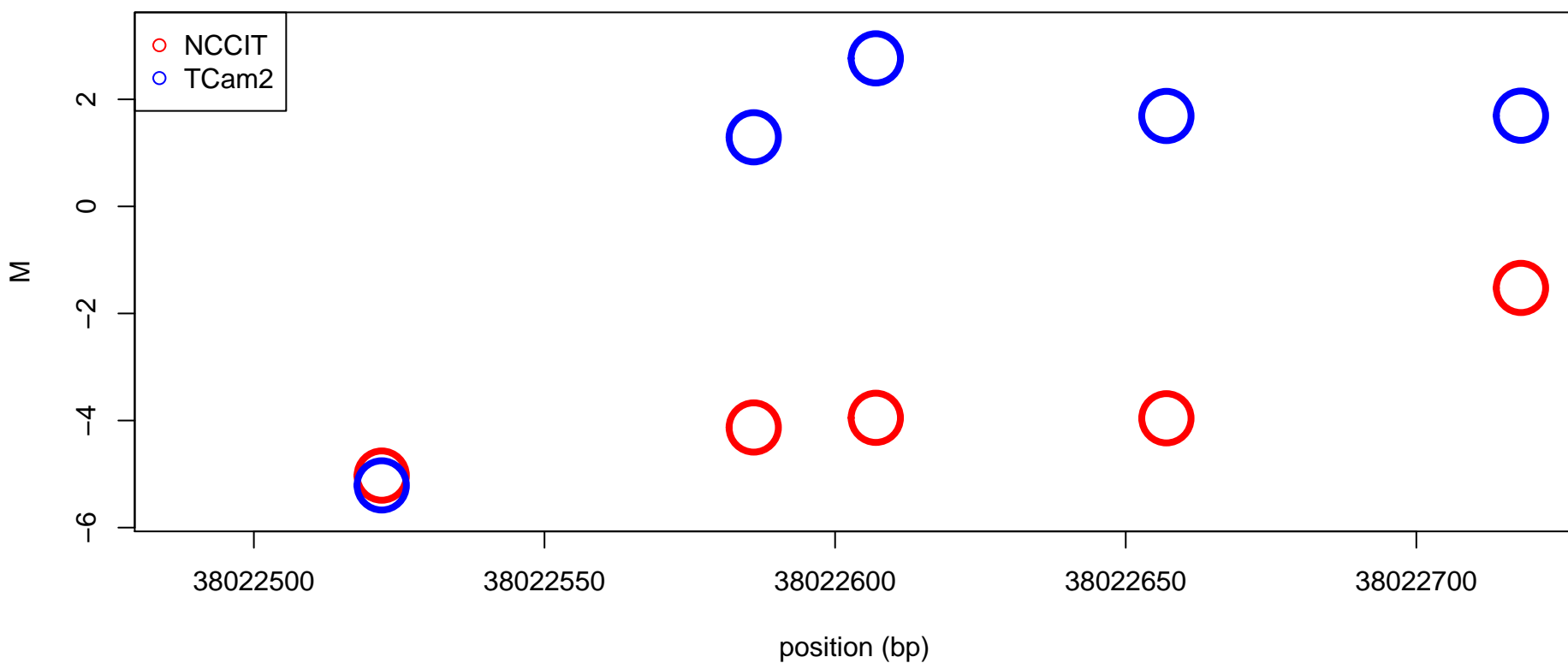

RegionID: 433, chr1:38022522-38022718-Beta\_values

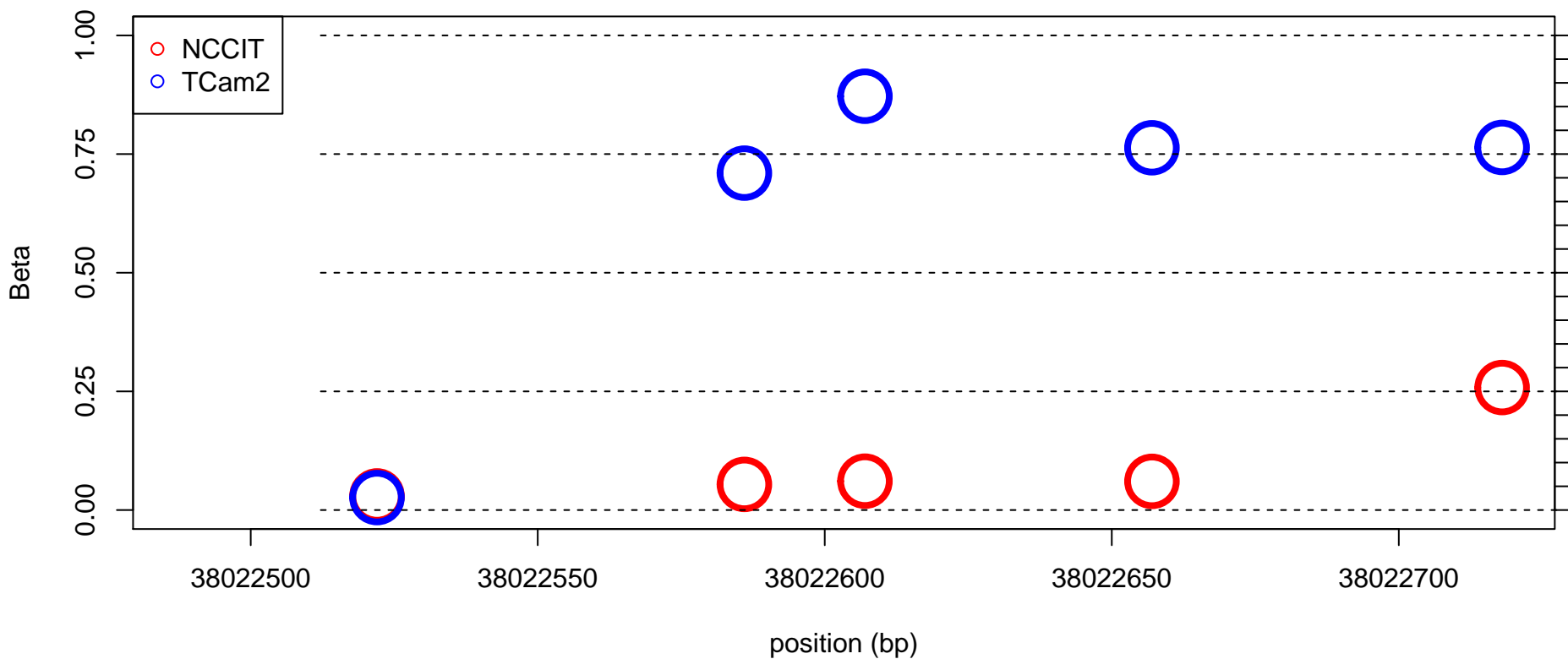

Supplement: File S1 — ZIP file containing DMRforPairs output for significant regions. Please start from the html files. (ZIP) [file pone.0098330.s008.zip › figures/433.pdf]
